# Current and Future Distribution of the Lone Star Tick, *Amblyomma americanum* (L.) (Acari: Ixodidae) in North America

**DOI:** 10.1371/journal.pone.0209082

**Published:** 2019-01-02

**Authors:** Ram K. Raghavan, A. Townsend Peterson, Marlon E. Cobos, Roman Ganta, Des Foley

**Affiliations:** 1 Department of Diagnostic Medicine and Pathobiology, College of Veterinary Medicine, Kansas State University, Manhattan, Kansas, United States of America; 2 Department of Ecology and Evolutionary Biology, College of Liberal Arts and Sciences, The University of Kansas, Lawrence, Kansas, United States of America; 3 Division of Entomology, Walter Reed Army Institute of Research, Silver Spring, Maryland, United States of America; University of Minnesota, UNITED STATES

## Abstract

Acarological surveys in areas outside the currently believed leading edge of the distribution of lone star ticks (*Amblyomma americanum*), coupled with recent reports of their identification in previously uninvaded areas in the public health literature, suggest that this species is more broadly distributed in North America than currently understood. Therefore, we evaluated the potential geographic extent under present and future conditions using ecological niche modeling approach based on museum records available for this species at the Walter Reed Biosystematics Unit (WRBU). The median prediction of a best fitting model indicated that lone star ticks are currently likely to be present in broader regions across the Eastern Seaboard as well as in the Upper Midwest, where this species could be expanding its range. Further northward and westward expansion of these ticks can be expected as a result of ongoing climate change, under both low- and high-emissions scenarios.

## Introduction

The medically significant tick species, *Amblyomma americanum* (Linn.) (Acari: Ixodidae), commonly known as the lone star tick, is distributed widely across the eastern United States. It has been implicated in transmission of several pathogens known to cause morbidity and mortality to humans and companion animals [1, 2]. Many vector-borne disease infections occur within geographic areas that are highly concordant with the range of transmitting, pathogen-infected vector species [3]; this point likely holds true also for infections vectored by lone star ticks. Reports of infections outside such endemic areas may relate to travel from endemic areas or misdiagnosis, but may also indicate range expansion of vector species into new regions.

Currently, the leading edge of the geographic distribution of lone star ticks is considered to lie in the central midwestern United States [4]. The methods used to identify this range limit are not fully clear, and this map is described as providing only broad guidance [4]; it was based on multiple surveys, some dating as far back as the 1940s [4]. This tick probably has expanded and/or shifted its range owing to non-stationary drivers such as climate change and the population explosion in white-tailed deer (*Odocoileus virginianus*) populations. In fact, our own surveys in Kansas, Oklahoma, and Nebraska over the past five years have yielded numerous records of all questing life stages of lone star ticks throughout the tick season (mid-April through mid-August), well outside the “known” distributional area, indicating that the ticks are well established in areas where they were presumed to be absent in the recent past. Our earlier analyses of ecological niches and geographic distributions of lone star ticks in Kansas indicated a westward range expansion compared to the CDC map [5].

Springer et al. (2015) [6] presented an ensemble spatial distribution model for current and future distributions of lone star ticks in the conterminous United States. The western edge of the current distribution described in their study, however, falls well short of areas from which we have routinely collected ticks in Kansas and Nebraska over the years, albeit at relatively low abundances. We note that this model failure is parallel to one on which we have remarked previously, as regards the western limits of the range of *Ixodes scapularis* which was related to erroneous assumptions in the process of model thresholding [7, 8].

Here, we have assessed the spatial distribution of lone star ticks in North America under present climate conditions, with the goal of understanding and anticipating its potential distribution under future climate scenarios. We used ecological niche modeling approaches under two scenarios of greenhouse gas emissions and consequent climate change: Representative Concentration Pathway (RCP) 4.5, representing medium-to-low emissions levels, and RCP 8.5, representing a high emissions scenario. We evaluated these scenarios *via* model outputs from 4 different general circulation models (GCMs) to permit detailed assessment of uncertainty in model predictions.

## Materials and methods

### Tick occurrence data

Locations within North America where lone star ticks have been collected (and positively identified) were made available for this study in digital format by the Walter Reed Biosystematics Unit (WRBU). This dataset had collection records from 1995–2015, and included all life stages of *A*. *americanum* that were questing or attached to military personnel. The ticks were identified morphologically by entomologists using standard taxonomic keys for North American tick identification. Geographic coordinate information was generated previously by WRBU using standard methods, including Biogeomancer (http://www.biogeomancer.org) and the MANIS georeferencing protocol (http://www.manisnet.org). Crucially, the data included uncertainty information in meters for geographic coordinates, summarizing the likely area across which each of the ticks was likely collected. The WRBU data covered the great majority of the currently known geographic distribution of lone star ticks.

The occurrence data were filtered in sequential steps to improve quality. First, records without specific locality information were removed, as well as occurrences either lacking uncertainty information or with uncertainty >10,000 m. Because spatial autocorrelation in environmental information and consequent non-independence of occurrence data could bias predictions [9], we rarefied occurrence data in ArcGIS using the Spatial Distribution Modeling toolbox (SDMToolbox 1.1c) [10]. We explored different filtering distances in sequential steps, visually inspecting results. We aimed to avoid excessive clustering within any particular region, and to attain a uniform distribution of occurrences across the species’ known distribution. Once a satisfactory distribution of occurrence data was obtained (i.e., when spatial clustering of records was no longer evident), we split the locations randomly in equal halves: one set of locations for calibration and another for evaluation during model calibration. The occurrence data used in this study are available in S1 File.

### Environmental data

We used the so-called “bioclimatic” variables in the WorldClim (version 1.4) climate data archive (www.worldclim.org) to summarize climatic conditions. Bioclimatic variables are derived from monthly temperature and precipitation values and are intended to approximate climate dimensions meaningful to biological species [11]. We used climate data at 10’ (~17 x ~17 km) spatial resolution, which more or less matches the uncertainty inherent in the occurrence data. We excluded four of these data layers (mean temperature of wettest quarter, mean temperature of driest quarter, precipitation of warmest quarter, precipitation of driest quarter) because these layers include known spatial artifacts [12]. We selected three sets of environmental variables based on exploration *via* the jackknife procedure in Maxent, wherein different models were constructed with progressively fewer variables, at each step inspecting variable contributions to the overall model [13]. Least contributing variables were removed sequentially, ending in three sets, consisting of 11, 7, and 4 variables.

### Future climate data

To assess future distributional potential of a species, it is crucial to take uncertainty in future distribution estimates into account. One way to consider this uncertainty is to explore variation deriving from analyses using multiple climate models and greenhouse gas emissions scenarios. We considered two emissions scenarios, representing medium low (RCP 4.5) and high (RCP 8.5) concentrations and climate consequences. For each of the two scenarios we explored four general circulation models (GCMs) since different GCMs are known to produce regional differences in future climate projections because they use different data and algorithms [14]. The four GCMs considered in this study were: (1) Climate System Model (CSIRO Mk3), CSIRO Atmospheric Research; (2) Model for Interdisciplinary Research on Climate (MIROC), Center for Climate System Research, University of Tokyo; (3) Community Climate System Model-4 (CCSM4), National Center for Atmospheric Research (NCAR); and (4) Canadian Earth System Model-2 (CanESM2), Canadian Center for Climate Modeling and Analysis (CCCMA). These data were downloaded from the Climate Change, Agriculture and Food Security—Climate Data Portal (http://www.ccafs-climate.org/data_spatial_downscaling/; CCAFS, 2018), at 10’ resolution.

### Ecological niche modeling

#### Model calibration

The ecological niche modeling approaches followed here have been discussed and described in detail generally [15], and specifically for disease-relevant systems [16, 17]. We used MaxEnt 3.4.1 [18] for estimating the climatic niche of lone star ticks across North America. One challenge for such models has traditionally been the task of choosing best parameter values, which has been done “by art,” and only recently via quantitative, rigorous approaches [17]. In this contribution, we take advantage of recent advances in this model calibration protocol following the general example of Muscarella et al., (2014) [19], and use the kuenm package (https://github.com/marlonecobos/kuenm; Cobos et al., in review) in R 3.5.0 (R Core Team, 2018).

Defining adequate accessible areas (**M**) as the area across which model calibration should be executed has been shown to improve model performance [20] by minimizing impacts of assumptions about absence of species from areas that are not accessible to the species. **M** is defined as the area to which the species likely had access via dispersal [21]. We calibrated models across an area within 7° (~770 km) of any of the occurrences, which was considered as a working hypothesis of **M** for the species [21]. Areas outside of the United States were not considered because we had no sampling available from those areas [16]. In all, 1479 candidate models were built under varying combinations of model response types (i.e., all potential combinations of linear, quadratic, product, threshold, and hinge, responses), 17 regularization multiplier values (0.1–1 with intervals of 0.1; 1–6 with intervals of 1; 8 and 10), and three distinct sets of environmental variables. Our aim was to evaluate (1) statistical significance, (2) model performance in anticipating independent subsets of available occurrence data, and (3) complexity of the modeled relationship of occurrences to the environmental data [22] [23].

The candidate models were first reduced based on partial ROC tests [based on a random 50% of occurrence data set aside before model calibration] [24], removing non-significant models from further consideration. We then filtered by omission rate (based on the same subset of occurrence data after an **E** = 5% adjusted training presence threshold was applied), removing all models for which omission rate was >0.05, which was considered as performance that was not adequate for our needs. Finally, we filtered models by lowest values of the Akaike Information Criterion corrected for small sample sizes (AICc) [22], choosing as final models those within 2 AICc units of the minimum among the significant, low-omission candidate models.

#### Final models

We created final models using the selected parameter settings and performing 10 replicates; models were then transferred to Mesoamerica and North America under present-day conditions and to future scenarios based on future climate models and scenarios described above, allowing extrapolation and clamping in Maxent projections. We used the median values across replicates as an estimate of the present-day spatial distribution of suitable and unsuitable conditions for lone star ticks across North America and Mesoamerica. The uncertainty in present-day model predictions was estimated using the range in the suitability values (i.e., maximum minus minimum) across model replicates. From models transferred to future conditions we obtained four results (one per GCM) per RCP scenario. Results for each RCP were maintained separate to represent two scenarios of potential changes of suitable areas. Agreement or lack of agreement among results from different GCMs was used to summarize degree of certainty in future expected changes of suitable areas, following Campbell et al. (2015) [25].

#### Extrapolation risk analysis

The mobility-oriented parity (MOP) [26] metric was used to analyze the novelty of future climate conditions relative to present-day conditions in the calibration area. This analysis helps determine areas where strict extrapolation (i.e., transfer areas with values outside the range of climates in the calibration area) occurs. Areas with higher extrapolative values indicate higher uncertainty; and caution is required when interpreting likelihood of species presence in such areas [27].

## Results

**Fig 1:**
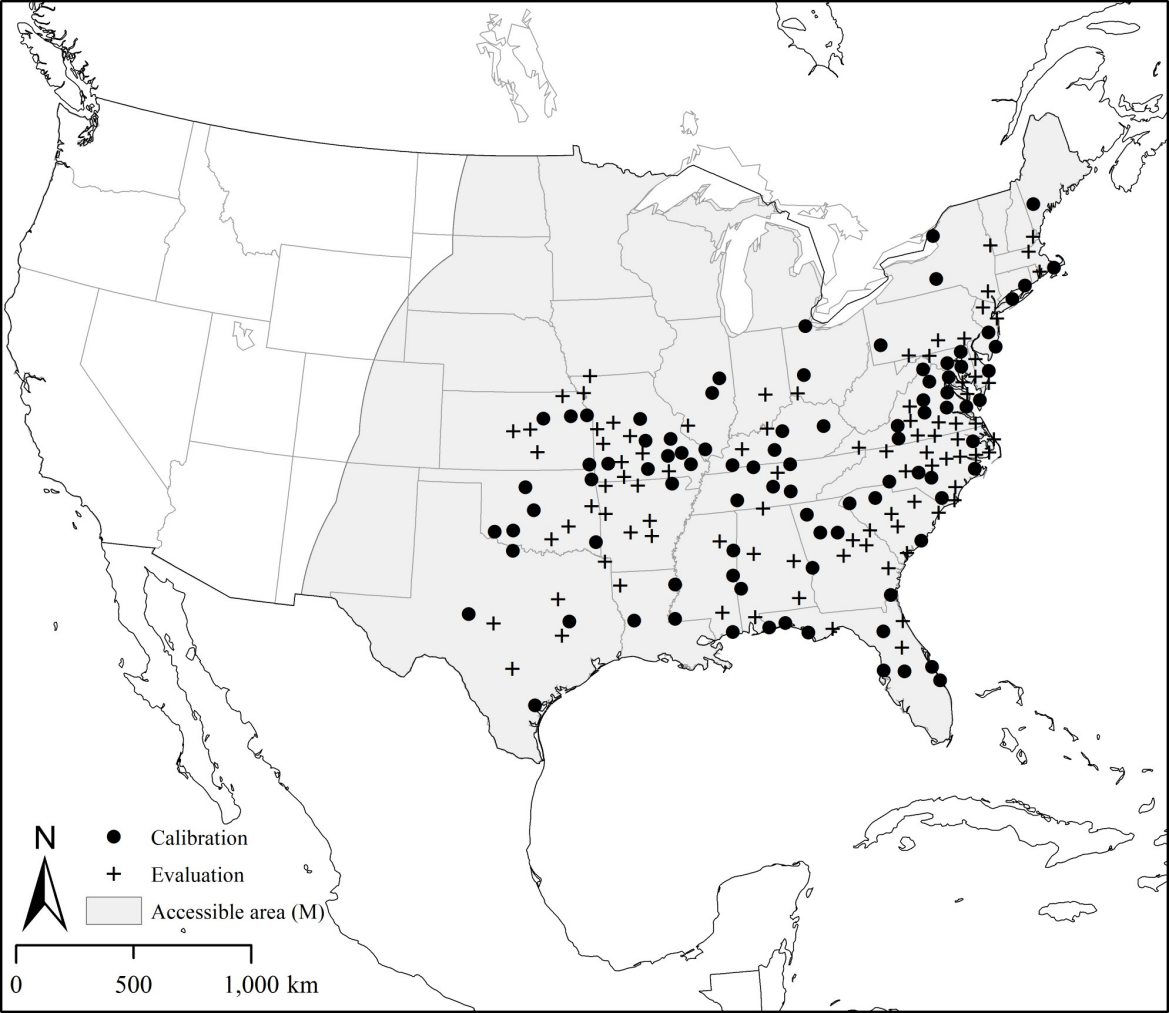
Geographic coordinates representing collection locations of *Amblyomma americanum* and accessible area (M) used in the construction of ecological niche models.

In all, 14,831 occurrence records with geographic coordinate information were available for lone star ticks in the WRBU database. Removing records that lacked uncertainty information or that had uncertainty >10,000 m left us with 6,492 records. A further 33 records were from outside the known range of the species, 31 of which were collected from human subjects reported by the U.S. Army Institute of Public Health, which may include significant recall bias: the human patients may not have remembered where they came in contact with ticks. The two other records, one each submitted by the University of Alberta Entomology Collection and the Australian Museum, lacked metadata that would have allowed objective confirmation of the records. Removal of these 33 locations resulted in 6,459 occurrence records. Four occurrence locations in Minnesota were removed since they likely represented single individuals rather than established populations, and information available in the WRBU database did not allow us to confirm these occurrences with high confidence. Finally, occurrence data were rarefied to an inter-occurrence separation of ≥50 km, which resulted in 181 presence-only locations for analysis (Fig 1; S1 File). S2 File includes all occurrences and corresponding uncertainty radii.

The initial jackknife analysis indicated removal of annual mean temperature (Bio1), mean temperature of the coldest quarter (Bio11), precipitation of the driest month (Bio14), isothermality (Bio3), and temperature seasonality (Bio4) as they did not affect model performance noticeably. In the second jackknife step, temperature annual range (Bio7), and minimum temperature of the coldest month (Bio6) were the variables that contributed the least. In two subsequent steps, precipitation of the wettest quarter (Bio16) and precipitation of the driest quarter (Bio17), and precipitation of the wettest month (Bio13) and mean temperature of the warmest quarter (Bio10) were identified as least important. Variables always retained were annual precipitation (Bio12), precipitation seasonality (Bio15), mean diurnal range (Bio2) and maximum temperature of warmest month (Bio5).

In model calibration, we assessed 1,479 models, all of which were statistically significant as compared with a null model of random prediction. Of these significant models, 15 (1%) met the omission criterion of 5%. Finally, of the significant, low-omission models, the model with the minimum AICc value was one with a regularization parameter value of 0.5; linear and quadratic features; and the second set of environmental variables. AICc values of all other models were higher by more than at least 2 units, so we explored this model as to its implications for present and future potential distributions of lone star ticks (see the complete table of model evaluation results in S3 File).

**Fig 2:**
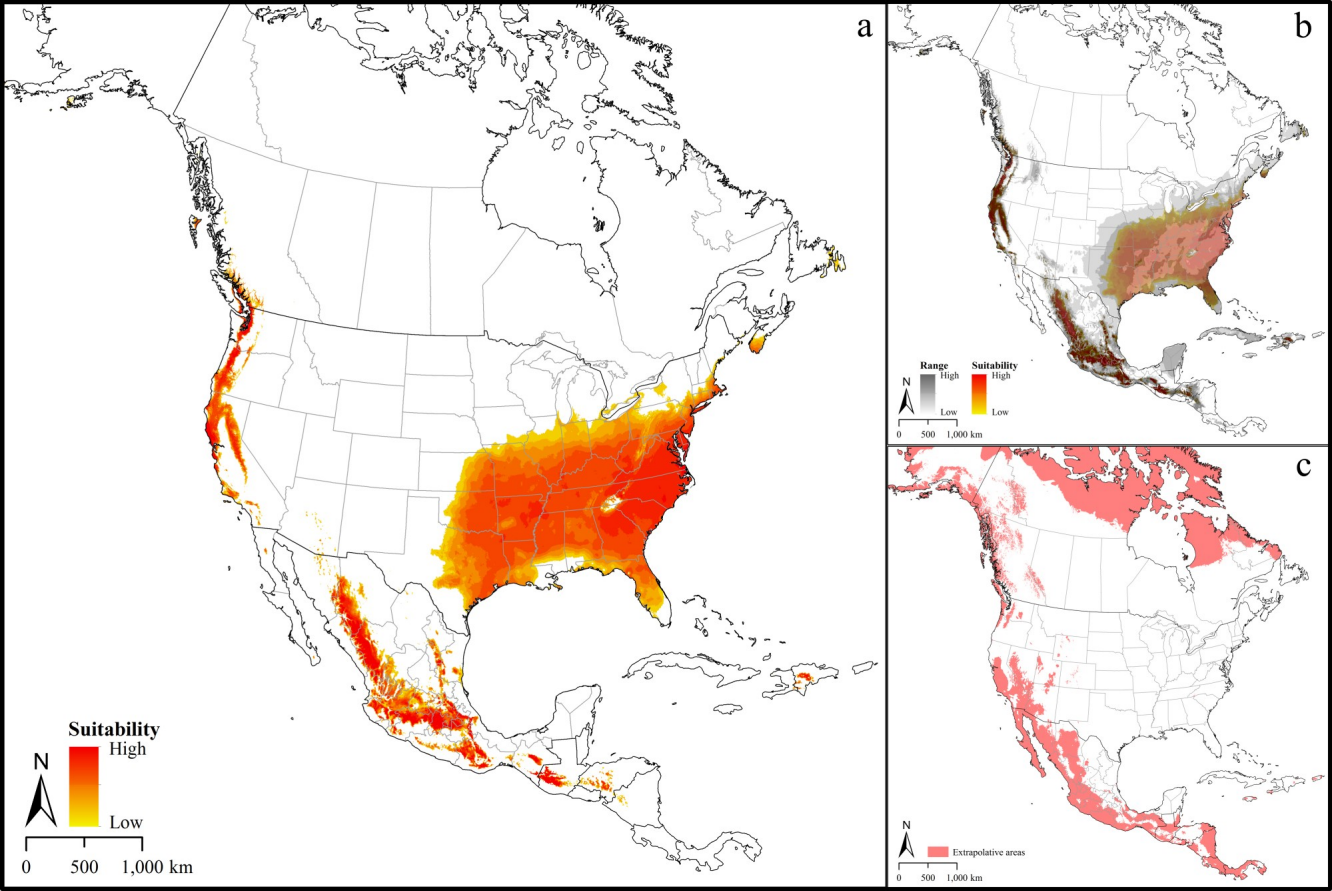
Climatologically suitable regions for *Amblyomma americanum* distribution in North America. a. Median prediction. b. Uncertainty associated with the median prediction of suitable regions for *Amblyomma americanum* in North America. c. Mobility-oriented parity analysis revealing areas in N. America for which the present-day model predictions are strictly based on extrapolation.

The median of the selected model (Fig 2A) identified areas with different levels of suitability for lone star ticks across North America. Highly suitable areas were concentrated in the lower Midwest and South, and suitability declined towards the central United States and southeastern Canada. Broad areas in northern California and the west coast of Oregon, Washington and British Columbia were suitable as were disparate areas in Mexico, with suitable areas extending into northern Central America. Much of the Caribbean was also identified by this model as highly suitable, although it may not be accessible to the species. The present-day model showed low uncertainty throughout the predicted distributional area (Fig 2B), and the MOP analysis revealed that only the southwestern United States and northern Mexico would present model extrapolation in current conditions (Fig 2C).

**Fig 3:**
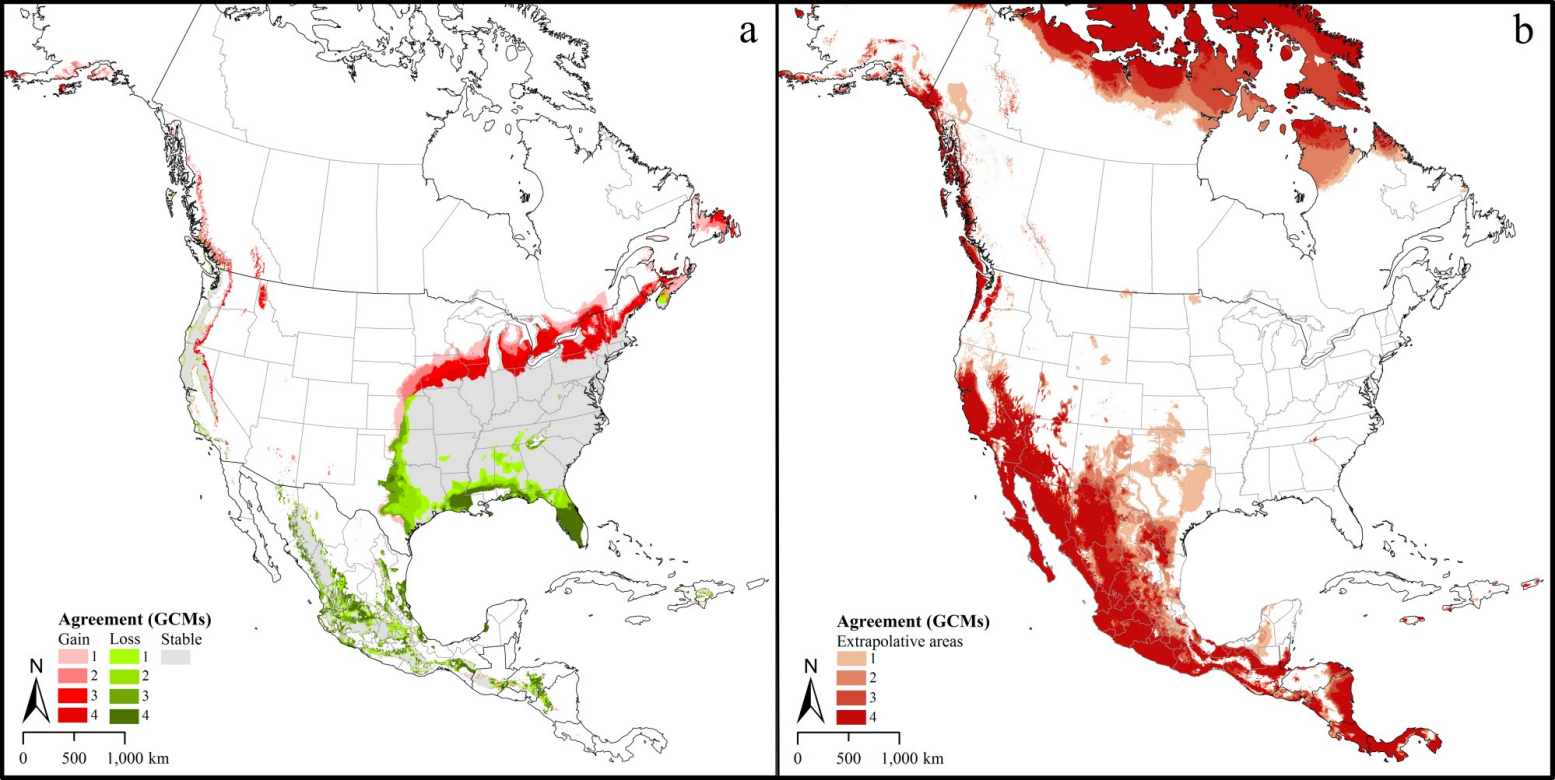
Predicted future suitable regions under climate change scenarios. a. Predictions under the Representative Concentration Pathway (RCP) 4.5, and agreement between different Global Circulation Models. 1 = areas in which one of the four GCMs predicted suitability for *Amblyomma americanum* distribution. 2, 3, 4 = two, three and four GCMs predicted suitability, respectively. 5 = areas in which one of the four GCMs predicted loss of territory for *A*. *americanum* compared to the present-day distribution. 6, 7, 8 = two, three, and four GCMs predicted loss of territory, respectively. b. Agreement among Global Circulation Models (GCMs) in North America with strict extrapolative conditions under the low emissions, RCP 4.5 scenario. 1 = Areas in which the prediction based on one of the four Global Circulation Models (GCMs) were strictly extrapolative. 2, 3, 4 = degree of agreement in strict extrapolative areas based on two, three and all four models, respectively.

**Fig 4:**
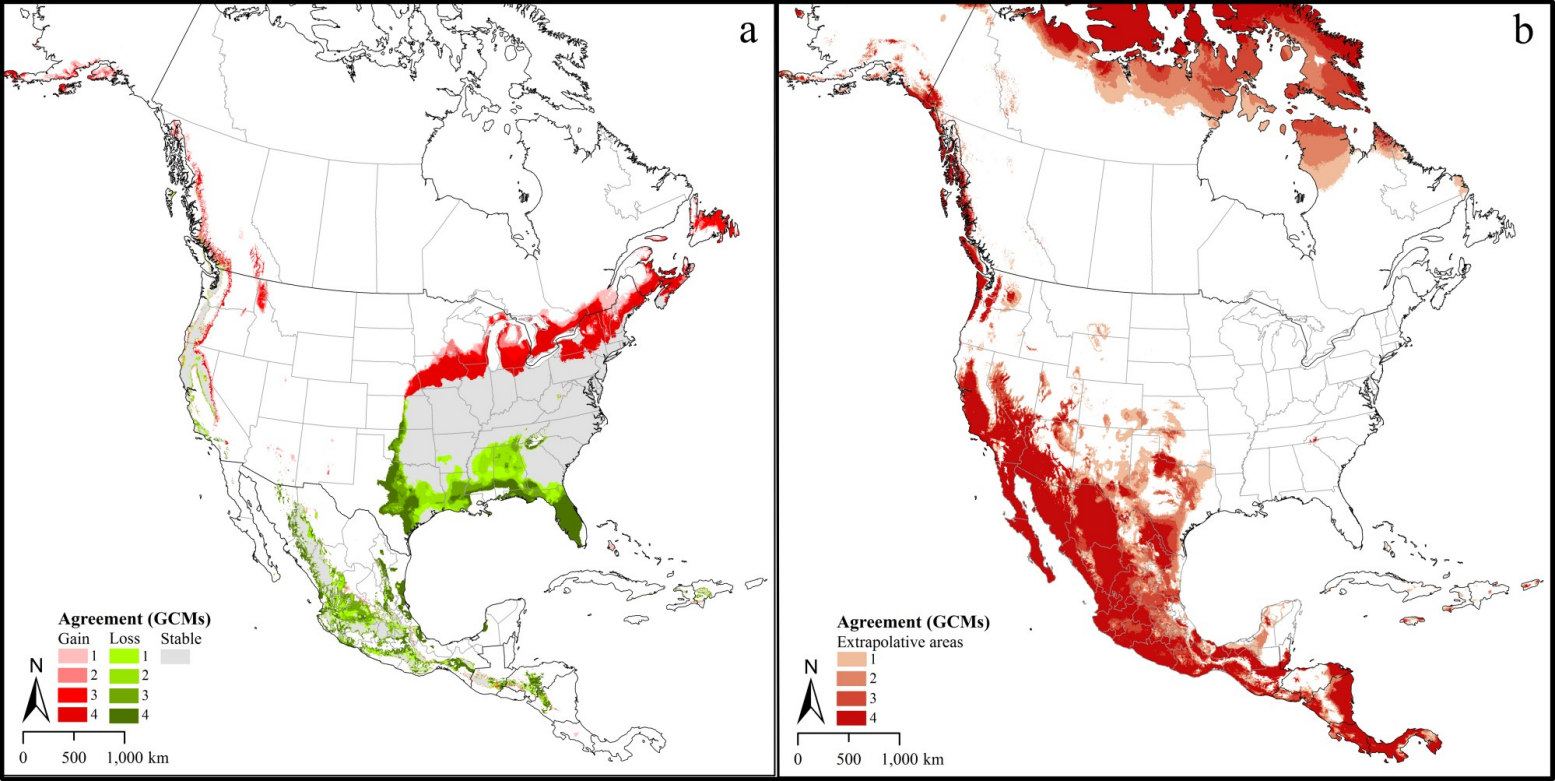
Predicted suitable distribution under climate change scenarios. a. a. Predictions under the Representative Concentration Pathway (RCP) 8.5 and agreement between different Global Circulation Models (GCMs). 1 = areas in which one of the four GCMs predicted suitability for *Amblyomma americanum* distribution. 2, 3, 4 = two, three and four GCMs predicted suitability, respectively. 5 = areas in which one of the four GCMs predicted loss of territory for *A*. *americanum* compared to the present-day distribution. 6, 7, 8 = two, three, and four GCMs predicted loss of territory, respectively. b. Agreement among Global Circulation Models (GCMs) in North America with strict extrapolative conditions under the high emissions, RCP 8.5 scenario. 1 = Areas in which the prediction based on one of the four Global Circulation Models (GCMs) were strictly extrapolative. 2, 3, 4 = degree of agreement in strict extrapolative areas based on two, three and all four models, respectively.

The models identified a future potential distribution that reflected dramatic expansion of the distributional potential of lone star ticks from the present distribution. Composite maps (i.e., across the four GCMs) for RCP 4.5 (Fig 3A and 3B) and RCP 8.5 (Fig 4A and 4B) both indicated potential range expansion well into the upper Midwest, southeastern Canada, and contiguous areas of the western United States. Predictions from different GCMs differed, as can be appreciated from the different levels of agreement in Figs 3A and 4A. A predicted potential range retraction in Mexico is rather less certain, as our occurrence data were exclusively from the United States and Canada and did not represent the Mexican part of the species’ range. The MOP analysis of future climates for both scenarios indicated that model predictions for broad areas in southern California, Mexico, and Central America were based on extrapolative conditions (Figs 3B and 4B).

## Discussion

Ecological niche models have been used widely to describe the spatial distributions of medically important vector species, such as lone star ticks. This study applied many of the recent methodological recommendations to produce robust ecological niche models, which included filtering and thinning occurrences [9, 28], testing distinct candidate sets of environmental variables, testing multiple combinations of model parameters (features, regularization multipliers, and sets of variables) [29], using an accessible area for model calibration [21], using multiple statistical criteria (partial ROC, omission rates, and AICc) for model selection, and accounting for model uncertainty in current and future predictions [26].

The models produced in this study indicate that the distribution of *A*. *americanum* is largely restricted to the east and central parts of the US. We used a 5% threshold for suitability values in our final models, which allows for some known occurrences to be omitted from the prediction, refining the final predicted potential distribution [30] and avoiding undue effects of erroneous localities that may be present. The prediction (Fig 2B) identified areas closer to the known distributional areas that are likely to be climatically suitable for *A*. *americanum* if the species is able to disperse to these areas. Previous efforts to characterize the potential distribution of this tick [6, 31] appear not to have considered model variability, so uncertainty in their predictions cannot be assessed. Further, our model transfers to future conditions considered two RCP scenarios, representing medium-low and high emissions scenarios. Our mobility-oriented parity analysis revealed areas of strict extrapolation in model projections in North America. Even though these areas may prove to be suitable, the absence of occurrence data from these area and low level of similarity to the areas where the models were calibrated (**M**) renders interpreting suitability in these extrapolated areas difficult.

From a public health standpoint, evaluating the current and future spatial distribution of lone star ticks in North America is important so that the geographic picture of disease risk posed by this species under current and future climatic conditions can be appreciated. The medical significance of this tick species has been discussed amply [1, 32]: lone star ticks are the most frequently encountered ticks by humans in some parts of the US, particularly in the southern and central Midwest [33, 34]. This species is a growing public health concern since spatiotemporal dimensions of several lone star tick-transmitted diseases appear to be worsening [35–37]. Lone star ticks are well-known as a vector of human ehrlichiosis [38] and Southern Tick Associated Rash Illness [39], but recent laboratory and field investigations have confirmed that the recently discovered human pathogens Heartland Virus (HRTV) and Bourbon Virus (BRBV) are also transmitted by nymphs of this species [40–42]. Levin et al. (2015) [43] recently showed that lone star ticks are competent, at least under laboratory conditions, to maintain and transmit *Rickettsia rickettsii*, the causative agent for Rocky Mountain spotted fever; and, the increase in incidences of spotted fever group (SFG) rickettsiosis in the US is suspected to be related to the expansion of lone star ticks [44]. Finally, a rapidly emerging and potentially fatal illness, commonly referred as red meat allergy (alpha-gal syndrome), is a condition that affects some individuals bitten by ticks—they develop anaphylaxis following red meat consumption—the tick responsible for this poorly understood malady in North America is most likely the lone star tick [45].

Considering the medical significance of lone star ticks, their broad host range, and the relative abundances at which they are at times found [1, 32], the present-day distributional pattern estimated in this study (Fig 2) points to a major public health problem. That is, this distributional estimate exceeds the mapped suitable area presented previously by CDC [4] and Springer et al (2015) [6] in all directions inland; as such, the model indicates that the ticks may have distributional potential in the upper Midwest (Wisconsin, Michigan and possibly also Minnesota). Lone star ticks have been collected occasionally in Wisconsin over the past few years, particularly in the southern parts of the state (Russell Lab, 2018) [46]; the Minnesota Department of Health indicates that these ticks have been collected in Minnesota [47], and similarly in Michigan [48], all in recent years. It is not clear if lone star ticks have long been established in this region, or if a range expansion process has occurred or is occurring. Our experience in Nebraska, Kansas, and Oklahoma is that these ticks occur in areas well west of the traditional boundary; correspondingly, incidence of human ehrlichiosis has apparently increased [37]. We note that the previous modeling and mapping efforts regarding species may have fallen into the same methodological traps as the CDC-associated team that worked with *Ixodes scapularis* [49], which similarly led to massive range under-estimation, particularly in areas of low-density occurrence. Our present-day model also indicated vast areas of California as climatically suitable for lone star ticks; however, current knowledge of tick communities in California (e.g., California Ticks, 2018) [50] suggests that these areas are free of this species, likely thanks to the effects of barriers to dispersal.

The future potential distribution of the lone star ticks under high and low emissions scenarios considered here indicate marked expansions of lone star tick range, and particularly northward expansion of suitable areas for this tick is clearly evident. The relevance of climate change to distributional shifts and range expansions of vector species have been discussed widely (e.g., [51] [52]) but they must be considered along with complex interactions and other non-stationary forces [53–55]. Nevertheless, ticks are ectothermic, can adjust to temperature fluctuations by moving around vegetation and leaf litter; eventually however, their geographic distribution, phenology, and the diseases they transmit are likely to change as a result of ongoing climate change (e.g., [56, 57]). Evidence of such changes in abundance and shifts in the distribution of another tick species, *Ixodes ricinus* towards northern latitudes in Europe have been reported (e.g., [58–60]) in general agreement with model predictions of Alkishe et al., 2017 [27], and climate change has been strongly suggested to be one of the leading factors in this ongoing dynamic. The increasing occurrence of lone star ticks in the upper midwestern states of Minnesota, Wisconsin, and Michigan may be an early indication of climate change-mediated northward shifts. Acarological surveys and surveillance of lone star tick-borne diseases in these states will aid in informing long-term public health policy and towards anticipating climate change implications for ticks and tick-borne diseases.

In this study, we used a rigorous modeling approach for approximating ecological niches of the tick species in question, and then assessing its distributional potential under current and future conditions. It is important, however, to recognize that the models presented here do not necessarily represent the population densities of lone star ticks at different sites, and rather focus on potential presence or absence of the species. Population densities of lone star tick are highly heterogeneous, resulting in diverse levels of risk for humans and other hosts [61]. Quantifying region-specific abundances and their spatial heterogeneity will be an important next step from a public health standpoint in efforts to characterize risk of contracting diseases transmitted by this tick.

## Supporting information

S1 File Occurrence locations used for calibrating niche models. (DOCX)

S2 File All occurrence locations with corresponding error radii. (DOCX)

S3 File Results of candidate model evaluation filtered according model selection criteria. (DOCX)
