# Supplementary material for: Current and Future Distribution of the Lone Star Tick, Amblyomma americanum (L.) (Acari: Ixodidae) in North America
Source: PLoS One. 2019 Jan 2;14(1):e0209082. doi: 10.1371/journal.pone.0209082 (PMC6314611; doi:10.1371/journal.pone.0209082)
Supplement: S1 File — (DOCX) [file pone.0209082.s001.docx]

Species Longitude Latitude

Amblyomma americanum -70.54528 44.21389

Amblyomma americanum -76.1302032 44.0670119

Amblyomma americanum -73.32806 43.17222

Amblyomma americanum -71.0742302 43.0334187

Amblyomma americanum -71.5109804 42.5375919

Amblyomma americanum -76.4966087 42.44063

Amblyomma americanum -71.2647679 41.6772631

Amblyomma americanum -83.5552101 41.6639366

Amblyomma americanum -70.521389 41.658611

Amblyomma americanum -74.0843672 41.5015441

Amblyomma americanum -72.1931343 41.328896

Amblyomma americanum -72.9934926 40.9669994

Amblyomma americanum -74.5435553 40.9548927

Amblyomma americanum -94.658661 40.561808

Amblyomma americanum -79.9805035 40.4313764

Amblyomma americanum -74.0074043 40.4037189

Amblyomma americanum -77.128315 40.1498127

Amblyomma americanum -88.2072 40.1106

Amblyomma americanum -75.8238335 39.98316

Amblyomma americanum -74.5829353 39.9726162

Amblyomma americanum -95.0066 39.91867

Amblyomma americanum -96.0641632 39.8341675

Amblyomma americanum -78.71833 39.82306

Amblyomma americanum -84.01944 39.82083

Amblyomma americanum -77.72028 39.64167

Amblyomma americanum -88.6493 39.5843

Amblyomma americanum -76.1646169 39.5095539

Amblyomma americanum -74.4292512 39.3713841

Amblyomma americanum -86.040556 39.290278

Amblyomma americanum -76.9430389 39.2064934

Amblyomma americanum -78.1633339 39.1856613

Amblyomma americanum -84.45694 39.16194

Amblyomma americanum -75.5418663 39.1134472

Amblyomma americanum -94.88358 39.05973

Amblyomma americanum -95.67778 39.04833

Amblyomma americanum -97.02222 38.97222

Amblyomma americanum -76.3133466 38.9539793

Amblyomma americanum -92.28506 38.8297

Amblyomma americanum -93.60188 38.74275

Amblyomma americanum -77.99555 38.67151

Amblyomma americanum -77.0283089 38.6676178

Amblyomma americanum -97.6744843 38.5690774

Amblyomma americanum -75.05518 38.53956

Amblyomma americanum -94.41583 38.51557

Amblyomma americanum -98.50063 38.50029

Amblyomma americanum -75.7591019 38.4598427

Amblyomma americanum -89.9851536 38.4403309

Amblyomma americanum -76.4340987 38.3623328

Amblyomma americanum -92.8335228 38.1991978

Amblyomma americanum -77.2750854 38.1097082

Amblyomma americanum -75.2038 38.09179

Amblyomma americanum -78.4766808 38.0293045

Amblyomma americanum -86.1694145 37.9899147

Amblyomma americanum -92.0937767 37.982841

Amblyomma americanum -90.8700101 37.9736648

Amblyomma americanum -94.15417 37.95611

Amblyomma americanum -79.1975 37.90611

Amblyomma americanum -76.34772 37.88319

Amblyomma americanum -85.4668999 37.8092308

Amblyomma americanum -83.4612923 37.7720337

Amblyomma americanum -97.3375435 37.692234

Amblyomma americanum -77.4602623 37.5537605

Amblyomma americanum -78.55556 37.55015

Amblyomma americanum -75.82333 37.53167

Amblyomma americanum -92.2662055 37.5102341

Amblyomma americanum -89.2470245 37.4603233

Amblyomma americanum -76.5255089 37.4137516

Amblyomma americanum -90.39195 37.40785

Amblyomma americanum -79.29 37.36361

Amblyomma americanum -91.06252 37.33234

Amblyomma americanum -87.4988899 37.3280983

Amblyomma americanum -79.9414253 37.2709694

Amblyomma americanum -93.2982445 37.2153263

Amblyomma americanum -93.94337 37.1741

Amblyomma americanum -94.8456651 37.1701488

Amblyomma americanum -85.95694 37.13667

Amblyomma americanum -77.9972267 37.0804272

Amblyomma americanum -89.99547 36.93682

Amblyomma americanum -77.20278 36.88889

Amblyomma americanum -92.0603 36.88254

Amblyomma americanum -80.027761 36.774436

Amblyomma americanum -79.1027946 36.756525

Amblyomma americanum -91.08047 36.7544

Amblyomma americanum -88.03589 36.74891

Amblyomma americanum -81.95966 36.72448

Amblyomma americanum -76.2802426 36.7081798

Amblyomma americanum -93.2185173 36.6436729

Amblyomma americanum -78.31089 36.600921

Amblyomma americanum -94.7691154 36.593689

Amblyomma americanum -87.0655556 36.5822678

Amblyomma americanum -85.31056 36.50778

Amblyomma americanum -80.7062227 36.3972359

Amblyomma americanum -97.912291 36.3413029

Amblyomma americanum -94.11833 36.33194

Amblyomma americanum -92.5971146 36.2789593

Amblyomma americanum -77.28472 36.27389

Amblyomma americanum -90.97333 36.26361

Amblyomma americanum -85.95167 36.25222

Amblyomma americanum -76.6077232 36.0598271

Amblyomma americanum -78.8572921 36.0512598

Amblyomma americanum -75.6329029 35.9569047

Amblyomma americanum -86.25027 35.75035

Amblyomma americanum -78.01 35.64528

Amblyomma americanum -77.3663521 35.6126633

Amblyomma americanum -94.8210564 35.5803719

Amblyomma americanum -76.6229935 35.5401669

Amblyomma americanum -76.0003393 35.5096615

Amblyomma americanum -78.73918 35.5071

Amblyomma americanum -80.00032 35.50069

Amblyomma americanum -85.46108 35.47507

Amblyomma americanum -97.5164299 35.4675617

Amblyomma americanum -87.99528 35.38694

Amblyomma americanum -79.4169731 35.3459873

Amblyomma americanum -94.1779421 35.2607508

Amblyomma americanum -80.8431282 35.2270889

Amblyomma americanum -78.8881989 35.0527

Amblyomma americanum -76.82549 35.02572

Amblyomma americanum -86.8469429 34.9696042

Amblyomma americanum -92.1395874 34.9039529

Amblyomma americanum -95.923889 34.826667

Amblyomma americanum -81.6237068 34.7154102

Amblyomma americanum -82.837368 34.6834393

Amblyomma americanum -98.4835099 34.6823508

Amblyomma americanum -99.3339729 34.6381245

Amblyomma americanum -77.8957481 34.5104348

Amblyomma americanum -93.055 34.50361

Amblyomma americanum -84.87575 34.50336

Amblyomma americanum -96.7211 34.3486

Amblyomma americanum -92.0857009 34.3258372

Amblyomma americanum -79.87639 34.29972

Amblyomma americanum -78.6154481 34.2141988

Amblyomma americanum -94.67556 34.19389

Amblyomma americanum -78.06721 34.03045

Amblyomma americanum -81.033777 34.0008472

Amblyomma americanum -98.4933891 33.9137115

Amblyomma americanum -88.9992218 33.8984451

Amblyomma americanum -84.3879852 33.7489948

Amblyomma americanum -78.8936673 33.6948946

Amblyomma americanum -83.6035042 33.6531773

Amblyomma americanum -82.1306725 33.5337467

Amblyomma americanum -88.4272652 33.4956722

Amblyomma americanum -80.855648 33.4918175

Amblyomma americanum -94.290393 33.434429

Amblyomma americanum -87.52503 33.28955

Amblyomma americanum -82.976265 33.2757015

Amblyomma americanum -82.4112358 33.0015373

Amblyomma americanum -85.76361 32.83111

Amblyomma americanum -79.93111 32.77639

Amblyomma americanum -83.50018 32.75042

Amblyomma americanum -88.555418 32.551715

Amblyomma americanum -93.6520767 32.5049515

Amblyomma americanum -84.9670713 32.4614086

Amblyomma americanum -80.6701655 32.4324804

Amblyomma americanum -91.187 32.408

Amblyomma americanum -96.4667 32.05

Amblyomma americanum -88.2632 32.01961

Amblyomma americanum -81.6164822 31.9937096

Amblyomma americanum -100.4370384 31.4637737

Amblyomma americanum -85.7496724 31.3992165

Amblyomma americanum -95.9718 31.1984

Amblyomma americanum -89.201237 31.1896126

Amblyomma americanum -99.33472 31.135

Amblyomma americanum -93.1104732 31.1310384

Amblyomma americanum -91.29944 31.10444

Amblyomma americanum -81.72278 30.96361

Amblyomma americanum -87.7730446 30.8829632

Amblyomma americanum -96.3167 30.6667

Amblyomma americanum -86.495307 30.522641

Amblyomma americanum -87.2201747 30.4287072

Amblyomma americanum -88.8271134 30.4167174

Amblyomma americanum -84.4887886 30.0599241

Amblyomma americanum -85.5486378 30.0445485

Amblyomma americanum -81.40989 29.91218

Amblyomma americanum -82.3248253 29.6516342

Amblyomma americanum -98.49333 29.42389

Amblyomma americanum -81.66583 28.92917

Amblyomma americanum -82.59194 28.19361

Amblyomma americanum -97.50889 28.03639

Amblyomma americanum -81.73306 28.02194

Amblyomma americanum -80.55 28

Amblyomma americanum -80.32583 27.44639
