# Supplementary material for: Current and Future Distribution of the Lone Star Tick, Amblyomma americanum (L.) (Acari: Ixodidae) in North America
Source: PLoS One. 2019 Jan 2;14(1):e0209082. doi: 10.1371/journal.pone.0209082 (PMC6314611; doi:10.1371/journal.pone.0209082)
Supplement: S2 File — (DOCX) [file pone.0209082.s002.docx]

ScientificName Species VerbatimScientificName DecimalLongitude DecimalLatitude CoordinateUncertaintyInMeters

Amblyomma americanum americanum A. americanum -88.03589 36.74891 37000

Amblyomma americanum americanum A. americanum -76.1646169 39.5095539 5104

Amblyomma americanum americanum A. americanum -76.1646169 39.5095539 5104

Amblyomma americanum americanum A. americanum -76.2791252 39.4623318 5062

Amblyomma americanum americanum A. americanum -77.0283089 38.6676178 5062

Amblyomma americanum americanum A. americanum -87.0655556 36.5822678 20000

Amblyomma americanum americanum A. americanum -77.0722032 38.7804695 3686

Amblyomma americanum americanum A. americanum -77.0722032 38.7804695 3686

Amblyomma americanum americanum A. americanum -77.0722032 38.7804695 3686

Amblyomma americanum americanum A. americanum -77.0722032 38.7804695 3686

Amblyomma americanum americanum A. americanum -77.0722032 38.7804695 3686

Amblyomma americanum americanum A. americanum -77.0722032 38.7804695 3686

Amblyomma americanum americanum A. americanum -77.0722032 38.7804695 3686

Amblyomma americanum americanum A. americanum -77.0722032 38.7804695 3686

Amblyomma americanum americanum A. americanum -77.0722032 38.7804695 3686

Amblyomma americanum americanum A. americanum -77.0722032 38.7804695 3686

Amblyomma americanum americanum A. americanum -77.0722032 38.7804695 3686

Amblyomma americanum americanum A. americanum -77.0722032 38.7804695 3686

Amblyomma americanum americanum A. americanum -77.0722032 38.7804695 3686

Amblyomma americanum americanum A. americanum -77.0722032 38.7804695 3686

Amblyomma americanum americanum A. americanum -77.0722032 38.7804695 3686

Amblyomma americanum americanum A. americanum -77.0722032 38.7804695 3686

Amblyomma americanum americanum A. americanum -77.0722032 38.7804695 3686

Amblyomma americanum americanum A. americanum -77.0722032 38.7804695 3686

Amblyomma americanum americanum A. americanum -77.0722032 38.7804695 3686

Amblyomma americanum americanum A. americanum -77.0722032 38.7804695 3686

Amblyomma americanum americanum A. americanum -77.0722032 38.7804695 3686

Amblyomma americanum americanum A. americanum -77.0722032 38.7804695 3686

Amblyomma americanum americanum A. americanum -77.0722032 38.7804695 3686

Amblyomma americanum americanum A. americanum -77.0722032 38.7804695 3686

Amblyomma americanum americanum A. americanum -77.0722032 38.7804695 3686

Amblyomma americanum americanum A. americanum -77.0722032 38.7804695 3686

Amblyomma americanum americanum A. americanum -77.0722032 38.7804695 3686

Amblyomma americanum americanum A. americanum -77.0722032 38.7804695 3686

Amblyomma americanum americanum A. americanum -77.0722032 38.7804695 3686

Amblyomma americanum americanum A. americanum -77.0722032 38.7804695 3686

Amblyomma americanum americanum A. americanum -77.0722032 38.7804695 3686

Amblyomma americanum americanum A. americanum -77.0722032 38.7804695 3686

Amblyomma americanum americanum A. americanum -77.0722032 38.7804695 3686

Amblyomma americanum americanum A. americanum -77.0722032 38.7804695 3686

Amblyomma americanum americanum A. americanum -77.0722032 38.7804695 3686

Amblyomma americanum americanum A. americanum -77.0722032 38.7804695 3686

Amblyomma americanum americanum A. americanum -77.0722032 38.7804695 3686

Amblyomma americanum americanum A. americanum -77.0722032 38.7804695 3686

Amblyomma americanum americanum A. americanum -77.0722032 38.7804695 3686

Amblyomma americanum americanum A. americanum -77.0722032 38.7804695 3686

Amblyomma americanum americanum A. americanum -77.0722032 38.7804695 3686

Amblyomma americanum americanum A. americanum -77.0722032 38.7804695 3686

Amblyomma americanum americanum A. americanum -77.99555 38.67151 4500

Amblyomma americanum americanum A. americanum -78.73918 35.5071 2500

Amblyomma americanum americanum A. americanum -77.19637 38.83039 6000

Amblyomma americanum americanum A. americanum -77.19637 38.83039 6000

Amblyomma americanum americanum A. americanum -77.19637 38.83039 6000

Amblyomma americanum americanum A. americanum -77.19637 38.83039 6000

Amblyomma americanum americanum A. americanum -77.19637 38.83039 6000

Amblyomma americanum americanum A. americanum -76.4956594 38.9784451 5315

Amblyomma americanum americanum A. americanum -76.4956594 38.9784451 5315

Amblyomma americanum americanum A. americanum -76.4956594 38.9784451 5315

Amblyomma americanum americanum A. americanum -77.2750854 38.1097082 13000

Amblyomma americanum americanum A. americanum -77.2750854 38.1097082 13000

Amblyomma americanum americanum A. americanum -77.2750854 38.1097082 13000

Amblyomma americanum americanum A. americanum -77.2750854 38.1097082 13000

Amblyomma americanum americanum A. americanum -77.2750854 38.1097082 13000

Amblyomma americanum americanum A. americanum -77.2750854 38.1097082 13000

Amblyomma americanum americanum A. americanum -77.2750854 38.1097082 13000

Amblyomma americanum americanum A. americanum -77.2750854 38.1097082 13000

Amblyomma americanum americanum A. americanum -77.2750854 38.1097082 13000

Amblyomma americanum americanum A. americanum -77.2750854 38.1097082 13000

Amblyomma americanum americanum A. americanum -77.2750854 38.1097082 13000

Amblyomma americanum americanum A. americanum -77.2750854 38.1097082 13000

Amblyomma americanum americanum A. americanum -77.2750854 38.1097082 13000

Amblyomma americanum americanum A. americanum -77.2750854 38.1097082 13000

Amblyomma americanum americanum A. americanum -77.2750854 38.1097082 13000

Amblyomma americanum americanum A. americanum -77.2750854 38.1097082 13000

Amblyomma americanum americanum A. americanum -78.85029 35.73265 7500

Amblyomma americanum americanum A. americanum -78.85029 35.73265 7500

Amblyomma americanum americanum A. americanum -76.1647796 39.4442798 12000

Amblyomma americanum americanum A. americanum -76.1647796 39.4442798 12000

Amblyomma americanum americanum A. americanum -76.1647796 39.4442798 12000

Amblyomma americanum americanum A. americanum -76.1647796 39.4442798 12000

Amblyomma americanum americanum A. americanum -76.82549 35.02572 4000

Amblyomma americanum americanum A. americanum -86.8469429 34.9696042 5674

Amblyomma americanum americanum A. americanum -77.0895563 38.8866454 5230

Amblyomma americanum americanum A. americanum -77.0895563 38.8866454 5230

Amblyomma americanum americanum A. americanum -77.0895563 38.8866454 5230

Amblyomma americanum americanum A. americanum -77.0895563 38.8866454 5230

Amblyomma americanum americanum A. americanum -77.0895563 38.8866454 5230

Amblyomma americanum americanum A. americanum -77.0895563 38.8866454 5230

Amblyomma americanum americanum A. americanum -76.5027428 39.0312967 5140

Amblyomma americanum americanum A. americanum -77.4874954 39.0437183 5062

Amblyomma americanum americanum A. americanum -75.2038 38.09179 30000

Amblyomma americanum americanum A. americanum -84.3879852 33.7489948 20206

Amblyomma americanum americanum A. americanum -74.4292512 39.3713841 5269

Amblyomma americanum americanum A. americanum -85.4668999 37.8092308 5062

Amblyomma americanum americanum A. americanum -74.2247732 39.7531776 5210

Amblyomma americanum americanum A. americanum -80.027761 36.774436 5000

Amblyomma americanum americanum A. americanum -80.027761 36.774436 5000

Amblyomma americanum americanum A. americanum -76.3482933 39.5359402 5062

Amblyomma americanum americanum A. americanum -76.3482933 39.5359402 5062

Amblyomma americanum americanum A. americanum -76.2332878 39.4693099 5096

Amblyomma americanum americanum A. americanum -76.2332878 39.4693099 5096

Amblyomma americanum americanum A. americanum -76.2332878 39.4693099 5096

Amblyomma americanum americanum A. americanum -76.2332878 39.4693099 5096

Amblyomma americanum americanum A. americanum -76.9069922 39.0348339 5103

Amblyomma americanum americanum A. americanum -76.9069922 39.0348339 5103

Amblyomma americanum americanum A. americanum -76.9069922 39.0348339 5103

Amblyomma americanum americanum A. americanum -76.9069922 39.0348339 5103

Amblyomma americanum americanum A. americanum -75.05518 38.53956 2000

Amblyomma americanum americanum A. americanum -80.41394 37.22957 6000

Amblyomma americanum americanum A. americanum -77.9972267 37.0804272 5063

Amblyomma americanum americanum A. americanum -78.06721 34.03045 9000

Amblyomma americanum americanum A. americanum -78.06721 34.03045 9000

Amblyomma americanum americanum A. americanum -77.128315 40.1498127 5062

Amblyomma americanum americanum A. americanum -77.0184516 38.8393064 3500

Amblyomma americanum americanum A. americanum -77.34665 38.04958 2000

Amblyomma americanum americanum A. americanum -94.88358 39.05973 6000

Amblyomma americanum americanum A. americanum -86.1694145 37.9899147 5706

Amblyomma americanum americanum A. americanum -76.8477516 38.6969317 5079

Amblyomma americanum americanum A. americanum -71.2647679 41.6772631 5113

Amblyomma americanum americanum A. americanum -74.5829353 39.9726162 5062

Amblyomma americanum americanum A. americanum -74.5829353 39.9726162 5062

Amblyomma americanum americanum A. americanum -74.5829353 39.9726162 5062

Amblyomma americanum americanum A. americanum -74.5829353 39.9726162 5062

Amblyomma americanum americanum A. americanum -74.5829353 39.9726162 5062

Amblyomma americanum americanum A. americanum -74.5829353 39.9726162 5062

Amblyomma americanum americanum A. americanum -77.07303 38.62706 4000

Amblyomma americanum americanum A. americanum -78.55556 37.55015 4000

Amblyomma americanum americanum A. americanum -78.55556 37.55015 4000

Amblyomma americanum americanum A. americanum -76.34772 37.88319 3500

Amblyomma americanum americanum A. americanum -77.2716522 38.7934494 5062

Amblyomma americanum americanum A. americanum -77.2716522 38.7934494 5062

Amblyomma americanum americanum A. americanum -77.2716522 38.7934494 5062

Amblyomma americanum americanum A. americanum -77.2716522 38.7934494 5062

Amblyomma americanum americanum A. americanum -77.2716522 38.7934494 5062

Amblyomma americanum americanum A. americanum -77.2716522 38.7934494 5062

Amblyomma americanum americanum A. americanum -77.2716522 38.7934494 5062

Amblyomma americanum americanum A. americanum -77.2716522 38.7934494 5062

Amblyomma americanum americanum A. americanum -77.2716522 38.7934494 5062

Amblyomma americanum americanum A. americanum -77.2716522 38.7934494 5062

Amblyomma americanum americanum A. americanum -77.2716522 38.7934494 5062

Amblyomma americanum americanum A. americanum -85.46108 35.47507 6000

Amblyomma americanum americanum A. americanum -76.51412 38.52512 2000

Amblyomma americanum americanum A. americanum -75.5418663 39.1134472 5062

Amblyomma americanum americanum A. americanum -79.2553062 35.3268223 5063

Amblyomma americanum americanum A. americanum -79.2553062 35.3268223 5063

Amblyomma americanum americanum A. americanum -79.256467 35.3251075 5202

Amblyomma americanum americanum A. americanum -74.9088603 38.9531777 5585

Amblyomma americanum americanum A. americanum -77.1888695 40.201479 5062

Amblyomma americanum americanum A. americanum -77.1888695 40.201479 5062

Amblyomma americanum americanum A. americanum -79.4169731 35.3459873 5063

Amblyomma americanum americanum A. americanum -85.95167 36.25222 2587

Amblyomma americanum americanum A. americanum -79.4169731 35.3459873 5063

Amblyomma americanum americanum A. americanum -78.78139 35.79139 12809

Amblyomma americanum americanum A. americanum -78.78139 35.79139 12809

Amblyomma americanum americanum A. americanum -85.95694 37.13667 3481

Amblyomma americanum americanum A. americanum -77.4288788 38.8403931 5062

Amblyomma americanum americanum A. americanum -77.4288788 38.8403931 5062

Amblyomma americanum americanum A. americanum -77.4288788 38.8403931 5062

Amblyomma americanum americanum A. americanum -77.4288788 38.8403931 5062

Amblyomma americanum americanum A. americanum -77.4288788 38.8403931 5062

Amblyomma americanum americanum A. americanum -77.4288788 38.8403931 5062

Amblyomma americanum americanum A. americanum -77.4288788 38.8403931 5062

Amblyomma americanum americanum A. americanum -77.4288788 38.8403931 5062

Amblyomma americanum americanum A. americanum -77.4288788 38.8403931 5062

Amblyomma americanum americanum A. americanum -77.4288788 38.8403931 5062

Amblyomma americanum americanum A. americanum -77.4288788 38.8403931 5062

Amblyomma americanum americanum A. americanum -77.4288788 38.8403931 5062

Amblyomma americanum americanum A. americanum -77.4310989 38.8942795 5062

Amblyomma americanum americanum A. americanum -79.0582065 35.9132004 5252

Amblyomma americanum americanum A. americanum -97.02222 38.97222 1677

Amblyomma americanum americanum A. americanum -79.93111 32.77639 23749

Amblyomma americanum americanum A. americanum -78.4766808 38.0293045 5062

Amblyomma americanum americanum A. americanum -78.4766808 38.0293045 5062

Amblyomma americanum americanum A. americanum -76.1302032 44.0670119 5063

Amblyomma americanum americanum A. americanum -76.2802426 36.7081798 25751

Amblyomma americanum americanum A. americanum -76.2802426 36.7081798 25751

Amblyomma americanum americanum A. americanum -76.535 38.68611 2640

Amblyomma americanum americanum A. americanum -75.8210411 39.5309467 5768

Amblyomma americanum americanum A. americanum -77.4416542 37.3568172 5062

Amblyomma americanum americanum A. americanum -77.4416542 37.3568172 5062

Amblyomma americanum americanum A. americanum -77.4416542 37.3568172 5062

Amblyomma americanum americanum A. americanum -77.4416542 37.3568172 5062

Amblyomma americanum americanum A. americanum -77.4416542 37.3568172 5062

Amblyomma americanum americanum A. americanum -77.4416542 37.3568172 5062

Amblyomma americanum americanum A. americanum -77.4416542 37.3568172 5062

Amblyomma americanum americanum A. americanum -77.4416542 37.3568172 5062

Amblyomma americanum americanum A. americanum -77.4416542 37.3568172 5062

Amblyomma americanum americanum A. americanum -77.4416542 37.3568172 5062

Amblyomma americanum americanum A. americanum -77.4416542 37.3568172 5062

Amblyomma americanum americanum A. americanum -77.20278 36.88889 3036

Amblyomma americanum americanum A. americanum -87.3594513 36.5297699 5063

Amblyomma americanum americanum A. americanum -87.3594513 36.5297699 5063

Amblyomma americanum americanum A. americanum -87.3594513 36.5297699 5063

Amblyomma americanum americanum A. americanum -87.3594513 36.5297699 5063

Amblyomma americanum americanum A. americanum -87.3594513 36.5297699 5063

Amblyomma americanum americanum A. americanum -87.3594513 36.5297699 5063

Amblyomma americanum americanum A. americanum -87.3594513 36.5297699 5063

Amblyomma americanum americanum A. americanum -87.3594513 36.5297699 5063

Amblyomma americanum americanum A. americanum -87.3594513 36.5297699 5063

Amblyomma americanum americanum A. americanum -87.3594513 36.5297699 5063

Amblyomma americanum americanum A. americanum -87.3594513 36.5297699 5063

Amblyomma americanum americanum A. americanum -87.3594513 36.5297699 5063

Amblyomma americanum americanum A. americanum -87.3594513 36.5297699 5063

Amblyomma americanum americanum A. americanum -87.3594513 36.5297699 5063

Amblyomma americanum americanum A. americanum -87.3594513 36.5297699 5063

Amblyomma americanum americanum A. americanum -87.3594513 36.5297699 5063

Amblyomma americanum americanum A. americanum -87.3594513 36.5297699 5063

Amblyomma americanum americanum A. americanum -87.3594513 36.5297699 5063

Amblyomma americanum americanum A. americanum -87.3594513 36.5297699 5063

Amblyomma americanum americanum A. americanum -87.3594513 36.5297699 5063

Amblyomma americanum americanum A. americanum -87.3594513 36.5297699 5063

Amblyomma americanum americanum A. americanum -87.3594513 36.5297699 5063

Amblyomma americanum americanum A. americanum -87.3594513 36.5297699 5063

Amblyomma americanum americanum A. americanum -87.3594513 36.5297699 5063

Amblyomma americanum americanum A. americanum -87.3594513 36.5297699 5063

Amblyomma americanum americanum A. americanum -87.3594513 36.5297699 5063

Amblyomma americanum americanum A. americanum -87.3594513 36.5297699 5063

Amblyomma americanum americanum A. americanum -87.3594513 36.5297699 5063

Amblyomma americanum americanum A. americanum -87.3594513 36.5297699 5063

Amblyomma americanum americanum A. americanum -87.3594513 36.5297699 5063

Amblyomma americanum americanum A. americanum -87.3594513 36.5297699 5063

Amblyomma americanum americanum A. americanum -87.3594513 36.5297699 5063

Amblyomma americanum americanum A. americanum -87.3594513 36.5297699 5063

Amblyomma americanum americanum A. americanum -87.3594513 36.5297699 5063

Amblyomma americanum americanum A. americanum -87.3594513 36.5297699 5063

Amblyomma americanum americanum A. americanum -87.3594513 36.5297699 5063

Amblyomma americanum americanum A. americanum -85.7595492 38.2698995 5102

Amblyomma americanum americanum A. americanum -87.3594513 36.5297699 5063

Amblyomma americanum americanum A. americanum -87.3594513 36.5297699 5063

Amblyomma americanum americanum A. americanum -87.3594513 36.5297699 5063

Amblyomma americanum americanum A. americanum -87.3594513 36.5297699 5063

Amblyomma americanum americanum A. americanum -87.3594513 36.5297699 5063

Amblyomma americanum americanum A. americanum -87.3594513 36.5297699 5063

Amblyomma americanum americanum A. americanum -87.3594513 36.5297699 5063

Amblyomma americanum americanum A. americanum -87.3594513 36.5297699 5063

Amblyomma americanum americanum A. americanum -87.3594513 36.5297699 5063

Amblyomma americanum americanum A. americanum -87.3594513 36.5297699 5063

Amblyomma americanum americanum A. americanum -87.3594513 36.5297699 5063

Amblyomma americanum americanum A. americanum -87.3594513 36.5297699 5063

Amblyomma americanum americanum A. americanum -87.3594513 36.5297699 5063

Amblyomma americanum americanum A. americanum -87.3594513 36.5297699 5063

Amblyomma americanum americanum A. americanum -87.3594513 36.5297699 5063

Amblyomma americanum americanum A. americanum -87.3594513 36.5297699 5063

Amblyomma americanum americanum A. americanum -87.3594513 36.5297699 5063

Amblyomma americanum americanum A. americanum -87.3594513 36.5297699 5063

Amblyomma americanum americanum A. americanum -87.3594513 36.5297699 5063

Amblyomma americanum americanum A. americanum -87.3594513 36.5297699 5063

Amblyomma americanum americanum A. americanum -87.3594513 36.5297699 5063

Amblyomma americanum americanum A. americanum -76.9430389 39.2064934 5062

Amblyomma americanum americanum A. americanum -87.3594513 36.5297699 5063

Amblyomma americanum americanum A. americanum -87.3594513 36.5297699 5063

Amblyomma americanum americanum A. americanum -87.3594513 36.5297699 5063

Amblyomma americanum americanum A. americanum -87.3594513 36.5297699 5063

Amblyomma americanum americanum A. americanum -87.3594513 36.5297699 5063

Amblyomma americanum americanum A. americanum -87.3594513 36.5297699 5063

Amblyomma americanum americanum A. americanum -87.3594513 36.5297699 5063

Amblyomma americanum americanum A. americanum -87.3594513 36.5297699 5063

Amblyomma americanum americanum A. americanum -87.3594513 36.5297699 5063

Amblyomma americanum americanum A. americanum -87.3594513 36.5297699 5063

Amblyomma americanum americanum A. americanum -78.45667 35.65056 2767

Amblyomma americanum americanum A. americanum -77.3840356 38.7803305 5249

Amblyomma americanum americanum A. americanum -77.3840356 38.7803305 5249

Amblyomma americanum americanum A. americanum -77.3840356 38.7803305 5249

Amblyomma americanum americanum A. americanum -87.99528 35.38694 4056

Amblyomma americanum americanum A. americanum -77.3840356 38.7803305 5249

Amblyomma americanum americanum A. americanum -77.3840356 38.7803305 5249

Amblyomma americanum americanum A. americanum -76.898304 38.7651157 5062

Amblyomma americanum americanum A. americanum -74.1723671 40.2876129 5062

Amblyomma americanum americanum A. americanum -81.033777 34.0008472 5147

Amblyomma americanum americanum A. americanum -81.033777 34.0008472 5147

Amblyomma americanum americanum A. americanum -81.033777 34.0008472 5147

Amblyomma americanum americanum A. americanum -76.8394241 39.2403851 5062

Amblyomma americanum americanum A. americanum -81.033777 34.0008472 5147

Amblyomma americanum americanum A. americanum -81.033777 34.0008472 5147

Amblyomma americanum americanum A. americanum -75.8424916 36.3817404 5447

Amblyomma americanum americanum A. americanum -75.8424916 36.3817404 5447

Amblyomma americanum americanum A. americanum -78.12361 37.17306 3086

Amblyomma americanum americanum A. americanum -76.6857168 39.0017757 5203

Amblyomma americanum americanum A. americanum -78.70083 38.06944 3724

Amblyomma americanum americanum A. americanum -77.9966583 38.4731827 5062

Amblyomma americanum americanum A. americanum -87.6426815 36.4033861 5990

Amblyomma americanum americanum A. americanum -85.76361 32.83111 6636

Amblyomma americanum americanum A. americanum -77.3110924 38.637064 5062

Amblyomma americanum americanum A. americanum -77.3110924 38.637064 5062

Amblyomma americanum americanum A. americanum -77.3110924 38.637064 5062

Amblyomma americanum americanum A. americanum -79.87639 34.29972 3616

Amblyomma americanum americanum A. americanum -75.58917 39.57778 1930

Amblyomma americanum americanum A. americanum -74.6167373 40.0086834 6500

Amblyomma americanum americanum A. americanum -74.6167373 40.0086834 6500

Amblyomma americanum americanum A. americanum -74.6167373 40.0086834 6500

Amblyomma americanum americanum A. americanum -74.6167373 40.0086834 6500

Amblyomma americanum americanum A. americanum -74.6167373 40.0086834 6500

Amblyomma americanum americanum A. americanum -86.23 36.29833 3036

Amblyomma americanum americanum A. americanum -92.0937767 37.982841 5697

Amblyomma americanum americanum A. americanum -92.09361 37.99167 1574

Amblyomma americanum americanum A. americanum -87.8383598 36.4878311 5063

Amblyomma americanum americanum A. americanum -75.5243645 39.158165 5062

Amblyomma americanum americanum A. americanum -75.5243645 39.158165 5062

Amblyomma americanum americanum A. americanum -75.5243645 39.158165 5062

Amblyomma americanum americanum A. americanum -75.5243645 39.158165 5062

Amblyomma americanum americanum A. americanum -75.5243645 39.158165 5062

Amblyomma americanum americanum A. americanum -75.5243645 39.158165 5062

Amblyomma americanum americanum A. americanum -87.8383598 36.4878311 5063

Amblyomma americanum americanum A. americanum -87.8383598 36.4878311 5063

Amblyomma americanum americanum A. americanum -76.26694 39.65083 3036

Amblyomma americanum americanum A. americanum -77.3280373 38.5676212 5062

Amblyomma americanum americanum A. americanum -77.3280373 38.5676212 5062

Amblyomma americanum americanum A. americanum -77.3280373 38.5676212 5062

Amblyomma americanum americanum A. americanum -77.3280373 38.5676212 5062

Amblyomma americanum americanum A. americanum -77.3280373 38.5676212 5062

Amblyomma americanum americanum A. americanum -78.8572921 36.0512598 25293

Amblyomma americanum americanum A. americanum -95.11556 39.345 720

Amblyomma americanum americanum A. americanum -95.11556 39.345 720

Amblyomma americanum americanum A. americanum -76.2959883 39.4187183 5189

Amblyomma americanum americanum A. americanum -76.2959883 39.4187183 5189

Amblyomma americanum americanum A. americanum -76.2959883 39.4187183 5189

Amblyomma americanum americanum A. americanum -76.2959883 39.4187183 5189

Amblyomma americanum americanum A. americanum -76.2959883 39.4187183 5189

Amblyomma americanum americanum A. americanum -86.17944 37.92917 481

Amblyomma americanum americanum A. americanum -85.859127 37.6939526 5062

Amblyomma americanum americanum A. americanum -85.859127 37.6939526 5062

Amblyomma americanum americanum A. americanum -85.859127 37.6939526 5062

Amblyomma americanum americanum A. americanum -85.859127 37.6939526 5062

Amblyomma americanum americanum A. americanum -85.859127 37.6939526 5062

Amblyomma americanum americanum A. americanum -85.859127 37.6939526 5062

Amblyomma americanum americanum A. americanum -78.6236305 38.4079037 5062

Amblyomma americanum americanum A. americanum -78.6236305 38.4079037 5062

Amblyomma americanum americanum A. americanum -85.859127 37.6939526 5062

Amblyomma americanum americanum A. americanum -75.82333 37.53167 1556

Amblyomma americanum americanum A. americanum -84.01944 39.82083 6868

Amblyomma americanum americanum A. americanum -77.3063698 38.8462238 5062

Amblyomma americanum americanum A. americanum -77.3063698 38.8462238 5062

Amblyomma americanum americanum A. americanum -77.3063698 38.8462238 5062

Amblyomma americanum americanum A. americanum -77.3063698 38.8462238 5062

Amblyomma americanum americanum A. americanum -77.3063698 38.8462238 5062

Amblyomma americanum americanum A. americanum -77.3063698 38.8462238 5062

Amblyomma americanum americanum A. americanum -77.3063698 38.8462238 5062

Amblyomma americanum americanum A. americanum -77.3063698 38.8462238 5062

Amblyomma americanum americanum A. americanum -77.3063698 38.8462238 5062

Amblyomma americanum americanum A. americanum -77.3063698 38.8462238 5062

Amblyomma americanum americanum A. americanum -77.3063698 38.8462238 5062

Amblyomma americanum americanum A. americanum -77.3063698 38.8462238 5062

Amblyomma americanum americanum A. americanum -77.3063698 38.8462238 5062

Amblyomma americanum americanum A. americanum -77.3063698 38.8462238 5062

Amblyomma americanum americanum A. americanum -77.3063698 38.8462238 5062

Amblyomma americanum americanum A. americanum -77.3063698 38.8462238 5062

Amblyomma americanum americanum A. americanum -77.3063698 38.8462238 5062

Amblyomma americanum americanum A. americanum -77.3063698 38.8462238 5062

Amblyomma americanum americanum A. americanum -77.3063698 38.8462238 5062

Amblyomma americanum americanum A. americanum -77.3063698 38.8462238 5062

Amblyomma americanum americanum A. americanum -77.3063698 38.8462238 5062

Amblyomma americanum americanum A. americanum -77.3063698 38.8462238 5062

Amblyomma americanum americanum A. americanum -77.3063698 38.8462238 5062

Amblyomma americanum americanum A. americanum -77.3063698 38.8462238 5062

Amblyomma americanum americanum A. americanum -77.3063698 38.8462238 5062

Amblyomma americanum americanum A. americanum -77.3063698 38.8462238 5062

Amblyomma americanum americanum A. americanum -77.3063698 38.8462238 5062

Amblyomma americanum americanum A. americanum -77.3063698 38.8462238 5062

Amblyomma americanum americanum A. americanum -77.3063698 38.8462238 5062

Amblyomma americanum americanum A. americanum -77.3063698 38.8462238 5062

Amblyomma americanum americanum A. americanum -77.3063698 38.8462238 5062

Amblyomma americanum americanum A. americanum -77.3063698 38.8462238 5062

Amblyomma americanum americanum A. americanum -77.3063698 38.8462238 5062

Amblyomma americanum americanum A. americanum -77.3063698 38.8462238 5062

Amblyomma americanum americanum A. americanum -77.3063698 38.8462238 5062

Amblyomma americanum americanum A. americanum -77.3063698 38.8462238 5062

Amblyomma americanum americanum A. americanum -78.5813942 35.9418163 3054

Amblyomma americanum americanum A. americanum -78.5813942 35.9418163 3054

Amblyomma americanum americanum A. americanum -77.3063698 38.8462238 5062

Amblyomma americanum americanum A. americanum -78.8881989 35.0527 6468

Amblyomma americanum americanum A. americanum -78.8881989 35.0527 6468

Amblyomma americanum americanum A. americanum -78.8881989 35.0527 6468

Amblyomma americanum americanum A. americanum -78.8881989 35.0527 6468

Amblyomma americanum americanum A. americanum -78.8881989 35.0527 6468

Amblyomma americanum americanum A. americanum -75.5779839 39.0084496 5062

Amblyomma americanum americanum A. americanum -75.5779839 39.0084496 5062

Amblyomma americanum americanum A. americanum -75.5779839 39.0084496 5062

Amblyomma americanum americanum A. americanum -75.5779839 39.0084496 5062

Amblyomma americanum americanum A. americanum -75.76361 44.02222 3036

Amblyomma americanum americanum A. americanum -86.06556 37.83639 3036

Amblyomma americanum americanum A. americanum -86.06556 37.83639 3036

Amblyomma americanum americanum A. americanum -79.29 37.36361 7122

Amblyomma americanum americanum A. americanum -76.3877411 39.5851097 5062

Amblyomma americanum americanum A. americanum -77.2750854 38.1097082 13000

Amblyomma americanum americanum A. americanum -77.2750854 38.1097082 13000

Amblyomma americanum americanum A. americanum -77.2750854 38.1097082 13000

Amblyomma americanum americanum A. americanum -77.2750854 38.1097082 13000

Amblyomma americanum americanum A. americanum -77.2750854 38.1097082 13000

Amblyomma americanum americanum A. americanum -77.2750854 38.1097082 13000

Amblyomma americanum americanum A. americanum -77.2750854 38.1097082 13000

Amblyomma americanum americanum A. americanum -77.2750854 38.1097082 13000

Amblyomma americanum americanum A. americanum -77.2750854 38.1097082 13000

Amblyomma americanum americanum A. americanum -77.2750854 38.1097082 13000

Amblyomma americanum americanum A. americanum -77.2750854 38.1097082 13000

Amblyomma americanum americanum A. americanum -77.2750854 38.1097082 13000

Amblyomma americanum americanum A. americanum -77.2750854 38.1097082 13000

Amblyomma americanum americanum A. americanum -77.2750854 38.1097082 13000

Amblyomma americanum americanum A. americanum -77.2750854 38.1097082 13000

Amblyomma americanum americanum A. americanum -77.2750854 38.1097082 13000

Amblyomma americanum americanum A. americanum -77.2750854 38.1097082 13000

Amblyomma americanum americanum A. americanum -77.2750854 38.1097082 13000

Amblyomma americanum americanum A. americanum -77.2750854 38.1097082 13000

Amblyomma americanum americanum A. americanum -77.2750854 38.1097082 13000

Amblyomma americanum americanum A. americanum -77.2750854 38.1097082 13000

Amblyomma americanum americanum A. americanum -77.2750854 38.1097082 13000

Amblyomma americanum americanum A. americanum -77.2750854 38.1097082 13000

Amblyomma americanum americanum A. americanum -77.2750854 38.1097082 13000

Amblyomma americanum americanum A. americanum -77.2750854 38.1097082 13000

Amblyomma americanum americanum A. americanum -77.4312973 39.4391096 3000

Amblyomma americanum americanum A. americanum -74.6167373 40.0086834 6500

Amblyomma americanum americanum A. americanum -77.05833 38.73278 4231

Amblyomma americanum americanum A. americanum -85.9658626 37.8897647 5709

Amblyomma americanum americanum A. americanum -85.9658626 37.8897647 5709

Amblyomma americanum americanum A. americanum -85.9658626 37.8897647 5709

Amblyomma americanum americanum A. americanum -85.9658626 37.8897647 5709

Amblyomma americanum americanum A. americanum -76.743264 39.108887 3000

Amblyomma americanum americanum A. americanum -76.3031387 37.009681 3000

Amblyomma americanum americanum A. americanum -76.3031387 37.009681 3000

Amblyomma americanum americanum A. americanum -77.4105415 39.4142685 5062

Amblyomma americanum americanum A. americanum -77.4105415 39.4142685 5062

Amblyomma americanum americanum A. americanum -75.4657555 39.0090008 5062

Amblyomma americanum americanum A. americanum -76.4282951 40.4436895 5063

Amblyomma americanum americanum A. americanum -77.4655535 38.2793694 5468

Amblyomma americanum americanum A. americanum -77.4655535 38.2793694 5468

Amblyomma americanum americanum A. americanum -74.2737541 40.2601109 5062

Amblyomma americanum americanum A. americanum -78.19472 38.91806 4810

Amblyomma americanum americanum A. americanum -78.19472 38.91806 4810

Amblyomma americanum americanum A. americanum -77.2750854 38.1097082 13000

Amblyomma americanum americanum A. americanum -77.2750854 38.1097082 13000

Amblyomma americanum americanum A. americanum -77.2750854 38.1097082 13000

Amblyomma americanum americanum A. americanum -77.2750854 38.1097082 13000

Amblyomma americanum americanum A. americanum -77.1602733 38.7121998 6272

Amblyomma americanum americanum A. americanum -77.1602733 38.7121998 6272

Amblyomma americanum americanum A. americanum -77.1602733 38.7121998 6272

Amblyomma americanum americanum A. americanum -79.1970062 35.1109218 17000

Amblyomma americanum americanum A. americanum -74.6167373 40.0086834 6500

Amblyomma americanum americanum A. americanum -74.6167373 40.0086834 6500

Amblyomma americanum americanum A. americanum -74.6167373 40.0086834 6500

Amblyomma americanum americanum A. americanum -74.6167373 40.0086834 6500

Amblyomma americanum americanum A. americanum -74.6167373 40.0086834 6500

Amblyomma americanum americanum A. americanum -74.6167373 40.0086834 6500

Amblyomma americanum americanum A. americanum -74.6167373 40.0086834 6500

Amblyomma americanum americanum A. americanum -74.6167373 40.0086834 6500

Amblyomma americanum americanum A. americanum -74.6167373 40.0086834 6500

Amblyomma americanum americanum A. americanum -74.6167373 40.0086834 6500

Amblyomma americanum americanum A. americanum -74.6167373 40.0086834 6500

Amblyomma americanum americanum A. americanum -74.6167373 40.0086834 6500

Amblyomma americanum americanum A. americanum -74.6167373 40.0086834 6500

Amblyomma americanum americanum A. americanum -74.6167373 40.0086834 6500

Amblyomma americanum americanum A. americanum -74.6167373 40.0086834 6500

Amblyomma americanum americanum A. americanum -74.6167373 40.0086834 6500

Amblyomma americanum americanum A. americanum -74.6167373 40.0086834 6500

Amblyomma americanum americanum A. americanum -74.6167373 40.0086834 6500

Amblyomma americanum americanum A. americanum -74.6167373 40.0086834 6500

Amblyomma americanum americanum A. americanum -74.6167373 40.0086834 6500

Amblyomma americanum americanum A. americanum -74.6167373 40.0086834 6500

Amblyomma americanum americanum A. americanum -74.6167373 40.0086834 6500

Amblyomma americanum americanum A. americanum -76.5877532 37.1534759 9500

Amblyomma americanum americanum A. americanum -85.8571243 37.9095336 21000

Amblyomma americanum americanum A. americanum -85.8571243 37.9095336 21000

Amblyomma americanum americanum A. americanum -85.8571243 37.9095336 21000

Amblyomma americanum americanum A. americanum -77.915039 37.0420244 10500

Amblyomma americanum americanum A. americanum -77.915039 37.0420244 10500

Amblyomma americanum americanum A. americanum -77.915039 37.0420244 10500

Amblyomma americanum americanum A. americanum -77.915039 37.0420244 10500

Amblyomma americanum americanum A. americanum -77.2750854 38.1097082 13000

Amblyomma americanum americanum A. americanum -77.915039 37.0420244 10500

Amblyomma americanum americanum A. americanum -77.915039 37.0420244 10500

Amblyomma americanum americanum A. americanum -77.915039 37.0420244 10500

Amblyomma americanum americanum A. americanum -77.915039 37.0420244 10500

Amblyomma americanum americanum A. americanum -77.915039 37.0420244 10500

Amblyomma americanum americanum A. americanum -77.915039 37.0420244 10500

Amblyomma americanum americanum A. americanum -77.915039 37.0420244 10500

Amblyomma americanum americanum A. americanum -77.915039 37.0420244 10500

Amblyomma americanum americanum A. americanum -77.915039 37.0420244 10500

Amblyomma americanum americanum A. americanum -77.915039 37.0420244 10500

Amblyomma americanum americanum A. americanum -77.6138802 38.7956715 5062

Amblyomma americanum americanum A. americanum -77.6138802 38.7956715 5062

Amblyomma americanum americanum A. americanum -77.6138802 38.7956715 5062

Amblyomma americanum americanum A. americanum -77.2013702 39.1434383 5062

Amblyomma americanum americanum A. americanum -76.6652374 39.0670547 5062

Amblyomma americanum americanum A. americanum -76.6652374 39.0670547 5062

Amblyomma americanum americanum A. americanum -94.92694 38.81083 3777

Amblyomma americanum americanum A. americanum -77.38556 36.75944 3036

Amblyomma americanum americanum A. americanum -76.624691 39.1626053 5062

Amblyomma americanum americanum A. americanum -76.624691 39.1626053 5062

Amblyomma americanum americanum A. americanum -76.5255089 37.4137516 5062

Amblyomma americanum americanum A. americanum -76.5255089 37.4137516 5062

Amblyomma americanum americanum A. americanum -76.5255089 37.4137516 5062

Amblyomma americanum americanum A. americanum -76.5255089 37.4137516 5062

Amblyomma americanum americanum A. americanum -76.5255089 37.4137516 5062

Amblyomma americanum americanum A. americanum -76.5255089 37.4137516 5062

Amblyomma americanum americanum A. americanum -76.8755341 39.0045547 5062

Amblyomma americanum americanum A. americanum -76.8755341 39.0045547 5062

Amblyomma americanum americanum A. americanum -76.8755341 39.0045547 5062

Amblyomma americanum americanum A. americanum -77.3663521 35.6126633 5063

Amblyomma americanum americanum A. americanum -77.72028 39.64167 5798

Amblyomma americanum americanum A. americanum -76.85028 39.60472 4074

Amblyomma americanum americanum A. americanum -76.3456386 37.0353572 5490

Amblyomma americanum americanum A. americanum -76.3456386 37.0353572 5490

Amblyomma americanum americanum A. americanum -76.3456386 37.0353572 5490

Amblyomma americanum americanum A. americanum -76.7177819 39.185083 5299

Amblyomma americanum americanum A. americanum -75.5777054 38.9237232 5062

Amblyomma americanum americanum A. americanum -75.5777054 38.9237232 5062

Amblyomma americanum americanum A. americanum -94.15417 37.95611 478

Amblyomma americanum americanum A. americanum -76.1131431 39.548738 4688

Amblyomma americanum americanum A. americanum -76.1131431 39.548738 4688

Amblyomma americanum americanum A. americanum -76.1131431 39.548738 4688

Amblyomma americanum americanum A. americanum -76.1131431 39.548738 4688

Amblyomma americanum americanum A. americanum -76.1131431 39.548738 4688

Amblyomma americanum americanum A. americanum -76.1131431 39.548738 4688

Amblyomma americanum americanum A. americanum -76.1131431 39.548738 4688

Amblyomma americanum americanum A. americanum -77.6363792 38.8120613 5062

Amblyomma americanum americanum A. americanum -77.386097 38.969553 5062

Amblyomma americanum americanum A. americanum -77.386097 38.969553 5062

Amblyomma americanum americanum A. americanum -77.386097 38.969553 5062

Amblyomma americanum americanum A. americanum -77.386097 38.969553 5062

Amblyomma americanum americanum A. americanum -76.605 36.34694 3036

Amblyomma americanum americanum A. americanum -78.833622 35.6512661 5063

Amblyomma americanum americanum A. americanum -77.2851026 37.2737314 6750

Amblyomma americanum americanum A. americanum -87.4886208 36.8656044 5063

Amblyomma americanum americanum A. americanum -87.4886208 36.8656044 5063

Amblyomma americanum americanum A. americanum -87.4886208 36.8656044 5063

Amblyomma americanum americanum A. americanum -87.4886208 36.8656044 5063

Amblyomma americanum americanum A. americanum -87.4886208 36.8656044 5063

Amblyomma americanum americanum A. americanum -74.208267 40.164578 5000

Amblyomma americanum americanum A. americanum -76.61333 38.61583 4237

Amblyomma americanum americanum A. americanum -86.5861053 34.7303677 5063

Amblyomma americanum americanum A. americanum -86.5861053 34.7303677 5063

Amblyomma americanum americanum A. americanum -86.5861053 34.7303677 5063

Amblyomma americanum americanum A. americanum -86.5861053 34.7303677 5063

Amblyomma americanum americanum A. americanum -86.5861053 34.7303677 5063

Amblyomma americanum americanum A. americanum -86.5861053 34.7303677 5063

Amblyomma americanum americanum A. americanum -86.5861053 34.7303677 5063

Amblyomma americanum americanum A. americanum -78.71833 39.82306 1390

Amblyomma americanum americanum A. americanum -87.693611 36.500833 5000

Amblyomma americanum americanum A. americanum -74.35618 40.093077 5000

Amblyomma americanum americanum A. americanum -77.4302444 34.754055 5063

Amblyomma americanum americanum A. americanum -71.4240877 41.5028614 2960

Amblyomma americanum americanum A. americanum -76.77556 39.14917 4087

Amblyomma americanum americanum A. americanum -76.3476329 39.4358542 5496

Amblyomma americanum americanum A. americanum -76.3476329 39.4358542 5496

Amblyomma americanum americanum A. americanum -76.3476329 39.4358542 5496

Amblyomma americanum americanum A. americanum -76.3476329 39.4358542 5496

Amblyomma americanum americanum A. americanum -94.7209166 39.2555592 14620

Amblyomma americanum americanum A. americanum -94.7209166 39.2555592 14620

Amblyomma americanum americanum A. americanum -78.12528 36.96194 3703

Amblyomma americanum americanum A. americanum -77.3003651 38.6802599 5496

Amblyomma americanum americanum A. americanum -77.3003651 38.6802599 5496

Amblyomma americanum americanum A. americanum -74.3112602 40.0145645 5062

Amblyomma americanum americanum A. americanum -74.3112602 40.0145645 5062

Amblyomma americanum americanum A. americanum -94.9002419 39.2486134 5062

Amblyomma americanum americanum A. americanum -94.9002419 39.2486134 5062

Amblyomma americanum americanum A. americanum -94.9002419 39.2486134 5062

Amblyomma americanum americanum A. americanum -76.83056 38.8975 3036

Amblyomma americanum americanum A. americanum -76.8600245 39.092708 5222

Amblyomma americanum americanum A. americanum -76.8600245 39.092708 5222

Amblyomma americanum americanum A. americanum -76.8600245 39.092708 5222

Amblyomma americanum americanum A. americanum -76.8600245 39.092708 5222

Amblyomma americanum americanum A. americanum -76.8600245 39.092708 5222

Amblyomma americanum americanum A. americanum -76.8600245 39.092708 5222

Amblyomma americanum americanum A. americanum -76.8600245 39.092708 5222

Amblyomma americanum americanum A. americanum -76.8600245 39.092708 5222

Amblyomma americanum americanum A. americanum -77.84722 36.7575 1335

Amblyomma americanum americanum A. americanum -94.9287936 39.3111076 5480

Amblyomma americanum americanum A. americanum -94.9287936 39.3111076 5480

Amblyomma americanum americanum A. americanum -94.9287936 39.3111076 5480

Amblyomma americanum americanum A. americanum -94.9287936 39.3111076 5480

Amblyomma americanum americanum A. americanum -94.9287936 39.3111076 5480

Amblyomma americanum americanum A. americanum -94.9287936 39.3111076 5480

Amblyomma americanum americanum A. americanum -94.9287936 39.3111076 5480

Amblyomma americanum americanum A. americanum -92.663784 37.6805973 5062

Amblyomma americanum americanum A. americanum -92.663784 37.6805973 5062

Amblyomma americanum americanum A. americanum -77.5636024 39.1156616 5062

Amblyomma americanum americanum A. americanum -78.045 34.25611 3036

Amblyomma americanum americanum A. americanum -75.13972 38.77444 4891

Amblyomma americanum americanum A. americanum -75.13972 38.77444 4891

Amblyomma americanum americanum A. americanum -75.13972 38.77444 4891

Amblyomma americanum americanum A. americanum -76.4538383 38.2657419 5170

Amblyomma americanum americanum A. americanum -76.4538383 38.2657419 5170

Amblyomma americanum americanum A. americanum -76.4538383 38.2657419 5170

Amblyomma americanum americanum A. americanum -76.4538383 38.2657419 5170

Amblyomma americanum americanum A. americanum -78.8158531 35.3993301 5063

Amblyomma americanum americanum A. americanum -75.42333 38.86972 3036

Amblyomma americanum americanum A. americanum -77.08833 39.74444 1638

Amblyomma americanum americanum A. americanum -71.5109804 42.5375919 5140

Amblyomma americanum americanum A. americanum -74.0085853 40.3042774 5761

Amblyomma americanum americanum A. americanum -72.9934926 40.9669994 103401

Amblyomma americanum americanum A. americanum -77.2277641 38.7042868 5063

Amblyomma americanum americanum A. americanum -77.2277641 38.7042868 5063

Amblyomma americanum americanum A. americanum -77.2277641 38.7042868 5063

Amblyomma americanum americanum A. americanum -77.2277641 38.7042868 5063

Amblyomma americanum americanum A. americanum -77.2277641 38.7042868 5063

Amblyomma americanum americanum A. americanum -77.2277641 38.7042868 5063

Amblyomma americanum americanum A. americanum -77.2277641 38.7042868 5063

Amblyomma americanum americanum A. americanum -77.2277641 38.7042868 5063

Amblyomma americanum americanum A. americanum -77.2277641 38.7042868 5063

Amblyomma americanum americanum A. americanum -77.2277641 38.7042868 5063

Amblyomma americanum americanum A. americanum -77.2277641 38.7042868 5063

Amblyomma americanum americanum A. americanum -77.2277641 38.7042868 5063

Amblyomma americanum americanum A. americanum -77.2277641 38.7042868 5063

Amblyomma americanum americanum A. americanum -77.2277641 38.7042868 5063

Amblyomma americanum americanum A. americanum -77.2277641 38.7042868 5063

Amblyomma americanum americanum A. americanum -77.2277641 38.7042868 5063

Amblyomma americanum americanum A. americanum -77.2277641 38.7042868 5063

Amblyomma americanum americanum A. americanum -77.2277641 38.7042868 5063

Amblyomma americanum americanum A. americanum -77.2277641 38.7042868 5063

Amblyomma americanum americanum A. americanum -77.2277641 38.7042868 5063

Amblyomma americanum americanum A. americanum -77.2277641 38.7042868 5063

Amblyomma americanum americanum A. americanum -77.2277641 38.7042868 5063

Amblyomma americanum americanum A. americanum -77.2277641 38.7042868 5063

Amblyomma americanum americanum A. americanum -77.2277641 38.7042868 5063

Amblyomma americanum americanum A. americanum -77.2277641 38.7042868 5063

Amblyomma americanum americanum A. americanum -77.2277641 38.7042868 5063

Amblyomma americanum americanum A. americanum -77.2277641 38.7042868 5063

Amblyomma americanum americanum A. americanum -77.2277641 38.7042868 5063

Amblyomma americanum americanum A. americanum -77.2277641 38.7042868 5063

Amblyomma americanum americanum A. americanum -77.2277641 38.7042868 5063

Amblyomma americanum americanum A. americanum -77.2277641 38.7042868 5063

Amblyomma americanum americanum A. americanum -77.2277641 38.7042868 5063

Amblyomma americanum americanum A. americanum -77.63694 39.2725 1471

Amblyomma americanum americanum A. americanum -78.01 35.64528 1333

Amblyomma americanum americanum A. americanum -80.68944 34.22722 6224

Amblyomma americanum americanum A. americanum -79.30111 37.14611 3036

Amblyomma americanum americanum A. americanum -86.7483368 34.6992588 5063

Amblyomma americanum americanum A. americanum -75.4760284 39.0712223 5062

Amblyomma americanum americanum A. americanum -75.4760284 39.0712223 5062

Amblyomma americanum americanum A. americanum -75.4760284 39.0712223 5062

Amblyomma americanum americanum A. americanum -75.4760284 39.0712223 5062

Amblyomma americanum americanum A. americanum -75.4760284 39.0712223 5062

Amblyomma americanum americanum A. americanum -77.4888616 38.7683483 7325

Amblyomma americanum americanum A. americanum -77.4888616 38.7683483 7325

Amblyomma americanum americanum A. americanum -77.4888616 38.7683483 7325

Amblyomma americanum americanum A. americanum -77.4888616 38.7683483 7325

Amblyomma americanum americanum A. americanum -77.4888616 38.7683483 7325

Amblyomma americanum americanum A. americanum -77.4888616 38.7683483 7325

Amblyomma americanum americanum A. americanum -77.4888616 38.7683483 7325

Amblyomma americanum americanum A. americanum -77.4888616 38.7683483 7325

Amblyomma americanum americanum A. americanum -77.4888616 38.7683483 7325

Amblyomma americanum americanum A. americanum -77.4888616 38.7683483 7325

Amblyomma americanum americanum A. americanum -77.4888616 38.7683483 7325

Amblyomma americanum americanum A. americanum -77.4888616 38.7683483 7325

Amblyomma americanum americanum A. americanum -77.4888616 38.7683483 7325

Amblyomma americanum americanum A. americanum -77.4888616 38.7683483 7325

Amblyomma americanum americanum A. americanum -77.4888616 38.7683483 7325

Amblyomma americanum americanum A. americanum -77.4888616 38.7683483 7325

Amblyomma americanum americanum A. americanum -77.4888616 38.7683483 7325

Amblyomma americanum americanum A. americanum -77.4888616 38.7683483 7325

Amblyomma americanum americanum A. americanum -77.4888616 38.7683483 7325

Amblyomma americanum americanum A. americanum -77.4888616 38.7683483 7325

Amblyomma americanum americanum A. americanum -77.4888616 38.7683483 7325

Amblyomma americanum americanum A. americanum -77.4888616 38.7683483 7325

Amblyomma americanum americanum A. americanum -77.4888616 38.7683483 7325

Amblyomma americanum americanum A. americanum -77.4888616 38.7683483 7325

Amblyomma americanum americanum A. americanum -77.4888616 38.7683483 7325

Amblyomma americanum americanum A. americanum -77.4888616 38.7683483 7325

Amblyomma americanum americanum A. americanum -77.4888616 38.7683483 7325

Amblyomma americanum americanum A. americanum -77.4888616 38.7683483 7325

Amblyomma americanum americanum A. americanum -77.4888616 38.7683483 7325

Amblyomma americanum americanum A. americanum -77.4888616 38.7683483 7325

Amblyomma americanum americanum A. americanum -77.4888616 38.7683483 7325

Amblyomma americanum americanum A. americanum -77.4888616 38.7683483 7325

Amblyomma americanum americanum A. americanum -77.4888616 38.7683483 7325

Amblyomma americanum americanum A. americanum -74.355812 39.9481742 5000

Amblyomma americanum americanum A. americanum -96.5724539 39.183609 5125

Amblyomma americanum americanum A. americanum -96.5724539 39.183609 5125

Amblyomma americanum americanum A. americanum -77.4888616 38.7683483 7325

Amblyomma americanum americanum A. americanum -78.73806 38.40944 5942

Amblyomma americanum americanum A. americanum -78.46389 38.92972 3036

Amblyomma americanum americanum A. americanum -74.5085906 40.0092093 18500

Amblyomma americanum americanum A. americanum -74.5085906 40.0092093 18500

Amblyomma americanum americanum A. americanum -74.82389 39.90083 3036

Amblyomma americanum americanum A. americanum -71.280988 41.545457 5639

Amblyomma americanum americanum A. americanum -71.280988 41.545457 5639

Amblyomma americanum americanum A. americanum -75.4406429 38.9278866 4972

Amblyomma americanum americanum A. americanum -77.77917 37.475 3036

Amblyomma americanum americanum A. americanum -77.0861053 38.7081464 588

Amblyomma americanum americanum A. americanum -85.99167 37.93694 2004

Amblyomma americanum americanum A. americanum -78.8936673 33.6948946 5178

Amblyomma americanum americanum A. americanum -71.966465 41.3585107 5442

Amblyomma americanum americanum A. americanum -71.8811798 41.4409332 5062

Amblyomma americanum americanum A. americanum -77.21722 38.45472 3036

Amblyomma americanum americanum A. americanum -86.7844429 36.1658897 5063

Amblyomma americanum americanum A. americanum -86.7844429 36.1658897 5063

Amblyomma americanum americanum A. americanum -86.7844429 36.1658897 5063

Amblyomma americanum americanum A. americanum -75.739352 39.6837215 5656

Amblyomma americanum americanum A. americanum -71.3128319 41.492733 5308

Amblyomma americanum americanum A. americanum -76.5028111 37.0918568 16209

Amblyomma americanum americanum A. americanum -76.5028111 37.0918568 16209

Amblyomma americanum americanum A. americanum -76.5028111 37.0918568 16209

Amblyomma americanum americanum A. americanum -76.5028111 37.0918568 16209

Amblyomma americanum americanum A. americanum -76.5028111 37.0918568 16209

Amblyomma americanum americanum A. americanum -76.5028111 37.0918568 16209

Amblyomma americanum americanum A. americanum -76.5028111 37.0918568 16209

Amblyomma americanum americanum A. americanum -76.5028111 37.0918568 16209

Amblyomma americanum americanum A. americanum -76.5028111 37.0918568 16209

Amblyomma americanum americanum A. americanum -76.5028111 37.0918568 16209

Amblyomma americanum americanum A. americanum -76.5028111 37.0918568 16209

Amblyomma americanum americanum A. americanum -76.5028111 37.0918568 16209

Amblyomma americanum americanum A. americanum -76.5028111 37.0918568 16209

Amblyomma americanum americanum A. americanum -76.5028111 37.0918568 16209

Amblyomma americanum americanum A. americanum -76.5028111 37.0918568 16209

Amblyomma americanum americanum A. americanum -76.5028111 37.0918568 16209

Amblyomma americanum americanum A. americanum -76.5028111 37.0918568 16209

Amblyomma americanum americanum A. americanum -76.5028111 37.0918568 16209

Amblyomma americanum americanum A. americanum -76.5028111 37.0918568 16209

Amblyomma americanum americanum A. americanum -76.5028111 37.0918568 16209

Amblyomma americanum americanum A. americanum -76.5028111 37.0918568 16209

Amblyomma americanum americanum A. americanum -72.1931343 41.328896 5289

Amblyomma americanum americanum A. americanum -77.5781676 38.6987267 5186

Amblyomma americanum americanum A. americanum -77.5781676 38.6987267 5186

Amblyomma americanum americanum A. americanum -77.5781676 38.6987267 5186

Amblyomma americanum americanum A. americanum -77.5781676 38.6987267 5186

Amblyomma americanum americanum A. americanum -77.5781676 38.6987267 5186

Amblyomma americanum americanum A. americanum -76.40667 37.44417 3036

Amblyomma americanum americanum A. americanum -75.9413338 39.6001148 5062

Amblyomma americanum americanum A. americanum -75.9413338 39.6001148 5062

Amblyomma americanum americanum A. americanum -75.9413338 39.6001148 5062

Amblyomma americanum americanum A. americanum -70.54528 44.21389 4313

Amblyomma americanum americanum A. americanum -85.31056 36.50778 3036

Amblyomma americanum americanum A. americanum -77.26056 38.68361 653

Amblyomma americanum americanum A. americanum -74.46083 39.47111 3036

Amblyomma americanum americanum A. americanum -76.7002449 39.0839996 5062

Amblyomma americanum americanum A. americanum -76.7002449 39.0839996 5062

Amblyomma americanum americanum A. americanum -76.7002449 39.0839996 5062

Amblyomma americanum americanum A. americanum -76.7002449 39.0839996 5062

Amblyomma americanum americanum A. americanum -76.7002449 39.0839996 5062

Amblyomma americanum americanum A. americanum -76.7002449 39.0839996 5062

Amblyomma americanum americanum A. americanum -76.7002449 39.0839996 5062

Amblyomma americanum americanum A. americanum -82.59194 28.19361 4740

Amblyomma americanum americanum A. americanum -94.81889 38.88139 12703

Amblyomma americanum americanum A. americanum -85.8571243 37.9095336 21000

Amblyomma americanum americanum A. americanum -85.8571243 37.9095336 21000

Amblyomma americanum americanum A. americanum -86.495307 30.522641 5000

Amblyomma americanum americanum A. americanum -76.1647796 39.4442798 12000

Amblyomma americanum americanum A. americanum -76.1647796 39.4442798 12000

Amblyomma americanum americanum A. americanum -76.1647796 39.4442798 12000

Amblyomma americanum americanum A. americanum -76.1647796 39.4442798 12000

Amblyomma americanum americanum A. americanum -77.2750854 38.1097082 13000

Amblyomma americanum americanum A. americanum -77.2750854 38.1097082 13000

Amblyomma americanum americanum A. americanum -77.2750854 38.1097082 13000

Amblyomma americanum americanum A. americanum -77.2750854 38.1097082 13000

Amblyomma americanum americanum A. americanum -87.6159667 36.6078112 18500

Amblyomma americanum americanum A. americanum -87.6159667 36.6078112 18500

Amblyomma americanum americanum A. americanum -92.1572583 37.7057025 16307

Amblyomma americanum americanum A. americanum -77.1459102 38.7021234 3500

Amblyomma americanum americanum A. americanum -92.1572583 37.7057025 16307

Amblyomma americanum americanum A. americanum -92.1572583 37.7057025 16307

Amblyomma americanum americanum A. americanum -87.6159667 36.6078112 18500

Amblyomma americanum americanum A. americanum -77.2750854 38.1097082 13000

Amblyomma americanum americanum A. americanum -77.2750854 38.1097082 13000

Amblyomma americanum americanum A. americanum -77.2750854 38.1097082 13000

Amblyomma americanum americanum A. americanum -77.2750854 38.1097082 13000

Amblyomma americanum americanum A. americanum -77.2750854 38.1097082 13000

Amblyomma americanum americanum A. americanum -77.2750854 38.1097082 13000

Amblyomma americanum americanum A. americanum -77.2750854 38.1097082 13000

Amblyomma americanum americanum A. americanum -77.2750854 38.1097082 13000

Amblyomma americanum americanum A. americanum -77.2750854 38.1097082 13000

Amblyomma americanum americanum A. americanum -76.1647796 39.4442798 12000

Amblyomma americanum americanum A. americanum -77.915039 37.0420244 10500

Amblyomma americanum americanum A. americanum -85.7496724 31.3992165 16240

Amblyomma americanum americanum A. americanum -76.1647796 39.4442798 12000

Amblyomma americanum americanum A. americanum -87.6159667 36.6078112 18500

Amblyomma americanum americanum A. americanum -92.1572583 37.7057025 16307

Amblyomma americanum americanum A. americanum -92.1572583 37.7057025 16307

Amblyomma americanum americanum A. americanum -92.1572583 37.7057025 16307

Amblyomma americanum americanum A. americanum -76.1647796 39.4442798 12000

Amblyomma americanum americanum A. americanum -87.6159667 36.6078112 18500

Amblyomma americanum americanum A. americanum -77.2750854 38.1097082 13000

Amblyomma americanum americanum A. americanum -77.2750854 38.1097082 13000

Amblyomma americanum americanum A. americanum -77.2750854 38.1097082 13000

Amblyomma americanum americanum A. americanum -77.2750854 38.1097082 13000

Amblyomma americanum americanum A. americanum -77.2750854 38.1097082 13000

Amblyomma americanum americanum A. americanum -77.2750854 38.1097082 13000

Amblyomma americanum americanum A. americanum -77.2750854 38.1097082 13000

Amblyomma americanum americanum A. americanum -77.2750854 38.1097082 13000

Amblyomma americanum americanum A. americanum -77.2750854 38.1097082 13000

Amblyomma americanum americanum A. americanum -77.2750854 38.1097082 13000

Amblyomma americanum americanum A. americanum -80.822222 34.039167 12000

Amblyomma americanum americanum A. americanum -96.8206687 39.1865859 20922

Amblyomma americanum americanum A. americanum -96.8206687 39.1865859 20922

Amblyomma americanum americanum A. americanum -76.84515 39.035445 2000

Amblyomma americanum americanum A. americanum -87.6159667 36.6078112 18500

Amblyomma americanum americanum A. americanum -74.1514316 40.2555169 6602

Amblyomma americanum americanum A. americanum -74.6167373 40.0086834 6500

Amblyomma americanum americanum A. americanum -74.6167373 40.0086834 6500

Amblyomma americanum americanum A. americanum -74.6167373 40.0086834 6500

Amblyomma americanum americanum A. americanum -74.6167373 40.0086834 6500

Amblyomma americanum americanum A. americanum -87.6159667 36.6078112 18500

Amblyomma americanum americanum A. americanum -76.1647796 39.4442798 12000

Amblyomma americanum americanum A. americanum -77.1459102 38.7021234 3500

Amblyomma americanum americanum A. americanum -87.6159667 36.6078112 18500

Amblyomma americanum americanum A. americanum -87.6159667 36.6078112 18500

Amblyomma americanum americanum A. americanum -77.2750854 38.1097082 13000

Amblyomma americanum americanum A. americanum -77.2750854 38.1097082 13000

Amblyomma americanum americanum A. americanum -77.2750854 38.1097082 13000

Amblyomma americanum americanum A. americanum -76.1647796 39.4442798 12000

Amblyomma americanum americanum A. americanum -76.1647796 39.4442798 12000

Amblyomma americanum americanum A. americanum -76.1647796 39.4442798 12000

Amblyomma americanum americanum A. americanum -76.1647796 39.4442798 12000

Amblyomma americanum americanum A. americanum -76.1647796 39.4442798 12000

Amblyomma americanum americanum A. americanum -85.8571243 37.9095336 21000

Amblyomma americanum americanum A. americanum -88.555418 32.551715 5000

Amblyomma americanum americanum A. americanum -76.5877532 37.1534759 9500

Amblyomma americanum americanum A. americanum -92.1572583 37.7057025 16307

Amblyomma americanum americanum A. americanum -76.1647796 39.4442798 12000

Amblyomma americanum americanum A. americanum -87.6159667 36.6078112 18500

Amblyomma americanum americanum A. americanum -87.6159667 36.6078112 18500

Amblyomma americanum americanum A. americanum -96.8206687 39.1865859 20922

Amblyomma americanum americanum A. americanum -96.8206687 39.1865859 20922

Amblyomma americanum americanum A. americanum -92.1572583 37.7057025 16307

Amblyomma americanum americanum A. americanum -76.1647796 39.4442798 12000

Amblyomma americanum americanum A. americanum -118.719744 38.565549 15000

Amblyomma americanum americanum A. americanum -76.7734909 39.0642483 10000

Amblyomma americanum americanum A. americanum -76.1647796 39.4442798 12000

Amblyomma americanum americanum A. americanum -87.6159667 36.6078112 18500

Amblyomma americanum americanum A. americanum -87.6159667 36.6078112 18500

Amblyomma americanum americanum A. americanum -87.6159667 36.6078112 18500

Amblyomma americanum americanum A. americanum -87.6159667 36.6078112 18500

Amblyomma americanum americanum A. americanum -87.6159667 36.6078112 18500

Amblyomma americanum americanum A. americanum -80.822222 34.039167 12000

Amblyomma americanum americanum A. americanum -85.8571243 37.9095336 21000

Amblyomma americanum americanum A. americanum -77.1459102 38.7021234 3500

Amblyomma americanum americanum A. americanum -87.6159667 36.6078112 18500

Amblyomma americanum americanum A. americanum -87.6159667 36.6078112 18500

Amblyomma americanum americanum A. americanum -76.1647796 39.4442798 12000

Amblyomma americanum americanum A. americanum -76.1647796 39.4442798 12000

Amblyomma americanum americanum A. americanum -87.6159667 36.6078112 18500

Amblyomma americanum americanum A. americanum -87.6159667 36.6078112 18500

Amblyomma americanum americanum A. americanum -74.370593 40.029739 6000

Amblyomma americanum americanum A. americanum -76.1647796 39.4442798 12000

Amblyomma americanum americanum A. americanum -79.1970062 35.1109218 17000

Amblyomma americanum americanum A. americanum -77.4312973 39.4391096 3000

Amblyomma americanum americanum A. americanum -92.1572583 37.7057025 16307

Amblyomma americanum americanum A. americanum -76.1647796 39.4442798 12000

Amblyomma americanum americanum A. americanum -87.6159667 36.6078112 18500

Amblyomma americanum americanum A. americanum -95.923889 34.826667 12000

Amblyomma americanum americanum A. americanum -76.1647796 39.4442798 12000

Amblyomma americanum americanum A. americanum -76.1647796 39.4442798 12000

Amblyomma americanum americanum A. americanum -85.8571243 37.9095336 21000

Amblyomma americanum americanum A. americanum -76.7734909 39.0642483 10000

Amblyomma americanum americanum A. americanum -74.1514316 40.2555169 40840

Amblyomma americanum americanum A. americanum -76.1647796 39.4442798 12000

Amblyomma americanum americanum A. americanum -87.6159667 36.6078112 18500

Amblyomma americanum americanum A. americanum -87.6159667 36.6078112 18500

Amblyomma americanum americanum A. americanum -77.2750854 38.1097082 13000

Amblyomma americanum americanum A. americanum -77.0184516 38.8393064 3500

Amblyomma americanum americanum A. americanum -87.6159667 36.6078112 18500

Amblyomma americanum americanum A. americanum -87.6159667 36.6078112 18500

Amblyomma americanum americanum A. americanum -87.6159667 36.6078112 18500

Amblyomma americanum americanum A. americanum -77.2750854 38.1097082 13000

Amblyomma americanum americanum A. americanum -92.1572583 37.7057025 16307

Amblyomma americanum americanum A. americanum -76.1647796 39.4442798 12000

Amblyomma americanum americanum A. americanum -87.6159667 36.6078112 18500

Amblyomma americanum americanum A. americanum -87.6159667 36.6078112 18500

Amblyomma americanum americanum A. americanum -74.370593 40.029739 6000

Amblyomma americanum americanum A. americanum -85.7496724 31.3992165 16240

Amblyomma americanum americanum A. americanum -76.84515 39.035445 2000

Amblyomma americanum americanum A. americanum -76.1647796 39.4442798 12000

Amblyomma americanum americanum A. americanum -76.1647796 39.4442798 12000

Amblyomma americanum americanum A. americanum -87.6159667 36.6078112 18500

Amblyomma americanum americanum A. americanum -96.8206687 39.1865859 20922

Amblyomma americanum americanum A. americanum -76.1647796 39.4442798 12000

Amblyomma americanum americanum A. americanum -76.1647796 39.4442798 12000

Amblyomma americanum americanum A. americanum -76.1647796 39.4442798 12000

Amblyomma americanum americanum A. americanum -76.1647796 39.4442798 12000

Amblyomma americanum americanum A. americanum -77.1459102 38.7021234 3500

Amblyomma americanum americanum A. americanum -85.8571243 37.9095336 21000

Amblyomma americanum americanum A. americanum -92.1572583 37.7057025 16307

Amblyomma americanum americanum A. americanum -92.1572583 37.7057025 16307

Amblyomma americanum americanum A. americanum -92.1572583 37.7057025 16307

Amblyomma americanum americanum A. americanum -92.1572583 37.7057025 16307

Amblyomma americanum americanum A. americanum -87.6159667 36.6078112 18500

Amblyomma americanum americanum A. americanum -87.6159667 36.6078112 18500

Amblyomma americanum americanum A. americanum -87.6159667 36.6078112 18500

Amblyomma americanum americanum A. americanum -87.6159667 36.6078112 18500

Amblyomma americanum americanum A. americanum -92.1572583 37.7057025 16307

Amblyomma americanum americanum A. americanum -92.1572583 37.7057025 16307

Amblyomma americanum americanum A. americanum -92.1572583 37.7057025 16307

Amblyomma americanum americanum A. americanum -76.1647796 39.4442798 12000

Amblyomma americanum americanum A. americanum -76.1647796 39.4442798 12000

Amblyomma americanum americanum A. americanum -76.1647796 39.4442798 12000

Amblyomma americanum americanum A. americanum -76.1647796 39.4442798 12000

Amblyomma americanum americanum A. americanum -76.1647796 39.4442798 12000

Amblyomma americanum americanum A. americanum -76.1647796 39.4442798 12000

Amblyomma americanum americanum A. americanum -76.1647796 39.4442798 12000

Amblyomma americanum americanum A. americanum -92.1572583 37.7057025 16307

Amblyomma americanum americanum A. americanum -92.1572583 37.7057025 16307

Amblyomma americanum americanum A. americanum -92.1572583 37.7057025 16307

Amblyomma americanum americanum A. americanum -92.1572583 37.7057025 16307

Amblyomma americanum americanum A. americanum -92.1572583 37.7057025 16307

Amblyomma americanum americanum A. americanum -92.1572583 37.7057025 16307

Amblyomma americanum americanum A. americanum -92.1572583 37.7057025 16307

Amblyomma americanum americanum A. americanum -92.1572583 37.7057025 16307

Amblyomma americanum americanum A. americanum -76.1647796 39.4442798 12000

Amblyomma americanum americanum A. americanum -76.1647796 39.4442798 12000

Amblyomma americanum americanum A. americanum -76.1647796 39.4442798 12000

Amblyomma americanum americanum A. americanum -74.370593 40.029739 6000

Amblyomma americanum americanum A. americanum -74.1514316 40.2555169 40840

Amblyomma americanum americanum A. americanum -87.6159667 36.6078112 18500

Amblyomma americanum americanum A. americanum -87.6159667 36.6078112 18500

Amblyomma americanum americanum A. americanum -87.6159667 36.6078112 18500

Amblyomma americanum americanum A. americanum -87.6159667 36.6078112 18500

Amblyomma americanum americanum A. americanum -87.6159667 36.6078112 18500

Amblyomma americanum americanum A. americanum -87.6159667 36.6078112 18500

Amblyomma americanum americanum A. americanum -77.3341369 37.2359319 4000

Amblyomma americanum americanum A. americanum -77.3341369 37.2359319 4000

Amblyomma americanum americanum A. americanum -77.915039 37.0420244 10500

Amblyomma americanum americanum A. americanum -77.915039 37.0420244 10500

Amblyomma americanum americanum A. americanum -77.915039 37.0420244 10500

Amblyomma americanum americanum A. americanum -77.915039 37.0420244 10500

Amblyomma americanum americanum A. americanum -77.915039 37.0420244 10500

Amblyomma americanum americanum A. americanum -77.915039 37.0420244 10500

Amblyomma americanum americanum A. americanum -71.289167 42.47 3000

Amblyomma americanum americanum A. americanum -77.915039 37.0420244 10500

Amblyomma americanum americanum A. americanum -76.1647796 39.4442798 12000

Amblyomma americanum americanum A. americanum -76.1647796 39.4442798 12000

Amblyomma americanum americanum A. americanum -76.1647796 39.4442798 12000

Amblyomma americanum americanum A. americanum -76.1647796 39.4442798 12000

Amblyomma americanum americanum A. americanum -79.1970062 35.1109218 17000

Amblyomma americanum americanum A. americanum -77.3341369 37.2359319 4000

Amblyomma americanum americanum A. americanum -77.3341369 37.2359319 4000

Amblyomma americanum americanum A. americanum -92.1395874 34.9039529 5500

Amblyomma americanum americanum A. americanum -77.975979 34.007135 5000

Amblyomma americanum americanum A. americanum -76.1647796 39.4442798 12000

Amblyomma americanum americanum A. americanum -76.1647796 39.4442798 12000

Amblyomma americanum americanum A. americanum -76.1647796 39.4442798 12000

Amblyomma americanum americanum A. americanum -76.1647796 39.4442798 12000

Amblyomma americanum americanum A. americanum -76.1647796 39.4442798 12000

Amblyomma americanum americanum A. americanum -76.1647796 39.4442798 12000

Amblyomma americanum americanum A. americanum -76.1647796 39.4442798 12000

Amblyomma americanum americanum A. americanum -76.1647796 39.4442798 12000

Amblyomma americanum americanum A. americanum -76.1647796 39.4442798 12000

Amblyomma americanum americanum A. americanum -74.576966 40.024197 8000

Amblyomma americanum americanum A. americanum -74.1514316 40.2555169 40840

Amblyomma americanum americanum A. americanum -76.1647796 39.4442798 12000

Amblyomma americanum americanum A. americanum -76.1647796 39.4442798 12000

Amblyomma americanum americanum A. americanum -76.1647796 39.4442798 12000

Amblyomma americanum americanum A. americanum -76.1647796 39.4442798 12000

Amblyomma americanum americanum A. americanum -76.1647796 39.4442798 12000

Amblyomma americanum americanum A. americanum -85.8571243 37.9095336 21000

Amblyomma americanum americanum A. americanum -86.6569934 34.6324885 12772

Amblyomma americanum americanum A. americanum -76.1647796 39.4442798 12000

Amblyomma americanum americanum A. americanum -76.1647796 39.4442798 12000

Amblyomma americanum americanum A. americanum -76.1647796 39.4442798 12000

Amblyomma americanum americanum A. americanum -76.1647796 39.4442798 12000

Amblyomma americanum americanum A. americanum -87.6159667 36.6078112 18500

Amblyomma americanum americanum A. americanum -87.6159667 36.6078112 18500

Amblyomma americanum americanum A. americanum -87.6159667 36.6078112 18500

Amblyomma americanum americanum A. americanum -77.915039 37.0420244 10500

Amblyomma americanum americanum A. americanum -77.915039 37.0420244 10500

Amblyomma americanum americanum A. americanum -77.915039 37.0420244 10500

Amblyomma americanum americanum A. americanum -76.1647796 39.4442798 12000

Amblyomma americanum americanum A. americanum -76.1647796 39.4442798 12000

Amblyomma americanum americanum A. americanum -76.1647796 39.4442798 12000

Amblyomma americanum americanum A. americanum -76.1647796 39.4442798 12000

Amblyomma americanum americanum A. americanum -76.1647796 39.4442798 12000

Amblyomma americanum americanum A. americanum -76.1647796 39.4442798 12000

Amblyomma americanum americanum A. americanum -76.1647796 39.4442798 12000

Amblyomma americanum americanum A. americanum -77.1459102 38.7021234 3500

Amblyomma americanum americanum A. americanum -80.822222 34.039167 12000

Amblyomma americanum americanum A. americanum -76.8773459 38.8066212 3000

Amblyomma americanum americanum A. americanum -92.304301 34.914329 12000

Amblyomma americanum americanum A. americanum -92.1572583 37.7057025 16307

Amblyomma americanum americanum A. americanum -92.1572583 37.7057025 16307

Amblyomma americanum americanum A. americanum -92.1572583 37.7057025 16307

Amblyomma americanum americanum A. americanum -92.1572583 37.7057025 16307

Amblyomma americanum americanum A. americanum -92.1572583 37.7057025 16307

Amblyomma americanum americanum A. americanum -92.1572583 37.7057025 16307

Amblyomma americanum americanum A. americanum -76.1647796 39.4442798 12000

Amblyomma americanum americanum A. americanum -76.1647796 39.4442798 12000

Amblyomma americanum americanum A. americanum -76.1647796 39.4442798 12000

Amblyomma americanum americanum A. americanum -76.1647796 39.4442798 12000

Amblyomma americanum americanum A. americanum -76.1647796 39.4442798 12000

Amblyomma americanum americanum A. americanum -76.1647796 39.4442798 12000

Amblyomma americanum americanum A. americanum -76.1647796 39.4442798 12000

Amblyomma americanum americanum A. americanum -76.1647796 39.4442798 12000

Amblyomma americanum americanum A. americanum -76.1647796 39.4442798 12000

Amblyomma americanum americanum A. americanum -76.1647796 39.4442798 12000

Amblyomma americanum americanum A. americanum -76.1647796 39.4442798 12000

Amblyomma americanum americanum A. americanum -76.1647796 39.4442798 12000

Amblyomma americanum americanum A. americanum -76.1647796 39.4442798 12000

Amblyomma americanum americanum A. americanum -76.1647796 39.4442798 12000

Amblyomma americanum americanum A. americanum -76.1647796 39.4442798 12000

Amblyomma americanum americanum A. americanum -76.1647796 39.4442798 12000

Amblyomma americanum americanum A. americanum -76.1647796 39.4442798 12000

Amblyomma americanum americanum A. americanum -85.8571243 37.9095336 21000

Amblyomma americanum americanum A. americanum -74.370593 40.029739 6000

Amblyomma americanum americanum A. americanum -74.370593 40.029739 6000

Amblyomma americanum americanum A. americanum -74.370593 40.029739 6000

Amblyomma americanum americanum A. americanum -74.370593 40.029739 6000

Amblyomma americanum americanum A. americanum -74.370593 40.029739 6000

Amblyomma americanum americanum A. americanum -74.370593 40.029739 6000

Amblyomma americanum americanum A. americanum -74.370593 40.029739 6000

Amblyomma americanum americanum A. americanum -74.370593 40.029739 6000

Amblyomma americanum americanum A. americanum -74.370593 40.029739 6000

Amblyomma americanum americanum A. americanum -74.370593 40.029739 6000

Amblyomma americanum americanum A. americanum -86.6569934 34.6324885 12772

Amblyomma americanum americanum A. americanum -76.1647796 39.4442798 12000

Amblyomma americanum americanum A. americanum -77.1459102 38.7021234 3500

Amblyomma americanum americanum A. americanum -77.1459102 38.7021234 3500

Amblyomma americanum americanum A. americanum -77.1459102 38.7021234 3500

Amblyomma americanum americanum A. americanum -87.6159667 36.6078112 18500

Amblyomma americanum americanum A. americanum -85.8571243 37.9095336 21000

Amblyomma americanum americanum A. americanum -85.8571243 37.9095336 21000

Amblyomma americanum americanum A. americanum -76.7734909 39.0642483 10000

Amblyomma americanum americanum A. americanum -76.7734909 39.0642483 10000

Amblyomma americanum americanum A. americanum -76.7734909 39.0642483 10000

Amblyomma americanum americanum A. americanum -76.7734909 39.0642483 10000

Amblyomma americanum americanum A. americanum -74.1514316 40.2555169 40840

Amblyomma americanum americanum A. americanum -74.1514316 40.2555169 40840

Amblyomma americanum americanum A. americanum -74.1514316 40.2555169 40840

Amblyomma americanum americanum A. americanum -76.1647796 39.4442798 12000

Amblyomma americanum americanum A. americanum -80.027761 36.774436 5000

Amblyomma americanum americanum A. americanum -76.5877532 37.1534759 9500

Amblyomma americanum americanum A. americanum -80.822222 34.039167 12000

Amblyomma americanum americanum A. americanum -74.576966 40.024197 8000

Amblyomma americanum americanum A. americanum -85.8571243 37.9095336 21000

Amblyomma americanum americanum A. americanum -85.8571243 37.9095336 21000

Amblyomma americanum americanum A. americanum -77.3341369 37.2359319 4000

Amblyomma americanum americanum A. americanum -77.3341369 37.2359319 4000

Amblyomma americanum americanum A. americanum -77.3341369 37.2359319 4000

Amblyomma americanum americanum A. americanum -74.1514316 40.2555169 40840

Amblyomma americanum americanum A. americanum -76.1647796 39.4442798 12000

Amblyomma americanum americanum A. americanum -76.1647796 39.4442798 12000

Amblyomma americanum americanum A. americanum -76.1647796 39.4442798 12000

Amblyomma americanum americanum A. americanum -76.1647796 39.4442798 12000

Amblyomma americanum americanum A. americanum -76.1647796 39.4442798 12000

Amblyomma americanum americanum A. americanum -76.1647796 39.4442798 12000

Amblyomma americanum americanum A. americanum -76.1647796 39.4442798 12000

Amblyomma americanum americanum A. americanum -76.1647796 39.4442798 12000

Amblyomma americanum americanum A. americanum -76.1647796 39.4442798 12000

Amblyomma americanum americanum A. americanum -76.1647796 39.4442798 12000

Amblyomma americanum americanum A. americanum -76.1647796 39.4442798 12000

Amblyomma americanum americanum A. americanum -76.1647796 39.4442798 12000

Amblyomma americanum americanum A. americanum -76.1647796 39.4442798 12000

Amblyomma americanum americanum A. americanum -76.1647796 39.4442798 12000

Amblyomma americanum americanum A. americanum -87.6159667 36.6078112 18500

Amblyomma americanum americanum A. americanum -76.7734909 39.0642483 10000

Amblyomma americanum americanum A. americanum -76.1647796 39.4442798 12000

Amblyomma americanum americanum A. americanum -76.1647796 39.4442798 12000

Amblyomma americanum americanum A. americanum -74.370593 40.029739 6000

Amblyomma americanum americanum A. americanum -76.1647796 39.4442798 12000

Amblyomma americanum americanum A. americanum -76.1647796 39.4442798 12000

Amblyomma americanum americanum A. americanum -87.6159667 36.6078112 18500

Amblyomma americanum americanum A. americanum -87.6159667 36.6078112 18500

Amblyomma americanum americanum A. americanum -87.6159667 36.6078112 18500

Amblyomma americanum americanum A. americanum -77.2750854 38.1097082 13000

Amblyomma americanum americanum A. americanum -77.2750854 38.1097082 13000

Amblyomma americanum americanum A. americanum -77.2750854 38.1097082 13000

Amblyomma americanum americanum A. americanum -76.7734909 39.0642483 10000

Amblyomma americanum americanum A. americanum -76.7734909 39.0642483 10000

Amblyomma americanum americanum A. americanum -92.1572583 37.7057025 16307

Amblyomma americanum americanum A. americanum -92.1572583 37.7057025 16307

Amblyomma americanum americanum A. americanum -92.1572583 37.7057025 16307

Amblyomma americanum americanum A. americanum -92.1572583 37.7057025 16307

Amblyomma americanum americanum A. americanum -92.1572583 37.7057025 16307

Amblyomma americanum americanum A. americanum -92.1572583 37.7057025 16307

Amblyomma americanum americanum A. americanum -92.1572583 37.7057025 16307

Amblyomma americanum americanum A. americanum -92.1572583 37.7057025 16307

Amblyomma americanum americanum A. americanum -77.1459102 38.7021234 3500

Amblyomma americanum americanum A. americanum -77.1459102 38.7021234 3500

Amblyomma americanum americanum A. americanum -77.1459102 38.7021234 3500

Amblyomma americanum americanum A. americanum -87.6159667 36.6078112 18500

Amblyomma americanum americanum A. americanum -87.6159667 36.6078112 18500

Amblyomma americanum americanum A. americanum -80.719539 34.010264 3000

Amblyomma americanum americanum A. americanum -76.7734909 39.0642483 10000

Amblyomma americanum americanum A. americanum -76.1647796 39.4442798 12000

Amblyomma americanum americanum A. americanum -76.1647796 39.4442798 12000

Amblyomma americanum americanum A. americanum -76.1647796 39.4442798 12000

Amblyomma americanum americanum A. americanum -76.1647796 39.4442798 12000

Amblyomma americanum americanum A. americanum -77.3341369 37.2359319 4000

Amblyomma americanum americanum A. americanum -77.3341369 37.2359319 4000

Amblyomma americanum americanum A. americanum -76.1647796 39.4442798 12000

Amblyomma americanum americanum A. americanum -76.1647796 39.4442798 12000

Amblyomma americanum americanum A. americanum -76.1647796 39.4442798 12000

Amblyomma americanum americanum A. americanum -76.1647796 39.4442798 12000

Amblyomma americanum americanum A. americanum -76.1647796 39.4442798 12000

Amblyomma americanum americanum A. americanum -76.1647796 39.4442798 12000

Amblyomma americanum americanum A. americanum -76.5877532 37.1534759 9500

Amblyomma americanum americanum A. americanum -80.719539 34.010264 3000

Amblyomma americanum americanum A. americanum -76.1647796 39.4442798 12000

Amblyomma americanum americanum A. americanum -76.1647796 39.4442798 12000

Amblyomma americanum americanum A. americanum -76.1647796 39.4442798 12000

Amblyomma americanum americanum A. americanum -76.1647796 39.4442798 12000

Amblyomma americanum americanum A. americanum -76.1647796 39.4442798 12000

Amblyomma americanum americanum A. americanum -74.370593 40.029739 6000

Amblyomma americanum americanum A. americanum -77.915039 37.0420244 10500

Amblyomma americanum americanum A. americanum -77.915039 37.0420244 10500

Amblyomma americanum americanum A. americanum -77.915039 37.0420244 10500

Amblyomma americanum americanum A. americanum -77.915039 37.0420244 10500

Amblyomma americanum americanum A. americanum -77.915039 37.0420244 10500

Amblyomma americanum americanum A. americanum -77.915039 37.0420244 10500

Amblyomma americanum americanum A. americanum -77.915039 37.0420244 10500

Amblyomma americanum americanum A. americanum -77.915039 37.0420244 10500

Amblyomma americanum americanum A. americanum -77.915039 37.0420244 10500

Amblyomma americanum americanum A. americanum -77.915039 37.0420244 10500

Amblyomma americanum americanum A. americanum -77.915039 37.0420244 10500

Amblyomma americanum americanum A. americanum -77.915039 37.0420244 10500

Amblyomma americanum americanum A. americanum -77.915039 37.0420244 10500

Amblyomma americanum americanum A. americanum -80.027761 36.774436 5000

Amblyomma americanum americanum A. americanum -77.1459102 38.7021234 3500

Amblyomma americanum americanum A. americanum -87.6159667 36.6078112 18500

Amblyomma americanum americanum A. americanum -80.719539 34.010264 3000

Amblyomma americanum americanum A. americanum -80.719539 34.010264 3000

Amblyomma americanum americanum A. americanum -80.719539 34.010264 3000

Amblyomma americanum americanum A. americanum -96.8206687 39.1865859 20922

Amblyomma americanum americanum A. americanum -96.8206687 39.1865859 20922

Amblyomma americanum americanum A. americanum -76.1647796 39.4442798 12000

Amblyomma americanum americanum A. americanum -76.1647796 39.4442798 12000

Amblyomma americanum americanum A. americanum -76.5877532 37.1534759 9500

Amblyomma americanum americanum A. americanum -76.5877532 37.1534759 9500

Amblyomma americanum americanum A. americanum -76.5877532 37.1534759 9500

Amblyomma americanum americanum A. americanum -77.2750854 38.1097082 13000

Amblyomma americanum americanum A. americanum -77.2750854 38.1097082 13000

Amblyomma americanum americanum A. americanum -77.2750854 38.1097082 13000

Amblyomma americanum americanum A. americanum -77.2750854 38.1097082 13000

Amblyomma americanum americanum A. americanum -77.2750854 38.1097082 13000

Amblyomma americanum americanum A. americanum -77.2750854 38.1097082 13000

Amblyomma americanum americanum A. americanum -77.2750854 38.1097082 13000

Amblyomma americanum americanum A. americanum -77.2750854 38.1097082 13000

Amblyomma americanum americanum A. americanum -77.2750854 38.1097082 13000

Amblyomma americanum americanum A. americanum -77.2750854 38.1097082 13000

Amblyomma americanum americanum A. americanum -77.2750854 38.1097082 13000

Amblyomma americanum americanum A. americanum -77.2750854 38.1097082 13000

Amblyomma americanum americanum A. americanum -77.2750854 38.1097082 13000

Amblyomma americanum americanum A. americanum -77.2750854 38.1097082 13000

Amblyomma americanum americanum A. americanum -77.2750854 38.1097082 13000

Amblyomma americanum americanum A. americanum -77.2750854 38.1097082 13000

Amblyomma americanum americanum A. americanum -77.2750854 38.1097082 13000

Amblyomma americanum americanum A. americanum -77.2750854 38.1097082 13000

Amblyomma americanum americanum A. americanum -77.2750854 38.1097082 13000

Amblyomma americanum americanum A. americanum -77.2750854 38.1097082 13000

Amblyomma americanum americanum A. americanum -77.2750854 38.1097082 13000

Amblyomma americanum americanum A. americanum -77.2750854 38.1097082 13000

Amblyomma americanum americanum A. americanum -77.2750854 38.1097082 13000

Amblyomma americanum americanum A. americanum -77.2750854 38.1097082 13000

Amblyomma americanum americanum A. americanum -77.2750854 38.1097082 13000

Amblyomma americanum americanum A. americanum -77.2750854 38.1097082 13000

Amblyomma americanum americanum A. americanum -77.2750854 38.1097082 13000

Amblyomma americanum americanum A. americanum -77.2750854 38.1097082 13000

Amblyomma americanum americanum A. americanum -77.2750854 38.1097082 13000

Amblyomma americanum americanum A. americanum -77.2750854 38.1097082 13000

Amblyomma americanum americanum A. americanum -77.2750854 38.1097082 13000

Amblyomma americanum americanum A. americanum -77.2750854 38.1097082 13000

Amblyomma americanum americanum A. americanum -77.2750854 38.1097082 13000

Amblyomma americanum americanum A. americanum -77.2750854 38.1097082 13000

Amblyomma americanum americanum A. americanum -74.370593 40.029739 6000

Amblyomma americanum americanum A. americanum -77.3341369 37.2359319 4000

Amblyomma americanum americanum A. americanum -77.3341369 37.2359319 4000

Amblyomma americanum americanum A. americanum -77.3341369 37.2359319 4000

Amblyomma americanum americanum A. americanum -74.1514316 40.2555169 40840

Amblyomma americanum americanum A. americanum -74.1514316 40.2555169 40840

Amblyomma americanum americanum A. americanum -74.1514316 40.2555169 40840

Amblyomma americanum americanum A. americanum -77.4568391 38.5520281 15927

Amblyomma americanum americanum A. americanum -92.1572583 37.7057025 16307

Amblyomma americanum americanum A. americanum -92.1572583 37.7057025 16307

Amblyomma americanum americanum A. americanum -92.1572583 37.7057025 16307

Amblyomma americanum americanum A. americanum -92.1572583 37.7057025 16307

Amblyomma americanum americanum A. americanum -92.1572583 37.7057025 16307

Amblyomma americanum americanum A. americanum -92.1572583 37.7057025 16307

Amblyomma americanum americanum A. americanum -74.370593 40.029739 6000

Amblyomma americanum americanum A. americanum -92.1572583 37.7057025 16307

Amblyomma americanum americanum A. americanum -76.1647796 39.4442798 12000

Amblyomma americanum americanum A. americanum -87.6159667 36.6078112 18500

Amblyomma americanum americanum A. americanum -87.6159667 36.6078112 18500

Amblyomma americanum americanum A. americanum -74.6167373 40.0086834 6500

Amblyomma americanum americanum A. americanum -74.6167373 40.0086834 6500

Amblyomma americanum americanum A. americanum -74.6167373 40.0086834 6500

Amblyomma americanum americanum A. americanum -74.6167373 40.0086834 6500

Amblyomma americanum americanum A. americanum -74.6167373 40.0086834 6500

Amblyomma americanum americanum A. americanum -74.6167373 40.0086834 6500

Amblyomma americanum americanum A. americanum -74.6167373 40.0086834 6500

Amblyomma americanum americanum A. americanum -76.1647796 39.4442798 12000

Amblyomma americanum americanum A. americanum -74.1514316 40.2555169 40840

Amblyomma americanum americanum A. americanum -77.4568391 38.5520281 15927

Amblyomma americanum americanum A. americanum -77.4568391 38.5520281 15927

Amblyomma americanum americanum A. americanum -76.1647796 39.4442798 12000

Amblyomma americanum americanum A. americanum -77.1459102 38.7021234 3500

Amblyomma americanum americanum A. americanum -77.1459102 38.7021234 3500

Amblyomma americanum americanum A. americanum -77.1459102 38.7021234 3500

Amblyomma americanum americanum A. americanum -87.6159667 36.6078112 18500

Amblyomma americanum americanum A. americanum -79.070374 35.654428 2000

Amblyomma americanum americanum A. americanum -76.1647796 39.4442798 12000

Amblyomma americanum americanum A. americanum -76.1647796 39.4442798 12000

Amblyomma americanum americanum A. americanum -76.1647796 39.4442798 12000

Amblyomma americanum americanum A. americanum -76.1647796 39.4442798 12000

Amblyomma americanum americanum A. americanum -76.1647796 39.4442798 12000

Amblyomma americanum americanum A. americanum -87.6159667 36.6078112 18500

Amblyomma americanum americanum A. americanum -87.6159667 36.6078112 18500

Amblyomma americanum americanum A. americanum -87.6159667 36.6078112 18500

Amblyomma americanum americanum A. americanum -77.2750854 38.1097082 13000

Amblyomma americanum americanum A. americanum -77.2750854 38.1097082 13000

Amblyomma americanum americanum A. americanum -77.2750854 38.1097082 13000

Amblyomma americanum americanum A. americanum -77.2750854 38.1097082 13000

Amblyomma americanum americanum A. americanum -77.2750854 38.1097082 13000

Amblyomma americanum americanum A. americanum -77.2750854 38.1097082 13000

Amblyomma americanum americanum A. americanum -77.2750854 38.1097082 13000

Amblyomma americanum americanum A. americanum -77.2750854 38.1097082 13000

Amblyomma americanum americanum A. americanum -77.2750854 38.1097082 13000

Amblyomma americanum americanum A. americanum -77.2750854 38.1097082 13000

Amblyomma americanum americanum A. americanum -77.2750854 38.1097082 13000

Amblyomma americanum americanum A. americanum -77.2750854 38.1097082 13000

Amblyomma americanum americanum A. americanum -77.2750854 38.1097082 13000

Amblyomma americanum americanum A. americanum -77.2750854 38.1097082 13000

Amblyomma americanum americanum A. americanum -77.2750854 38.1097082 13000

Amblyomma americanum americanum A. americanum -77.2750854 38.1097082 13000

Amblyomma americanum americanum A. americanum -77.2750854 38.1097082 13000

Amblyomma americanum americanum A. americanum -77.2750854 38.1097082 13000

Amblyomma americanum americanum A. americanum -77.2750854 38.1097082 13000

Amblyomma americanum americanum A. americanum -77.2750854 38.1097082 13000

Amblyomma americanum americanum A. americanum -77.2750854 38.1097082 13000

Amblyomma americanum americanum A. americanum -77.2750854 38.1097082 13000

Amblyomma americanum americanum A. americanum -77.2750854 38.1097082 13000

Amblyomma americanum americanum A. americanum -77.2750854 38.1097082 13000

Amblyomma americanum americanum A. americanum -77.2750854 38.1097082 13000

Amblyomma americanum americanum A. americanum -77.2750854 38.1097082 13000

Amblyomma americanum americanum A. americanum -77.2750854 38.1097082 13000

Amblyomma americanum americanum A. americanum -77.2750854 38.1097082 13000

Amblyomma americanum americanum A. americanum -77.2750854 38.1097082 13000

Amblyomma americanum americanum A. americanum -77.2750854 38.1097082 13000

Amblyomma americanum americanum A. americanum -77.2750854 38.1097082 13000

Amblyomma americanum americanum A. americanum -77.2750854 38.1097082 13000

Amblyomma americanum americanum A. americanum -77.2750854 38.1097082 13000

Amblyomma americanum americanum A. americanum -77.2750854 38.1097082 13000

Amblyomma americanum americanum A. americanum -77.2750854 38.1097082 13000

Amblyomma americanum americanum A. americanum -77.2750854 38.1097082 13000

Amblyomma americanum americanum A. americanum -77.2750854 38.1097082 13000

Amblyomma americanum americanum A. americanum -77.2750854 38.1097082 13000

Amblyomma americanum americanum A. americanum -77.2750854 38.1097082 13000

Amblyomma americanum americanum A. americanum -77.2750854 38.1097082 13000

Amblyomma americanum americanum A. americanum -77.2750854 38.1097082 13000

Amblyomma americanum americanum A. americanum -77.2750854 38.1097082 13000

Amblyomma americanum americanum A. americanum -77.2750854 38.1097082 13000

Amblyomma americanum americanum A. americanum -77.2750854 38.1097082 13000

Amblyomma americanum americanum A. americanum -77.2750854 38.1097082 13000

Amblyomma americanum americanum A. americanum -77.2750854 38.1097082 13000

Amblyomma americanum americanum A. americanum -77.2750854 38.1097082 13000

Amblyomma americanum americanum A. americanum -77.2750854 38.1097082 13000

Amblyomma americanum americanum A. americanum -77.2750854 38.1097082 13000

Amblyomma americanum americanum A. americanum -77.2750854 38.1097082 13000

Amblyomma americanum americanum A. americanum -77.2750854 38.1097082 13000

Amblyomma americanum americanum A. americanum -77.2750854 38.1097082 13000

Amblyomma americanum americanum A. americanum -77.2750854 38.1097082 13000

Amblyomma americanum americanum A. americanum -77.2750854 38.1097082 13000

Amblyomma americanum americanum A. americanum -77.2750854 38.1097082 13000

Amblyomma americanum americanum A. americanum -77.2750854 38.1097082 13000

Amblyomma americanum americanum A. americanum -77.2750854 38.1097082 13000

Amblyomma americanum americanum A. americanum -77.2750854 38.1097082 13000

Amblyomma americanum americanum A. americanum -77.2750854 38.1097082 13000

Amblyomma americanum americanum A. americanum -77.2750854 38.1097082 13000

Amblyomma americanum americanum A. americanum -74.576966 40.024197 8000

Amblyomma americanum americanum A. americanum -74.576966 40.024197 8000

Amblyomma americanum americanum A. americanum -74.5435553 40.9548927 23433

Amblyomma americanum americanum A. americanum -76.7734909 39.0642483 10000

Amblyomma americanum americanum A. americanum -76.7734909 39.0642483 10000

Amblyomma americanum americanum A. americanum -76.7734909 39.0642483 10000

Amblyomma americanum americanum A. americanum -76.7734909 39.0642483 10000

Amblyomma americanum americanum A. americanum -76.7734909 39.0642483 10000

Amblyomma americanum americanum A. americanum -76.1647796 39.4442798 12000

Amblyomma americanum americanum A. americanum -76.1647796 39.4442798 12000

Amblyomma americanum americanum A. americanum -76.1647796 39.4442798 12000

Amblyomma americanum americanum A. americanum -76.1647796 39.4442798 12000

Amblyomma americanum americanum A. americanum -87.6159667 36.6078112 18500

Amblyomma americanum americanum A. americanum -94.9129486 39.3571307 6500

Amblyomma americanum americanum A. americanum -77.3341369 37.2359319 4000

Amblyomma americanum americanum A. americanum -74.1514316 40.2555169 40840

Amblyomma americanum americanum A. americanum -77.915039 37.0420244 10500

Amblyomma americanum americanum A. americanum -77.915039 37.0420244 10500

Amblyomma americanum americanum A. americanum -77.915039 37.0420244 10500

Amblyomma americanum americanum A. americanum -77.915039 37.0420244 10500

Amblyomma americanum americanum A. americanum -77.915039 37.0420244 10500

Amblyomma americanum americanum A. americanum -77.915039 37.0420244 10500

Amblyomma americanum americanum A. americanum -77.915039 37.0420244 10500

Amblyomma americanum americanum A. americanum -77.915039 37.0420244 10500

Amblyomma americanum americanum A. americanum -77.915039 37.0420244 10500

Amblyomma americanum americanum A. americanum -77.915039 37.0420244 10500

Amblyomma americanum americanum A. americanum -77.915039 37.0420244 10500

Amblyomma americanum americanum A. americanum -77.915039 37.0420244 10500

Amblyomma americanum americanum A. americanum -77.915039 37.0420244 10500

Amblyomma americanum americanum A. americanum -77.915039 37.0420244 10500

Amblyomma americanum americanum A. americanum -77.915039 37.0420244 10500

Amblyomma americanum americanum A. americanum -77.915039 37.0420244 10500

Amblyomma americanum americanum A. americanum -77.915039 37.0420244 10500

Amblyomma americanum americanum A. americanum -77.915039 37.0420244 10500

Amblyomma americanum americanum A. americanum -77.915039 37.0420244 10500

Amblyomma americanum americanum A. americanum -77.915039 37.0420244 10500

Amblyomma americanum americanum A. americanum -77.915039 37.0420244 10500

Amblyomma americanum americanum A. americanum -77.915039 37.0420244 10500

Amblyomma americanum americanum A. americanum -77.915039 37.0420244 10500

Amblyomma americanum americanum A. americanum -77.915039 37.0420244 10500

Amblyomma americanum americanum A. americanum -77.915039 37.0420244 10500

Amblyomma americanum americanum A. americanum -77.915039 37.0420244 10500

Amblyomma americanum americanum A. americanum -77.915039 37.0420244 10500

Amblyomma americanum americanum A. americanum -77.915039 37.0420244 10500

Amblyomma americanum americanum A. americanum -77.915039 37.0420244 10500

Amblyomma americanum americanum A. americanum -77.915039 37.0420244 10500

Amblyomma americanum americanum A. americanum -77.915039 37.0420244 10500

Amblyomma americanum americanum A. americanum -77.915039 37.0420244 10500

Amblyomma americanum americanum A. americanum -77.915039 37.0420244 10500

Amblyomma americanum americanum A. americanum -77.915039 37.0420244 10500

Amblyomma americanum americanum A. americanum -77.915039 37.0420244 10500

Amblyomma americanum americanum A. americanum -77.915039 37.0420244 10500

Amblyomma americanum americanum A. americanum -77.915039 37.0420244 10500

Amblyomma americanum americanum A. americanum -77.915039 37.0420244 10500

Amblyomma americanum americanum A. americanum -77.915039 37.0420244 10500

Amblyomma americanum americanum A. americanum -77.915039 37.0420244 10500

Amblyomma americanum americanum A. americanum -77.915039 37.0420244 10500

Amblyomma americanum americanum A. americanum -77.915039 37.0420244 10500

Amblyomma americanum americanum A. americanum -77.915039 37.0420244 10500

Amblyomma americanum americanum A. americanum -77.915039 37.0420244 10500

Amblyomma americanum americanum A. americanum -77.915039 37.0420244 10500

Amblyomma americanum americanum A. americanum -77.915039 37.0420244 10500

Amblyomma americanum americanum A. americanum -77.915039 37.0420244 10500

Amblyomma americanum americanum A. americanum -77.915039 37.0420244 10500

Amblyomma americanum americanum A. americanum -77.915039 37.0420244 10500

Amblyomma americanum americanum A. americanum -77.915039 37.0420244 10500

Amblyomma americanum americanum A. americanum -77.915039 37.0420244 10500

Amblyomma americanum americanum A. americanum -77.915039 37.0420244 10500

Amblyomma americanum americanum A. americanum -77.915039 37.0420244 10500

Amblyomma americanum americanum A. americanum -77.915039 37.0420244 10500

Amblyomma americanum americanum A. americanum -77.915039 37.0420244 10500

Amblyomma americanum americanum A. americanum -79.1970062 35.1109218 17000

Amblyomma americanum americanum A. americanum -86.5295486 30.572647 44515

Amblyomma americanum americanum A. americanum -86.5295486 30.572647 44515

Amblyomma americanum americanum A. americanum -86.5295486 30.572647 44515

Amblyomma americanum americanum A. americanum -77.2750854 38.1097082 13000

Amblyomma americanum americanum A. americanum -77.2750854 38.1097082 13000

Amblyomma americanum americanum A. americanum -77.2750854 38.1097082 13000

Amblyomma americanum americanum A. americanum -77.2750854 38.1097082 13000

Amblyomma americanum americanum A. americanum -77.2750854 38.1097082 13000

Amblyomma americanum americanum A. americanum -77.2750854 38.1097082 13000

Amblyomma americanum americanum A. americanum -77.2750854 38.1097082 13000

Amblyomma americanum americanum A. americanum -77.2750854 38.1097082 13000

Amblyomma americanum americanum A. americanum -77.2750854 38.1097082 13000

Amblyomma americanum americanum A. americanum -77.2750854 38.1097082 13000

Amblyomma americanum americanum A. americanum -77.2750854 38.1097082 13000

Amblyomma americanum americanum A. americanum -77.2750854 38.1097082 13000

Amblyomma americanum americanum A. americanum -77.2750854 38.1097082 13000

Amblyomma americanum americanum A. americanum -77.2750854 38.1097082 13000

Amblyomma americanum americanum A. americanum -77.2750854 38.1097082 13000

Amblyomma americanum americanum A. americanum -77.2750854 38.1097082 13000

Amblyomma americanum americanum A. americanum -77.2750854 38.1097082 13000

Amblyomma americanum americanum A. americanum -77.2750854 38.1097082 13000

Amblyomma americanum americanum A. americanum -77.2750854 38.1097082 13000

Amblyomma americanum americanum A. americanum -77.2750854 38.1097082 13000

Amblyomma americanum americanum A. americanum -77.2750854 38.1097082 13000

Amblyomma americanum americanum A. americanum -77.2750854 38.1097082 13000

Amblyomma americanum americanum A. americanum -77.2750854 38.1097082 13000

Amblyomma americanum americanum A. americanum -77.2750854 38.1097082 13000

Amblyomma americanum americanum A. americanum -77.2750854 38.1097082 13000

Amblyomma americanum americanum A. americanum -77.2750854 38.1097082 13000

Amblyomma americanum americanum A. americanum -77.2750854 38.1097082 13000

Amblyomma americanum americanum A. americanum -77.2750854 38.1097082 13000

Amblyomma americanum americanum A. americanum -77.2750854 38.1097082 13000

Amblyomma americanum americanum A. americanum -77.2750854 38.1097082 13000

Amblyomma americanum americanum A. americanum -77.2750854 38.1097082 13000

Amblyomma americanum americanum A. americanum -77.2750854 38.1097082 13000

Amblyomma americanum americanum A. americanum -77.2750854 38.1097082 13000

Amblyomma americanum americanum A. americanum -77.2750854 38.1097082 13000

Amblyomma americanum americanum A. americanum -77.2750854 38.1097082 13000

Amblyomma americanum americanum A. americanum -77.2750854 38.1097082 13000

Amblyomma americanum americanum A. americanum -77.2750854 38.1097082 13000

Amblyomma americanum americanum A. americanum -77.2750854 38.1097082 13000

Amblyomma americanum americanum A. americanum -74.1514316 40.2555169 40840

Amblyomma americanum americanum A. americanum -76.419739 38.2817689 24985

Amblyomma americanum americanum A. americanum -96.8206687 39.1865859 20922

Amblyomma americanum americanum A. americanum -76.1647796 39.4442798 12000

Amblyomma americanum americanum A. americanum -76.1647796 39.4442798 12000

Amblyomma americanum americanum A. americanum -76.1647796 39.4442798 12000

Amblyomma americanum americanum A. americanum -86.5295486 30.572647 44515

Amblyomma americanum americanum A. americanum -78.5825 35.941667 1000

Amblyomma americanum americanum A. americanum -96.8206687 39.1865859 20922

Amblyomma americanum americanum A. americanum -76.1647796 39.4442798 12000

Amblyomma americanum americanum A. americanum -75.4650878 39.1272638 3000

Amblyomma americanum americanum A. americanum -92.1572583 37.7057025 16307

Amblyomma americanum americanum A. americanum -76.1647796 39.4442798 12000

Amblyomma americanum americanum A. americanum -76.1647796 39.4442798 12000

Amblyomma americanum americanum A. americanum -76.1647796 39.4442798 12000

Amblyomma americanum americanum A. americanum -76.1647796 39.4442798 12000

Amblyomma americanum americanum A. americanum -77.3341369 37.2359319 4000

Amblyomma americanum americanum A. americanum -76.1647796 39.4442798 12000

Amblyomma americanum americanum A. americanum -76.1647796 39.4442798 12000

Amblyomma americanum americanum A. americanum -76.1647796 39.4442798 12000

Amblyomma americanum americanum A. americanum -76.1647796 39.4442798 12000

Amblyomma americanum americanum A. americanum -74.1514316 40.2555169 40840

Amblyomma americanum americanum A. americanum -77.915039 37.0420244 10500

Amblyomma americanum americanum A. americanum -87.6159667 36.6078112 18500

Amblyomma americanum americanum A. americanum -95.923889 34.826667 12000

Amblyomma americanum americanum A. americanum -92.1572583 37.7057025 16307

Amblyomma americanum americanum A. americanum -92.1572583 37.7057025 16307

Amblyomma americanum americanum A. americanum -92.1572583 37.7057025 16307

Amblyomma americanum americanum A. americanum -92.1572583 37.7057025 16307

Amblyomma americanum americanum A. americanum -92.1572583 37.7057025 16307

Amblyomma americanum americanum A. americanum -76.1647796 39.4442798 12000

Amblyomma americanum americanum A. americanum -76.1647796 39.4442798 12000

Amblyomma americanum americanum A. americanum -76.1647796 39.4442798 12000

Amblyomma americanum americanum A. americanum -76.5877532 37.1534759 9500

Amblyomma americanum americanum A. americanum -77.2750854 38.1097082 13000

Amblyomma americanum americanum A. americanum -77.2750854 38.1097082 13000

Amblyomma americanum americanum A. americanum -77.2750854 38.1097082 13000

Amblyomma americanum americanum A. americanum -77.2750854 38.1097082 13000

Amblyomma americanum americanum A. americanum -77.2750854 38.1097082 13000

Amblyomma americanum americanum A. americanum -77.2750854 38.1097082 13000

Amblyomma americanum americanum A. americanum -77.2750854 38.1097082 13000

Amblyomma americanum americanum A. americanum -77.2750854 38.1097082 13000

Amblyomma americanum americanum A. americanum -77.2750854 38.1097082 13000

Amblyomma americanum americanum A. americanum -77.2750854 38.1097082 13000

Amblyomma americanum americanum A. americanum -77.2750854 38.1097082 13000

Amblyomma americanum americanum A. americanum -77.2750854 38.1097082 13000

Amblyomma americanum americanum A. americanum -77.2750854 38.1097082 13000

Amblyomma americanum americanum A. americanum -77.2750854 38.1097082 13000

Amblyomma americanum americanum A. americanum -77.2750854 38.1097082 13000

Amblyomma americanum americanum A. americanum -77.2750854 38.1097082 13000

Amblyomma americanum americanum A. americanum -77.2750854 38.1097082 13000

Amblyomma americanum americanum A. americanum -77.2750854 38.1097082 13000

Amblyomma americanum americanum A. americanum -77.2750854 38.1097082 13000

Amblyomma americanum americanum A. americanum -77.2750854 38.1097082 13000

Amblyomma americanum americanum A. americanum -77.2750854 38.1097082 13000

Amblyomma americanum americanum A. americanum -77.2750854 38.1097082 13000

Amblyomma americanum americanum A. americanum -77.2750854 38.1097082 13000

Amblyomma americanum americanum A. americanum -77.2750854 38.1097082 13000

Amblyomma americanum americanum A. americanum -77.2750854 38.1097082 13000

Amblyomma americanum americanum A. americanum -77.2750854 38.1097082 13000

Amblyomma americanum americanum A. americanum -77.2750854 38.1097082 13000

Amblyomma americanum americanum A. americanum -77.2750854 38.1097082 13000

Amblyomma americanum americanum A. americanum -77.2750854 38.1097082 13000

Amblyomma americanum americanum A. americanum -77.2750854 38.1097082 13000

Amblyomma americanum americanum A. americanum -85.8571243 37.9095336 21000

Amblyomma americanum americanum A. americanum -85.8571243 37.9095336 21000

Amblyomma americanum americanum A. americanum -77.3341369 37.2359319 4000

Amblyomma americanum americanum A. americanum -76.1647796 39.4442798 12000

Amblyomma americanum americanum A. americanum -77.1459102 38.7021234 3500

Amblyomma americanum americanum A. americanum -87.6159667 36.6078112 18500

Amblyomma americanum americanum A. americanum -76.1647796 39.4442798 12000

Amblyomma americanum americanum A. americanum -74.576966 40.024197 8000

Amblyomma americanum americanum A. americanum -74.576966 40.024197 8000

Amblyomma americanum americanum A. americanum -74.576966 40.024197 8000

Amblyomma americanum americanum A. americanum -77.915039 37.0420244 10500

Amblyomma americanum americanum A. americanum -77.915039 37.0420244 10500

Amblyomma americanum americanum A. americanum -77.915039 37.0420244 10500

Amblyomma americanum americanum A. americanum -77.915039 37.0420244 10500

Amblyomma americanum americanum A. americanum -77.915039 37.0420244 10500

Amblyomma americanum americanum A. americanum -77.915039 37.0420244 10500

Amblyomma americanum americanum A. americanum -77.915039 37.0420244 10500

Amblyomma americanum americanum A. americanum -92.1572583 37.7057025 16307

Amblyomma americanum americanum A. americanum -92.1572583 37.7057025 16307

Amblyomma americanum americanum A. americanum -84.047648 39.818854 5000

Amblyomma americanum americanum A. americanum -76.1647796 39.4442798 12000

Amblyomma americanum americanum A. americanum -76.1647796 39.4442798 12000

Amblyomma americanum americanum A. americanum -76.1647796 39.4442798 12000

Amblyomma americanum americanum A. americanum -77.915039 37.0420244 10500

Amblyomma americanum americanum A. americanum -77.915039 37.0420244 10500

Amblyomma americanum americanum A. americanum -77.915039 37.0420244 10500

Amblyomma americanum americanum A. americanum -77.915039 37.0420244 10500

Amblyomma americanum americanum A. americanum -77.915039 37.0420244 10500

Amblyomma americanum americanum A. americanum -77.915039 37.0420244 10500

Amblyomma americanum americanum A. americanum -77.915039 37.0420244 10500

Amblyomma americanum americanum A. americanum -77.915039 37.0420244 10500

Amblyomma americanum americanum A. americanum -77.915039 37.0420244 10500

Amblyomma americanum americanum A. americanum -77.915039 37.0420244 10500

Amblyomma americanum americanum A. americanum -77.915039 37.0420244 10500

Amblyomma americanum americanum A. americanum -77.915039 37.0420244 10500

Amblyomma americanum americanum A. americanum -77.915039 37.0420244 10500

Amblyomma americanum americanum A. americanum -77.915039 37.0420244 10500

Amblyomma americanum americanum A. americanum -77.915039 37.0420244 10500

Amblyomma americanum americanum A. americanum -77.915039 37.0420244 10500

Amblyomma americanum americanum A. americanum -77.915039 37.0420244 10500

Amblyomma americanum americanum A. americanum -77.915039 37.0420244 10500

Amblyomma americanum americanum A. americanum -77.915039 37.0420244 10500

Amblyomma americanum americanum A. americanum -77.915039 37.0420244 10500

Amblyomma americanum americanum A. americanum -77.915039 37.0420244 10500

Amblyomma americanum americanum A. americanum -77.915039 37.0420244 10500

Amblyomma americanum americanum A. americanum -77.915039 37.0420244 10500

Amblyomma americanum americanum A. americanum -77.915039 37.0420244 10500

Amblyomma americanum americanum A. americanum -77.915039 37.0420244 10500

Amblyomma americanum americanum A. americanum -77.915039 37.0420244 10500

Amblyomma americanum americanum A. americanum -77.915039 37.0420244 10500

Amblyomma americanum americanum A. americanum -77.915039 37.0420244 10500

Amblyomma americanum americanum A. americanum -77.915039 37.0420244 10500

Amblyomma americanum americanum A. americanum -77.915039 37.0420244 10500

Amblyomma americanum americanum A. americanum -77.915039 37.0420244 10500

Amblyomma americanum americanum A. americanum -77.915039 37.0420244 10500

Amblyomma americanum americanum A. americanum -77.915039 37.0420244 10500

Amblyomma americanum americanum A. americanum -77.915039 37.0420244 10500

Amblyomma americanum americanum A. americanum -77.915039 37.0420244 10500

Amblyomma americanum americanum A. americanum -77.915039 37.0420244 10500

Amblyomma americanum americanum A. americanum -77.915039 37.0420244 10500

Amblyomma americanum americanum A. americanum -77.915039 37.0420244 10500

Amblyomma americanum americanum A. americanum -77.915039 37.0420244 10500

Amblyomma americanum americanum A. americanum -77.915039 37.0420244 10500

Amblyomma americanum americanum A. americanum -77.915039 37.0420244 10500

Amblyomma americanum americanum A. americanum -77.915039 37.0420244 10500

Amblyomma americanum americanum A. americanum -77.915039 37.0420244 10500

Amblyomma americanum americanum A. americanum -77.915039 37.0420244 10500

Amblyomma americanum americanum A. americanum -77.915039 37.0420244 10500

Amblyomma americanum americanum A. americanum -77.915039 37.0420244 10500

Amblyomma americanum americanum A. americanum -77.915039 37.0420244 10500

Amblyomma americanum americanum A. americanum -77.915039 37.0420244 10500

Amblyomma americanum americanum A. americanum -77.915039 37.0420244 10500

Amblyomma americanum americanum A. americanum -77.915039 37.0420244 10500

Amblyomma americanum americanum A. americanum -77.915039 37.0420244 10500

Amblyomma americanum americanum A. americanum -77.915039 37.0420244 10500

Amblyomma americanum americanum A. americanum -77.915039 37.0420244 10500

Amblyomma americanum americanum A. americanum -77.915039 37.0420244 10500

Amblyomma americanum americanum A. americanum -77.915039 37.0420244 10500

Amblyomma americanum americanum A. americanum -77.915039 37.0420244 10500

Amblyomma americanum americanum A. americanum -77.915039 37.0420244 10500

Amblyomma americanum americanum A. americanum -77.915039 37.0420244 10500

Amblyomma americanum americanum A. americanum -77.915039 37.0420244 10500

Amblyomma americanum americanum A. americanum -85.8571243 37.9095336 21000

Amblyomma americanum americanum A. americanum -85.8571243 37.9095336 21000

Amblyomma americanum americanum A. americanum -77.915039 37.0420244 10500

Amblyomma americanum americanum A. americanum -77.915039 37.0420244 10500

Amblyomma americanum americanum A. americanum -77.915039 37.0420244 10500

Amblyomma americanum americanum A. americanum -77.915039 37.0420244 10500

Amblyomma americanum americanum A. americanum -77.915039 37.0420244 10500

Amblyomma americanum americanum A. americanum -77.915039 37.0420244 10500

Amblyomma americanum americanum A. americanum -77.915039 37.0420244 10500

Amblyomma americanum americanum A. americanum -77.915039 37.0420244 10500

Amblyomma americanum americanum A. americanum -77.915039 37.0420244 10500

Amblyomma americanum americanum A. americanum -77.915039 37.0420244 10500

Amblyomma americanum americanum A. americanum -77.915039 37.0420244 10500

Amblyomma americanum americanum A. americanum -77.915039 37.0420244 10500

Amblyomma americanum americanum A. americanum -77.915039 37.0420244 10500

Amblyomma americanum americanum A. americanum -77.915039 37.0420244 10500

Amblyomma americanum americanum A. americanum -77.915039 37.0420244 10500

Amblyomma americanum americanum A. americanum -77.915039 37.0420244 10500

Amblyomma americanum americanum A. americanum -77.915039 37.0420244 10500

Amblyomma americanum americanum A. americanum -77.915039 37.0420244 10500

Amblyomma americanum americanum A. americanum -77.915039 37.0420244 10500

Amblyomma americanum americanum A. americanum -77.915039 37.0420244 10500

Amblyomma americanum americanum A. americanum -77.915039 37.0420244 10500

Amblyomma americanum americanum A. americanum -77.915039 37.0420244 10500

Amblyomma americanum americanum A. americanum -77.915039 37.0420244 10500

Amblyomma americanum americanum A. americanum -77.915039 37.0420244 10500

Amblyomma americanum americanum A. americanum -77.915039 37.0420244 10500

Amblyomma americanum americanum A. americanum -77.915039 37.0420244 10500

Amblyomma americanum americanum A. americanum -77.915039 37.0420244 10500

Amblyomma americanum americanum A. americanum -77.915039 37.0420244 10500

Amblyomma americanum americanum A. americanum -77.915039 37.0420244 10500

Amblyomma americanum americanum A. americanum -77.915039 37.0420244 10500

Amblyomma americanum americanum A. americanum -77.915039 37.0420244 10500

Amblyomma americanum americanum A. americanum -77.915039 37.0420244 10500

Amblyomma americanum americanum A. americanum -77.915039 37.0420244 10500

Amblyomma americanum americanum A. americanum -77.915039 37.0420244 10500

Amblyomma americanum americanum A. americanum -77.915039 37.0420244 10500

Amblyomma americanum americanum A. americanum -77.915039 37.0420244 10500

Amblyomma americanum americanum A. americanum -92.304301 34.914329 12000

Amblyomma americanum americanum A. americanum -85.8571243 37.9095336 21000

Amblyomma americanum americanum A. americanum -85.8571243 37.9095336 21000

Amblyomma americanum americanum A. americanum -78.31089 36.600921 5000

Amblyomma americanum americanum A. americanum -76.1647796 39.4442798 12000

Amblyomma americanum americanum A. americanum -88.555418 32.551715 5000

Amblyomma americanum americanum A. americanum -74.1514316 40.2555169 40840

Amblyomma americanum americanum A. americanum -80.822222 34.039167 12000

Amblyomma americanum americanum A. americanum -80.822222 34.039167 12000

Amblyomma americanum americanum A. americanum -77.915039 37.0420244 10500

Amblyomma americanum americanum A. americanum -77.915039 37.0420244 10500

Amblyomma americanum americanum A. americanum -77.915039 37.0420244 10500

Amblyomma americanum americanum A. americanum -77.915039 37.0420244 10500

Amblyomma americanum americanum A. americanum -77.915039 37.0420244 10500

Amblyomma americanum americanum A. americanum -77.915039 37.0420244 10500

Amblyomma americanum americanum A. americanum -77.915039 37.0420244 10500

Amblyomma americanum americanum A. americanum -77.915039 37.0420244 10500

Amblyomma americanum americanum A. americanum -77.915039 37.0420244 10500

Amblyomma americanum americanum A. americanum -77.915039 37.0420244 10500

Amblyomma americanum americanum A. americanum -77.915039 37.0420244 10500

Amblyomma americanum americanum A. americanum -77.915039 37.0420244 10500

Amblyomma americanum americanum A. americanum -77.915039 37.0420244 10500

Amblyomma americanum americanum A. americanum -77.915039 37.0420244 10500

Amblyomma americanum americanum A. americanum -77.915039 37.0420244 10500

Amblyomma americanum americanum A. americanum -77.915039 37.0420244 10500

Amblyomma americanum americanum A. americanum -77.915039 37.0420244 10500

Amblyomma americanum americanum A. americanum -77.915039 37.0420244 10500

Amblyomma americanum americanum A. americanum -77.915039 37.0420244 10500

Amblyomma americanum americanum A. americanum -77.915039 37.0420244 10500

Amblyomma americanum americanum A. americanum -77.915039 37.0420244 10500

Amblyomma americanum americanum A. americanum -77.915039 37.0420244 10500

Amblyomma americanum americanum A. americanum -77.915039 37.0420244 10500

Amblyomma americanum americanum A. americanum -77.915039 37.0420244 10500

Amblyomma americanum americanum A. americanum -77.915039 37.0420244 10500

Amblyomma americanum americanum A. americanum -77.915039 37.0420244 10500

Amblyomma americanum americanum A. americanum -77.915039 37.0420244 10500

Amblyomma americanum americanum A. americanum -77.915039 37.0420244 10500

Amblyomma americanum americanum A. americanum -77.915039 37.0420244 10500

Amblyomma americanum americanum A. americanum -77.915039 37.0420244 10500

Amblyomma americanum americanum A. americanum -77.915039 37.0420244 10500

Amblyomma americanum americanum A. americanum -77.915039 37.0420244 10500

Amblyomma americanum americanum A. americanum -77.915039 37.0420244 10500

Amblyomma americanum americanum A. americanum -77.915039 37.0420244 10500

Amblyomma americanum americanum A. americanum -77.915039 37.0420244 10500

Amblyomma americanum americanum A. americanum -77.915039 37.0420244 10500

Amblyomma americanum americanum A. americanum -77.915039 37.0420244 10500

Amblyomma americanum americanum A. americanum -77.915039 37.0420244 10500

Amblyomma americanum americanum A. americanum -77.915039 37.0420244 10500

Amblyomma americanum americanum A. americanum -77.915039 37.0420244 10500

Amblyomma americanum americanum A. americanum -77.915039 37.0420244 10500

Amblyomma americanum americanum A. americanum -77.915039 37.0420244 10500

Amblyomma americanum americanum A. americanum -77.915039 37.0420244 10500

Amblyomma americanum americanum A. americanum -77.915039 37.0420244 10500

Amblyomma americanum americanum A. americanum -77.915039 37.0420244 10500

Amblyomma americanum americanum A. americanum -77.915039 37.0420244 10500

Amblyomma americanum americanum A. americanum -77.915039 37.0420244 10500

Amblyomma americanum americanum A. americanum -76.1647796 39.4442798 12000

Amblyomma americanum americanum A. americanum -74.576966 40.024197 8000

Amblyomma americanum americanum A. americanum -77.3341369 37.2359319 4000

Amblyomma americanum americanum A. americanum -76.1647796 39.4442798 12000

Amblyomma americanum americanum A. americanum -76.1647796 39.4442798 12000

Amblyomma americanum americanum A. americanum -87.6159667 36.6078112 18500

Amblyomma americanum americanum A. americanum -80.822222 34.039167 12000

Amblyomma americanum americanum A. americanum -77.1459102 38.7021234 3500

Amblyomma americanum americanum A. americanum -77.1459102 38.7021234 3500

Amblyomma americanum americanum A. americanum -76.1647796 39.4442798 12000

Amblyomma americanum americanum A. americanum -77.3341369 37.2359319 4000

Amblyomma americanum americanum A. americanum -76.1647796 39.4442798 12000

Amblyomma americanum americanum A. americanum -76.1647796 39.4442798 12000

Amblyomma americanum americanum A. americanum -76.1647796 39.4442798 12000

Amblyomma americanum americanum A. americanum -76.1647796 39.4442798 12000

Amblyomma americanum americanum A. americanum -76.1647796 39.4442798 12000

Amblyomma americanum americanum A. americanum -87.6159667 36.6078112 18500

Amblyomma americanum americanum A. americanum -87.6159667 36.6078112 18500

Amblyomma americanum americanum A. americanum -74.576966 40.024197 8000

Amblyomma americanum americanum A. americanum -74.576966 40.024197 8000

Amblyomma americanum americanum A. americanum -74.576966 40.024197 8000

Amblyomma americanum americanum A. americanum -74.576966 40.024197 8000

Amblyomma americanum americanum A. americanum -74.576966 40.024197 8000

Amblyomma americanum americanum A. americanum -74.576966 40.024197 8000

Amblyomma americanum americanum A. americanum -74.576966 40.024197 8000

Amblyomma americanum americanum A. americanum -74.1514316 40.2555169 40840

Amblyomma americanum americanum A. americanum -76.1647796 39.4442798 12000

Amblyomma americanum americanum A. americanum -76.1647796 39.4442798 12000

Amblyomma americanum americanum A. americanum -76.1647796 39.4442798 12000

Amblyomma americanum americanum A. americanum -76.1647796 39.4442798 12000

Amblyomma americanum americanum A. americanum -85.8571243 37.9095336 21000

Amblyomma americanum americanum A. americanum -77.3341369 37.2359319 4000

Amblyomma americanum americanum A. americanum -76.1647796 39.4442798 12000

Amblyomma americanum americanum A. americanum -157.889061 21.360713 1000

Amblyomma americanum americanum A. americanum -76.1647796 39.4442798 12000

Amblyomma americanum americanum A. americanum -80.822222 34.039167 12000

Amblyomma americanum americanum A. americanum -74.370593 40.029739 6000

Amblyomma americanum americanum A. americanum -76.1647796 39.4442798 12000

Amblyomma americanum americanum A. americanum -76.7734909 39.0642483 10000

Amblyomma americanum americanum A. americanum -87.6159667 36.6078112 18500

Amblyomma americanum americanum A. americanum -85.8571243 37.9095336 21000

Amblyomma americanum americanum A. americanum -79.497496 35.03639 6000

Amblyomma americanum americanum A. americanum -76.7734909 39.0642483 10000

Amblyomma americanum americanum A. americanum -92.1572583 37.7057025 16307

Amblyomma americanum americanum A. americanum -75.4650878 39.1272638 3000

Amblyomma americanum americanum A. americanum -76.1647796 39.4442798 12000

Amblyomma americanum americanum A. americanum -76.1647796 39.4442798 12000

Amblyomma americanum americanum A. americanum -77.1459102 38.7021234 3500

Amblyomma americanum americanum A. americanum -76.1647796 39.4442798 12000

Amblyomma americanum americanum A. americanum -76.1647796 39.4442798 12000

Amblyomma americanum americanum A. americanum -76.5877532 37.1534759 9500

Amblyomma americanum americanum A. americanum -74.576966 40.024197 8000

Amblyomma americanum americanum A. americanum -76.7734909 39.0642483 10000

Amblyomma americanum americanum A. americanum -88.555418 32.551715 5000

Amblyomma americanum americanum A. americanum -76.5877532 37.1534759 9500

Amblyomma americanum americanum A. americanum -76.1647796 39.4442798 12000

Amblyomma americanum americanum A. americanum -85.5486378 30.0445485 11033

Amblyomma americanum americanum A. americanum -87.6159667 36.6078112 18500

Amblyomma americanum americanum A. americanum -76.1647796 39.4442798 12000

Amblyomma americanum americanum A. americanum -77.2750854 38.1097082 13000

Amblyomma americanum americanum A. americanum -77.2750854 38.1097082 13000

Amblyomma americanum americanum A. americanum -74.576966 40.024197 8000

Amblyomma americanum americanum A. americanum -76.1647796 39.4442798 12000

Amblyomma americanum americanum A. americanum -76.1647796 39.4442798 12000

Amblyomma americanum americanum A. americanum -76.1647796 39.4442798 12000

Amblyomma americanum americanum A. americanum -76.1647796 39.4442798 12000

Amblyomma americanum americanum A. americanum -85.8571243 37.9095336 21000

Amblyomma americanum americanum A. americanum -77.3341369 37.2359319 4000

Amblyomma americanum americanum A. americanum -76.1647796 39.4442798 12000

Amblyomma americanum americanum A. americanum -77.1459102 38.7021234 3500

Amblyomma americanum americanum A. americanum -76.84515 39.035445 2000

Amblyomma americanum americanum A. americanum -76.1647796 39.4442798 12000

Amblyomma americanum americanum A. americanum -76.1647796 39.4442798 12000

Amblyomma americanum americanum A. americanum -76.1647796 39.4442798 12000

Amblyomma americanum americanum A. americanum -76.1647796 39.4442798 12000

Amblyomma americanum americanum A. americanum -77.3341369 37.2359319 4000

Amblyomma americanum americanum A. americanum -78.507778 38.054167 100

Amblyomma americanum americanum A. americanum -78.507778 38.054167 100

Amblyomma americanum americanum A. americanum -78.507778 38.054167 100

Amblyomma americanum americanum A. americanum -92.1572583 37.7057025 16307

Amblyomma americanum americanum A. americanum -76.1647796 39.4442798 12000

Amblyomma americanum americanum A. americanum -77.3341369 37.2359319 4000

Amblyomma americanum americanum A. americanum -77.3341369 37.2359319 4000

Amblyomma americanum americanum A. americanum -77.3341369 37.2359319 4000

Amblyomma americanum americanum A. americanum -96.8206687 39.1865859 20922

Amblyomma americanum americanum A. americanum -77.1459102 38.7021234 3500

Amblyomma americanum americanum A. americanum -76.1647796 39.4442798 12000

Amblyomma americanum americanum A. americanum -76.1647796 39.4442798 12000

Amblyomma americanum americanum A. americanum -76.1647796 39.4442798 12000

Amblyomma americanum americanum A. americanum -76.1647796 39.4442798 12000

Amblyomma americanum americanum A. americanum -74.576966 40.024197 8000

Amblyomma americanum americanum A. americanum -77.915039 37.0420244 10500

Amblyomma americanum americanum A. americanum -77.1459102 38.7021234 3500

Amblyomma americanum americanum A. americanum -87.6159667 36.6078112 18500

Amblyomma americanum americanum A. americanum -77.3341369 37.2359319 4000

Amblyomma americanum americanum A. americanum -92.1572583 37.7057025 16307

Amblyomma americanum americanum A. americanum -76.1647796 39.4442798 12000

Amblyomma americanum americanum A. americanum -94.9129486 39.3571307 6500

Amblyomma americanum americanum A. americanum -76.1647796 39.4442798 12000

Amblyomma americanum americanum A. americanum -76.1647796 39.4442798 12000

Amblyomma americanum americanum A. americanum -96.8206687 39.1865859 20922

Amblyomma americanum americanum A. americanum -87.6159667 36.6078112 18500

Amblyomma americanum americanum A. americanum -87.6159667 36.6078112 18500

Amblyomma americanum americanum A. americanum -74.1514316 40.2555169 40840

Amblyomma americanum americanum A. americanum -96.8206687 39.1865859 20922

Amblyomma americanum americanum A. americanum -85.5486378 30.0445485 11033

Amblyomma americanum americanum A. americanum -76.1647796 39.4442798 12000

Amblyomma americanum americanum A. americanum -74.576966 40.024197 8000

Amblyomma americanum americanum A. americanum -76.7734909 39.0642483 10000

Amblyomma americanum americanum A. americanum -92.1572583 37.7057025 16307

Amblyomma americanum americanum A. americanum -92.1572583 37.7057025 16307

Amblyomma americanum americanum A. americanum -92.1572583 37.7057025 16307

Amblyomma americanum americanum A. americanum -92.1572583 37.7057025 16307

Amblyomma americanum americanum A. americanum -77.915039 37.0420244 10500

Amblyomma americanum americanum A. americanum -77.915039 37.0420244 10500

Amblyomma americanum americanum A. americanum -77.915039 37.0420244 10500

Amblyomma americanum americanum A. americanum -77.915039 37.0420244 10500

Amblyomma americanum americanum A. americanum -74.576966 40.024197 8000

Amblyomma americanum americanum A. americanum -87.6159667 36.6078112 18500

Amblyomma americanum americanum A. americanum -77.2750854 38.1097082 13000

Amblyomma americanum americanum A. americanum -85.8571243 37.9095336 21000

Amblyomma americanum americanum A. americanum -77.915039 37.0420244 10500

Amblyomma americanum americanum A. americanum -76.1647796 39.4442798 12000

Amblyomma americanum americanum A. americanum -76.1647796 39.4442798 12000

Amblyomma americanum americanum A. americanum -76.1647796 39.4442798 12000

Amblyomma americanum americanum A. americanum -76.1647796 39.4442798 12000

Amblyomma americanum americanum A. americanum -77.1459102 38.7021234 3500

Amblyomma americanum americanum A. americanum -76.1647796 39.4442798 12000

Amblyomma americanum americanum A. americanum -76.1647796 39.4442798 12000

Amblyomma americanum americanum A. americanum -76.1647796 39.4442798 12000

Amblyomma americanum americanum A. americanum -90.6749725 44.0382442 18000

Amblyomma americanum americanum A. americanum -92.1572583 37.7057025 16307

Amblyomma americanum americanum A. americanum -76.1647796 39.4442798 12000

Amblyomma americanum americanum A. americanum -76.1647796 39.4442798 12000

Amblyomma americanum americanum A. americanum -76.1647796 39.4442798 12000

Amblyomma americanum americanum A. americanum -77.915039 37.0420244 10500

Amblyomma americanum americanum A. americanum -79.1970062 35.1109218 17000

Amblyomma americanum americanum A. americanum -87.6159667 36.6078112 18500

Amblyomma americanum americanum A. americanum -74.576966 40.024197 8000

Amblyomma americanum americanum A. americanum -77.3341369 37.2359319 4000

Amblyomma americanum americanum A. americanum -92.1572583 37.7057025 16307

Amblyomma americanum americanum A. americanum -76.1647796 39.4442798 12000

Amblyomma americanum americanum A. americanum -76.1647796 39.4442798 12000

Amblyomma americanum americanum A. americanum -76.1647796 39.4442798 12000

Amblyomma americanum americanum A. americanum -76.1647796 39.4442798 12000

Amblyomma americanum americanum A. americanum -76.1647796 39.4442798 12000

Amblyomma americanum americanum A. americanum -76.1647796 39.4442798 12000

Amblyomma americanum americanum A. americanum -76.1647796 39.4442798 12000

Amblyomma americanum americanum A. americanum -77.1459102 38.7021234 3500

Amblyomma americanum americanum A. americanum -76.1647796 39.4442798 12000

Amblyomma americanum americanum A. americanum -76.1647796 39.4442798 12000

Amblyomma americanum americanum A. americanum -76.1647796 39.4442798 12000

Amblyomma americanum americanum A. americanum -77.1459102 38.7021234 3500

Amblyomma americanum americanum A. americanum -87.6159667 36.6078112 18500

Amblyomma americanum americanum A. americanum -87.6159667 36.6078112 18500

Amblyomma americanum americanum A. americanum -76.5877532 37.1534759 9500

Amblyomma americanum americanum A. americanum -74.576966 40.024197 8000

Amblyomma americanum americanum A. americanum -74.1514316 40.2555169 40840

Amblyomma americanum americanum A. americanum -74.1514316 40.2555169 40840

Amblyomma americanum americanum A. americanum -76.1647796 39.4442798 12000

Amblyomma americanum americanum A. americanum -96.8206687 39.1865859 20922

Amblyomma americanum americanum A. americanum -76.1647796 39.4442798 12000

Amblyomma americanum americanum A. americanum -76.1647796 39.4442798 12000

Amblyomma americanum americanum A. americanum -76.1647796 39.4442798 12000

Amblyomma americanum americanum A. americanum -87.6159667 36.6078112 18500

Amblyomma americanum americanum A. americanum -79.070374 35.654428 2000

Amblyomma americanum americanum A. americanum -85.8571243 37.9095336 21000

Amblyomma americanum americanum A. americanum -94.9129486 39.3571307 6500

Amblyomma americanum americanum A. americanum -94.9129486 39.3571307 6500

Amblyomma americanum americanum A. americanum -74.1514316 40.2555169 40840

Amblyomma americanum americanum A. americanum -74.1514316 40.2555169 40840

Amblyomma americanum americanum A. americanum -92.1572583 37.7057025 16307

Amblyomma americanum americanum A. americanum -76.1647796 39.4442798 12000

Amblyomma americanum americanum A. americanum -76.1647796 39.4442798 12000

Amblyomma americanum americanum A. americanum -76.1647796 39.4442798 12000

Amblyomma americanum americanum A. americanum -76.1647796 39.4442798 12000

Amblyomma americanum americanum A. americanum -77.1459102 38.7021234 3500

Amblyomma americanum americanum A. americanum -96.8206687 39.1865859 20922

Amblyomma americanum americanum A. americanum -96.8206687 39.1865859 20922

Amblyomma americanum americanum A. americanum -76.1647796 39.4442798 12000

Amblyomma americanum americanum A. americanum -76.1647796 39.4442798 12000

Amblyomma americanum americanum A. americanum -76.1647796 39.4442798 12000

Amblyomma americanum americanum A. americanum -77.915039 37.0420244 10500

Amblyomma americanum americanum A. americanum -77.915039 37.0420244 10500

Amblyomma americanum americanum A. americanum -77.915039 37.0420244 10500

Amblyomma americanum americanum A. americanum -77.915039 37.0420244 10500

Amblyomma americanum americanum A. americanum -77.915039 37.0420244 10500

Amblyomma americanum americanum A. americanum -77.915039 37.0420244 10500

Amblyomma americanum americanum A. americanum -77.915039 37.0420244 10500

Amblyomma americanum americanum A. americanum -77.915039 37.0420244 10500

Amblyomma americanum americanum A. americanum -77.915039 37.0420244 10500

Amblyomma americanum americanum A. americanum -77.915039 37.0420244 10500

Amblyomma americanum americanum A. americanum -77.915039 37.0420244 10500

Amblyomma americanum americanum A. americanum -77.915039 37.0420244 10500

Amblyomma americanum americanum A. americanum -77.915039 37.0420244 10500

Amblyomma americanum americanum A. americanum -77.915039 37.0420244 10500

Amblyomma americanum americanum A. americanum -77.915039 37.0420244 10500

Amblyomma americanum americanum A. americanum -77.915039 37.0420244 10500

Amblyomma americanum americanum A. americanum -77.915039 37.0420244 10500

Amblyomma americanum americanum A. americanum -77.915039 37.0420244 10500

Amblyomma americanum americanum A. americanum -77.915039 37.0420244 10500

Amblyomma americanum americanum A. americanum -77.915039 37.0420244 10500

Amblyomma americanum americanum A. americanum -77.915039 37.0420244 10500

Amblyomma americanum americanum A. americanum -77.915039 37.0420244 10500

Amblyomma americanum americanum A. americanum -77.915039 37.0420244 10500

Amblyomma americanum americanum A. americanum -77.915039 37.0420244 10500

Amblyomma americanum americanum A. americanum -77.915039 37.0420244 10500

Amblyomma americanum americanum A. americanum -77.915039 37.0420244 10500

Amblyomma americanum americanum A. americanum -77.915039 37.0420244 10500

Amblyomma americanum americanum A. americanum -77.915039 37.0420244 10500

Amblyomma americanum americanum A. americanum -77.915039 37.0420244 10500

Amblyomma americanum americanum A. americanum -77.915039 37.0420244 10500

Amblyomma americanum americanum A. americanum -92.1572583 37.7057025 16307

Amblyomma americanum americanum A. americanum -76.1647796 39.4442798 12000

Amblyomma americanum americanum A. americanum -87.6159667 36.6078112 18500

Amblyomma americanum americanum A. americanum -87.6159667 36.6078112 18500

Amblyomma americanum americanum A. americanum -87.6159667 36.6078112 18500

Amblyomma americanum americanum A. americanum -85.8571243 37.9095336 21000

Amblyomma americanum americanum A. americanum -85.8571243 37.9095336 21000

Amblyomma americanum americanum A. americanum -85.8571243 37.9095336 21000

Amblyomma americanum americanum A. americanum -85.8571243 37.9095336 21000

Amblyomma americanum americanum A. americanum -74.1514316 40.2555169 40840

Amblyomma americanum americanum A. americanum -76.1647796 39.4442798 12000

Amblyomma americanum americanum A. americanum -76.1647796 39.4442798 12000

Amblyomma americanum americanum A. americanum -77.1459102 38.7021234 3500

Amblyomma americanum americanum A. americanum -76.1647796 39.4442798 12000

Amblyomma americanum americanum A. americanum -76.1647796 39.4442798 12000

Amblyomma americanum americanum A. americanum -76.1647796 39.4442798 12000

Amblyomma americanum americanum A. americanum -76.1647796 39.4442798 12000

Amblyomma americanum americanum A. americanum -76.1647796 39.4442798 12000

Amblyomma americanum americanum A. americanum -76.1647796 39.4442798 12000

Amblyomma americanum americanum A. americanum -76.1647796 39.4442798 12000

Amblyomma americanum americanum A. americanum -87.6159667 36.6078112 18500

Amblyomma americanum americanum A. americanum -76.7734909 39.0642483 10000

Amblyomma americanum americanum A. americanum -76.7734909 39.0642483 10000

Amblyomma americanum americanum A. americanum -76.7734909 39.0642483 10000

Amblyomma americanum americanum A. americanum -76.1647796 39.4442798 12000

Amblyomma americanum americanum A. americanum -76.1647796 39.4442798 12000

Amblyomma americanum americanum A. americanum -74.1514316 40.2555169 40840

Amblyomma americanum americanum A. americanum -74.1514316 40.2555169 40840

Amblyomma americanum americanum A. americanum -76.84515 39.035445 2000

Amblyomma americanum americanum A. americanum -76.1647796 39.4442798 12000

Amblyomma americanum americanum A. americanum -76.1647796 39.4442798 12000

Amblyomma americanum americanum A. americanum -76.1647796 39.4442798 12000

Amblyomma americanum americanum A. americanum -76.1647796 39.4442798 12000

Amblyomma americanum americanum A. americanum -76.1647796 39.4442798 12000

Amblyomma americanum americanum A. americanum -80.027761 36.774436 5000

Amblyomma americanum americanum A. americanum -80.027761 36.774436 5000

Amblyomma americanum americanum A. americanum -80.027761 36.774436 5000

Amblyomma americanum americanum A. americanum -87.6159667 36.6078112 18500

Amblyomma americanum americanum A. americanum -77.169167 38.588333 6000

Amblyomma americanum americanum A. americanum -77.3341369 37.2359319 4000

Amblyomma americanum americanum A. americanum -74.1514316 40.2555169 40840

Amblyomma americanum americanum A. americanum -74.1514316 40.2555169 40840

Amblyomma americanum americanum A. americanum -74.1514316 40.2555169 40840

Amblyomma americanum americanum A. americanum -74.1514316 40.2555169 40840

Amblyomma americanum americanum A. americanum -76.84515 39.035445 2000

Amblyomma americanum americanum A. americanum -76.1647796 39.4442798 12000

Amblyomma americanum americanum A. americanum -76.1647796 39.4442798 12000

Amblyomma americanum americanum A. americanum -77.0184516 38.8393064 3500

Amblyomma americanum americanum A. americanum -74.1514316 40.2555169 40840

Amblyomma americanum americanum A. americanum -76.1647796 39.4442798 12000

Amblyomma americanum americanum A. americanum -76.1647796 39.4442798 12000

Amblyomma americanum americanum A. americanum -76.1647796 39.4442798 12000

Amblyomma americanum americanum A. americanum -77.2750854 38.1097082 13000

Amblyomma americanum americanum A. americanum -77.2750854 38.1097082 13000

Amblyomma americanum americanum A. americanum -77.2750854 38.1097082 13000

Amblyomma americanum americanum A. americanum -77.2750854 38.1097082 13000

Amblyomma americanum americanum A. americanum -77.2750854 38.1097082 13000

Amblyomma americanum americanum A. americanum -77.2750854 38.1097082 13000

Amblyomma americanum americanum A. americanum -77.2750854 38.1097082 13000

Amblyomma americanum americanum A. americanum -77.2750854 38.1097082 13000

Amblyomma americanum americanum A. americanum -77.2750854 38.1097082 13000

Amblyomma americanum americanum A. americanum -77.2750854 38.1097082 13000

Amblyomma americanum americanum A. americanum -77.2750854 38.1097082 13000

Amblyomma americanum americanum A. americanum -77.2750854 38.1097082 13000

Amblyomma americanum americanum A. americanum -77.2750854 38.1097082 13000

Amblyomma americanum americanum A. americanum -77.2750854 38.1097082 13000

Amblyomma americanum americanum A. americanum -77.2750854 38.1097082 13000

Amblyomma americanum americanum A. americanum -77.2750854 38.1097082 13000

Amblyomma americanum americanum A. americanum -77.2750854 38.1097082 13000

Amblyomma americanum americanum A. americanum -77.2750854 38.1097082 13000

Amblyomma americanum americanum A. americanum -77.2750854 38.1097082 13000

Amblyomma americanum americanum A. americanum -77.2750854 38.1097082 13000

Amblyomma americanum americanum A. americanum -77.2750854 38.1097082 13000

Amblyomma americanum americanum A. americanum -77.2750854 38.1097082 13000

Amblyomma americanum americanum A. americanum -77.2750854 38.1097082 13000

Amblyomma americanum americanum A. americanum -94.9129486 39.3571307 6500

Amblyomma americanum americanum A. americanum -74.1514316 40.2555169 40840

Amblyomma americanum americanum A. americanum -77.1459102 38.7021234 3500

Amblyomma americanum americanum A. americanum -77.2750854 38.1097082 13000

Amblyomma americanum americanum A. americanum -76.1647796 39.4442798 12000

Amblyomma americanum americanum A. americanum -76.1647796 39.4442798 12000

Amblyomma americanum americanum A. americanum -76.1647796 39.4442798 12000

Amblyomma americanum americanum A. americanum -76.1647796 39.4442798 12000

Amblyomma americanum americanum A. americanum -76.1647796 39.4442798 12000

Amblyomma americanum americanum A. americanum -80.719539 34.010264 3000

Amblyomma americanum americanum A. americanum -76.1647796 39.4442798 12000

Amblyomma americanum americanum A. americanum -76.1647796 39.4442798 12000

Amblyomma americanum americanum A. americanum -76.1647796 39.4442798 12000

Amblyomma americanum americanum A. americanum -87.6159667 36.6078112 18500

Amblyomma americanum americanum A. americanum -87.6159667 36.6078112 18500

Amblyomma americanum americanum A. americanum -76.5877532 37.1534759 9500

Amblyomma americanum americanum A. americanum -74.576966 40.024197 8000

Amblyomma americanum americanum A. americanum -74.576966 40.024197 8000

Amblyomma americanum americanum A. americanum -76.7734909 39.0642483 10000

Amblyomma americanum americanum A. americanum -77.0807647 38.8794077 2000

Amblyomma americanum americanum A. americanum -92.304301 34.914329 12000

Amblyomma americanum americanum A. americanum -76.1647796 39.4442798 12000

Amblyomma americanum americanum A. americanum -76.1647796 39.4442798 12000

Amblyomma americanum americanum A. americanum -76.1647796 39.4442798 12000

Amblyomma americanum americanum A. americanum -76.1647796 39.4442798 12000

Amblyomma americanum americanum A. americanum -76.1647796 39.4442798 12000

Amblyomma americanum americanum A. americanum -74.1514316 40.2555169 40840

Amblyomma americanum americanum A. americanum -76.1647796 39.4442798 12000

Amblyomma americanum americanum A. americanum -76.1647796 39.4442798 12000

Amblyomma americanum americanum A. americanum -77.1459102 38.7021234 3500

Amblyomma americanum americanum A. americanum -77.1459102 38.7021234 3500

Amblyomma americanum americanum A. americanum -87.6159667 36.6078112 18500

Amblyomma americanum americanum A. americanum -87.6159667 36.6078112 18500

Amblyomma americanum americanum A. americanum -76.1647796 39.4442798 12000

Amblyomma americanum americanum A. americanum -76.1647796 39.4442798 12000

Amblyomma americanum americanum A. americanum -76.1647796 39.4442798 12000

Amblyomma americanum americanum A. americanum -76.1647796 39.4442798 12000

Amblyomma americanum americanum A. americanum -76.1647796 39.4442798 12000

Amblyomma americanum americanum A. americanum -76.1647796 39.4442798 12000

Amblyomma americanum americanum A. americanum -76.1647796 39.4442798 12000

Amblyomma americanum americanum A. americanum -87.6159667 36.6078112 18500

Amblyomma americanum americanum A. americanum -87.6159667 36.6078112 18500

Amblyomma americanum americanum A. americanum -87.6159667 36.6078112 18500

Amblyomma americanum americanum A. americanum -87.6159667 36.6078112 18500

Amblyomma americanum americanum A. americanum -80.719539 34.010264 3000

Amblyomma americanum americanum A. americanum -77.1459102 38.7021234 3500

Amblyomma americanum americanum A. americanum -74.576966 40.024197 8000

Amblyomma americanum americanum A. americanum -74.576966 40.024197 8000

Amblyomma americanum americanum A. americanum -74.1514316 40.2555169 40840

Amblyomma americanum americanum A. americanum -74.1514316 40.2555169 40840

Amblyomma americanum americanum A. americanum -76.1647796 39.4442798 12000

Amblyomma americanum americanum A. americanum -77.1459102 38.7021234 3500

Amblyomma americanum americanum A. americanum -79.1970062 35.1109218 17000

Amblyomma americanum americanum A. americanum -77.2750854 38.1097082 13000

Amblyomma americanum americanum A. americanum -85.8571243 37.9095336 21000

Amblyomma americanum americanum A. americanum -76.1647796 39.4442798 12000

Amblyomma americanum americanum A. americanum -76.1647796 39.4442798 12000

Amblyomma americanum americanum A. americanum -76.1647796 39.4442798 12000

Amblyomma americanum americanum A. americanum -76.1647796 39.4442798 12000

Amblyomma americanum americanum A. americanum -77.1459102 38.7021234 3500

Amblyomma americanum americanum A. americanum -86.5295486 30.572647 44515

Amblyomma americanum americanum A. americanum -76.84515 39.035445 2000

Amblyomma americanum americanum A. americanum -76.1647796 39.4442798 12000

Amblyomma americanum americanum A. americanum -76.1647796 39.4442798 12000

Amblyomma americanum americanum A. americanum -76.1647796 39.4442798 12000

Amblyomma americanum americanum A. americanum -80.822222 34.039167 12000

Amblyomma americanum americanum A. americanum -76.7734909 39.0642483 10000

Amblyomma americanum americanum A. americanum -76.7734909 39.0642483 10000

Amblyomma americanum americanum A. americanum -76.7734909 39.0642483 10000

Amblyomma americanum americanum A. americanum -76.7734909 39.0642483 10000

Amblyomma americanum americanum A. americanum -74.1514316 40.2555169 40840

Amblyomma americanum americanum A. americanum -74.1514316 40.2555169 40840

Amblyomma americanum americanum A. americanum -74.1514316 40.2555169 40840

Amblyomma americanum americanum A. americanum -76.1647796 39.4442798 12000

Amblyomma americanum americanum A. americanum -76.1647796 39.4442798 12000

Amblyomma americanum americanum A. americanum -76.1647796 39.4442798 12000

Amblyomma americanum americanum A. americanum -77.915039 37.0420244 10500

Amblyomma americanum americanum A. americanum -76.1647796 39.4442798 12000

Amblyomma americanum americanum A. americanum -76.1647796 39.4442798 12000

Amblyomma americanum americanum A. americanum -76.1647796 39.4442798 12000

Amblyomma americanum americanum A. americanum -77.1459102 38.7021234 3500

Amblyomma americanum americanum A. americanum -77.2750854 38.1097082 13000

Amblyomma americanum americanum A. americanum -77.2750854 38.1097082 13000

Amblyomma americanum americanum A. americanum -77.2750854 38.1097082 13000

Amblyomma americanum americanum A. americanum -77.2750854 38.1097082 13000

Amblyomma americanum americanum A. americanum -77.2750854 38.1097082 13000

Amblyomma americanum americanum A. americanum -77.2750854 38.1097082 13000

Amblyomma americanum americanum A. americanum -77.2750854 38.1097082 13000

Amblyomma americanum americanum A. americanum -77.2750854 38.1097082 13000

Amblyomma americanum americanum A. americanum -77.2750854 38.1097082 13000

Amblyomma americanum americanum A. americanum -77.2750854 38.1097082 13000

Amblyomma americanum americanum A. americanum -77.2750854 38.1097082 13000

Amblyomma americanum americanum A. americanum -77.2750854 38.1097082 13000

Amblyomma americanum americanum A. americanum -77.2750854 38.1097082 13000

Amblyomma americanum americanum A. americanum -77.2750854 38.1097082 13000

Amblyomma americanum americanum A. americanum -77.2750854 38.1097082 13000

Amblyomma americanum americanum A. americanum -77.2750854 38.1097082 13000

Amblyomma americanum americanum A. americanum -77.2750854 38.1097082 13000

Amblyomma americanum americanum A. americanum -77.2750854 38.1097082 13000

Amblyomma americanum americanum A. americanum -77.2750854 38.1097082 13000

Amblyomma americanum americanum A. americanum -77.2750854 38.1097082 13000

Amblyomma americanum americanum A. americanum -77.2750854 38.1097082 13000

Amblyomma americanum americanum A. americanum -77.2750854 38.1097082 13000

Amblyomma americanum americanum A. americanum -77.2750854 38.1097082 13000

Amblyomma americanum americanum A. americanum -77.2750854 38.1097082 13000

Amblyomma americanum americanum A. americanum -77.2750854 38.1097082 13000

Amblyomma americanum americanum A. americanum -77.2750854 38.1097082 13000

Amblyomma americanum americanum A. americanum -77.2750854 38.1097082 13000

Amblyomma americanum americanum A. americanum -77.2750854 38.1097082 13000

Amblyomma americanum americanum A. americanum -77.2750854 38.1097082 13000

Amblyomma americanum americanum A. americanum -77.2750854 38.1097082 13000

Amblyomma americanum americanum A. americanum -77.2750854 38.1097082 13000

Amblyomma americanum americanum A. americanum -77.2750854 38.1097082 13000

Amblyomma americanum americanum A. americanum -77.2750854 38.1097082 13000

Amblyomma americanum americanum A. americanum -77.2750854 38.1097082 13000

Amblyomma americanum americanum A. americanum -77.2750854 38.1097082 13000

Amblyomma americanum americanum A. americanum -77.2750854 38.1097082 13000

Amblyomma americanum americanum A. americanum -77.2750854 38.1097082 13000

Amblyomma americanum americanum A. americanum -77.2750854 38.1097082 13000

Amblyomma americanum americanum A. americanum -77.2750854 38.1097082 13000

Amblyomma americanum americanum A. americanum -77.2750854 38.1097082 13000

Amblyomma americanum americanum A. americanum -76.1647796 39.4442798 12000

Amblyomma americanum americanum A. americanum -75.4650878 39.1272638 3000

Amblyomma americanum americanum A. americanum -85.8571243 37.9095336 21000

Amblyomma americanum americanum A. americanum -85.8571243 37.9095336 21000

Amblyomma americanum americanum A. americanum -77.915039 37.0420244 10500

Amblyomma americanum americanum A. americanum -77.915039 37.0420244 10500

Amblyomma americanum americanum A. americanum -77.915039 37.0420244 10500

Amblyomma americanum americanum A. americanum -77.915039 37.0420244 10500

Amblyomma americanum americanum A. americanum -77.915039 37.0420244 10500

Amblyomma americanum americanum A. americanum -77.915039 37.0420244 10500

Amblyomma americanum americanum A. americanum -77.915039 37.0420244 10500

Amblyomma americanum americanum A. americanum -77.915039 37.0420244 10500

Amblyomma americanum americanum A. americanum -77.915039 37.0420244 10500

Amblyomma americanum americanum A. americanum -77.915039 37.0420244 10500

Amblyomma americanum americanum A. americanum -77.915039 37.0420244 10500

Amblyomma americanum americanum A. americanum -77.915039 37.0420244 10500

Amblyomma americanum americanum A. americanum -77.915039 37.0420244 10500

Amblyomma americanum americanum A. americanum -77.915039 37.0420244 10500

Amblyomma americanum americanum A. americanum -77.915039 37.0420244 10500

Amblyomma americanum americanum A. americanum -77.915039 37.0420244 10500

Amblyomma americanum americanum A. americanum -77.915039 37.0420244 10500

Amblyomma americanum americanum A. americanum -77.915039 37.0420244 10500

Amblyomma americanum americanum A. americanum -77.915039 37.0420244 10500

Amblyomma americanum americanum A. americanum -77.915039 37.0420244 10500

Amblyomma americanum americanum A. americanum -77.915039 37.0420244 10500

Amblyomma americanum americanum A. americanum -77.915039 37.0420244 10500

Amblyomma americanum americanum A. americanum -77.915039 37.0420244 10500

Amblyomma americanum americanum A. americanum -77.915039 37.0420244 10500

Amblyomma americanum americanum A. americanum -77.915039 37.0420244 10500

Amblyomma americanum americanum A. americanum -77.915039 37.0420244 10500

Amblyomma americanum americanum A. americanum -77.915039 37.0420244 10500

Amblyomma americanum americanum A. americanum -77.915039 37.0420244 10500

Amblyomma americanum americanum A. americanum -77.915039 37.0420244 10500

Amblyomma americanum americanum A. americanum -77.915039 37.0420244 10500

Amblyomma americanum americanum A. americanum -77.915039 37.0420244 10500

Amblyomma americanum americanum A. americanum -77.915039 37.0420244 10500

Amblyomma americanum americanum A. americanum -77.915039 37.0420244 10500

Amblyomma americanum americanum A. americanum -77.915039 37.0420244 10500

Amblyomma americanum americanum A. americanum -77.915039 37.0420244 10500

Amblyomma americanum americanum A. americanum -77.915039 37.0420244 10500

Amblyomma americanum americanum A. americanum -77.915039 37.0420244 10500

Amblyomma americanum americanum A. americanum -77.915039 37.0420244 10500

Amblyomma americanum americanum A. americanum -77.915039 37.0420244 10500

Amblyomma americanum americanum A. americanum -77.915039 37.0420244 10500

Amblyomma americanum americanum A. americanum -77.915039 37.0420244 10500

Amblyomma americanum americanum A. americanum -77.915039 37.0420244 10500

Amblyomma americanum americanum A. americanum -77.915039 37.0420244 10500

Amblyomma americanum americanum A. americanum -77.915039 37.0420244 10500

Amblyomma americanum americanum A. americanum -77.915039 37.0420244 10500

Amblyomma americanum americanum A. americanum -77.915039 37.0420244 10500

Amblyomma americanum americanum A. americanum -77.915039 37.0420244 10500

Amblyomma americanum americanum A. americanum -77.915039 37.0420244 10500

Amblyomma americanum americanum A. americanum -77.915039 37.0420244 10500

Amblyomma americanum americanum A. americanum -77.915039 37.0420244 10500

Amblyomma americanum americanum A. americanum -76.1647796 39.4442798 12000

Amblyomma americanum americanum A. americanum -76.1647796 39.4442798 12000

Amblyomma americanum americanum A. americanum -76.1647796 39.4442798 12000

Amblyomma americanum americanum A. americanum -76.1647796 39.4442798 12000

Amblyomma americanum americanum A. americanum -76.1647796 39.4442798 12000

Amblyomma americanum americanum A. americanum -76.1647796 39.4442798 12000

Amblyomma americanum americanum A. americanum -87.6159667 36.6078112 18500

Amblyomma americanum americanum A. americanum -85.8571243 37.9095336 21000

Amblyomma americanum americanum A. americanum -94.9129486 39.3571307 6500

Amblyomma americanum americanum A. americanum -76.1647796 39.4442798 12000

Amblyomma americanum americanum A. americanum -76.1647796 39.4442798 12000

Amblyomma americanum americanum A. americanum -76.1647796 39.4442798 12000

Amblyomma americanum americanum A. americanum -76.1647796 39.4442798 12000

Amblyomma americanum americanum A. americanum -77.1459102 38.7021234 3500

Amblyomma americanum americanum A. americanum -87.6159667 36.6078112 18500

Amblyomma americanum americanum A. americanum -76.5877532 37.1534759 9500

Amblyomma americanum americanum A. americanum -74.1514316 40.2555169 40840

Amblyomma americanum americanum A. americanum -74.1514316 40.2555169 40840

Amblyomma americanum americanum A. americanum -74.1514316 40.2555169 40840

Amblyomma americanum americanum A. americanum -74.1514316 40.2555169 40840

Amblyomma americanum americanum A. americanum -74.1514316 40.2555169 40840

Amblyomma americanum americanum A. americanum -96.8206687 39.1865859 20922

Amblyomma americanum americanum A. americanum -76.1647796 39.4442798 12000

Amblyomma americanum americanum A. americanum -76.1647796 39.4442798 12000

Amblyomma americanum americanum A. americanum -76.1647796 39.4442798 12000

Amblyomma americanum americanum A. americanum -76.1647796 39.4442798 12000

Amblyomma americanum americanum A. americanum -77.3341369 37.2359319 4000

Amblyomma americanum americanum A. americanum -76.7734909 39.0642483 10000

Amblyomma americanum americanum A. americanum -74.576966 40.024197 8000

Amblyomma americanum americanum A. americanum -74.576966 40.024197 8000

Amblyomma americanum americanum A. americanum -94.9129486 39.3571307 6500

Amblyomma americanum americanum A. americanum -77.915039 37.0420244 10500

Amblyomma americanum americanum A. americanum -76.1647796 39.4442798 12000

Amblyomma americanum americanum A. americanum -76.1647796 39.4442798 12000

Amblyomma americanum americanum A. americanum -76.5877532 37.1534759 9500

Amblyomma americanum americanum A. americanum -77.915039 37.0420244 10500

Amblyomma americanum americanum A. americanum -77.915039 37.0420244 10500

Amblyomma americanum americanum A. americanum -77.915039 37.0420244 10500

Amblyomma americanum americanum A. americanum -77.915039 37.0420244 10500

Amblyomma americanum americanum A. americanum -77.915039 37.0420244 10500

Amblyomma americanum americanum A. americanum -77.915039 37.0420244 10500

Amblyomma americanum americanum A. americanum -77.915039 37.0420244 10500

Amblyomma americanum americanum A. americanum -77.915039 37.0420244 10500

Amblyomma americanum americanum A. americanum -77.915039 37.0420244 10500

Amblyomma americanum americanum A. americanum -77.915039 37.0420244 10500

Amblyomma americanum americanum A. americanum -77.915039 37.0420244 10500

Amblyomma americanum americanum A. americanum -77.915039 37.0420244 10500

Amblyomma americanum americanum A. americanum -77.915039 37.0420244 10500

Amblyomma americanum americanum A. americanum -77.915039 37.0420244 10500

Amblyomma americanum americanum A. americanum -77.915039 37.0420244 10500

Amblyomma americanum americanum A. americanum -77.915039 37.0420244 10500

Amblyomma americanum americanum A. americanum -77.915039 37.0420244 10500

Amblyomma americanum americanum A. americanum -77.915039 37.0420244 10500

Amblyomma americanum americanum A. americanum -77.915039 37.0420244 10500

Amblyomma americanum americanum A. americanum -77.915039 37.0420244 10500

Amblyomma americanum americanum A. americanum -77.915039 37.0420244 10500

Amblyomma americanum americanum A. americanum -77.915039 37.0420244 10500

Amblyomma americanum americanum A. americanum -77.915039 37.0420244 10500

Amblyomma americanum americanum A. americanum -77.915039 37.0420244 10500

Amblyomma americanum americanum A. americanum -77.915039 37.0420244 10500

Amblyomma americanum americanum A. americanum -77.915039 37.0420244 10500

Amblyomma americanum americanum A. americanum -77.915039 37.0420244 10500

Amblyomma americanum americanum A. americanum -77.915039 37.0420244 10500

Amblyomma americanum americanum A. americanum -77.915039 37.0420244 10500

Amblyomma americanum americanum A. americanum -77.915039 37.0420244 10500

Amblyomma americanum americanum A. americanum -77.915039 37.0420244 10500

Amblyomma americanum americanum A. americanum -77.915039 37.0420244 10500

Amblyomma americanum americanum A. americanum -77.915039 37.0420244 10500

Amblyomma americanum americanum A. americanum -77.1459102 38.7021234 3500

Amblyomma americanum americanum A. americanum -79.1970062 35.1109218 17000

Amblyomma americanum americanum A. americanum -85.8571243 37.9095336 21000

Amblyomma americanum americanum A. americanum -85.8571243 37.9095336 21000

Amblyomma americanum americanum A. americanum -85.8571243 37.9095336 21000

Amblyomma americanum americanum A. americanum -85.8571243 37.9095336 21000

Amblyomma americanum americanum A. americanum -85.8571243 37.9095336 21000

Amblyomma americanum americanum A. americanum -85.8571243 37.9095336 21000

Amblyomma americanum americanum A. americanum -76.1647796 39.4442798 12000

Amblyomma americanum americanum A. americanum -77.0184516 38.8393064 3500

Amblyomma americanum americanum A. americanum -75.4650878 39.1272638 3000

Amblyomma americanum americanum A. americanum -74.1514316 40.2555169 40840

Amblyomma americanum americanum A. americanum -96.8206687 39.1865859 20922

Amblyomma americanum americanum A. americanum -76.1647796 39.4442798 12000

Amblyomma americanum americanum A. americanum -76.1647796 39.4442798 12000

Amblyomma americanum americanum A. americanum -76.1647796 39.4442798 12000

Amblyomma americanum americanum A. americanum -76.1647796 39.4442798 12000

Amblyomma americanum americanum A. americanum -87.6159667 36.6078112 18500

Amblyomma americanum americanum A. americanum -75.4650878 39.1272638 3000

Amblyomma americanum americanum A. americanum -77.3341369 37.2359319 4000

Amblyomma americanum americanum A. americanum -77.3341369 37.2359319 4000

Amblyomma americanum americanum A. americanum -76.7734909 39.0642483 10000

Amblyomma americanum americanum A. americanum -77.915039 37.0420244 10500

Amblyomma americanum americanum A. americanum -77.915039 37.0420244 10500

Amblyomma americanum americanum A. americanum -77.915039 37.0420244 10500

Amblyomma americanum americanum A. americanum -77.915039 37.0420244 10500

Amblyomma americanum americanum A. americanum -77.915039 37.0420244 10500

Amblyomma americanum americanum A. americanum -77.915039 37.0420244 10500

Amblyomma americanum americanum A. americanum -77.915039 37.0420244 10500

Amblyomma americanum americanum A. americanum -77.915039 37.0420244 10500

Amblyomma americanum americanum A. americanum -77.915039 37.0420244 10500

Amblyomma americanum americanum A. americanum -77.915039 37.0420244 10500

Amblyomma americanum americanum A. americanum -77.915039 37.0420244 10500

Amblyomma americanum americanum A. americanum -77.915039 37.0420244 10500

Amblyomma americanum americanum A. americanum -77.915039 37.0420244 10500

Amblyomma americanum americanum A. americanum -77.915039 37.0420244 10500

Amblyomma americanum americanum A. americanum -77.915039 37.0420244 10500

Amblyomma americanum americanum A. americanum -77.915039 37.0420244 10500

Amblyomma americanum americanum A. americanum -77.915039 37.0420244 10500

Amblyomma americanum americanum A. americanum -77.915039 37.0420244 10500

Amblyomma americanum americanum A. americanum -77.915039 37.0420244 10500

Amblyomma americanum americanum A. americanum -77.915039 37.0420244 10500

Amblyomma americanum americanum A. americanum -77.915039 37.0420244 10500

Amblyomma americanum americanum A. americanum -77.915039 37.0420244 10500

Amblyomma americanum americanum A. americanum -77.915039 37.0420244 10500

Amblyomma americanum americanum A. americanum -77.915039 37.0420244 10500

Amblyomma americanum americanum A. americanum -77.915039 37.0420244 10500

Amblyomma americanum americanum A. americanum -77.915039 37.0420244 10500

Amblyomma americanum americanum A. americanum -77.915039 37.0420244 10500

Amblyomma americanum americanum A. americanum -77.915039 37.0420244 10500

Amblyomma americanum americanum A. americanum -77.915039 37.0420244 10500

Amblyomma americanum americanum A. americanum -77.915039 37.0420244 10500

Amblyomma americanum americanum A. americanum -77.915039 37.0420244 10500

Amblyomma americanum americanum A. americanum -77.915039 37.0420244 10500

Amblyomma americanum americanum A. americanum -77.915039 37.0420244 10500

Amblyomma americanum americanum A. americanum -77.915039 37.0420244 10500

Amblyomma americanum americanum A. americanum -77.915039 37.0420244 10500

Amblyomma americanum americanum A. americanum -77.915039 37.0420244 10500

Amblyomma americanum americanum A. americanum -77.915039 37.0420244 10500

Amblyomma americanum americanum A. americanum -77.915039 37.0420244 10500

Amblyomma americanum americanum A. americanum -77.915039 37.0420244 10500

Amblyomma americanum americanum A. americanum -77.915039 37.0420244 10500

Amblyomma americanum americanum A. americanum -77.915039 37.0420244 10500

Amblyomma americanum americanum A. americanum -77.915039 37.0420244 10500

Amblyomma americanum americanum A. americanum -77.915039 37.0420244 10500

Amblyomma americanum americanum A. americanum -77.915039 37.0420244 10500

Amblyomma americanum americanum A. americanum -77.915039 37.0420244 10500

Amblyomma americanum americanum A. americanum -77.915039 37.0420244 10500

Amblyomma americanum americanum A. americanum -77.915039 37.0420244 10500

Amblyomma americanum americanum A. americanum -77.915039 37.0420244 10500

Amblyomma americanum americanum A. americanum -77.915039 37.0420244 10500

Amblyomma americanum americanum A. americanum -77.915039 37.0420244 10500

Amblyomma americanum americanum A. americanum -77.915039 37.0420244 10500

Amblyomma americanum americanum A. americanum -77.915039 37.0420244 10500

Amblyomma americanum americanum A. americanum -77.915039 37.0420244 10500

Amblyomma americanum americanum A. americanum -77.915039 37.0420244 10500

Amblyomma americanum americanum A. americanum -76.1647796 39.4442798 12000

Amblyomma americanum americanum A. americanum -76.1647796 39.4442798 12000

Amblyomma americanum americanum A. americanum -76.1647796 39.4442798 12000

Amblyomma americanum americanum A. americanum -76.1647796 39.4442798 12000

Amblyomma americanum americanum A. americanum -87.6159667 36.6078112 18500

Amblyomma americanum americanum A. americanum -87.6159667 36.6078112 18500

Amblyomma americanum americanum A. americanum -85.8571243 37.9095336 21000

Amblyomma americanum americanum A. americanum -77.915039 37.0420244 10500

Amblyomma americanum americanum A. americanum -77.915039 37.0420244 10500

Amblyomma americanum americanum A. americanum -77.915039 37.0420244 10500

Amblyomma americanum americanum A. americanum -77.915039 37.0420244 10500

Amblyomma americanum americanum A. americanum -77.915039 37.0420244 10500

Amblyomma americanum americanum A. americanum -77.915039 37.0420244 10500

Amblyomma americanum americanum A. americanum -77.915039 37.0420244 10500

Amblyomma americanum americanum A. americanum -77.915039 37.0420244 10500

Amblyomma americanum americanum A. americanum -77.915039 37.0420244 10500

Amblyomma americanum americanum A. americanum -77.915039 37.0420244 10500

Amblyomma americanum americanum A. americanum -77.915039 37.0420244 10500

Amblyomma americanum americanum A. americanum -77.915039 37.0420244 10500

Amblyomma americanum americanum A. americanum -77.915039 37.0420244 10500

Amblyomma americanum americanum A. americanum -77.915039 37.0420244 10500

Amblyomma americanum americanum A. americanum -77.915039 37.0420244 10500

Amblyomma americanum americanum A. americanum -77.915039 37.0420244 10500

Amblyomma americanum americanum A. americanum -77.915039 37.0420244 10500

Amblyomma americanum americanum A. americanum -77.915039 37.0420244 10500

Amblyomma americanum americanum A. americanum -90.6749725 44.0382442 18000

Amblyomma americanum americanum A. americanum -86.5295486 30.572647 44515

Amblyomma americanum americanum A. americanum -76.1647796 39.4442798 12000

Amblyomma americanum americanum A. americanum -76.1647796 39.4442798 12000

Amblyomma americanum americanum A. americanum -80.822222 34.039167 12000

Amblyomma americanum americanum A. americanum -74.1514316 40.2555169 40840

Amblyomma americanum americanum A. americanum -76.1647796 39.4442798 12000

Amblyomma americanum americanum A. americanum -76.1647796 39.4442798 12000

Amblyomma americanum americanum A. americanum -76.1647796 39.4442798 12000

Amblyomma americanum americanum A. americanum -76.1647796 39.4442798 12000

Amblyomma americanum americanum A. americanum -76.1647796 39.4442798 12000

Amblyomma americanum americanum A. americanum -86.5295486 30.572647 44515

Amblyomma americanum americanum A. americanum -94.9129486 39.3571307 6500

Amblyomma americanum americanum A. americanum -94.9129486 39.3571307 6500

Amblyomma americanum americanum A. americanum -77.3341369 37.2359319 4000

Amblyomma americanum americanum A. americanum -76.1647796 39.4442798 12000

Amblyomma americanum americanum A. americanum -76.1647796 39.4442798 12000

Amblyomma americanum americanum A. americanum -76.1647796 39.4442798 12000

Amblyomma americanum americanum A. americanum -76.1647796 39.4442798 12000

Amblyomma americanum americanum A. americanum -76.1647796 39.4442798 12000

Amblyomma americanum americanum A. americanum -76.1647796 39.4442798 12000

Amblyomma americanum americanum A. americanum -87.6159667 36.6078112 18500

Amblyomma americanum americanum A. americanum -76.1647796 39.4442798 12000

Amblyomma americanum americanum A. americanum -76.1647796 39.4442798 12000

Amblyomma americanum americanum A. americanum -76.84515 39.035445 2000

Amblyomma americanum americanum A. americanum -76.1647796 39.4442798 12000

Amblyomma americanum americanum A. americanum -76.1647796 39.4442798 12000

Amblyomma americanum americanum A. americanum -76.1647796 39.4442798 12000

Amblyomma americanum americanum A. americanum -94.9129486 39.3571307 6500

Amblyomma americanum americanum A. americanum -76.1647796 39.4442798 12000

Amblyomma americanum americanum A. americanum -74.1514316 40.2555169 40840

Amblyomma americanum americanum A. americanum -76.7734909 39.0642483 10000

Amblyomma americanum americanum A. americanum -77.1459102 38.7021234 3500

Amblyomma americanum americanum A. americanum -87.6159667 36.6078112 18500

Amblyomma americanum americanum A. americanum -87.6159667 36.6078112 18500

Amblyomma americanum americanum A. americanum -85.8571243 37.9095336 21000

Amblyomma americanum americanum A. americanum -77.975979 34.007135 5000

Amblyomma americanum americanum A. americanum -76.1647796 39.4442798 12000

Amblyomma americanum americanum A. americanum -76.1647796 39.4442798 12000

Amblyomma americanum americanum A. americanum -76.1647796 39.4442798 12000

Amblyomma americanum americanum A. americanum -76.1647796 39.4442798 12000

Amblyomma americanum americanum A. americanum -87.6159667 36.6078112 18500

Amblyomma americanum americanum A. americanum -87.6159667 36.6078112 18500

Amblyomma americanum americanum A. americanum -86.6569934 34.6324885 12772

Amblyomma americanum americanum A. americanum -75.5948638 44.1156908 23000

Amblyomma americanum americanum A. americanum -77.915039 37.0420244 10500

Amblyomma americanum americanum A. americanum -87.6159667 36.6078112 18500

Amblyomma americanum americanum A. americanum -76.1647796 39.4442798 12000

Amblyomma americanum americanum A. americanum -87.6159667 36.6078112 18500

Amblyomma americanum americanum A. americanum -87.6159667 36.6078112 18500

Amblyomma americanum americanum A. americanum -77.915039 37.0420244 10500

Amblyomma americanum americanum A. americanum -76.1647796 39.4442798 12000

Amblyomma americanum americanum A. americanum -87.6159667 36.6078112 18500

Amblyomma americanum americanum A. americanum -92.1572583 37.7057025 16307

Amblyomma americanum americanum A. americanum -77.1459102 38.7021234 3500

Amblyomma americanum americanum A. americanum -76.1647796 39.4442798 12000

Amblyomma americanum americanum A. americanum -78.31089 36.600921 5000

Amblyomma americanum americanum A. americanum -76.84515 39.035445 2000

Amblyomma americanum americanum A. americanum -76.1647796 39.4442798 12000

Amblyomma americanum americanum A. americanum -76.1647796 39.4442798 12000

Amblyomma americanum americanum A. americanum -92.1572583 37.7057025 16307

Amblyomma americanum americanum A. americanum -76.1647796 39.4442798 12000

Amblyomma americanum americanum A. americanum -87.6159667 36.6078112 18500

Amblyomma americanum americanum A. americanum -77.3341369 37.2359319 4000

Amblyomma americanum americanum A. americanum -76.1647796 39.4442798 12000

Amblyomma americanum americanum A. americanum -76.1647796 39.4442798 12000

Amblyomma americanum americanum A. americanum -76.1647796 39.4442798 12000

Amblyomma americanum americanum A. americanum -78.31089 36.600921 5000

Amblyomma americanum americanum A. americanum -78.31089 36.600921 5000

Amblyomma americanum americanum A. americanum -74.576966 40.024197 8000

Amblyomma americanum americanum A. americanum -76.1647796 39.4442798 12000

Amblyomma americanum americanum A. americanum -77.3341369 37.2359319 4000

Amblyomma americanum americanum A. americanum -76.1647796 39.4442798 12000

Amblyomma americanum americanum A. americanum -87.6159667 36.6078112 18500

Amblyomma americanum americanum A. americanum -74.1514316 40.2555169 40840

Amblyomma americanum americanum A. americanum -76.1647796 39.4442798 12000

Amblyomma americanum americanum A. americanum -96.8206687 39.1865859 20922

Amblyomma americanum americanum A. americanum -76.1647796 39.4442798 12000

Amblyomma americanum americanum A. americanum -76.1647796 39.4442798 12000

Amblyomma americanum americanum A. americanum -76.1647796 39.4442798 12000

Amblyomma americanum americanum A. americanum -96.8206687 39.1865859 20922

Amblyomma americanum americanum A. americanum -76.1647796 39.4442798 12000

Amblyomma americanum americanum A. americanum -77.915039 37.0420244 10500

Amblyomma americanum americanum A. americanum -92.1572583 37.7057025 16307

Amblyomma americanum americanum A. americanum -76.1647796 39.4442798 12000

Amblyomma americanum americanum A. americanum -76.1647796 39.4442798 12000

Amblyomma americanum americanum A. americanum -76.1647796 39.4442798 12000

Amblyomma americanum americanum A. americanum -76.1647796 39.4442798 12000

Amblyomma americanum americanum A. americanum -76.1647796 39.4442798 12000

Amblyomma americanum americanum A. americanum -76.1647796 39.4442798 12000

Amblyomma americanum americanum A. americanum -76.1647796 39.4442798 12000

Amblyomma americanum americanum A. americanum -76.1647796 39.4442798 12000

Amblyomma americanum americanum A. americanum -76.1647796 39.4442798 12000

Amblyomma americanum americanum A. americanum -76.1647796 39.4442798 12000

Amblyomma americanum americanum A. americanum -87.6159667 36.6078112 18500

Amblyomma americanum americanum A. americanum -87.6159667 36.6078112 18500

Amblyomma americanum americanum A. americanum -87.6159667 36.6078112 18500

Amblyomma americanum americanum A. americanum -87.6159667 36.6078112 18500

Amblyomma americanum americanum A. americanum -77.915039 37.0420244 10500

Amblyomma americanum americanum A. americanum -76.1647796 39.4442798 12000

Amblyomma americanum americanum A. americanum -76.1647796 39.4442798 12000

Amblyomma americanum americanum A. americanum -76.1647796 39.4442798 12000

Amblyomma americanum americanum A. americanum -76.1647796 39.4442798 12000

Amblyomma americanum americanum A. americanum -76.1647796 39.4442798 12000

Amblyomma americanum americanum A. americanum -76.1647796 39.4442798 12000

Amblyomma americanum americanum A. americanum -76.1647796 39.4442798 12000

Amblyomma americanum americanum A. americanum -76.1647796 39.4442798 12000

Amblyomma americanum americanum A. americanum -76.1647796 39.4442798 12000

Amblyomma americanum americanum A. americanum -77.1459102 38.7021234 3500

Amblyomma americanum americanum A. americanum -77.1459102 38.7021234 3500

Amblyomma americanum americanum A. americanum -77.1459102 38.7021234 3500

Amblyomma americanum americanum A. americanum -77.0184516 38.8393064 3500

Amblyomma americanum americanum A. americanum -78.31089 36.600921 5000

Amblyomma americanum americanum A. americanum -87.6159667 36.6078112 18500

Amblyomma americanum americanum A. americanum -87.6159667 36.6078112 18500

Amblyomma americanum americanum A. americanum -74.576966 40.024197 8000

Amblyomma americanum americanum A. americanum -74.576966 40.024197 8000

Amblyomma americanum americanum A. americanum -74.576966 40.024197 8000

Amblyomma americanum americanum A. americanum -74.576966 40.024197 8000

Amblyomma americanum americanum A. americanum -74.576966 40.024197 8000

Amblyomma americanum americanum A. americanum -74.576966 40.024197 8000

Amblyomma americanum americanum A. americanum -74.576966 40.024197 8000

Amblyomma americanum americanum A. americanum -85.8571243 37.9095336 21000

Amblyomma americanum americanum A. americanum -85.8571243 37.9095336 21000

Amblyomma americanum americanum A. americanum -85.8571243 37.9095336 21000

Amblyomma americanum americanum A. americanum -95.923889 34.826667 12000

Amblyomma americanum americanum A. americanum -96.8206687 39.1865859 20922

Amblyomma americanum americanum A. americanum -76.1647796 39.4442798 12000

Amblyomma americanum americanum A. americanum -92.23117 34.827169 100

Amblyomma americanum americanum A. americanum -78.31089 36.600921 5000

Amblyomma americanum americanum A. americanum -87.6159667 36.6078112 18500

Amblyomma americanum americanum A. americanum -87.6159667 36.6078112 18500

Amblyomma americanum americanum A. americanum -86.5295486 30.572647 44515

Amblyomma americanum americanum A. americanum -78.5825 35.941667 1000

Amblyomma americanum americanum A. americanum -74.576966 40.024197 8000

Amblyomma americanum americanum A. americanum -74.1514316 40.2555169 40840

Amblyomma americanum americanum A. americanum -76.1647796 39.4442798 12000

Amblyomma americanum americanum A. americanum -76.8773459 38.8066212 3000

Amblyomma americanum americanum A. americanum -74.576966 40.024197 8000

Amblyomma americanum americanum A. americanum -76.1647796 39.4442798 12000

Amblyomma americanum americanum A. americanum -76.1647796 39.4442798 12000

Amblyomma americanum americanum A. americanum -76.1647796 39.4442798 12000

Amblyomma americanum americanum A. americanum -77.1459102 38.7021234 3500

Amblyomma americanum americanum A. americanum -74.576966 40.024197 8000

Amblyomma americanum americanum A. americanum -74.576966 40.024197 8000

Amblyomma americanum americanum A. americanum -81.6164822 31.9937096 34659

Amblyomma americanum americanum A. americanum -76.1647796 39.4442798 12000

Amblyomma americanum americanum A. americanum -76.1647796 39.4442798 12000

Amblyomma americanum americanum A. americanum -76.1647796 39.4442798 12000

Amblyomma americanum americanum A. americanum -76.1647796 39.4442798 12000

Amblyomma americanum americanum A. americanum -76.1647796 39.4442798 12000

Amblyomma americanum americanum A. americanum -96.8206687 39.1865859 20922

Amblyomma americanum americanum A. americanum -96.8206687 39.1865859 20922

Amblyomma americanum americanum A. americanum -96.8206687 39.1865859 20922

Amblyomma americanum americanum A. americanum -96.8206687 39.1865859 20922

Amblyomma americanum americanum A. americanum -76.1647796 39.4442798 12000

Amblyomma americanum americanum A. americanum -76.1647796 39.4442798 12000

Amblyomma americanum americanum A. americanum -78.31089 36.600921 5000

Amblyomma americanum americanum A. americanum -74.576966 40.024197 8000

Amblyomma americanum americanum A. americanum -74.576966 40.024197 8000

Amblyomma americanum americanum A. americanum -74.576966 40.024197 8000

Amblyomma americanum americanum A. americanum -74.576966 40.024197 8000

Amblyomma americanum americanum A. americanum -79.070374 35.654428 2000

Amblyomma americanum americanum A. americanum -77.915039 37.0420244 10500

Amblyomma americanum americanum A. americanum -94.290393 33.434429 8000

Amblyomma americanum americanum A. americanum -76.1647796 39.4442798 12000

Amblyomma americanum americanum A. americanum -76.1647796 39.4442798 12000

Amblyomma americanum americanum A. americanum -76.1647796 39.4442798 12000

Amblyomma americanum americanum A. americanum -77.1459102 38.7021234 3500

Amblyomma americanum americanum A. americanum -77.1459102 38.7021234 3500

Amblyomma americanum americanum A. americanum -77.1459102 38.7021234 3500

Amblyomma americanum americanum A. americanum -98.4835099 34.6823508 20062

Amblyomma americanum americanum A. americanum -98.4835099 34.6823508 20062

Amblyomma americanum americanum A. americanum -76.1647796 39.4442798 12000

Amblyomma americanum americanum A. americanum -77.3341369 37.2359319 4000

Amblyomma americanum americanum A. americanum -76.1647796 39.4442798 12000

Amblyomma americanum americanum A. americanum -76.1647796 39.4442798 12000

Amblyomma americanum americanum A. americanum -76.1647796 39.4442798 12000

Amblyomma americanum americanum A. americanum -87.6159667 36.6078112 18500

Amblyomma americanum americanum A. americanum -87.6159667 36.6078112 18500

Amblyomma americanum americanum A. americanum -74.1514316 40.2555169 40840

Amblyomma americanum americanum A. americanum -86.5295486 30.572647 44515

Amblyomma americanum americanum A. americanum -71.289167 42.47 3000

Amblyomma americanum americanum A. americanum -74.576966 40.024197 8000

Amblyomma americanum americanum A. americanum -94.9129486 39.3571307 6500

Amblyomma americanum americanum A. americanum -74.1514316 40.2555169 40840

Amblyomma americanum americanum A. americanum -74.5435553 40.9548927 23433

Amblyomma americanum americanum A. americanum -76.1647796 39.4442798 12000

Amblyomma americanum americanum A. americanum -76.1647796 39.4442798 12000

Amblyomma americanum americanum A. americanum -76.1647796 39.4442798 12000

Amblyomma americanum americanum A. americanum -77.1459102 38.7021234 3500

Amblyomma americanum americanum A. americanum -77.1459102 38.7021234 3500

Amblyomma americanum americanum A. americanum -71.289167 42.47 3000

Amblyomma americanum americanum A. americanum -74.576966 40.024197 8000

Amblyomma americanum americanum A. americanum -77.3341369 37.2359319 4000

Amblyomma americanum americanum A. americanum -76.1647796 39.4442798 12000

Amblyomma americanum americanum A. americanum -76.1647796 39.4442798 12000

Amblyomma americanum americanum A. americanum -76.1647796 39.4442798 12000

Amblyomma americanum americanum A. americanum -76.1647796 39.4442798 12000

Amblyomma americanum americanum A. americanum -76.1647796 39.4442798 12000

Amblyomma americanum americanum A. americanum -77.1459102 38.7021234 3500

Amblyomma americanum americanum A. americanum -77.1459102 38.7021234 3500

Amblyomma americanum americanum A. americanum -77.1459102 38.7021234 3500

Amblyomma americanum americanum A. americanum -77.1459102 38.7021234 3500

Amblyomma americanum americanum A. americanum -96.8206687 39.1865859 20922

Amblyomma americanum americanum A. americanum -92.1572583 37.7057025 16307

Amblyomma americanum americanum A. americanum -92.1572583 37.7057025 16307

Amblyomma americanum americanum A. americanum -92.1572583 37.7057025 16307

Amblyomma americanum americanum A. americanum -76.1647796 39.4442798 12000

Amblyomma americanum americanum A. americanum -76.1647796 39.4442798 12000

Amblyomma americanum americanum A. americanum -76.1647796 39.4442798 12000

Amblyomma americanum americanum A. americanum -77.1459102 38.7021234 3500

Amblyomma americanum americanum A. americanum -77.1459102 38.7021234 3500

Amblyomma americanum americanum A. americanum -77.1459102 38.7021234 3500

Amblyomma americanum americanum A. americanum -79.1970062 35.1109218 17000

Amblyomma americanum americanum A. americanum -122.578636 47.100278 12000

Amblyomma americanum americanum A. americanum -74.576966 40.024197 8000

Amblyomma americanum americanum A. americanum -74.576966 40.024197 8000

Amblyomma americanum americanum A. americanum -74.576966 40.024197 8000

Amblyomma americanum americanum A. americanum -74.576966 40.024197 8000

Amblyomma americanum americanum A. americanum -79.070374 35.654428 2000

Amblyomma americanum americanum A. americanum -79.070374 35.654428 2000

Amblyomma americanum americanum A. americanum -76.7734909 39.0642483 10000

Amblyomma americanum americanum A. americanum -76.7734909 39.0642483 10000

Amblyomma americanum americanum A. americanum -74.1514316 40.2555169 40840

Amblyomma americanum americanum A. americanum -74.1514316 40.2555169 40840

Amblyomma americanum americanum A. americanum -77.915039 37.0420244 10500

Amblyomma americanum americanum A. americanum -86.6569934 34.6324885 12772

Amblyomma americanum americanum A. americanum -76.84515 39.035445 2000

Amblyomma americanum americanum A. americanum -76.84515 39.035445 2000

Amblyomma americanum americanum A. americanum -76.1647796 39.4442798 12000

Amblyomma americanum americanum A. americanum -74.576966 40.024197 8000

Amblyomma americanum americanum A. americanum -94.9129486 39.3571307 6500

Amblyomma americanum americanum A. americanum -77.3341369 37.2359319 4000

Amblyomma americanum americanum A. americanum -76.7734909 39.0642483 10000

Amblyomma americanum americanum A. americanum -76.1647796 39.4442798 12000

Amblyomma americanum americanum A. americanum -76.1647796 39.4442798 12000

Amblyomma americanum americanum A. americanum -76.1647796 39.4442798 12000

Amblyomma americanum americanum A. americanum -76.1647796 39.4442798 12000

Amblyomma americanum americanum A. americanum -76.1647796 39.4442798 12000

Amblyomma americanum americanum A. americanum -76.1647796 39.4442798 12000

Amblyomma americanum americanum A. americanum -76.1647796 39.4442798 12000

Amblyomma americanum americanum A. americanum -76.1647796 39.4442798 12000

Amblyomma americanum americanum A. americanum -76.1647796 39.4442798 12000

Amblyomma americanum americanum A. americanum -76.1647796 39.4442798 12000

Amblyomma americanum americanum A. americanum -87.6159667 36.6078112 18500

Amblyomma americanum americanum A. americanum -87.6159667 36.6078112 18500

Amblyomma americanum americanum A. americanum -74.1514316 40.2555169 40840

Amblyomma americanum americanum A. americanum -74.1514316 40.2555169 40840

Amblyomma americanum americanum A. americanum -74.1514316 40.2555169 40840

Amblyomma americanum americanum A. americanum -92.1572583 37.7057025 16307

Amblyomma americanum americanum A. americanum -92.1572583 37.7057025 16307

Amblyomma americanum americanum A. americanum -92.1572583 37.7057025 16307

Amblyomma americanum americanum A. americanum -92.1572583 37.7057025 16307

Amblyomma americanum americanum A. americanum -74.576966 40.024197 8000

Amblyomma americanum americanum A. americanum -74.576966 40.024197 8000

Amblyomma americanum americanum A. americanum -74.1514316 40.2555169 40840

Amblyomma americanum americanum A. americanum -76.1647796 39.4442798 12000

Amblyomma americanum americanum A. americanum -77.1459102 38.7021234 3500

Amblyomma americanum americanum A. americanum -77.1459102 38.7021234 3500

Amblyomma americanum americanum A. americanum -75.5948638 44.1156908 23000

Amblyomma americanum americanum A. americanum -74.576966 40.024197 8000

Amblyomma americanum americanum A. americanum -74.576966 40.024197 8000

Amblyomma americanum americanum A. americanum -74.576966 40.024197 8000

Amblyomma americanum americanum A. americanum -85.8571243 37.9095336 21000

Amblyomma americanum americanum A. americanum -85.8571243 37.9095336 21000

Amblyomma americanum americanum A. americanum -85.8571243 37.9095336 21000

Amblyomma americanum americanum A. americanum -77.3341369 37.2359319 4000

Amblyomma americanum americanum A. americanum -77.3341369 37.2359319 4000

Amblyomma americanum americanum A. americanum -77.3341369 37.2359319 4000

Amblyomma americanum americanum A. americanum -77.3341369 37.2359319 4000

Amblyomma americanum americanum A. americanum -77.3341369 37.2359319 4000

Amblyomma americanum americanum A. americanum -77.3341369 37.2359319 4000

Amblyomma americanum americanum A. americanum -77.3341369 37.2359319 4000

Amblyomma americanum americanum A. americanum -77.3341369 37.2359319 4000

Amblyomma americanum americanum A. americanum -77.3341369 37.2359319 4000

Amblyomma americanum americanum A. americanum -77.3341369 37.2359319 4000

Amblyomma americanum americanum A. americanum -77.3341369 37.2359319 4000

Amblyomma americanum americanum A. americanum -77.3341369 37.2359319 4000

Amblyomma americanum americanum A. americanum -80.719539 34.010264 3000

Amblyomma americanum americanum A. americanum -77.4568391 38.5520281 15927

Amblyomma americanum americanum A. americanum -92.1572583 37.7057025 16307

Amblyomma americanum americanum A. americanum -92.1572583 37.7057025 16307

Amblyomma americanum americanum A. americanum -76.1647796 39.4442798 12000

Amblyomma americanum americanum A. americanum -76.1647796 39.4442798 12000

Amblyomma americanum americanum A. americanum -76.1647796 39.4442798 12000

Amblyomma americanum americanum A. americanum -76.1647796 39.4442798 12000

Amblyomma americanum americanum A. americanum -76.1647796 39.4442798 12000

Amblyomma americanum americanum A. americanum -76.882867 38.996036 500

Amblyomma americanum americanum A. americanum -76.882867 38.996036 500

Amblyomma americanum americanum A. americanum -76.882867 38.996036 500

Amblyomma americanum americanum A. americanum -76.882867 38.996036 500

Amblyomma americanum americanum A. americanum -76.882867 38.996036 500

Amblyomma americanum americanum A. americanum -76.882867 38.996036 500

Amblyomma americanum americanum A. americanum -76.882867 38.996036 500

Amblyomma americanum americanum A. americanum -76.882867 38.996036 500

Amblyomma americanum americanum A. americanum -76.1647796 39.4442798 12000

Amblyomma americanum americanum A. americanum -76.1647796 39.4442798 12000

Amblyomma americanum americanum A. americanum -74.576966 40.024197 8000

Amblyomma americanum americanum A. americanum -74.576966 40.024197 8000

Amblyomma americanum americanum A. americanum -75.5948638 44.1156908 23000

Amblyomma americanum americanum A. americanum -76.1647796 39.4442798 12000

Amblyomma americanum americanum A. americanum -74.576966 40.024197 8000

Amblyomma americanum americanum A. americanum -92.1572583 37.7057025 16307

Amblyomma americanum americanum A. americanum -74.576966 40.024197 8000

Amblyomma americanum americanum A. americanum -76.882867 38.996036 500

Amblyomma americanum americanum A. americanum -76.882867 38.996036 500

Amblyomma americanum americanum A. americanum -76.882867 38.996036 500

Amblyomma americanum americanum A. americanum -76.882867 38.996036 500

Amblyomma americanum americanum A. americanum -76.882867 38.996036 500

Amblyomma americanum americanum A. americanum -76.882867 38.996036 500

Amblyomma americanum americanum A. americanum -76.882867 38.996036 500

Amblyomma americanum americanum A. americanum -76.882867 38.996036 500

Amblyomma americanum americanum A. americanum -76.882867 38.996036 500

Amblyomma americanum americanum A. americanum -76.882867 38.996036 500

Amblyomma americanum americanum A. americanum -76.882867 38.996036 500

Amblyomma americanum americanum A. americanum -76.882867 38.996036 500

Amblyomma americanum americanum A. americanum -76.882867 38.996036 500

Amblyomma americanum americanum A. americanum -76.882867 38.996036 500

Amblyomma americanum americanum A. americanum -76.882867 38.996036 500

Amblyomma americanum americanum A. americanum -76.882867 38.996036 500

Amblyomma americanum americanum A. americanum -76.882867 38.996036 500

Amblyomma americanum americanum A. americanum -76.882867 38.996036 500

Amblyomma americanum americanum A. americanum -76.882867 38.996036 500

Amblyomma americanum americanum A. americanum -76.882867 38.996036 500

Amblyomma americanum americanum A. americanum -76.882867 38.996036 500

Amblyomma americanum americanum A. americanum -76.882867 38.996036 500

Amblyomma americanum americanum A. americanum -76.882867 38.996036 500

Amblyomma americanum americanum A. americanum -76.882867 38.996036 500

Amblyomma americanum americanum A. americanum -76.882867 38.996036 500

Amblyomma americanum americanum A. americanum -76.882867 38.996036 500

Amblyomma americanum americanum A. americanum -76.882867 38.996036 500

Amblyomma americanum americanum A. americanum -76.882867 38.996036 500

Amblyomma americanum americanum A. americanum -76.882867 38.996036 500

Amblyomma americanum americanum A. americanum -76.882867 38.996036 500

Amblyomma americanum americanum A. americanum -76.882867 38.996036 500

Amblyomma americanum americanum A. americanum -76.882867 38.996036 500

Amblyomma americanum americanum A. americanum -76.882867 38.996036 500

Amblyomma americanum americanum A. americanum -76.882867 38.996036 500

Amblyomma americanum americanum A. americanum -76.882867 38.996036 500

Amblyomma americanum americanum A. americanum -76.882867 38.996036 500

Amblyomma americanum americanum A. americanum -76.882867 38.996036 500

Amblyomma americanum americanum A. americanum -76.882867 38.996036 500

Amblyomma americanum americanum A. americanum -76.882867 38.996036 500

Amblyomma americanum americanum A. americanum -76.882867 38.996036 500

Amblyomma americanum americanum A. americanum -76.882867 38.996036 500

Amblyomma americanum americanum A. americanum -76.882867 38.996036 500

Amblyomma americanum americanum A. americanum -76.882867 38.996036 500

Amblyomma americanum americanum A. americanum -76.1647796 39.4442798 12000

Amblyomma americanum americanum A. americanum -76.1647796 39.4442798 12000

Amblyomma americanum americanum A. americanum -76.1647796 39.4442798 12000

Amblyomma americanum americanum A. americanum -76.1647796 39.4442798 12000

Amblyomma americanum americanum A. americanum -76.1647796 39.4442798 12000

Amblyomma americanum americanum A. americanum -77.1459102 38.7021234 3500

Amblyomma americanum americanum A. americanum -77.1459102 38.7021234 3500

Amblyomma americanum americanum A. americanum -87.6159667 36.6078112 18500

Amblyomma americanum americanum A. americanum -122.578636 47.100278 12000

Amblyomma americanum americanum A. americanum -74.576966 40.024197 8000

Amblyomma americanum americanum A. americanum -74.576966 40.024197 8000

Amblyomma americanum americanum A. americanum -74.576966 40.024197 8000

Amblyomma americanum americanum A. americanum -74.576966 40.024197 8000

Amblyomma americanum americanum A. americanum -74.576966 40.024197 8000

Amblyomma americanum americanum A. americanum -96.8206687 39.1865859 20922

Amblyomma americanum americanum A. americanum -76.1647796 39.4442798 12000

Amblyomma americanum americanum A. americanum -76.1647796 39.4442798 12000

Amblyomma americanum americanum A. americanum -76.1647796 39.4442798 12000

Amblyomma americanum americanum A. americanum -76.1647796 39.4442798 12000

Amblyomma americanum americanum A. americanum -76.1647796 39.4442798 12000

Amblyomma americanum americanum A. americanum -76.1647796 39.4442798 12000

Amblyomma americanum americanum A. americanum -76.1647796 39.4442798 12000

Amblyomma americanum americanum A. americanum -76.1647796 39.4442798 12000

Amblyomma americanum americanum A. americanum -76.1647796 39.4442798 12000

Amblyomma americanum americanum A. americanum -76.1647796 39.4442798 12000

Amblyomma americanum americanum A. americanum -76.1647796 39.4442798 12000

Amblyomma americanum americanum A. americanum -87.6159667 36.6078112 18500

Amblyomma americanum americanum A. americanum -87.6159667 36.6078112 18500

Amblyomma americanum americanum A. americanum -87.6159667 36.6078112 18500

Amblyomma americanum americanum A. americanum -87.6159667 36.6078112 18500

Amblyomma americanum americanum A. americanum -86.5295486 30.572647 44515

Amblyomma americanum americanum A. americanum -74.576966 40.024197 8000

Amblyomma americanum americanum A. americanum -74.576966 40.024197 8000

Amblyomma americanum americanum A. americanum -74.576966 40.024197 8000

Amblyomma americanum americanum A. americanum -74.576966 40.024197 8000

Amblyomma americanum americanum A. americanum -74.576966 40.024197 8000

Amblyomma americanum americanum A. americanum -74.576966 40.024197 8000

Amblyomma americanum americanum A. americanum -76.882867 38.996036 500

Amblyomma americanum americanum A. americanum -76.882867 38.996036 500

Amblyomma americanum americanum A. americanum -76.882867 38.996036 500

Amblyomma americanum americanum A. americanum -76.882867 38.996036 500

Amblyomma americanum americanum A. americanum -76.882867 38.996036 500

Amblyomma americanum americanum A. americanum -76.882867 38.996036 500

Amblyomma americanum americanum A. americanum -76.882867 38.996036 500

Amblyomma americanum americanum A. americanum -76.882867 38.996036 500

Amblyomma americanum americanum A. americanum -76.882867 38.996036 500

Amblyomma americanum americanum A. americanum -76.882867 38.996036 500

Amblyomma americanum americanum A. americanum -76.882867 38.996036 500

Amblyomma americanum americanum A. americanum -76.882867 38.996036 500

Amblyomma americanum americanum A. americanum -76.7734909 39.0642483 10000

Amblyomma americanum americanum A. americanum -76.7734909 39.0642483 10000

Amblyomma americanum americanum A. americanum -77.915039 37.0420244 10500

Amblyomma americanum americanum A. americanum -77.915039 37.0420244 10500

Amblyomma americanum americanum A. americanum -77.4568391 38.5520281 15927

Amblyomma americanum americanum A. americanum -77.4568391 38.5520281 15927

Amblyomma americanum americanum A. americanum -77.4568391 38.5520281 15927

Amblyomma americanum americanum A. americanum -77.4568391 38.5520281 15927

Amblyomma americanum americanum A. americanum -77.4568391 38.5520281 15927

Amblyomma americanum americanum A. americanum -77.4568391 38.5520281 15927

Amblyomma americanum americanum A. americanum -77.4568391 38.5520281 15927

Amblyomma americanum americanum A. americanum -77.4568391 38.5520281 15927

Amblyomma americanum americanum A. americanum -76.84515 39.035445 2000

Amblyomma americanum americanum A. americanum -76.84515 39.035445 2000

Amblyomma americanum americanum A. americanum -76.84515 39.035445 2000

Amblyomma americanum americanum A. americanum -76.1647796 39.4442798 12000

Amblyomma americanum americanum A. americanum -76.1647796 39.4442798 12000

Amblyomma americanum americanum A. americanum -76.1647796 39.4442798 12000

Amblyomma americanum americanum A. americanum -76.1647796 39.4442798 12000

Amblyomma americanum americanum A. americanum -76.1647796 39.4442798 12000

Amblyomma americanum americanum A. americanum -76.1647796 39.4442798 12000

Amblyomma americanum americanum A. americanum -76.1647796 39.4442798 12000

Amblyomma americanum americanum A. americanum -76.1647796 39.4442798 12000

Amblyomma americanum americanum A. americanum -79.1970062 35.1109218 17000

Amblyomma americanum americanum A. americanum -87.6159667 36.6078112 18500

Amblyomma americanum americanum A. americanum -86.5295486 30.572647 44515

Amblyomma americanum americanum A. americanum -74.576966 40.024197 8000

Amblyomma americanum americanum A. americanum -74.576966 40.024197 8000

Amblyomma americanum americanum A. americanum -74.576966 40.024197 8000

Amblyomma americanum americanum A. americanum -74.576966 40.024197 8000

Amblyomma americanum americanum A. americanum -85.8571243 37.9095336 21000

Amblyomma americanum americanum A. americanum -85.8571243 37.9095336 21000

Amblyomma americanum americanum A. americanum -85.8571243 37.9095336 21000

Amblyomma americanum americanum A. americanum -85.8571243 37.9095336 21000

Amblyomma americanum americanum A. americanum -85.8571243 37.9095336 21000

Amblyomma americanum americanum A. americanum -85.8571243 37.9095336 21000

Amblyomma americanum americanum A. americanum -85.8571243 37.9095336 21000

Amblyomma americanum americanum A. americanum -85.8571243 37.9095336 21000

Amblyomma americanum americanum A. americanum -85.8571243 37.9095336 21000

Amblyomma americanum americanum A. americanum -85.8571243 37.9095336 21000

Amblyomma americanum americanum A. americanum -85.8571243 37.9095336 21000

Amblyomma americanum americanum A. americanum -85.8571243 37.9095336 21000

Amblyomma americanum americanum A. americanum -85.8571243 37.9095336 21000

Amblyomma americanum americanum A. americanum -85.8571243 37.9095336 21000

Amblyomma americanum americanum A. americanum -85.8571243 37.9095336 21000

Amblyomma americanum americanum A. americanum -85.8571243 37.9095336 21000

Amblyomma americanum americanum A. americanum -85.8571243 37.9095336 21000

Amblyomma americanum americanum A. americanum -85.8571243 37.9095336 21000

Amblyomma americanum americanum A. americanum -85.8571243 37.9095336 21000

Amblyomma americanum americanum A. americanum -85.8571243 37.9095336 21000

Amblyomma americanum americanum A. americanum -85.8571243 37.9095336 21000

Amblyomma americanum americanum A. americanum -85.8571243 37.9095336 21000

Amblyomma americanum americanum A. americanum -85.8571243 37.9095336 21000

Amblyomma americanum americanum A. americanum -85.8571243 37.9095336 21000

Amblyomma americanum americanum A. americanum -85.8571243 37.9095336 21000

Amblyomma americanum americanum A. americanum -85.8571243 37.9095336 21000

Amblyomma americanum americanum A. americanum -85.8571243 37.9095336 21000

Amblyomma americanum americanum A. americanum -85.8571243 37.9095336 21000

Amblyomma americanum americanum A. americanum -85.8571243 37.9095336 21000

Amblyomma americanum americanum A. americanum -85.8571243 37.9095336 21000

Amblyomma americanum americanum A. americanum -85.8571243 37.9095336 21000

Amblyomma americanum americanum A. americanum -85.8571243 37.9095336 21000

Amblyomma americanum americanum A. americanum -85.8571243 37.9095336 21000

Amblyomma americanum americanum A. americanum -85.8571243 37.9095336 21000

Amblyomma americanum americanum A. americanum -85.8571243 37.9095336 21000

Amblyomma americanum americanum A. americanum -85.8571243 37.9095336 21000

Amblyomma americanum americanum A. americanum -85.8571243 37.9095336 21000

Amblyomma americanum americanum A. americanum -85.8571243 37.9095336 21000

Amblyomma americanum americanum A. americanum -85.8571243 37.9095336 21000

Amblyomma americanum americanum A. americanum -85.8571243 37.9095336 21000

Amblyomma americanum americanum A. americanum -85.8571243 37.9095336 21000

Amblyomma americanum americanum A. americanum -85.8571243 37.9095336 21000

Amblyomma americanum americanum A. americanum -85.8571243 37.9095336 21000

Amblyomma americanum americanum A. americanum -85.8571243 37.9095336 21000

Amblyomma americanum americanum A. americanum -85.8571243 37.9095336 21000

Amblyomma americanum americanum A. americanum -85.8571243 37.9095336 21000

Amblyomma americanum americanum A. americanum -85.8571243 37.9095336 21000

Amblyomma americanum americanum A. americanum -85.8571243 37.9095336 21000

Amblyomma americanum americanum A. americanum -85.8571243 37.9095336 21000

Amblyomma americanum americanum A. americanum -85.8571243 37.9095336 21000

Amblyomma americanum americanum A. americanum -85.8571243 37.9095336 21000

Amblyomma americanum americanum A. americanum -85.8571243 37.9095336 21000

Amblyomma americanum americanum A. americanum -85.8571243 37.9095336 21000

Amblyomma americanum americanum A. americanum -85.8571243 37.9095336 21000

Amblyomma americanum americanum A. americanum -85.8571243 37.9095336 21000

Amblyomma americanum americanum A. americanum -85.8571243 37.9095336 21000

Amblyomma americanum americanum A. americanum -85.8571243 37.9095336 21000

Amblyomma americanum americanum A. americanum -85.8571243 37.9095336 21000

Amblyomma americanum americanum A. americanum -85.8571243 37.9095336 21000

Amblyomma americanum americanum A. americanum -85.8571243 37.9095336 21000

Amblyomma americanum americanum A. americanum -85.8571243 37.9095336 21000

Amblyomma americanum americanum A. americanum -85.8571243 37.9095336 21000

Amblyomma americanum americanum A. americanum -85.8571243 37.9095336 21000

Amblyomma americanum americanum A. americanum -85.8571243 37.9095336 21000

Amblyomma americanum americanum A. americanum -85.8571243 37.9095336 21000

Amblyomma americanum americanum A. americanum -85.8571243 37.9095336 21000

Amblyomma americanum americanum A. americanum -85.8571243 37.9095336 21000

Amblyomma americanum americanum A. americanum -85.8571243 37.9095336 21000

Amblyomma americanum americanum A. americanum -85.8571243 37.9095336 21000

Amblyomma americanum americanum A. americanum -85.8571243 37.9095336 21000

Amblyomma americanum americanum A. americanum -77.3341369 37.2359319 4000

Amblyomma americanum americanum A. americanum -77.3341369 37.2359319 4000

Amblyomma americanum americanum A. americanum -85.8571243 37.9095336 21000

Amblyomma americanum americanum A. americanum -85.8571243 37.9095336 21000

Amblyomma americanum americanum A. americanum -85.8571243 37.9095336 21000

Amblyomma americanum americanum A. americanum -85.8571243 37.9095336 21000

Amblyomma americanum americanum A. americanum -85.8571243 37.9095336 21000

Amblyomma americanum americanum A. americanum -85.8571243 37.9095336 21000

Amblyomma americanum americanum A. americanum -85.8571243 37.9095336 21000

Amblyomma americanum americanum A. americanum -85.8571243 37.9095336 21000

Amblyomma americanum americanum A. americanum -85.8571243 37.9095336 21000

Amblyomma americanum americanum A. americanum -85.8571243 37.9095336 21000

Amblyomma americanum americanum A. americanum -85.8571243 37.9095336 21000

Amblyomma americanum americanum A. americanum -85.8571243 37.9095336 21000

Amblyomma americanum americanum A. americanum -85.8571243 37.9095336 21000

Amblyomma americanum americanum A. americanum -85.8571243 37.9095336 21000

Amblyomma americanum americanum A. americanum -85.8571243 37.9095336 21000

Amblyomma americanum americanum A. americanum -85.8571243 37.9095336 21000

Amblyomma americanum americanum A. americanum -85.8571243 37.9095336 21000

Amblyomma americanum americanum A. americanum -85.8571243 37.9095336 21000

Amblyomma americanum americanum A. americanum -85.8571243 37.9095336 21000

Amblyomma americanum americanum A. americanum -85.8571243 37.9095336 21000

Amblyomma americanum americanum A. americanum -85.8571243 37.9095336 21000

Amblyomma americanum americanum A. americanum -85.8571243 37.9095336 21000

Amblyomma americanum americanum A. americanum -85.8571243 37.9095336 21000

Amblyomma americanum americanum A. americanum -85.8571243 37.9095336 21000

Amblyomma americanum americanum A. americanum -85.8571243 37.9095336 21000

Amblyomma americanum americanum A. americanum -85.8571243 37.9095336 21000

Amblyomma americanum americanum A. americanum -85.8571243 37.9095336 21000

Amblyomma americanum americanum A. americanum -85.8571243 37.9095336 21000

Amblyomma americanum americanum A. americanum -85.8571243 37.9095336 21000

Amblyomma americanum americanum A. americanum -85.8571243 37.9095336 21000

Amblyomma americanum americanum A. americanum -85.8571243 37.9095336 21000

Amblyomma americanum americanum A. americanum -85.8571243 37.9095336 21000

Amblyomma americanum americanum A. americanum -85.8571243 37.9095336 21000

Amblyomma americanum americanum A. americanum -85.8571243 37.9095336 21000

Amblyomma americanum americanum A. americanum -85.8571243 37.9095336 21000

Amblyomma americanum americanum A. americanum -85.8571243 37.9095336 21000

Amblyomma americanum americanum A. americanum -85.8571243 37.9095336 21000

Amblyomma americanum americanum A. americanum -85.8571243 37.9095336 21000

Amblyomma americanum americanum A. americanum -85.8571243 37.9095336 21000

Amblyomma americanum americanum A. americanum -85.8571243 37.9095336 21000

Amblyomma americanum americanum A. americanum -85.8571243 37.9095336 21000

Amblyomma americanum americanum A. americanum -85.8571243 37.9095336 21000

Amblyomma americanum americanum A. americanum -85.8571243 37.9095336 21000

Amblyomma americanum americanum A. americanum -85.8571243 37.9095336 21000

Amblyomma americanum americanum A. americanum -85.8571243 37.9095336 21000

Amblyomma americanum americanum A. americanum -85.8571243 37.9095336 21000

Amblyomma americanum americanum A. americanum -85.8571243 37.9095336 21000

Amblyomma americanum americanum A. americanum -85.8571243 37.9095336 21000

Amblyomma americanum americanum A. americanum -85.8571243 37.9095336 21000

Amblyomma americanum americanum A. americanum -85.8571243 37.9095336 21000

Amblyomma americanum americanum A. americanum -85.8571243 37.9095336 21000

Amblyomma americanum americanum A. americanum -85.8571243 37.9095336 21000

Amblyomma americanum americanum A. americanum -85.8571243 37.9095336 21000

Amblyomma americanum americanum A. americanum -85.8571243 37.9095336 21000

Amblyomma americanum americanum A. americanum -85.8571243 37.9095336 21000

Amblyomma americanum americanum A. americanum -85.8571243 37.9095336 21000

Amblyomma americanum americanum A. americanum -85.8571243 37.9095336 21000

Amblyomma americanum americanum A. americanum -85.8571243 37.9095336 21000

Amblyomma americanum americanum A. americanum -85.8571243 37.9095336 21000

Amblyomma americanum americanum A. americanum -85.8571243 37.9095336 21000

Amblyomma americanum americanum A. americanum -85.8571243 37.9095336 21000

Amblyomma americanum americanum A. americanum -85.8571243 37.9095336 21000

Amblyomma americanum americanum A. americanum -85.8571243 37.9095336 21000

Amblyomma americanum americanum A. americanum -85.8571243 37.9095336 21000

Amblyomma americanum americanum A. americanum -85.8571243 37.9095336 21000

Amblyomma americanum americanum A. americanum -85.8571243 37.9095336 21000

Amblyomma americanum americanum A. americanum -85.8571243 37.9095336 21000

Amblyomma americanum americanum A. americanum -85.8571243 37.9095336 21000

Amblyomma americanum americanum A. americanum -85.8571243 37.9095336 21000

Amblyomma americanum americanum A. americanum -85.8571243 37.9095336 21000

Amblyomma americanum americanum A. americanum -85.8571243 37.9095336 21000

Amblyomma americanum americanum A. americanum -85.8571243 37.9095336 21000

Amblyomma americanum americanum A. americanum -85.8571243 37.9095336 21000

Amblyomma americanum americanum A. americanum -85.8571243 37.9095336 21000

Amblyomma americanum americanum A. americanum -85.8571243 37.9095336 21000

Amblyomma americanum americanum A. americanum -85.8571243 37.9095336 21000

Amblyomma americanum americanum A. americanum -85.8571243 37.9095336 21000

Amblyomma americanum americanum A. americanum -85.8571243 37.9095336 21000

Amblyomma americanum americanum A. americanum -85.8571243 37.9095336 21000

Amblyomma americanum americanum A. americanum -85.8571243 37.9095336 21000

Amblyomma americanum americanum A. americanum -85.8571243 37.9095336 21000

Amblyomma americanum americanum A. americanum -85.8571243 37.9095336 21000

Amblyomma americanum americanum A. americanum -85.8571243 37.9095336 21000

Amblyomma americanum americanum A. americanum -85.8571243 37.9095336 21000

Amblyomma americanum americanum A. americanum -85.8571243 37.9095336 21000

Amblyomma americanum americanum A. americanum -85.8571243 37.9095336 21000

Amblyomma americanum americanum A. americanum -85.8571243 37.9095336 21000

Amblyomma americanum americanum A. americanum -85.8571243 37.9095336 21000

Amblyomma americanum americanum A. americanum -85.8571243 37.9095336 21000

Amblyomma americanum americanum A. americanum -85.8571243 37.9095336 21000

Amblyomma americanum americanum A. americanum -85.8571243 37.9095336 21000

Amblyomma americanum americanum A. americanum -85.8571243 37.9095336 21000

Amblyomma americanum americanum A. americanum -85.8571243 37.9095336 21000

Amblyomma americanum americanum A. americanum -85.8571243 37.9095336 21000

Amblyomma americanum americanum A. americanum -85.8571243 37.9095336 21000

Amblyomma americanum americanum A. americanum -85.8571243 37.9095336 21000

Amblyomma americanum americanum A. americanum -85.8571243 37.9095336 21000

Amblyomma americanum americanum A. americanum -85.8571243 37.9095336 21000

Amblyomma americanum americanum A. americanum -85.8571243 37.9095336 21000

Amblyomma americanum americanum A. americanum -85.8571243 37.9095336 21000

Amblyomma americanum americanum A. americanum -85.8571243 37.9095336 21000

Amblyomma americanum americanum A. americanum -85.8571243 37.9095336 21000

Amblyomma americanum americanum A. americanum -85.8571243 37.9095336 21000

Amblyomma americanum americanum A. americanum -85.8571243 37.9095336 21000

Amblyomma americanum americanum A. americanum -76.1647796 39.4442798 12000

Amblyomma americanum americanum A. americanum -74.576966 40.024197 8000

Amblyomma americanum americanum A. americanum -77.4568391 38.5520281 15927

Amblyomma americanum americanum A. americanum -77.4568391 38.5520281 15927

Amblyomma americanum americanum A. americanum -76.1647796 39.4442798 12000

Amblyomma americanum americanum A. americanum -76.1647796 39.4442798 12000

Amblyomma americanum americanum A. americanum -77.1691372 40.2075888 1401

Amblyomma americanum americanum A. americanum -86.5295486 30.572647 44515

Amblyomma americanum americanum A. americanum -85.8571243 37.9095336 21000

Amblyomma americanum americanum A. americanum -85.8571243 37.9095336 21000

Amblyomma americanum americanum A. americanum -85.8571243 37.9095336 21000

Amblyomma americanum americanum A. americanum -85.8571243 37.9095336 21000

Amblyomma americanum americanum A. americanum -85.8571243 37.9095336 21000

Amblyomma americanum americanum A. americanum -85.8571243 37.9095336 21000

Amblyomma americanum americanum A. americanum -85.8571243 37.9095336 21000

Amblyomma americanum americanum A. americanum -85.8571243 37.9095336 21000

Amblyomma americanum americanum A. americanum -85.8571243 37.9095336 21000

Amblyomma americanum americanum A. americanum -85.8571243 37.9095336 21000

Amblyomma americanum americanum A. americanum -85.8571243 37.9095336 21000

Amblyomma americanum americanum A. americanum -85.8571243 37.9095336 21000

Amblyomma americanum americanum A. americanum -85.8571243 37.9095336 21000

Amblyomma americanum americanum A. americanum -85.8571243 37.9095336 21000

Amblyomma americanum americanum A. americanum -85.8571243 37.9095336 21000

Amblyomma americanum americanum A. americanum -85.8571243 37.9095336 21000

Amblyomma americanum americanum A. americanum -85.8571243 37.9095336 21000

Amblyomma americanum americanum A. americanum -85.8571243 37.9095336 21000

Amblyomma americanum americanum A. americanum -85.8571243 37.9095336 21000

Amblyomma americanum americanum A. americanum -85.8571243 37.9095336 21000

Amblyomma americanum americanum A. americanum -85.8571243 37.9095336 21000

Amblyomma americanum americanum A. americanum -85.8571243 37.9095336 21000

Amblyomma americanum americanum A. americanum -85.8571243 37.9095336 21000

Amblyomma americanum americanum A. americanum -85.8571243 37.9095336 21000

Amblyomma americanum americanum A. americanum -85.8571243 37.9095336 21000

Amblyomma americanum americanum A. americanum -85.8571243 37.9095336 21000

Amblyomma americanum americanum A. americanum -85.8571243 37.9095336 21000

Amblyomma americanum americanum A. americanum -85.8571243 37.9095336 21000

Amblyomma americanum americanum A. americanum -85.8571243 37.9095336 21000

Amblyomma americanum americanum A. americanum -85.8571243 37.9095336 21000

Amblyomma americanum americanum A. americanum -85.8571243 37.9095336 21000

Amblyomma americanum americanum A. americanum -85.8571243 37.9095336 21000

Amblyomma americanum americanum A. americanum -85.8571243 37.9095336 21000

Amblyomma americanum americanum A. americanum -85.8571243 37.9095336 21000

Amblyomma americanum americanum A. americanum -85.8571243 37.9095336 21000

Amblyomma americanum americanum A. americanum -85.8571243 37.9095336 21000

Amblyomma americanum americanum A. americanum -85.8571243 37.9095336 21000

Amblyomma americanum americanum A. americanum -85.8571243 37.9095336 21000

Amblyomma americanum americanum A. americanum -85.8571243 37.9095336 21000

Amblyomma americanum americanum A. americanum -85.8571243 37.9095336 21000

Amblyomma americanum americanum A. americanum -85.8571243 37.9095336 21000

Amblyomma americanum americanum A. americanum -76.1646169 39.5095539 5104

Amblyomma americanum americanum A. americanum -85.8571243 37.9095336 21000

Amblyomma americanum americanum A. americanum -85.8571243 37.9095336 21000

Amblyomma americanum americanum A. americanum -85.8571243 37.9095336 21000

Amblyomma americanum americanum A. americanum -85.8571243 37.9095336 21000

Amblyomma americanum americanum A. americanum -85.8571243 37.9095336 21000

Amblyomma americanum americanum A. americanum -85.8571243 37.9095336 21000

Amblyomma americanum americanum A. americanum -85.8571243 37.9095336 21000

Amblyomma americanum americanum A. americanum -85.8571243 37.9095336 21000

Amblyomma americanum americanum A. americanum -85.8571243 37.9095336 21000

Amblyomma americanum americanum A. americanum -85.8571243 37.9095336 21000

Amblyomma americanum americanum A. americanum -85.8571243 37.9095336 21000

Amblyomma americanum americanum A. americanum -85.8571243 37.9095336 21000

Amblyomma americanum americanum A. americanum -85.8571243 37.9095336 21000

Amblyomma americanum americanum A. americanum -85.8571243 37.9095336 21000

Amblyomma americanum americanum A. americanum -85.8571243 37.9095336 21000

Amblyomma americanum americanum A. americanum -85.8571243 37.9095336 21000

Amblyomma americanum americanum A. americanum -85.8571243 37.9095336 21000

Amblyomma americanum americanum A. americanum -85.8571243 37.9095336 21000

Amblyomma americanum americanum A. americanum -85.8571243 37.9095336 21000

Amblyomma americanum americanum A. americanum -85.8571243 37.9095336 21000

Amblyomma americanum americanum A. americanum -85.8571243 37.9095336 21000

Amblyomma americanum americanum A. americanum -85.8571243 37.9095336 21000

Amblyomma americanum americanum A. americanum -85.8571243 37.9095336 21000

Amblyomma americanum americanum A. americanum -85.8571243 37.9095336 21000

Amblyomma americanum americanum A. americanum -85.8571243 37.9095336 21000

Amblyomma americanum americanum A. americanum -85.8571243 37.9095336 21000

Amblyomma americanum americanum A. americanum -85.8571243 37.9095336 21000

Amblyomma americanum americanum A. americanum -85.8571243 37.9095336 21000

Amblyomma americanum americanum A. americanum -85.8571243 37.9095336 21000

Amblyomma americanum americanum A. americanum -85.8571243 37.9095336 21000

Amblyomma americanum americanum A. americanum -85.8571243 37.9095336 21000

Amblyomma americanum americanum A. americanum -85.8571243 37.9095336 21000

Amblyomma americanum americanum A. americanum -85.8571243 37.9095336 21000

Amblyomma americanum americanum A. americanum -85.8571243 37.9095336 21000

Amblyomma americanum americanum A. americanum -85.8571243 37.9095336 21000

Amblyomma americanum americanum A. americanum -85.8571243 37.9095336 21000

Amblyomma americanum americanum A. americanum -85.8571243 37.9095336 21000

Amblyomma americanum americanum A. americanum -85.8571243 37.9095336 21000

Amblyomma americanum americanum A. americanum -85.8571243 37.9095336 21000

Amblyomma americanum americanum A. americanum -85.8571243 37.9095336 21000

Amblyomma americanum americanum A. americanum -85.8571243 37.9095336 21000

Amblyomma americanum americanum A. americanum -85.8571243 37.9095336 21000

Amblyomma americanum americanum A. americanum -85.8571243 37.9095336 21000

Amblyomma americanum americanum A. americanum -85.8571243 37.9095336 21000

Amblyomma americanum americanum A. americanum -85.8571243 37.9095336 21000

Amblyomma americanum americanum A. americanum -85.8571243 37.9095336 21000

Amblyomma americanum americanum A. americanum -85.8571243 37.9095336 21000

Amblyomma americanum americanum A. americanum -85.8571243 37.9095336 21000

Amblyomma americanum americanum A. americanum -85.8571243 37.9095336 21000

Amblyomma americanum americanum A. americanum -85.8571243 37.9095336 21000

Amblyomma americanum americanum A. americanum -85.8571243 37.9095336 21000

Amblyomma americanum americanum A. americanum -85.8571243 37.9095336 21000

Amblyomma americanum americanum A. americanum -85.8571243 37.9095336 21000

Amblyomma americanum americanum A. americanum -85.8571243 37.9095336 21000

Amblyomma americanum americanum A. americanum -85.8571243 37.9095336 21000

Amblyomma americanum americanum A. americanum -85.8571243 37.9095336 21000

Amblyomma americanum americanum A. americanum -85.8571243 37.9095336 21000

Amblyomma americanum americanum A. americanum -85.8571243 37.9095336 21000

Amblyomma americanum americanum A. americanum -85.8571243 37.9095336 21000

Amblyomma americanum americanum A. americanum -85.8571243 37.9095336 21000

Amblyomma americanum americanum A. americanum -85.8571243 37.9095336 21000

Amblyomma americanum americanum A. americanum -85.8571243 37.9095336 21000

Amblyomma americanum americanum A. americanum -85.8571243 37.9095336 21000

Amblyomma americanum americanum A. americanum -85.8571243 37.9095336 21000

Amblyomma americanum americanum A. americanum -85.8571243 37.9095336 21000

Amblyomma americanum americanum A. americanum -85.8571243 37.9095336 21000

Amblyomma americanum americanum A. americanum -85.8571243 37.9095336 21000

Amblyomma americanum americanum A. americanum -85.8571243 37.9095336 21000

Amblyomma americanum americanum A. americanum -85.8571243 37.9095336 21000

Amblyomma americanum americanum A. americanum -85.8571243 37.9095336 21000

Amblyomma americanum americanum A. americanum -85.8571243 37.9095336 21000

Amblyomma americanum americanum A. americanum -85.8571243 37.9095336 21000

Amblyomma americanum americanum A. americanum -85.8571243 37.9095336 21000

Amblyomma americanum americanum A. americanum -85.8571243 37.9095336 21000

Amblyomma americanum americanum A. americanum -85.8571243 37.9095336 21000

Amblyomma americanum americanum A. americanum -85.8571243 37.9095336 21000

Amblyomma americanum americanum A. americanum -85.8571243 37.9095336 21000

Amblyomma americanum americanum A. americanum -85.8571243 37.9095336 21000

Amblyomma americanum americanum A. americanum -85.8571243 37.9095336 21000

Amblyomma americanum americanum A. americanum -85.8571243 37.9095336 21000

Amblyomma americanum americanum A. americanum -85.8571243 37.9095336 21000

Amblyomma americanum americanum A. americanum -85.8571243 37.9095336 21000

Amblyomma americanum americanum A. americanum -85.8571243 37.9095336 21000

Amblyomma americanum americanum A. americanum -85.8571243 37.9095336 21000

Amblyomma americanum americanum A. americanum -85.8571243 37.9095336 21000

Amblyomma americanum americanum A. americanum -85.8571243 37.9095336 21000

Amblyomma americanum americanum A. americanum -85.8571243 37.9095336 21000

Amblyomma americanum americanum A. americanum -85.8571243 37.9095336 21000

Amblyomma americanum americanum A. americanum -85.8571243 37.9095336 21000

Amblyomma americanum americanum A. americanum -85.8571243 37.9095336 21000

Amblyomma americanum americanum A. americanum -85.8571243 37.9095336 21000

Amblyomma americanum americanum A. americanum -85.8571243 37.9095336 21000

Amblyomma americanum americanum A. americanum -85.8571243 37.9095336 21000

Amblyomma americanum americanum A. americanum -85.8571243 37.9095336 21000

Amblyomma americanum americanum A. americanum -85.8571243 37.9095336 21000

Amblyomma americanum americanum A. americanum -85.8571243 37.9095336 21000

Amblyomma americanum americanum A. americanum -85.8571243 37.9095336 21000

Amblyomma americanum americanum A. americanum -85.8571243 37.9095336 21000

Amblyomma americanum americanum A. americanum -85.8571243 37.9095336 21000

Amblyomma americanum americanum A. americanum -85.8571243 37.9095336 21000

Amblyomma americanum americanum A. americanum -85.8571243 37.9095336 21000

Amblyomma americanum americanum A. americanum -85.8571243 37.9095336 21000

Amblyomma americanum americanum A. americanum -85.8571243 37.9095336 21000

Amblyomma americanum americanum A. americanum -85.8571243 37.9095336 21000

Amblyomma americanum americanum A. americanum -85.8571243 37.9095336 21000

Amblyomma americanum americanum A. americanum -85.8571243 37.9095336 21000

Amblyomma americanum americanum A. americanum -85.8571243 37.9095336 21000

Amblyomma americanum americanum A. americanum -85.8571243 37.9095336 21000

Amblyomma americanum americanum A. americanum -85.8571243 37.9095336 21000

Amblyomma americanum americanum A. americanum -85.8571243 37.9095336 21000

Amblyomma americanum americanum A. americanum -85.8571243 37.9095336 21000

Amblyomma americanum americanum A. americanum -85.8571243 37.9095336 21000

Amblyomma americanum americanum A. americanum -85.8571243 37.9095336 21000

Amblyomma americanum americanum A. americanum -85.8571243 37.9095336 21000

Amblyomma americanum americanum A. americanum -85.8571243 37.9095336 21000

Amblyomma americanum americanum A. americanum -85.8571243 37.9095336 21000

Amblyomma americanum americanum A. americanum -85.8571243 37.9095336 21000

Amblyomma americanum americanum A. americanum -76.1646169 39.5095539 5104

Amblyomma americanum americanum A. americanum -76.1646169 39.5095539 5104

Amblyomma americanum americanum A. americanum -76.1646169 39.5095539 5104

Amblyomma americanum americanum A. americanum -76.1646169 39.5095539 5104

Amblyomma americanum americanum A. americanum -85.8571243 37.9095336 21000

Amblyomma americanum americanum A. americanum -85.8571243 37.9095336 21000

Amblyomma americanum americanum A. americanum -85.8571243 37.9095336 21000

Amblyomma americanum americanum A. americanum -85.8571243 37.9095336 21000

Amblyomma americanum americanum A. americanum -85.8571243 37.9095336 21000

Amblyomma americanum americanum A. americanum -85.8571243 37.9095336 21000

Amblyomma americanum americanum A. americanum -85.8571243 37.9095336 21000

Amblyomma americanum americanum A. americanum -85.8571243 37.9095336 21000

Amblyomma americanum americanum A. americanum -85.8571243 37.9095336 21000

Amblyomma americanum americanum A. americanum -85.8571243 37.9095336 21000

Amblyomma americanum americanum A. americanum -85.8571243 37.9095336 21000

Amblyomma americanum americanum A. americanum -85.8571243 37.9095336 21000

Amblyomma americanum americanum A. americanum -85.8571243 37.9095336 21000

Amblyomma americanum americanum A. americanum -85.8571243 37.9095336 21000

Amblyomma americanum americanum A. americanum -85.8571243 37.9095336 21000

Amblyomma americanum americanum A. americanum -85.8571243 37.9095336 21000

Amblyomma americanum americanum A. americanum -85.8571243 37.9095336 21000

Amblyomma americanum americanum A. americanum -85.8571243 37.9095336 21000

Amblyomma americanum americanum A. americanum -85.8571243 37.9095336 21000

Amblyomma americanum americanum A. americanum -85.8571243 37.9095336 21000

Amblyomma americanum americanum A. americanum -85.8571243 37.9095336 21000

Amblyomma americanum americanum A. americanum -85.8571243 37.9095336 21000

Amblyomma americanum americanum A. americanum -85.8571243 37.9095336 21000

Amblyomma americanum americanum A. americanum -85.8571243 37.9095336 21000

Amblyomma americanum americanum A. americanum -76.1647796 39.4442798 12000

Amblyomma americanum americanum A. americanum -76.1647796 39.4442798 12000

Amblyomma americanum americanum A. americanum -77.1459102 38.7021234 3500

Amblyomma americanum americanum A. americanum -77.1459102 38.7021234 3500

Amblyomma americanum americanum A. americanum -77.1459102 38.7021234 3500

Amblyomma americanum americanum A. americanum -74.576966 40.024197 8000

Amblyomma americanum americanum A. americanum -74.576966 40.024197 8000

Amblyomma americanum americanum A. americanum -76.882867 38.996036 500

Amblyomma americanum americanum A. americanum -74.1514316 40.2555169 40840

Amblyomma americanum americanum A. americanum -74.1514316 40.2555169 40840

Amblyomma americanum americanum A. americanum -74.1514316 40.2555169 40840

Amblyomma americanum americanum A. americanum -70.521389 41.658611 6000

Amblyomma americanum americanum A. americanum -77.915039 37.0420244 10500

Amblyomma americanum americanum A. americanum -77.915039 37.0420244 10500

Amblyomma americanum americanum A. americanum -77.915039 37.0420244 10500

Amblyomma americanum americanum A. americanum -77.915039 37.0420244 10500

Amblyomma americanum americanum A. americanum -77.915039 37.0420244 10500

Amblyomma americanum americanum A. americanum -77.915039 37.0420244 10500

Amblyomma americanum americanum A. americanum -77.915039 37.0420244 10500

Amblyomma americanum americanum A. americanum -77.915039 37.0420244 10500

Amblyomma americanum americanum A. americanum -77.915039 37.0420244 10500

Amblyomma americanum americanum A. americanum -77.915039 37.0420244 10500

Amblyomma americanum americanum A. americanum -77.915039 37.0420244 10500

Amblyomma americanum americanum A. americanum -77.915039 37.0420244 10500

Amblyomma americanum americanum A. americanum -77.915039 37.0420244 10500

Amblyomma americanum americanum A. americanum -77.915039 37.0420244 10500

Amblyomma americanum americanum A. americanum -77.915039 37.0420244 10500

Amblyomma americanum americanum A. americanum -77.915039 37.0420244 10500

Amblyomma americanum americanum A. americanum -77.915039 37.0420244 10500

Amblyomma americanum americanum A. americanum -77.915039 37.0420244 10500

Amblyomma americanum americanum A. americanum -77.915039 37.0420244 10500

Amblyomma americanum americanum A. americanum -77.915039 37.0420244 10500

Amblyomma americanum americanum A. americanum -77.915039 37.0420244 10500

Amblyomma americanum americanum A. americanum -77.915039 37.0420244 10500

Amblyomma americanum americanum A. americanum -77.915039 37.0420244 10500

Amblyomma americanum americanum A. americanum -77.915039 37.0420244 10500

Amblyomma americanum americanum A. americanum -77.915039 37.0420244 10500

Amblyomma americanum americanum A. americanum -77.915039 37.0420244 10500

Amblyomma americanum americanum A. americanum -77.915039 37.0420244 10500

Amblyomma americanum americanum A. americanum -77.915039 37.0420244 10500

Amblyomma americanum americanum A. americanum -77.915039 37.0420244 10500

Amblyomma americanum americanum A. americanum -77.915039 37.0420244 10500

Amblyomma americanum americanum A. americanum -77.915039 37.0420244 10500

Amblyomma americanum americanum A. americanum -77.915039 37.0420244 10500

Amblyomma americanum americanum A. americanum -77.915039 37.0420244 10500

Amblyomma americanum americanum A. americanum -77.915039 37.0420244 10500

Amblyomma americanum americanum A. americanum -77.915039 37.0420244 10500

Amblyomma americanum americanum A. americanum -77.915039 37.0420244 10500

Amblyomma americanum americanum A. americanum -77.915039 37.0420244 10500

Amblyomma americanum americanum A. americanum -77.915039 37.0420244 10500

Amblyomma americanum americanum A. americanum -77.915039 37.0420244 10500

Amblyomma americanum americanum A. americanum -77.915039 37.0420244 10500

Amblyomma americanum americanum A. americanum -77.915039 37.0420244 10500

Amblyomma americanum americanum A. americanum -77.915039 37.0420244 10500

Amblyomma americanum americanum A. americanum -77.915039 37.0420244 10500

Amblyomma americanum americanum A. americanum -77.915039 37.0420244 10500

Amblyomma americanum americanum A. americanum -77.915039 37.0420244 10500

Amblyomma americanum americanum A. americanum -77.915039 37.0420244 10500

Amblyomma americanum americanum A. americanum -77.915039 37.0420244 10500

Amblyomma americanum americanum A. americanum -77.915039 37.0420244 10500

Amblyomma americanum americanum A. americanum -77.915039 37.0420244 10500

Amblyomma americanum americanum A. americanum -77.915039 37.0420244 10500

Amblyomma americanum americanum A. americanum -77.915039 37.0420244 10500

Amblyomma americanum americanum A. americanum -77.915039 37.0420244 10500

Amblyomma americanum americanum A. americanum -77.915039 37.0420244 10500

Amblyomma americanum americanum A. americanum -77.915039 37.0420244 10500

Amblyomma americanum americanum A. americanum -77.915039 37.0420244 10500

Amblyomma americanum americanum A. americanum -77.915039 37.0420244 10500

Amblyomma americanum americanum A. americanum -77.915039 37.0420244 10500

Amblyomma americanum americanum A. americanum -77.915039 37.0420244 10500

Amblyomma americanum americanum A. americanum -77.915039 37.0420244 10500

Amblyomma americanum americanum A. americanum -77.915039 37.0420244 10500

Amblyomma americanum americanum A. americanum -77.915039 37.0420244 10500

Amblyomma americanum americanum A. americanum -77.915039 37.0420244 10500

Amblyomma americanum americanum A. americanum -77.915039 37.0420244 10500

Amblyomma americanum americanum A. americanum -77.915039 37.0420244 10500

Amblyomma americanum americanum A. americanum -77.915039 37.0420244 10500

Amblyomma americanum americanum A. americanum -77.915039 37.0420244 10500

Amblyomma americanum americanum A. americanum -77.915039 37.0420244 10500

Amblyomma americanum americanum A. americanum -77.915039 37.0420244 10500

Amblyomma americanum americanum A. americanum -77.915039 37.0420244 10500

Amblyomma americanum americanum A. americanum -77.915039 37.0420244 10500

Amblyomma americanum americanum A. americanum -77.915039 37.0420244 10500

Amblyomma americanum americanum A. americanum -77.915039 37.0420244 10500

Amblyomma americanum americanum A. americanum -77.915039 37.0420244 10500

Amblyomma americanum americanum A. americanum -77.915039 37.0420244 10500

Amblyomma americanum americanum A. americanum -77.915039 37.0420244 10500

Amblyomma americanum americanum A. americanum -77.915039 37.0420244 10500

Amblyomma americanum americanum A. americanum -77.915039 37.0420244 10500

Amblyomma americanum americanum A. americanum -92.1572583 37.7057025 16307

Amblyomma americanum americanum A. americanum -76.1647796 39.4442798 12000

Amblyomma americanum americanum A. americanum -76.1647796 39.4442798 12000

Amblyomma americanum americanum A. americanum -76.1647796 39.4442798 12000

Amblyomma americanum americanum A. americanum -74.1514316 40.2555169 40840

Amblyomma americanum americanum A. americanum -71.3191935 41.5173901 5851

Amblyomma americanum americanum A. americanum -77.915039 37.0420244 10500

Amblyomma americanum americanum A. americanum -77.915039 37.0420244 10500

Amblyomma americanum americanum A. americanum -77.915039 37.0420244 10500

Amblyomma americanum americanum A. americanum -77.915039 37.0420244 10500

Amblyomma americanum americanum A. americanum -77.915039 37.0420244 10500

Amblyomma americanum americanum A. americanum -77.915039 37.0420244 10500

Amblyomma americanum americanum A. americanum -77.915039 37.0420244 10500

Amblyomma americanum americanum A. americanum -77.915039 37.0420244 10500

Amblyomma americanum americanum A. americanum -77.915039 37.0420244 10500

Amblyomma americanum americanum A. americanum -77.915039 37.0420244 10500

Amblyomma americanum americanum A. americanum -77.915039 37.0420244 10500

Amblyomma americanum americanum A. americanum -77.915039 37.0420244 10500

Amblyomma americanum americanum A. americanum -77.915039 37.0420244 10500

Amblyomma americanum americanum A. americanum -77.915039 37.0420244 10500

Amblyomma americanum americanum A. americanum -77.915039 37.0420244 10500

Amblyomma americanum americanum A. americanum -77.915039 37.0420244 10500

Amblyomma americanum americanum A. americanum -77.915039 37.0420244 10500

Amblyomma americanum americanum A. americanum -77.915039 37.0420244 10500

Amblyomma americanum americanum A. americanum -77.915039 37.0420244 10500

Amblyomma americanum americanum A. americanum -77.915039 37.0420244 10500

Amblyomma americanum americanum A. americanum -77.915039 37.0420244 10500

Amblyomma americanum americanum A. americanum -77.915039 37.0420244 10500

Amblyomma americanum americanum A. americanum -77.915039 37.0420244 10500

Amblyomma americanum americanum A. americanum -77.915039 37.0420244 10500

Amblyomma americanum americanum A. americanum -77.915039 37.0420244 10500

Amblyomma americanum americanum A. americanum -77.915039 37.0420244 10500

Amblyomma americanum americanum A. americanum -77.915039 37.0420244 10500

Amblyomma americanum americanum A. americanum -77.915039 37.0420244 10500

Amblyomma americanum americanum A. americanum -77.915039 37.0420244 10500

Amblyomma americanum americanum A. americanum -77.915039 37.0420244 10500

Amblyomma americanum americanum A. americanum -77.915039 37.0420244 10500

Amblyomma americanum americanum A. americanum -77.915039 37.0420244 10500

Amblyomma americanum americanum A. americanum -77.915039 37.0420244 10500

Amblyomma americanum americanum A. americanum -77.915039 37.0420244 10500

Amblyomma americanum americanum A. americanum -77.915039 37.0420244 10500

Amblyomma americanum americanum A. americanum -77.915039 37.0420244 10500

Amblyomma americanum americanum A. americanum -77.915039 37.0420244 10500

Amblyomma americanum americanum A. americanum -77.915039 37.0420244 10500

Amblyomma americanum americanum A. americanum -77.915039 37.0420244 10500

Amblyomma americanum americanum A. americanum -77.915039 37.0420244 10500

Amblyomma americanum americanum A. americanum -77.915039 37.0420244 10500

Amblyomma americanum americanum A. americanum -77.915039 37.0420244 10500

Amblyomma americanum americanum A. americanum -77.915039 37.0420244 10500

Amblyomma americanum americanum A. americanum -77.915039 37.0420244 10500

Amblyomma americanum americanum A. americanum -77.915039 37.0420244 10500

Amblyomma americanum americanum A. americanum -77.915039 37.0420244 10500

Amblyomma americanum americanum A. americanum -77.915039 37.0420244 10500

Amblyomma americanum americanum A. americanum -77.915039 37.0420244 10500

Amblyomma americanum americanum A. americanum -79.070374 35.654428 2000

Amblyomma americanum americanum A. americanum -77.975979 34.007135 5000

Amblyomma americanum americanum A. americanum -76.1647796 39.4442798 12000

Amblyomma americanum americanum A. americanum -76.1647796 39.4442798 12000

Amblyomma americanum americanum A. americanum -76.1647796 39.4442798 12000

Amblyomma americanum americanum A. americanum -76.1647796 39.4442798 12000

Amblyomma americanum americanum A. americanum -76.7734909 39.0642483 10000

Amblyomma americanum americanum A. americanum -76.7734909 39.0642483 10000

Amblyomma americanum americanum A. americanum -76.7734909 39.0642483 10000

Amblyomma americanum americanum A. americanum -76.7734909 39.0642483 10000

Amblyomma americanum americanum A. americanum -76.7734909 39.0642483 10000

Amblyomma americanum americanum A. americanum -77.4568391 38.5520281 15927

Amblyomma americanum americanum A. americanum -76.1647796 39.4442798 12000

Amblyomma americanum americanum A. americanum -76.1647796 39.4442798 12000

Amblyomma americanum americanum A. americanum -76.1647796 39.4442798 12000

Amblyomma americanum americanum A. americanum -76.1647796 39.4442798 12000

Amblyomma americanum americanum A. americanum -76.1647796 39.4442798 12000

Amblyomma americanum americanum A. americanum -76.1647796 39.4442798 12000

Amblyomma americanum americanum A. americanum -74.576966 40.024197 8000

Amblyomma americanum americanum A. americanum -76.1647796 39.4442798 12000

Amblyomma americanum americanum A. americanum -76.1647796 39.4442798 12000

Amblyomma americanum americanum A. americanum -74.576966 40.024197 8000

Amblyomma americanum americanum A. americanum -74.1514316 40.2555169 40840

Amblyomma americanum americanum A. americanum -74.1514316 40.2555169 40840

Amblyomma americanum americanum A. americanum -74.1514316 40.2555169 40840

Amblyomma americanum americanum A. americanum -76.1647796 39.4442798 12000

Amblyomma americanum americanum A. americanum -76.1647796 39.4442798 12000

Amblyomma americanum americanum A. americanum -76.1647796 39.4442798 12000

Amblyomma americanum americanum A. americanum -92.23117 34.827169 100

Amblyomma americanum americanum A. americanum -87.6159667 36.6078112 18500

Amblyomma americanum americanum A. americanum -122.578636 47.100278 12000

Amblyomma americanum americanum A. americanum -76.1647796 39.4442798 12000

Amblyomma americanum americanum A. americanum -76.1647796 39.4442798 12000

Amblyomma americanum americanum A. americanum -74.576966 40.024197 8000

Amblyomma americanum americanum A. americanum -85.8571243 37.9095336 21000

Amblyomma americanum americanum A. americanum -85.8571243 37.9095336 21000

Amblyomma americanum americanum A. americanum -85.8571243 37.9095336 21000

Amblyomma americanum americanum A. americanum -85.8571243 37.9095336 21000

Amblyomma americanum americanum A. americanum -85.8571243 37.9095336 21000

Amblyomma americanum americanum A. americanum -85.8571243 37.9095336 21000

Amblyomma americanum americanum A. americanum -85.8571243 37.9095336 21000

Amblyomma americanum americanum A. americanum -85.8571243 37.9095336 21000

Amblyomma americanum americanum A. americanum -85.8571243 37.9095336 21000

Amblyomma americanum americanum A. americanum -85.8571243 37.9095336 21000

Amblyomma americanum americanum A. americanum -85.8571243 37.9095336 21000

Amblyomma americanum americanum A. americanum -85.8571243 37.9095336 21000

Amblyomma americanum americanum A. americanum -85.8571243 37.9095336 21000

Amblyomma americanum americanum A. americanum -85.8571243 37.9095336 21000

Amblyomma americanum americanum A. americanum -85.8571243 37.9095336 21000

Amblyomma americanum americanum A. americanum -85.8571243 37.9095336 21000

Amblyomma americanum americanum A. americanum -85.8571243 37.9095336 21000

Amblyomma americanum americanum A. americanum -85.8571243 37.9095336 21000

Amblyomma americanum americanum A. americanum -85.8571243 37.9095336 21000

Amblyomma americanum americanum A. americanum -85.8571243 37.9095336 21000

Amblyomma americanum americanum A. americanum -85.8571243 37.9095336 21000

Amblyomma americanum americanum A. americanum -85.8571243 37.9095336 21000

Amblyomma americanum americanum A. americanum -85.8571243 37.9095336 21000

Amblyomma americanum americanum A. americanum -85.8571243 37.9095336 21000

Amblyomma americanum americanum A. americanum -85.8571243 37.9095336 21000

Amblyomma americanum americanum A. americanum -85.8571243 37.9095336 21000

Amblyomma americanum americanum A. americanum -85.8571243 37.9095336 21000

Amblyomma americanum americanum A. americanum -85.8571243 37.9095336 21000

Amblyomma americanum americanum A. americanum -85.8571243 37.9095336 21000

Amblyomma americanum americanum A. americanum -85.8571243 37.9095336 21000

Amblyomma americanum americanum A. americanum -85.8571243 37.9095336 21000

Amblyomma americanum americanum A. americanum -85.8571243 37.9095336 21000

Amblyomma americanum americanum A. americanum -85.8571243 37.9095336 21000

Amblyomma americanum americanum A. americanum -85.8571243 37.9095336 21000

Amblyomma americanum americanum A. americanum -85.8571243 37.9095336 21000

Amblyomma americanum americanum A. americanum -85.8571243 37.9095336 21000

Amblyomma americanum americanum A. americanum -85.8571243 37.9095336 21000

Amblyomma americanum americanum A. americanum -85.8571243 37.9095336 21000

Amblyomma americanum americanum A. americanum -85.8571243 37.9095336 21000

Amblyomma americanum americanum A. americanum -85.8571243 37.9095336 21000

Amblyomma americanum americanum A. americanum -85.8571243 37.9095336 21000

Amblyomma americanum americanum A. americanum -85.8571243 37.9095336 21000

Amblyomma americanum americanum A. americanum -85.8571243 37.9095336 21000

Amblyomma americanum americanum A. americanum -85.8571243 37.9095336 21000

Amblyomma americanum americanum A. americanum -85.8571243 37.9095336 21000

Amblyomma americanum americanum A. americanum -85.8571243 37.9095336 21000

Amblyomma americanum americanum A. americanum -85.8571243 37.9095336 21000

Amblyomma americanum americanum A. americanum -85.8571243 37.9095336 21000

Amblyomma americanum americanum A. americanum -85.8571243 37.9095336 21000

Amblyomma americanum americanum A. americanum -85.8571243 37.9095336 21000

Amblyomma americanum americanum A. americanum -85.8571243 37.9095336 21000

Amblyomma americanum americanum A. americanum -85.8571243 37.9095336 21000

Amblyomma americanum americanum A. americanum -85.8571243 37.9095336 21000

Amblyomma americanum americanum A. americanum -85.8571243 37.9095336 21000

Amblyomma americanum americanum A. americanum -85.8571243 37.9095336 21000

Amblyomma americanum americanum A. americanum -85.8571243 37.9095336 21000

Amblyomma americanum americanum A. americanum -85.8571243 37.9095336 21000

Amblyomma americanum americanum A. americanum -85.8571243 37.9095336 21000

Amblyomma americanum americanum A. americanum -85.8571243 37.9095336 21000

Amblyomma americanum americanum A. americanum -85.8571243 37.9095336 21000

Amblyomma americanum americanum A. americanum -85.8571243 37.9095336 21000

Amblyomma americanum americanum A. americanum -85.8571243 37.9095336 21000

Amblyomma americanum americanum A. americanum -76.84515 39.035445 2000

Amblyomma americanum americanum A. americanum -87.6159667 36.6078112 18500

Amblyomma americanum americanum A. americanum -85.8571243 37.9095336 21000

Amblyomma americanum americanum A. americanum -85.8571243 37.9095336 21000

Amblyomma americanum americanum A. americanum -85.8571243 37.9095336 21000

Amblyomma americanum americanum A. americanum -85.8571243 37.9095336 21000

Amblyomma americanum americanum A. americanum -85.8571243 37.9095336 21000

Amblyomma americanum americanum A. americanum -85.8571243 37.9095336 21000

Amblyomma americanum americanum A. americanum -85.8571243 37.9095336 21000

Amblyomma americanum americanum A. americanum -85.8571243 37.9095336 21000

Amblyomma americanum americanum A. americanum -85.8571243 37.9095336 21000

Amblyomma americanum americanum A. americanum -85.8571243 37.9095336 21000

Amblyomma americanum americanum A. americanum -85.8571243 37.9095336 21000

Amblyomma americanum americanum A. americanum -85.8571243 37.9095336 21000

Amblyomma americanum americanum A. americanum -85.8571243 37.9095336 21000

Amblyomma americanum americanum A. americanum -85.8571243 37.9095336 21000

Amblyomma americanum americanum A. americanum -85.8571243 37.9095336 21000

Amblyomma americanum americanum A. americanum -85.8571243 37.9095336 21000

Amblyomma americanum americanum A. americanum -85.8571243 37.9095336 21000

Amblyomma americanum americanum A. americanum -85.8571243 37.9095336 21000

Amblyomma americanum americanum A. americanum -85.8571243 37.9095336 21000

Amblyomma americanum americanum A. americanum -85.8571243 37.9095336 21000

Amblyomma americanum americanum A. americanum -85.8571243 37.9095336 21000

Amblyomma americanum americanum A. americanum -85.8571243 37.9095336 21000

Amblyomma americanum americanum A. americanum -85.8571243 37.9095336 21000

Amblyomma americanum americanum A. americanum -85.8571243 37.9095336 21000

Amblyomma americanum americanum A. americanum -85.8571243 37.9095336 21000

Amblyomma americanum americanum A. americanum -85.8571243 37.9095336 21000

Amblyomma americanum americanum A. americanum -85.8571243 37.9095336 21000

Amblyomma americanum americanum A. americanum -85.8571243 37.9095336 21000

Amblyomma americanum americanum A. americanum -85.8571243 37.9095336 21000

Amblyomma americanum americanum A. americanum -85.8571243 37.9095336 21000

Amblyomma americanum americanum A. americanum -85.8571243 37.9095336 21000

Amblyomma americanum americanum A. americanum -85.8571243 37.9095336 21000

Amblyomma americanum americanum A. americanum -85.8571243 37.9095336 21000

Amblyomma americanum americanum A. americanum -85.8571243 37.9095336 21000

Amblyomma americanum americanum A. americanum -85.8571243 37.9095336 21000

Amblyomma americanum americanum A. americanum -85.8571243 37.9095336 21000

Amblyomma americanum americanum A. americanum -85.8571243 37.9095336 21000

Amblyomma americanum americanum A. americanum -85.8571243 37.9095336 21000

Amblyomma americanum americanum A. americanum -85.8571243 37.9095336 21000

Amblyomma americanum americanum A. americanum -85.8571243 37.9095336 21000

Amblyomma americanum americanum A. americanum -85.8571243 37.9095336 21000

Amblyomma americanum americanum A. americanum -85.8571243 37.9095336 21000

Amblyomma americanum americanum A. americanum -85.8571243 37.9095336 21000

Amblyomma americanum americanum A. americanum -94.9129486 39.3571307 6500

Amblyomma americanum americanum A. americanum -96.8206687 39.1865859 20922

Amblyomma americanum americanum A. americanum -92.1572583 37.7057025 16307

Amblyomma americanum americanum A. americanum -76.576111 40.436944 6000

Amblyomma americanum americanum A. americanum -76.576111 40.436944 6000

Amblyomma americanum americanum A. americanum -122.578636 47.100278 12000

Amblyomma americanum americanum A. americanum -85.8571243 37.9095336 21000

Amblyomma americanum americanum A. americanum -85.8571243 37.9095336 21000

Amblyomma americanum americanum A. americanum -85.8571243 37.9095336 21000

Amblyomma americanum americanum A. americanum -85.8571243 37.9095336 21000

Amblyomma americanum americanum A. americanum -85.8571243 37.9095336 21000

Amblyomma americanum americanum A. americanum -85.8571243 37.9095336 21000

Amblyomma americanum americanum A. americanum -85.8571243 37.9095336 21000

Amblyomma americanum americanum A. americanum -85.8571243 37.9095336 21000

Amblyomma americanum americanum A. americanum -85.8571243 37.9095336 21000

Amblyomma americanum americanum A. americanum -85.8571243 37.9095336 21000

Amblyomma americanum americanum A. americanum -85.8571243 37.9095336 21000

Amblyomma americanum americanum A. americanum -85.8571243 37.9095336 21000

Amblyomma americanum americanum A. americanum -85.8571243 37.9095336 21000

Amblyomma americanum americanum A. americanum -85.8571243 37.9095336 21000

Amblyomma americanum americanum A. americanum -85.8571243 37.9095336 21000

Amblyomma americanum americanum A. americanum -85.8571243 37.9095336 21000

Amblyomma americanum americanum A. americanum -85.8571243 37.9095336 21000

Amblyomma americanum americanum A. americanum -85.8571243 37.9095336 21000

Amblyomma americanum americanum A. americanum -85.8571243 37.9095336 21000

Amblyomma americanum americanum A. americanum -85.8571243 37.9095336 21000

Amblyomma americanum americanum A. americanum -85.8571243 37.9095336 21000

Amblyomma americanum americanum A. americanum -85.8571243 37.9095336 21000

Amblyomma americanum americanum A. americanum -85.8571243 37.9095336 21000

Amblyomma americanum americanum A. americanum -85.8571243 37.9095336 21000

Amblyomma americanum americanum A. americanum -85.8571243 37.9095336 21000

Amblyomma americanum americanum A. americanum -85.8571243 37.9095336 21000

Amblyomma americanum americanum A. americanum -85.8571243 37.9095336 21000

Amblyomma americanum americanum A. americanum -85.8571243 37.9095336 21000

Amblyomma americanum americanum A. americanum -85.8571243 37.9095336 21000

Amblyomma americanum americanum A. americanum -85.8571243 37.9095336 21000

Amblyomma americanum americanum A. americanum -85.8571243 37.9095336 21000

Amblyomma americanum americanum A. americanum -85.8571243 37.9095336 21000

Amblyomma americanum americanum A. americanum -85.8571243 37.9095336 21000

Amblyomma americanum americanum A. americanum -85.8571243 37.9095336 21000

Amblyomma americanum americanum A. americanum -85.8571243 37.9095336 21000

Amblyomma americanum americanum A. americanum -85.8571243 37.9095336 21000

Amblyomma americanum americanum A. americanum -85.8571243 37.9095336 21000

Amblyomma americanum americanum A. americanum -85.8571243 37.9095336 21000

Amblyomma americanum americanum A. americanum -85.8571243 37.9095336 21000

Amblyomma americanum americanum A. americanum -85.8571243 37.9095336 21000

Amblyomma americanum americanum A. americanum -85.8571243 37.9095336 21000

Amblyomma americanum americanum A. americanum -85.8571243 37.9095336 21000

Amblyomma americanum americanum A. americanum -85.8571243 37.9095336 21000

Amblyomma americanum americanum A. americanum -85.8571243 37.9095336 21000

Amblyomma americanum americanum A. americanum -85.8571243 37.9095336 21000

Amblyomma americanum americanum A. americanum -85.8571243 37.9095336 21000

Amblyomma americanum americanum A. americanum -85.8571243 37.9095336 21000

Amblyomma americanum americanum A. americanum -85.8571243 37.9095336 21000

Amblyomma americanum americanum A. americanum -85.8571243 37.9095336 21000

Amblyomma americanum americanum A. americanum -85.8571243 37.9095336 21000

Amblyomma americanum americanum A. americanum -85.8571243 37.9095336 21000

Amblyomma americanum americanum A. americanum -85.8571243 37.9095336 21000

Amblyomma americanum americanum A. americanum -85.8571243 37.9095336 21000

Amblyomma americanum americanum A. americanum -85.8571243 37.9095336 21000

Amblyomma americanum americanum A. americanum -76.1647796 39.4442798 12000

Amblyomma americanum americanum A. americanum -86.5295486 30.572647 44515

Amblyomma americanum americanum A. americanum -85.8571243 37.9095336 21000

Amblyomma americanum americanum A. americanum -85.8571243 37.9095336 21000

Amblyomma americanum americanum A. americanum -85.8571243 37.9095336 21000

Amblyomma americanum americanum A. americanum -85.8571243 37.9095336 21000

Amblyomma americanum americanum A. americanum -85.8571243 37.9095336 21000

Amblyomma americanum americanum A. americanum -85.8571243 37.9095336 21000

Amblyomma americanum americanum A. americanum -85.8571243 37.9095336 21000

Amblyomma americanum americanum A. americanum -85.8571243 37.9095336 21000

Amblyomma americanum americanum A. americanum -85.8571243 37.9095336 21000

Amblyomma americanum americanum A. americanum -85.8571243 37.9095336 21000

Amblyomma americanum americanum A. americanum -85.8571243 37.9095336 21000

Amblyomma americanum americanum A. americanum -85.8571243 37.9095336 21000

Amblyomma americanum americanum A. americanum -85.8571243 37.9095336 21000

Amblyomma americanum americanum A. americanum -85.8571243 37.9095336 21000

Amblyomma americanum americanum A. americanum -85.8571243 37.9095336 21000

Amblyomma americanum americanum A. americanum -85.8571243 37.9095336 21000

Amblyomma americanum americanum A. americanum -85.8571243 37.9095336 21000

Amblyomma americanum americanum A. americanum -85.8571243 37.9095336 21000

Amblyomma americanum americanum A. americanum -85.8571243 37.9095336 21000

Amblyomma americanum americanum A. americanum -85.8571243 37.9095336 21000

Amblyomma americanum americanum A. americanum -85.8571243 37.9095336 21000

Amblyomma americanum americanum A. americanum -85.8571243 37.9095336 21000

Amblyomma americanum americanum A. americanum -85.8571243 37.9095336 21000

Amblyomma americanum americanum A. americanum -85.8571243 37.9095336 21000

Amblyomma americanum americanum A. americanum -85.8571243 37.9095336 21000

Amblyomma americanum americanum A. americanum -85.8571243 37.9095336 21000

Amblyomma americanum americanum A. americanum -85.8571243 37.9095336 21000

Amblyomma americanum americanum A. americanum -85.8571243 37.9095336 21000

Amblyomma americanum americanum A. americanum -85.8571243 37.9095336 21000

Amblyomma americanum americanum A. americanum -85.8571243 37.9095336 21000

Amblyomma americanum americanum A. americanum -85.8571243 37.9095336 21000

Amblyomma americanum americanum A. americanum -85.8571243 37.9095336 21000

Amblyomma americanum americanum A. americanum -85.8571243 37.9095336 21000

Amblyomma americanum americanum A. americanum -85.8571243 37.9095336 21000

Amblyomma americanum americanum A. americanum -85.8571243 37.9095336 21000

Amblyomma americanum americanum A. americanum -85.8571243 37.9095336 21000

Amblyomma americanum americanum A. americanum -85.8571243 37.9095336 21000

Amblyomma americanum americanum A. americanum -85.8571243 37.9095336 21000

Amblyomma americanum americanum A. americanum -85.8571243 37.9095336 21000

Amblyomma americanum americanum A. americanum -85.8571243 37.9095336 21000

Amblyomma americanum americanum A. americanum -85.8571243 37.9095336 21000

Amblyomma americanum americanum A. americanum -85.8571243 37.9095336 21000

Amblyomma americanum americanum A. americanum -85.8571243 37.9095336 21000

Amblyomma americanum americanum A. americanum -85.8571243 37.9095336 21000

Amblyomma americanum americanum A. americanum -85.8571243 37.9095336 21000

Amblyomma americanum americanum A. americanum -85.8571243 37.9095336 21000

Amblyomma americanum americanum A. americanum -85.8571243 37.9095336 21000

Amblyomma americanum americanum A. americanum -85.8571243 37.9095336 21000

Amblyomma americanum americanum A. americanum -85.8571243 37.9095336 21000

Amblyomma americanum americanum A. americanum -85.8571243 37.9095336 21000

Amblyomma americanum americanum A. americanum -85.8571243 37.9095336 21000

Amblyomma americanum americanum A. americanum -85.8571243 37.9095336 21000

Amblyomma americanum americanum A. americanum -85.8571243 37.9095336 21000

Amblyomma americanum americanum A. americanum -85.8571243 37.9095336 21000

Amblyomma americanum americanum A. americanum -85.8571243 37.9095336 21000

Amblyomma americanum americanum A. americanum -85.8571243 37.9095336 21000

Amblyomma americanum americanum A. americanum -85.8571243 37.9095336 21000

Amblyomma americanum americanum A. americanum -85.8571243 37.9095336 21000

Amblyomma americanum americanum A. americanum -85.8571243 37.9095336 21000

Amblyomma americanum americanum A. americanum -85.8571243 37.9095336 21000

Amblyomma americanum americanum A. americanum -85.8571243 37.9095336 21000

Amblyomma americanum americanum A. americanum -85.8571243 37.9095336 21000

Amblyomma americanum americanum A. americanum -85.8571243 37.9095336 21000

Amblyomma americanum americanum A. americanum -85.8571243 37.9095336 21000

Amblyomma americanum americanum A. americanum -85.8571243 37.9095336 21000

Amblyomma americanum americanum A. americanum -85.8571243 37.9095336 21000

Amblyomma americanum americanum A. americanum -85.8571243 37.9095336 21000

Amblyomma americanum americanum A. americanum -85.8571243 37.9095336 21000

Amblyomma americanum americanum A. americanum -85.8571243 37.9095336 21000

Amblyomma americanum americanum A. americanum -85.8571243 37.9095336 21000

Amblyomma americanum americanum A. americanum -92.1572583 37.7057025 16307

Amblyomma americanum americanum A. americanum -85.8571243 37.9095336 21000

Amblyomma americanum americanum A. americanum -85.8571243 37.9095336 21000

Amblyomma americanum americanum A. americanum -85.8571243 37.9095336 21000

Amblyomma americanum americanum A. americanum -85.8571243 37.9095336 21000

Amblyomma americanum americanum A. americanum -85.8571243 37.9095336 21000

Amblyomma americanum americanum A. americanum -85.8571243 37.9095336 21000

Amblyomma americanum americanum A. americanum -85.8571243 37.9095336 21000

Amblyomma americanum americanum A. americanum -85.8571243 37.9095336 21000

Amblyomma americanum americanum A. americanum -85.8571243 37.9095336 21000

Amblyomma americanum americanum A. americanum -85.8571243 37.9095336 21000

Amblyomma americanum americanum A. americanum -85.8571243 37.9095336 21000

Amblyomma americanum americanum A. americanum -85.8571243 37.9095336 21000

Amblyomma americanum americanum A. americanum -85.8571243 37.9095336 21000

Amblyomma americanum americanum A. americanum -85.8571243 37.9095336 21000

Amblyomma americanum americanum A. americanum -85.8571243 37.9095336 21000

Amblyomma americanum americanum A. americanum -85.8571243 37.9095336 21000

Amblyomma americanum americanum A. americanum -85.8571243 37.9095336 21000

Amblyomma americanum americanum A. americanum -85.8571243 37.9095336 21000

Amblyomma americanum americanum A. americanum -85.8571243 37.9095336 21000

Amblyomma americanum americanum A. americanum -85.8571243 37.9095336 21000

Amblyomma americanum americanum A. americanum -85.8571243 37.9095336 21000

Amblyomma americanum americanum A. americanum -85.8571243 37.9095336 21000

Amblyomma americanum americanum A. americanum -85.8571243 37.9095336 21000

Amblyomma americanum americanum A. americanum -85.8571243 37.9095336 21000

Amblyomma americanum americanum A. americanum -85.8571243 37.9095336 21000

Amblyomma americanum americanum A. americanum -85.8571243 37.9095336 21000

Amblyomma americanum americanum A. americanum -85.8571243 37.9095336 21000

Amblyomma americanum americanum A. americanum -85.8571243 37.9095336 21000

Amblyomma americanum americanum A. americanum -85.8571243 37.9095336 21000

Amblyomma americanum americanum A. americanum -85.8571243 37.9095336 21000

Amblyomma americanum americanum A. americanum -85.8571243 37.9095336 21000

Amblyomma americanum americanum A. americanum -85.8571243 37.9095336 21000

Amblyomma americanum americanum A. americanum -85.8571243 37.9095336 21000

Amblyomma americanum americanum A. americanum -85.8571243 37.9095336 21000

Amblyomma americanum americanum A. americanum -85.8571243 37.9095336 21000

Amblyomma americanum americanum A. americanum -85.8571243 37.9095336 21000

Amblyomma americanum americanum A. americanum -85.8571243 37.9095336 21000

Amblyomma americanum americanum A. americanum -85.8571243 37.9095336 21000

Amblyomma americanum americanum A. americanum -85.8571243 37.9095336 21000

Amblyomma americanum americanum A. americanum -85.8571243 37.9095336 21000

Amblyomma americanum americanum A. americanum -85.8571243 37.9095336 21000

Amblyomma americanum americanum A. americanum -85.8571243 37.9095336 21000

Amblyomma americanum americanum A. americanum -85.8571243 37.9095336 21000

Amblyomma americanum americanum A. americanum -85.8571243 37.9095336 21000

Amblyomma americanum americanum A. americanum -85.8571243 37.9095336 21000

Amblyomma americanum americanum A. americanum -85.8571243 37.9095336 21000

Amblyomma americanum americanum A. americanum -85.8571243 37.9095336 21000

Amblyomma americanum americanum A. americanum -85.8571243 37.9095336 21000

Amblyomma americanum americanum A. americanum -85.8571243 37.9095336 21000

Amblyomma americanum americanum A. americanum -85.8571243 37.9095336 21000

Amblyomma americanum americanum A. americanum -85.8571243 37.9095336 21000

Amblyomma americanum americanum A. americanum -85.8571243 37.9095336 21000

Amblyomma americanum americanum A. americanum -85.8571243 37.9095336 21000

Amblyomma americanum americanum A. americanum -85.8571243 37.9095336 21000

Amblyomma americanum americanum A. americanum -85.8571243 37.9095336 21000

Amblyomma americanum americanum A. americanum -85.8571243 37.9095336 21000

Amblyomma americanum americanum A. americanum -85.8571243 37.9095336 21000

Amblyomma americanum americanum A. americanum -85.8571243 37.9095336 21000

Amblyomma americanum americanum A. americanum -85.8571243 37.9095336 21000

Amblyomma americanum americanum A. americanum -85.8571243 37.9095336 21000

Amblyomma americanum americanum A. americanum -85.8571243 37.9095336 21000

Amblyomma americanum americanum A. americanum -85.8571243 37.9095336 21000

Amblyomma americanum americanum A. americanum -85.8571243 37.9095336 21000

Amblyomma americanum americanum A. americanum -85.8571243 37.9095336 21000

Amblyomma americanum americanum A. americanum -85.8571243 37.9095336 21000

Amblyomma americanum americanum A. americanum -85.8571243 37.9095336 21000

Amblyomma americanum americanum A. americanum -85.8571243 37.9095336 21000

Amblyomma americanum americanum A. americanum -85.8571243 37.9095336 21000

Amblyomma americanum americanum A. americanum -85.8571243 37.9095336 21000

Amblyomma americanum americanum A. americanum -85.8571243 37.9095336 21000

Amblyomma americanum americanum A. americanum -85.8571243 37.9095336 21000

Amblyomma americanum americanum A. americanum -85.8571243 37.9095336 21000

Amblyomma americanum americanum A. americanum -76.84515 39.035445 2000

Amblyomma americanum americanum A. americanum -85.8571243 37.9095336 21000

Amblyomma americanum americanum A. americanum -85.8571243 37.9095336 21000

Amblyomma americanum americanum A. americanum -85.8571243 37.9095336 21000

Amblyomma americanum americanum A. americanum -85.8571243 37.9095336 21000

Amblyomma americanum americanum A. americanum -85.8571243 37.9095336 21000

Amblyomma americanum americanum A. americanum -85.8571243 37.9095336 21000

Amblyomma americanum americanum A. americanum -85.8571243 37.9095336 21000

Amblyomma americanum americanum A. americanum -85.8571243 37.9095336 21000

Amblyomma americanum americanum A. americanum -85.8571243 37.9095336 21000

Amblyomma americanum americanum A. americanum -85.8571243 37.9095336 21000

Amblyomma americanum americanum A. americanum -85.8571243 37.9095336 21000

Amblyomma americanum americanum A. americanum -85.8571243 37.9095336 21000

Amblyomma americanum americanum A. americanum -85.8571243 37.9095336 21000

Amblyomma americanum americanum A. americanum -85.8571243 37.9095336 21000

Amblyomma americanum americanum A. americanum -85.8571243 37.9095336 21000

Amblyomma americanum americanum A. americanum -85.8571243 37.9095336 21000

Amblyomma americanum americanum A. americanum -85.8571243 37.9095336 21000

Amblyomma americanum americanum A. americanum -85.8571243 37.9095336 21000

Amblyomma americanum americanum A. americanum -85.8571243 37.9095336 21000

Amblyomma americanum americanum A. americanum -85.8571243 37.9095336 21000

Amblyomma americanum americanum A. americanum -85.8571243 37.9095336 21000

Amblyomma americanum americanum A. americanum -85.8571243 37.9095336 21000

Amblyomma americanum americanum A. americanum -85.8571243 37.9095336 21000

Amblyomma americanum americanum A. americanum -85.8571243 37.9095336 21000

Amblyomma americanum americanum A. americanum -85.8571243 37.9095336 21000

Amblyomma americanum americanum A. americanum -85.8571243 37.9095336 21000

Amblyomma americanum americanum A. americanum -85.8571243 37.9095336 21000

Amblyomma americanum americanum A. americanum -85.8571243 37.9095336 21000

Amblyomma americanum americanum A. americanum -85.8571243 37.9095336 21000

Amblyomma americanum americanum A. americanum -85.8571243 37.9095336 21000

Amblyomma americanum americanum A. americanum -85.8571243 37.9095336 21000

Amblyomma americanum americanum A. americanum -85.8571243 37.9095336 21000

Amblyomma americanum americanum A. americanum -85.8571243 37.9095336 21000

Amblyomma americanum americanum A. americanum -85.8571243 37.9095336 21000

Amblyomma americanum americanum A. americanum -85.8571243 37.9095336 21000

Amblyomma americanum americanum A. americanum -85.8571243 37.9095336 21000

Amblyomma americanum americanum A. americanum -85.8571243 37.9095336 21000

Amblyomma americanum americanum A. americanum -85.8571243 37.9095336 21000

Amblyomma americanum americanum A. americanum -85.8571243 37.9095336 21000

Amblyomma americanum americanum A. americanum -85.8571243 37.9095336 21000

Amblyomma americanum americanum A. americanum -85.8571243 37.9095336 21000

Amblyomma americanum americanum A. americanum -85.8571243 37.9095336 21000

Amblyomma americanum americanum A. americanum -85.8571243 37.9095336 21000

Amblyomma americanum americanum A. americanum -85.8571243 37.9095336 21000

Amblyomma americanum americanum A. americanum -85.8571243 37.9095336 21000

Amblyomma americanum americanum A. americanum -85.8571243 37.9095336 21000

Amblyomma americanum americanum A. americanum -85.8571243 37.9095336 21000

Amblyomma americanum americanum A. americanum -85.8571243 37.9095336 21000

Amblyomma americanum americanum A. americanum -85.8571243 37.9095336 21000

Amblyomma americanum americanum A. americanum -85.8571243 37.9095336 21000

Amblyomma americanum americanum A. americanum -85.8571243 37.9095336 21000

Amblyomma americanum americanum A. americanum -85.8571243 37.9095336 21000

Amblyomma americanum americanum A. americanum -85.8571243 37.9095336 21000

Amblyomma americanum americanum A. americanum -85.8571243 37.9095336 21000

Amblyomma americanum americanum A. americanum -85.8571243 37.9095336 21000

Amblyomma americanum americanum A. americanum -85.8571243 37.9095336 21000

Amblyomma americanum americanum A. americanum -76.1647796 39.4442798 12000

Amblyomma americanum americanum A. americanum -76.1647796 39.4442798 12000

Amblyomma americanum americanum A. americanum -79.1970062 35.1109218 17000

Amblyomma americanum americanum A. americanum -77.3341369 37.2359319 4000

Amblyomma americanum americanum A. americanum -76.882867 38.996036 500

Amblyomma americanum americanum A. americanum -76.882867 38.996036 500

Amblyomma americanum americanum A. americanum -77.915039 37.0420244 10500

Amblyomma americanum americanum A. americanum -77.915039 37.0420244 10500

Amblyomma americanum americanum A. americanum -77.915039 37.0420244 10500

Amblyomma americanum americanum A. americanum -77.915039 37.0420244 10500

Amblyomma americanum americanum A. americanum -77.915039 37.0420244 10500

Amblyomma americanum americanum A. americanum -77.915039 37.0420244 10500

Amblyomma americanum americanum A. americanum -77.915039 37.0420244 10500

Amblyomma americanum americanum A. americanum -77.915039 37.0420244 10500

Amblyomma americanum americanum A. americanum -76.1647796 39.4442798 12000

Amblyomma americanum americanum A. americanum -76.1647796 39.4442798 12000

Amblyomma americanum americanum A. americanum -78.31089 36.600921 5000

Amblyomma americanum americanum A. americanum -76.7734909 39.0642483 10000

Amblyomma americanum americanum A. americanum -76.1647796 39.4442798 12000

Amblyomma americanum americanum A. americanum -76.1647796 39.4442798 12000

Amblyomma americanum americanum A. americanum -85.8571243 37.9095336 21000

Amblyomma americanum americanum A. americanum -85.8571243 37.9095336 21000

Amblyomma americanum americanum A. americanum -85.8571243 37.9095336 21000

Amblyomma americanum americanum A. americanum -85.8571243 37.9095336 21000

Amblyomma americanum americanum A. americanum -85.8571243 37.9095336 21000

Amblyomma americanum americanum A. americanum -85.8571243 37.9095336 21000

Amblyomma americanum americanum A. americanum -85.8571243 37.9095336 21000

Amblyomma americanum americanum A. americanum -85.8571243 37.9095336 21000

Amblyomma americanum americanum A. americanum -85.8571243 37.9095336 21000

Amblyomma americanum americanum A. americanum -85.8571243 37.9095336 21000

Amblyomma americanum americanum A. americanum -85.8571243 37.9095336 21000

Amblyomma americanum americanum A. americanum -85.8571243 37.9095336 21000

Amblyomma americanum americanum A. americanum -85.8571243 37.9095336 21000

Amblyomma americanum americanum A. americanum -85.8571243 37.9095336 21000

Amblyomma americanum americanum A. americanum -85.8571243 37.9095336 21000

Amblyomma americanum americanum A. americanum -85.8571243 37.9095336 21000

Amblyomma americanum americanum A. americanum -85.8571243 37.9095336 21000

Amblyomma americanum americanum A. americanum -85.8571243 37.9095336 21000

Amblyomma americanum americanum A. americanum -85.8571243 37.9095336 21000

Amblyomma americanum americanum A. americanum -85.8571243 37.9095336 21000

Amblyomma americanum americanum A. americanum -85.8571243 37.9095336 21000

Amblyomma americanum americanum A. americanum -85.8571243 37.9095336 21000

Amblyomma americanum americanum A. americanum -85.8571243 37.9095336 21000

Amblyomma americanum americanum A. americanum -85.8571243 37.9095336 21000

Amblyomma americanum americanum A. americanum -85.8571243 37.9095336 21000

Amblyomma americanum americanum A. americanum -85.8571243 37.9095336 21000

Amblyomma americanum americanum A. americanum -85.8571243 37.9095336 21000

Amblyomma americanum americanum A. americanum -85.8571243 37.9095336 21000

Amblyomma americanum americanum A. americanum -85.8571243 37.9095336 21000

Amblyomma americanum americanum A. americanum -85.8571243 37.9095336 21000

Amblyomma americanum americanum A. americanum -85.8571243 37.9095336 21000

Amblyomma americanum americanum A. americanum -85.8571243 37.9095336 21000

Amblyomma americanum americanum A. americanum -85.8571243 37.9095336 21000

Amblyomma americanum americanum A. americanum -85.8571243 37.9095336 21000

Amblyomma americanum americanum A. americanum -85.8571243 37.9095336 21000

Amblyomma americanum americanum A. americanum -85.8571243 37.9095336 21000

Amblyomma americanum americanum A. americanum -85.8571243 37.9095336 21000

Amblyomma americanum americanum A. americanum -85.8571243 37.9095336 21000

Amblyomma americanum americanum A. americanum -85.8571243 37.9095336 21000

Amblyomma americanum americanum A. americanum -85.8571243 37.9095336 21000

Amblyomma americanum americanum A. americanum -85.8571243 37.9095336 21000

Amblyomma americanum americanum A. americanum -85.8571243 37.9095336 21000

Amblyomma americanum americanum A. americanum -85.8571243 37.9095336 21000

Amblyomma americanum americanum A. americanum -85.8571243 37.9095336 21000

Amblyomma americanum americanum A. americanum -85.8571243 37.9095336 21000

Amblyomma americanum americanum A. americanum -85.8571243 37.9095336 21000

Amblyomma americanum americanum A. americanum -85.8571243 37.9095336 21000

Amblyomma americanum americanum A. americanum -85.8571243 37.9095336 21000

Amblyomma americanum americanum A. americanum -85.8571243 37.9095336 21000

Amblyomma americanum americanum A. americanum -85.8571243 37.9095336 21000

Amblyomma americanum americanum A. americanum -85.8571243 37.9095336 21000

Amblyomma americanum americanum A. americanum -85.8571243 37.9095336 21000

Amblyomma americanum americanum A. americanum -85.8571243 37.9095336 21000

Amblyomma americanum americanum A. americanum -85.8571243 37.9095336 21000

Amblyomma americanum americanum A. americanum -85.8571243 37.9095336 21000

Amblyomma americanum americanum A. americanum -85.8571243 37.9095336 21000

Amblyomma americanum americanum A. americanum -85.8571243 37.9095336 21000

Amblyomma americanum americanum A. americanum -85.8571243 37.9095336 21000

Amblyomma americanum americanum A. americanum -85.8571243 37.9095336 21000

Amblyomma americanum americanum A. americanum -85.8571243 37.9095336 21000

Amblyomma americanum americanum A. americanum -85.8571243 37.9095336 21000

Amblyomma americanum americanum A. americanum -85.8571243 37.9095336 21000

Amblyomma americanum americanum A. americanum -85.8571243 37.9095336 21000

Amblyomma americanum americanum A. americanum -85.8571243 37.9095336 21000

Amblyomma americanum americanum A. americanum -85.8571243 37.9095336 21000

Amblyomma americanum americanum A. americanum -85.8571243 37.9095336 21000

Amblyomma americanum americanum A. americanum -85.8571243 37.9095336 21000

Amblyomma americanum americanum A. americanum -85.8571243 37.9095336 21000

Amblyomma americanum americanum A. americanum -85.8571243 37.9095336 21000

Amblyomma americanum americanum A. americanum -85.8571243 37.9095336 21000

Amblyomma americanum americanum A. americanum -85.8571243 37.9095336 21000

Amblyomma americanum americanum A. americanum -85.8571243 37.9095336 21000

Amblyomma americanum americanum A. americanum -85.8571243 37.9095336 21000

Amblyomma americanum americanum A. americanum -85.8571243 37.9095336 21000

Amblyomma americanum americanum A. americanum -85.8571243 37.9095336 21000

Amblyomma americanum americanum A. americanum -85.8571243 37.9095336 21000

Amblyomma americanum americanum A. americanum -85.8571243 37.9095336 21000

Amblyomma americanum americanum A. americanum -85.8571243 37.9095336 21000

Amblyomma americanum americanum A. americanum -85.8571243 37.9095336 21000

Amblyomma americanum americanum A. americanum -85.8571243 37.9095336 21000

Amblyomma americanum americanum A. americanum -85.8571243 37.9095336 21000

Amblyomma americanum americanum A. americanum -85.8571243 37.9095336 21000

Amblyomma americanum americanum A. americanum -85.8571243 37.9095336 21000

Amblyomma americanum americanum A. americanum -85.8571243 37.9095336 21000

Amblyomma americanum americanum A. americanum -85.8571243 37.9095336 21000

Amblyomma americanum americanum A. americanum -85.8571243 37.9095336 21000

Amblyomma americanum americanum A. americanum -85.8571243 37.9095336 21000

Amblyomma americanum americanum A. americanum -85.8571243 37.9095336 21000

Amblyomma americanum americanum A. americanum -85.8571243 37.9095336 21000

Amblyomma americanum americanum A. americanum -85.8571243 37.9095336 21000

Amblyomma americanum americanum A. americanum -85.8571243 37.9095336 21000

Amblyomma americanum americanum A. americanum -85.8571243 37.9095336 21000

Amblyomma americanum americanum A. americanum -85.8571243 37.9095336 21000

Amblyomma americanum americanum A. americanum -85.8571243 37.9095336 21000

Amblyomma americanum americanum A. americanum -85.8571243 37.9095336 21000

Amblyomma americanum americanum A. americanum -85.8571243 37.9095336 21000

Amblyomma americanum americanum A. americanum -85.8571243 37.9095336 21000

Amblyomma americanum americanum A. americanum -85.8571243 37.9095336 21000

Amblyomma americanum americanum A. americanum -85.8571243 37.9095336 21000

Amblyomma americanum americanum A. americanum -85.8571243 37.9095336 21000

Amblyomma americanum americanum A. americanum -85.8571243 37.9095336 21000

Amblyomma americanum americanum A. americanum -85.8571243 37.9095336 21000

Amblyomma americanum americanum A. americanum -85.8571243 37.9095336 21000

Amblyomma americanum americanum A. americanum -90.6749725 44.0382442 18000

Amblyomma americanum americanum A. americanum -76.1647796 39.4442798 12000

Amblyomma americanum americanum A. americanum -76.1647796 39.4442798 12000

Amblyomma americanum americanum A. americanum -74.1514316 40.2555169 40840

Amblyomma americanum americanum A. americanum -77.915039 37.0420244 10500

Amblyomma americanum americanum A. americanum -76.1647796 39.4442798 12000

Amblyomma americanum americanum A. americanum -74.576966 40.024197 8000

Amblyomma americanum americanum A. americanum -87.6159667 36.6078112 18500

Amblyomma americanum americanum A. americanum -74.1514316 40.2555169 40840

Amblyomma americanum americanum A. americanum -76.882867 38.996036 500

Amblyomma americanum americanum A. americanum -76.882867 38.996036 500

Amblyomma americanum americanum A. americanum -76.1647796 39.4442798 12000

Amblyomma americanum americanum A. americanum -74.576966 40.024197 8000

Amblyomma americanum americanum A. americanum -76.1647796 39.4442798 12000

Amblyomma americanum americanum A. americanum -76.1647796 39.4442798 12000

Amblyomma americanum americanum A. americanum -76.1647796 39.4442798 12000

Amblyomma americanum americanum A. americanum -76.84515 39.035445 2000

Amblyomma americanum americanum A. americanum -76.84515 39.035445 2000

Amblyomma americanum americanum A. americanum -76.1647796 39.4442798 12000

Amblyomma americanum americanum A. americanum -74.1514316 40.2555169 40840

Amblyomma americanum americanum A. americanum -76.1647796 39.4442798 12000

Amblyomma americanum americanum A. americanum -85.8571243 37.9095336 21000

Amblyomma americanum americanum A. americanum -85.8571243 37.9095336 21000

Amblyomma americanum americanum A. americanum -85.8571243 37.9095336 21000

Amblyomma americanum americanum A. americanum -85.8571243 37.9095336 21000

Amblyomma americanum americanum A. americanum -85.8571243 37.9095336 21000

Amblyomma americanum americanum A. americanum -85.8571243 37.9095336 21000

Amblyomma americanum americanum A. americanum -85.8571243 37.9095336 21000

Amblyomma americanum americanum A. americanum -85.8571243 37.9095336 21000

Amblyomma americanum americanum A. americanum -85.8571243 37.9095336 21000

Amblyomma americanum americanum A. americanum -85.8571243 37.9095336 21000

Amblyomma americanum americanum A. americanum -85.8571243 37.9095336 21000

Amblyomma americanum americanum A. americanum -85.8571243 37.9095336 21000

Amblyomma americanum americanum A. americanum -85.8571243 37.9095336 21000

Amblyomma americanum americanum A. americanum -85.8571243 37.9095336 21000

Amblyomma americanum americanum A. americanum -85.8571243 37.9095336 21000

Amblyomma americanum americanum A. americanum -85.8571243 37.9095336 21000

Amblyomma americanum americanum A. americanum -85.8571243 37.9095336 21000

Amblyomma americanum americanum A. americanum -85.8571243 37.9095336 21000

Amblyomma americanum americanum A. americanum -85.8571243 37.9095336 21000

Amblyomma americanum americanum A. americanum -85.8571243 37.9095336 21000

Amblyomma americanum americanum A. americanum -85.8571243 37.9095336 21000

Amblyomma americanum americanum A. americanum -85.8571243 37.9095336 21000

Amblyomma americanum americanum A. americanum -85.8571243 37.9095336 21000

Amblyomma americanum americanum A. americanum -85.8571243 37.9095336 21000

Amblyomma americanum americanum A. americanum -85.8571243 37.9095336 21000

Amblyomma americanum americanum A. americanum -85.8571243 37.9095336 21000

Amblyomma americanum americanum A. americanum -85.8571243 37.9095336 21000

Amblyomma americanum americanum A. americanum -85.8571243 37.9095336 21000

Amblyomma americanum americanum A. americanum -85.8571243 37.9095336 21000

Amblyomma americanum americanum A. americanum -85.8571243 37.9095336 21000

Amblyomma americanum americanum A. americanum -85.8571243 37.9095336 21000

Amblyomma americanum americanum A. americanum -85.8571243 37.9095336 21000

Amblyomma americanum americanum A. americanum -85.8571243 37.9095336 21000

Amblyomma americanum americanum A. americanum -85.8571243 37.9095336 21000

Amblyomma americanum americanum A. americanum -85.8571243 37.9095336 21000

Amblyomma americanum americanum A. americanum -76.1647796 39.4442798 12000

Amblyomma americanum americanum A. americanum -76.1647796 39.4442798 12000

Amblyomma americanum americanum A. americanum -77.915039 37.0420244 10500

Amblyomma americanum americanum A. americanum -76.1647796 39.4442798 12000

Amblyomma americanum americanum A. americanum -77.3341369 37.2359319 4000

Amblyomma americanum americanum A. americanum -76.84515 39.035445 2000

Amblyomma americanum americanum A. americanum -76.1647796 39.4442798 12000

Amblyomma americanum americanum A. americanum -76.1647796 39.4442798 12000

Amblyomma americanum americanum A. americanum -77.3341369 37.2359319 4000

Amblyomma americanum americanum A. americanum -87.6159667 36.6078112 18500

Amblyomma americanum americanum A. americanum -74.576966 40.024197 8000

Amblyomma americanum americanum A. americanum -76.1647796 39.4442798 12000

Amblyomma americanum americanum A. americanum -76.1647796 39.4442798 12000

Amblyomma americanum americanum A. americanum -77.4568391 38.5520281 15927

Amblyomma americanum americanum A. americanum -76.1647796 39.4442798 12000

Amblyomma americanum americanum A. americanum -87.6159667 36.6078112 18500

Amblyomma americanum americanum A. americanum -85.8571243 37.9095336 21000

Amblyomma americanum americanum A. americanum -85.8571243 37.9095336 21000

Amblyomma americanum americanum A. americanum -85.8571243 37.9095336 21000

Amblyomma americanum americanum A. americanum -85.8571243 37.9095336 21000

Amblyomma americanum americanum A. americanum -85.8571243 37.9095336 21000

Amblyomma americanum americanum A. americanum -85.8571243 37.9095336 21000

Amblyomma americanum americanum A. americanum -85.8571243 37.9095336 21000

Amblyomma americanum americanum A. americanum -85.8571243 37.9095336 21000

Amblyomma americanum americanum A. americanum -85.8571243 37.9095336 21000

Amblyomma americanum americanum A. americanum -85.8571243 37.9095336 21000

Amblyomma americanum americanum A. americanum -85.8571243 37.9095336 21000

Amblyomma americanum americanum A. americanum -85.8571243 37.9095336 21000

Amblyomma americanum americanum A. americanum -85.8571243 37.9095336 21000

Amblyomma americanum americanum A. americanum -85.8571243 37.9095336 21000

Amblyomma americanum americanum A. americanum -85.8571243 37.9095336 21000

Amblyomma americanum americanum A. americanum -85.8571243 37.9095336 21000

Amblyomma americanum americanum A. americanum -85.8571243 37.9095336 21000

Amblyomma americanum americanum A. americanum -85.8571243 37.9095336 21000

Amblyomma americanum americanum A. americanum -85.8571243 37.9095336 21000

Amblyomma americanum americanum A. americanum -85.8571243 37.9095336 21000

Amblyomma americanum americanum A. americanum -85.8571243 37.9095336 21000

Amblyomma americanum americanum A. americanum -85.8571243 37.9095336 21000

Amblyomma americanum americanum A. americanum -85.8571243 37.9095336 21000

Amblyomma americanum americanum A. americanum -85.8571243 37.9095336 21000

Amblyomma americanum americanum A. americanum -85.8571243 37.9095336 21000

Amblyomma americanum americanum A. americanum -85.8571243 37.9095336 21000

Amblyomma americanum americanum A. americanum -85.8571243 37.9095336 21000

Amblyomma americanum americanum A. americanum -85.8571243 37.9095336 21000

Amblyomma americanum americanum A. americanum -85.8571243 37.9095336 21000

Amblyomma americanum americanum A. americanum -85.8571243 37.9095336 21000

Amblyomma americanum americanum A. americanum -85.8571243 37.9095336 21000

Amblyomma americanum americanum A. americanum -85.8571243 37.9095336 21000

Amblyomma americanum americanum A. americanum -74.576966 40.024197 8000

Amblyomma americanum americanum A. americanum -76.7734909 39.0642483 10000

Amblyomma americanum americanum A. americanum -87.6159667 36.6078112 18500

Amblyomma americanum americanum A. americanum -77.1459102 38.7021234 3500

Amblyomma americanum americanum A. americanum -86.5295486 30.572647 44515

Amblyomma americanum americanum A. americanum -74.1514316 40.2555169 40840

Amblyomma americanum americanum A. americanum -74.1514316 40.2555169 40840

Amblyomma americanum americanum A. americanum -77.4568391 38.5520281 15927

Amblyomma americanum americanum A. americanum -77.3341369 37.2359319 4000

Amblyomma americanum americanum A. americanum -76.7734909 39.0642483 10000

Amblyomma americanum americanum A. americanum -85.77472 37.14028 3036

Amblyomma americanum americanum A. americanum -76.5396843 39.377327 5062

Amblyomma americanum americanum A. americanum -76.5710793 39.107538 5085

Amblyomma americanum americanum A. americanum -76.5710793 39.107538 5085

Amblyomma americanum americanum A. americanum -76.5710793 39.107538 5085

Amblyomma americanum americanum A. americanum -76.5710793 39.107538 5085

Amblyomma americanum americanum A. americanum -76.5710793 39.107538 5085

Amblyomma americanum americanum A. americanum -76.5710793 39.107538 5085

Amblyomma americanum americanum A. americanum -74.6829338 39.9720612 5062

Amblyomma americanum americanum A. americanum -74.6829338 39.9720612 5062

Amblyomma americanum americanum A. americanum -79.62833 36.98222 6113

Amblyomma americanum americanum A. americanum -76.0636921 39.5746197 5079

Amblyomma americanum americanum A. americanum -76.0636921 39.5746197 5079

Amblyomma americanum americanum A. americanum -77.4131082 37.2012885 4110

Amblyomma americanum americanum A. americanum -77.911263 37.056273 5000

Amblyomma americanum americanum A. americanum -77.915039 37.0420244 10500

Amblyomma americanum americanum A. americanum -77.915039 37.0420244 10500

Amblyomma americanum americanum A. americanum -77.915039 37.0420244 10500

Amblyomma americanum americanum A. americanum -77.915039 37.0420244 10500

Amblyomma americanum americanum A. americanum -77.915039 37.0420244 10500

Amblyomma americanum americanum A. americanum -77.915039 37.0420244 10500

Amblyomma americanum americanum A. americanum -77.915039 37.0420244 10500

Amblyomma americanum americanum A. americanum -77.915039 37.0420244 10500

Amblyomma americanum americanum A. americanum -77.915039 37.0420244 10500

Amblyomma americanum americanum A. americanum -77.915039 37.0420244 10500

Amblyomma americanum americanum A. americanum -77.915039 37.0420244 10500

Amblyomma americanum americanum A. americanum -77.915039 37.0420244 10500

Amblyomma americanum americanum A. americanum -77.915039 37.0420244 10500

Amblyomma americanum americanum A. americanum -77.915039 37.0420244 10500

Amblyomma americanum americanum A. americanum -77.915039 37.0420244 10500

Amblyomma americanum americanum A. americanum -77.915039 37.0420244 10500

Amblyomma americanum americanum A. americanum -77.915039 37.0420244 10500

Amblyomma americanum americanum A. americanum -77.915039 37.0420244 10500

Amblyomma americanum americanum A. americanum -77.915039 37.0420244 10500

Amblyomma americanum americanum A. americanum -77.915039 37.0420244 10500

Amblyomma americanum americanum A. americanum -77.915039 37.0420244 10500

Amblyomma americanum americanum A. americanum -77.915039 37.0420244 10500

Amblyomma americanum americanum A. americanum -77.915039 37.0420244 10500

Amblyomma americanum americanum A. americanum -77.915039 37.0420244 10500

Amblyomma americanum americanum A. americanum -77.915039 37.0420244 10500

Amblyomma americanum americanum A. americanum -77.915039 37.0420244 10500

Amblyomma americanum americanum A. americanum -77.915039 37.0420244 10500

Amblyomma americanum americanum A. americanum -77.915039 37.0420244 10500

Amblyomma americanum americanum A. americanum -77.915039 37.0420244 10500

Amblyomma americanum americanum A. americanum -77.915039 37.0420244 10500

Amblyomma americanum americanum A. americanum -77.915039 37.0420244 10500

Amblyomma americanum americanum A. americanum -77.915039 37.0420244 10500

Amblyomma americanum americanum A. americanum -77.915039 37.0420244 10500

Amblyomma americanum americanum A. americanum -77.915039 37.0420244 10500

Amblyomma americanum americanum A. americanum -94.86111 39.14306 3036

Amblyomma americanum americanum A. americanum -87.0409347 36.3879927 5345

Amblyomma americanum americanum A. americanum -71.2503204 41.6011111 5187

Amblyomma americanum americanum A. americanum -77.055 37.64389 3036

Amblyomma americanum americanum A. americanum -77.2880363 37.2204266 5062

Amblyomma americanum americanum A. americanum -77.2880363 37.2204266 5062

Amblyomma americanum americanum A. americanum -77.2880363 37.2204266 5062

Amblyomma americanum americanum A. americanum -76.90806 37.79 3036

Amblyomma americanum americanum A. americanum -76.90806 37.79 3036

Amblyomma americanum americanum A. americanum -76.3730125 39.6827374 5625

Amblyomma americanum americanum A. americanum -77.3050865 38.5344991 4853

Amblyomma americanum americanum A. americanum -77.3050865 38.5344991 4853

Amblyomma americanum americanum A. americanum -77.3050865 38.5344991 4853

Amblyomma americanum americanum A. americanum -85.9474654 37.8403473 5198

Amblyomma americanum americanum A. americanum -85.9474654 37.8403473 5198

Amblyomma americanum americanum A. americanum -85.9474654 37.8403473 5198

Amblyomma americanum americanum A. americanum -85.9474654 37.8403473 5198

Amblyomma americanum americanum A. americanum -78.9069633 35.4726601 5067

Amblyomma americanum americanum A. americanum -78.61528 36.78417 3036

Amblyomma americanum americanum A. americanum -77.3410988 38.9687214 5062

Amblyomma americanum americanum A. americanum -77.28472 36.27389 1863

Amblyomma americanum americanum A. americanum -92.4043427 37.8569851 5062

Amblyomma americanum americanum A. americanum -77.4602623 37.5537605 5062

Amblyomma americanum americanum A. americanum -80.96056 34.3075 1141

Amblyomma americanum americanum A. americanum -81.23556 32.29583 3833

Amblyomma americanum americanum A. americanum -85.9699593 37.7495079 5062

Amblyomma americanum americanum A. americanum -76.0627251 39.6875458 5767

Amblyomma americanum americanum A. americanum -75.51583 39.10028 3036

Amblyomma americanum americanum A. americanum -77.97917 38.57972 3036

Amblyomma americanum americanum A. americanum -96.83056 39.29889 977

Amblyomma americanum americanum A. americanum -79.9414253 37.2709694 5062

Amblyomma americanum americanum A. americanum -77.8133342 35.9406803 5576

Amblyomma americanum americanum A. americanum -78.45778 35.92306 2999

Amblyomma americanum americanum A. americanum -91.77111 37.95139 6697

Amblyomma americanum americanum A. americanum -73.32806 43.17222 2349

Amblyomma americanum americanum A. americanum -98.49333 29.42389 36305

Amblyomma americanum americanum A. americanum -79.1803017 35.4798756 5063

Amblyomma americanum americanum A. americanum -79.1803017 35.4798756 5063

Amblyomma americanum americanum A. americanum -79.1803017 35.4798756 5063

Amblyomma americanum americanum A. americanum -79.1803017 35.4798756 5063

Amblyomma americanum americanum A. americanum -76.82417 39.13778 3036

Amblyomma americanum americanum A. americanum -81.0998344 32.0820937 5212

Amblyomma americanum americanum A. americanum -76.6982994 39.1370525 5062

Amblyomma americanum americanum A. americanum -76.6982994 39.1370525 5062

Amblyomma americanum americanum A. americanum -76.6982994 39.1370525 5062

Amblyomma americanum americanum A. americanum -76.5465393 39.070385 5167

Amblyomma americanum americanum A. americanum -76.5465393 39.070385 5167

Amblyomma americanum americanum A. americanum -79.4625 35.72333 3053

Amblyomma americanum americanum A. americanum -77.0299744 39.0022059 5533

Amblyomma americanum americanum A. americanum -77.0299744 39.0022059 5533

Amblyomma americanum americanum A. americanum -97.50889 28.03639 2546

Amblyomma americanum americanum A. americanum -79.86111 35.82306 3036

Amblyomma americanum americanum A. americanum -78.02056 33.92139 2789

Amblyomma americanum americanum A. americanum -78.02056 33.92139 2789

Amblyomma americanum americanum A. americanum -78.02056 33.92139 2789

Amblyomma americanum americanum A. americanum -78.02056 33.92139 2789

Amblyomma americanum americanum A. americanum -77.5891571 38.2009659 5062

Amblyomma americanum americanum A. americanum -77.5891571 38.2009659 5062

Amblyomma americanum americanum A. americanum -77.1872063 38.7892799 5062

Amblyomma americanum americanum A. americanum -78.9740015 35.2123843 855

Amblyomma americanum americanum A. americanum -78.9740015 35.2123843 855

Amblyomma americanum americanum A. americanum -77.1872063 38.7892799 5062

Amblyomma americanum americanum A. americanum -77.1872063 38.7892799 5062

Amblyomma americanum americanum A. americanum -77.1872063 38.7892799 5062

Amblyomma americanum americanum A. americanum -77.1872063 38.7892799 5062

Amblyomma americanum americanum A. americanum -77.1872063 38.7892799 5062

Amblyomma americanum americanum A. americanum -77.1872063 38.7892799 5062

Amblyomma americanum americanum A. americanum -77.1872063 38.7892799 5062

Amblyomma americanum americanum A. americanum -77.1872063 38.7892799 5062

Amblyomma americanum americanum A. americanum -77.1872063 38.7892799 5062

Amblyomma americanum americanum A. americanum -77.1872063 38.7892799 5062

Amblyomma americanum americanum A. americanum -77.1872063 38.7892799 5062

Amblyomma americanum americanum A. americanum -77.1872063 38.7892799 5062

Amblyomma americanum americanum A. americanum -77.1872063 38.7892799 5062

Amblyomma americanum americanum A. americanum -77.1872063 38.7892799 5062

Amblyomma americanum americanum A. americanum -77.1872063 38.7892799 5062

Amblyomma americanum americanum A. americanum -77.1872063 38.7892799 5062

Amblyomma americanum americanum A. americanum -77.1872063 38.7892799 5062

Amblyomma americanum americanum A. americanum -77.1872063 38.7892799 5062

Amblyomma americanum americanum A. americanum -77.1872063 38.7892799 5062

Amblyomma americanum americanum A. americanum -77.1872063 38.7892799 5062

Amblyomma americanum americanum A. americanum -77.1872063 38.7892799 5062

Amblyomma americanum americanum A. americanum -77.1872063 38.7892799 5062

Amblyomma americanum americanum A. americanum -77.1872063 38.7892799 5062

Amblyomma americanum americanum A. americanum -77.1872063 38.7892799 5062

Amblyomma americanum americanum A. americanum -77.1872063 38.7892799 5062

Amblyomma americanum americanum A. americanum -77.1872063 38.7892799 5062

Amblyomma americanum americanum A. americanum -77.1872063 38.7892799 5062

Amblyomma americanum americanum A. americanum -77.1872063 38.7892799 5062

Amblyomma americanum americanum A. americanum -77.1872063 38.7892799 5062

Amblyomma americanum americanum A. americanum -77.1872063 38.7892799 5062

Amblyomma americanum americanum A. americanum -77.1872063 38.7892799 5062

Amblyomma americanum americanum A. americanum -77.1872063 38.7892799 5062

Amblyomma americanum americanum A. americanum -77.1872063 38.7892799 5062

Amblyomma americanum americanum A. americanum -77.1872063 38.7892799 5062

Amblyomma americanum americanum A. americanum -77.1872063 38.7892799 5062

Amblyomma americanum americanum A. americanum -77.1872063 38.7892799 5062

Amblyomma americanum americanum A. americanum -77.1872063 38.7892799 5062

Amblyomma americanum americanum A. americanum -77.1872063 38.7892799 5062

Amblyomma americanum americanum A. americanum -77.1872063 38.7892799 5062

Amblyomma americanum americanum A. americanum -77.1872063 38.7892799 5062

Amblyomma americanum americanum A. americanum -77.1872063 38.7892799 5062

Amblyomma americanum americanum A. americanum -77.1872063 38.7892799 5062

Amblyomma americanum americanum A. americanum -77.1872063 38.7892799 5062

Amblyomma americanum americanum A. americanum -77.1872063 38.7892799 5062

Amblyomma americanum americanum A. americanum -77.1872063 38.7892799 5062

Amblyomma americanum americanum A. americanum -92.1776695 37.8280964 5062

Amblyomma americanum americanum A. americanum -92.1776695 37.8280964 5062

Amblyomma americanum americanum A. americanum -92.1776695 37.8280964 5062

Amblyomma americanum americanum A. americanum -92.1776695 37.8280964 5062

Amblyomma americanum americanum A. americanum -92.1776695 37.8280964 5062

Amblyomma americanum americanum A. americanum -76.22472 38.785 3036

Amblyomma americanum americanum A. americanum -77.4089934 38.4220676 5117

Amblyomma americanum americanum A. americanum -77.4089934 38.4220676 5117

Amblyomma americanum americanum A. americanum -77.4089934 38.4220676 5117

Amblyomma americanum americanum A. americanum -77.4089934 38.4220676 5117

Amblyomma americanum americanum A. americanum -77.4089934 38.4220676 5117

Amblyomma americanum americanum A. americanum -77.4089934 38.4220676 5117

Amblyomma americanum americanum A. americanum -77.4089934 38.4220676 5117

Amblyomma americanum americanum A. americanum -77.4089934 38.4220676 5117

Amblyomma americanum americanum A. americanum -77.4089934 38.4220676 5117

Amblyomma americanum americanum A. americanum -77.4089934 38.4220676 5117

Amblyomma americanum americanum A. americanum -77.4089934 38.4220676 5117

Amblyomma americanum americanum A. americanum -77.4089934 38.4220676 5117

Amblyomma americanum americanum A. americanum -77.4089934 38.4220676 5117

Amblyomma americanum americanum A. americanum -77.4089934 38.4220676 5117

Amblyomma americanum americanum A. americanum -77.4089934 38.4220676 5117

Amblyomma americanum americanum A. americanum -77.4089934 38.4220676 5117

Amblyomma americanum americanum A. americanum -77.4089934 38.4220676 5117

Amblyomma americanum americanum A. americanum -79.5749397 35.7959709 5784

Amblyomma americanum americanum A. americanum -79.5749397 35.7959709 5784

Amblyomma americanum americanum A. americanum -79.5749397 35.7959709 5784

Amblyomma americanum americanum A. americanum -79.5749397 35.7959709 5784

Amblyomma americanum americanum A. americanum -79.5749397 35.7959709 5784

Amblyomma americanum americanum A. americanum -79.5749397 35.7959709 5784

Amblyomma americanum americanum A. americanum -79.5749397 35.7959709 5784

Amblyomma americanum americanum A. americanum -79.5749397 35.7959709 5784

Amblyomma americanum americanum A. americanum -79.5749397 35.7959709 5784

Amblyomma americanum americanum A. americanum -79.5749397 35.7959709 5784

Amblyomma americanum americanum A. americanum -79.5749397 35.7959709 5784

Amblyomma americanum americanum A. americanum -79.5749397 35.7959709 5784

Amblyomma americanum americanum A. americanum -79.5749397 35.7959709 5784

Amblyomma americanum americanum A. americanum -79.5749397 35.7959709 5784

Amblyomma americanum americanum A. americanum -79.5749397 35.7959709 5784

Amblyomma americanum americanum A. americanum -79.5749397 35.7959709 5784

Amblyomma americanum americanum A. americanum -79.5749397 35.7959709 5784

Amblyomma americanum americanum A. americanum -79.5749397 35.7959709 5784

Amblyomma americanum americanum A. americanum -79.5749397 35.7959709 5784

Amblyomma americanum americanum A. americanum -79.5749397 35.7959709 5784

Amblyomma americanum americanum A. americanum -79.5749397 35.7959709 5784

Amblyomma americanum americanum A. americanum -79.5749397 35.7959709 5784

Amblyomma americanum americanum A. americanum -79.5749397 35.7959709 5784

Amblyomma americanum americanum A. americanum -79.5749397 35.7959709 5784

Amblyomma americanum americanum A. americanum -79.5749397 35.7959709 5784

Amblyomma americanum americanum A. americanum -79.5749397 35.7959709 5784

Amblyomma americanum americanum A. americanum -79.5749397 35.7959709 5784

Amblyomma americanum americanum A. americanum -79.5749397 35.7959709 5784

Amblyomma americanum americanum A. americanum -79.5749397 35.7959709 5784

Amblyomma americanum americanum A. americanum -79.5749397 35.7959709 5784

Amblyomma americanum americanum A. americanum -79.5749397 35.7959709 5784

Amblyomma americanum americanum A. americanum -79.5749397 35.7959709 5784

Amblyomma americanum americanum A. americanum -79.5749397 35.7959709 5784

Amblyomma americanum americanum A. americanum -79.5749397 35.7959709 5784

Amblyomma americanum americanum A. americanum -79.5749397 35.7959709 5784

Amblyomma americanum americanum A. americanum -79.5749397 35.7959709 5784

Amblyomma americanum americanum A. americanum -79.5749397 35.7959709 5784

Amblyomma americanum americanum A. americanum -79.5749397 35.7959709 5784

Amblyomma americanum americanum A. americanum -79.5749397 35.7959709 5784

Amblyomma americanum americanum A. americanum -79.5749397 35.7959709 5784

Amblyomma americanum americanum A. americanum -79.5749397 35.7959709 5784

Amblyomma americanum americanum A. americanum -79.5749397 35.7959709 5784

Amblyomma americanum americanum A. americanum -79.5749397 35.7959709 5784

Amblyomma americanum americanum A. americanum -79.5749397 35.7959709 5784

Amblyomma americanum americanum A. americanum -79.5749397 35.7959709 5784

Amblyomma americanum americanum A. americanum -79.5749397 35.7959709 5784

Amblyomma americanum americanum A. americanum -79.5749397 35.7959709 5784

Amblyomma americanum americanum A. americanum -79.5749397 35.7959709 5784

Amblyomma americanum americanum A. americanum -79.5749397 35.7959709 5784

Amblyomma americanum americanum A. americanum -79.5749397 35.7959709 5784

Amblyomma americanum americanum A. americanum -79.5749397 35.7959709 5784

Amblyomma americanum americanum A. americanum -79.5749397 35.7959709 5784

Amblyomma americanum americanum A. americanum -79.5749397 35.7959709 5784

Amblyomma americanum americanum A. americanum -79.5749397 35.7959709 5784

Amblyomma americanum americanum A. americanum -79.5749397 35.7959709 5784

Amblyomma americanum americanum A. americanum -79.5749397 35.7959709 5784

Amblyomma americanum americanum A. americanum -79.5749397 35.7959709 5784

Amblyomma americanum americanum A. americanum -79.5749397 35.7959709 5784

Amblyomma americanum americanum A. americanum -79.5749397 35.7959709 5784

Amblyomma americanum americanum A. americanum -79.5749397 35.7959709 5784

Amblyomma americanum americanum A. americanum -79.5749397 35.7959709 5784

Amblyomma americanum americanum A. americanum -79.5749397 35.7959709 5784

Amblyomma americanum americanum A. americanum -79.5749397 35.7959709 5784

Amblyomma americanum americanum A. americanum -79.5749397 35.7959709 5784

Amblyomma americanum americanum A. americanum -79.5749397 35.7959709 5784

Amblyomma americanum americanum A. americanum -79.5749397 35.7959709 5784

Amblyomma americanum americanum A. americanum -79.5749397 35.7959709 5784

Amblyomma americanum americanum A. americanum -79.5749397 35.7959709 5784

Amblyomma americanum americanum A. americanum -79.5749397 35.7959709 5784

Amblyomma americanum americanum A. americanum -79.5749397 35.7959709 5784

Amblyomma americanum americanum A. americanum -79.5749397 35.7959709 5784

Amblyomma americanum americanum A. americanum -79.5749397 35.7959709 5784

Amblyomma americanum americanum A. americanum -79.5749397 35.7959709 5784

Amblyomma americanum americanum A. americanum -79.5749397 35.7959709 5784

Amblyomma americanum americanum A. americanum -79.5749397 35.7959709 5784

Amblyomma americanum americanum A. americanum -79.5749397 35.7959709 5784

Amblyomma americanum americanum A. americanum -79.5749397 35.7959709 5784

Amblyomma americanum americanum A. americanum -79.5749397 35.7959709 5784

Amblyomma americanum americanum A. americanum -79.5749397 35.7959709 5784

Amblyomma americanum americanum A. americanum -79.5749397 35.7959709 5784

Amblyomma americanum americanum A. americanum -79.5749397 35.7959709 5784

Amblyomma americanum americanum A. americanum -79.5749397 35.7959709 5784

Amblyomma americanum americanum A. americanum -79.5749397 35.7959709 5784

Amblyomma americanum americanum A. americanum -79.5749397 35.7959709 5784

Amblyomma americanum americanum A. americanum -79.5749397 35.7959709 5784

Amblyomma americanum americanum A. americanum -79.5749397 35.7959709 5784

Amblyomma americanum americanum A. americanum -79.5749397 35.7959709 5784

Amblyomma americanum americanum A. americanum -79.5749397 35.7959709 5784

Amblyomma americanum americanum A. americanum -79.5749397 35.7959709 5784

Amblyomma americanum americanum A. americanum -79.5749397 35.7959709 5784

Amblyomma americanum americanum A. americanum -79.5749397 35.7959709 5784

Amblyomma americanum americanum A. americanum -79.5749397 35.7959709 5784

Amblyomma americanum americanum A. americanum -79.5749397 35.7959709 5784

Amblyomma americanum americanum A. americanum -79.5749397 35.7959709 5784

Amblyomma americanum americanum A. americanum -79.5749397 35.7959709 5784

Amblyomma americanum americanum A. americanum -79.5749397 35.7959709 5784

Amblyomma americanum americanum A. americanum -79.5749397 35.7959709 5784

Amblyomma americanum americanum A. americanum -79.5749397 35.7959709 5784

Amblyomma americanum americanum A. americanum -79.5749397 35.7959709 5784

Amblyomma americanum americanum A. americanum -79.5749397 35.7959709 5784

Amblyomma americanum americanum A. americanum -79.5749397 35.7959709 5784

Amblyomma americanum americanum A. americanum -79.5749397 35.7959709 5784

Amblyomma americanum americanum A. americanum -79.5749397 35.7959709 5784

Amblyomma americanum americanum A. americanum -79.5749397 35.7959709 5784

Amblyomma americanum americanum A. americanum -79.5749397 35.7959709 5784

Amblyomma americanum americanum A. americanum -79.5749397 35.7959709 5784

Amblyomma americanum americanum A. americanum -79.5749397 35.7959709 5784

Amblyomma americanum americanum A. americanum -79.5749397 35.7959709 5784

Amblyomma americanum americanum A. americanum -79.5749397 35.7959709 5784

Amblyomma americanum americanum A. americanum -79.5749397 35.7959709 5784

Amblyomma americanum americanum A. americanum -79.5749397 35.7959709 5784

Amblyomma americanum americanum A. americanum -79.5749397 35.7959709 5784

Amblyomma americanum americanum A. americanum -79.5749397 35.7959709 5784

Amblyomma americanum americanum A. americanum -79.5749397 35.7959709 5784

Amblyomma americanum americanum A. americanum -79.5749397 35.7959709 5784

Amblyomma americanum americanum A. americanum -79.5749397 35.7959709 5784

Amblyomma americanum americanum A. americanum -79.5749397 35.7959709 5784

Amblyomma americanum americanum A. americanum -79.5749397 35.7959709 5784

Amblyomma americanum americanum A. americanum -79.5749397 35.7959709 5784

Amblyomma americanum americanum A. americanum -77.4286003 39.0062199 5062

Amblyomma americanum americanum A. americanum -77.4286003 39.0062199 5062

Amblyomma americanum americanum A. americanum -76.379406 39.6689949 5062

Amblyomma americanum americanum A. americanum -76.379406 39.6689949 5062

Amblyomma americanum americanum A. americanum -76.5835648 36.7282047 5063

Amblyomma americanum americanum A. americanum -76.5835648 36.7282047 5063

Amblyomma americanum americanum A. americanum -76.5835648 36.7282047 5063

Amblyomma americanum americanum A. americanum -76.5835648 36.7282047 5063

Amblyomma americanum americanum A. americanum -76.5835648 36.7282047 5063

Amblyomma americanum americanum A. americanum -74.10083 40.30417 8839

Amblyomma americanum americanum A. americanum -76.8041267 37.3798676 5062

Amblyomma americanum americanum A. americanum -76.8041267 37.3798676 5062

Amblyomma americanum americanum A. americanum -76.8041267 37.3798676 5062

Amblyomma americanum americanum A. americanum -95.0877495 39.109724 5062

Amblyomma americanum americanum A. americanum -95.0877495 39.109724 5062

Amblyomma americanum americanum A. americanum -95.67778 39.04833 10539

Amblyomma americanum americanum A. americanum -87.26278 36.72389 1310

Amblyomma americanum americanum A. americanum -74.74333 40.21694 5227

Amblyomma americanum americanum A. americanum -77.3366508 38.5467854 5062

Amblyomma americanum americanum A. americanum -77.3366508 38.5467854 5062

Amblyomma americanum americanum A. americanum -77.3366508 38.5467854 5062

Amblyomma americanum americanum A. americanum -77.3366508 38.5467854 5062

Amblyomma americanum americanum A. americanum -77.3366508 38.5467854 5062

Amblyomma americanum americanum A. americanum -77.3366508 38.5467854 5062

Amblyomma americanum americanum A. americanum -77.3366508 38.5467854 5062

Amblyomma americanum americanum A. americanum -81.66583 28.92917 3652

Amblyomma americanum americanum A. americanum -80.00032 35.50069 219000

Amblyomma americanum americanum A. americanum -78.2933696 34.1051869 42199

Amblyomma americanum americanum A. americanum -76.749691 38.8159466 5062

Amblyomma americanum americanum A. americanum -76.483236 38.982047 500

Amblyomma americanum americanum A. americanum -95.46 39.34333 1606

Amblyomma americanum americanum A. americanum -79.1975 37.90611 3036

Amblyomma americanum americanum A. americanum -79.1975 37.90611 3036

Amblyomma americanum americanum A. americanum -77.2652626 38.9012184 5062

Amblyomma americanum americanum A. americanum -77.2652626 38.9012184 5062

Amblyomma americanum americanum A. americanum -77.2652626 38.9012184 5062

Amblyomma americanum americanum A. americanum -77.2652626 38.9012184 5062

Amblyomma americanum americanum A. americanum -74.7484894 39.9340038 5062

Amblyomma americanum americanum A. americanum -85.9813499 37.8100662 5062

Amblyomma americanum americanum A. americanum -75.9998902 36.8525752 5783

Amblyomma americanum americanum A. americanum -78.5097198 35.9765678 5372

Amblyomma americanum americanum A. americanum -76.9391365 38.620069 5427

Amblyomma americanum americanum A. americanum -76.9391365 38.620069 5427

Amblyomma americanum americanum A. americanum -76.9391365 38.620069 5427

Amblyomma americanum americanum A. americanum -76.9391365 38.620069 5427

Amblyomma americanum americanum A. americanum -76.9391365 38.620069 5427

Amblyomma americanum americanum A. americanum -76.9391365 38.620069 5427

Amblyomma americanum americanum A. americanum -76.9391365 38.620069 5427

Amblyomma americanum americanum A. americanum -76.9391365 38.620069 5427

Amblyomma americanum americanum A. americanum -76.9391365 38.620069 5427

Amblyomma americanum americanum A. americanum -77.795269 38.7134495 5062

Amblyomma americanum americanum A. americanum -77.795269 38.7134495 5062

Amblyomma americanum americanum A. americanum -77.0352237 38.8958606 5123

Amblyomma americanum americanum A. americanum -77.0352237 38.8958606 5123

Amblyomma americanum americanum A. americanum -77.0352237 38.8958606 5123

Amblyomma americanum americanum A. americanum -77.795269 38.7134495 5062

Amblyomma americanum americanum A. americanum -92.2007294 37.8286514 5062

Amblyomma americanum americanum A. americanum -92.2007294 37.8286514 5062

Amblyomma americanum americanum A. americanum -92.2007294 37.8286514 5062

Amblyomma americanum americanum A. americanum -92.2007294 37.8286514 5062

Amblyomma americanum americanum A. americanum -92.2007294 37.8286514 5062

Amblyomma americanum americanum A. americanum -92.2007294 37.8286514 5062

Amblyomma americanum americanum A. americanum -92.2007294 37.8286514 5062

Amblyomma americanum americanum A. americanum -92.2007294 37.8286514 5062

Amblyomma americanum americanum A. americanum -92.2007294 37.8286514 5062

Amblyomma americanum americanum A. americanum -92.2007294 37.8286514 5062

Amblyomma americanum americanum A. americanum -92.2007294 37.8286514 5062

Amblyomma americanum americanum A. americanum -92.2007294 37.8286514 5062

Amblyomma americanum americanum A. americanum -92.2007294 37.8286514 5062

Amblyomma americanum americanum A. americanum -96.41333 39.39389 1034

Amblyomma americanum americanum A. americanum -94.9012875 39.4115531 5105

Amblyomma americanum americanum A. americanum -76.9402542 38.5903988 5062

Amblyomma americanum americanum A. americanum -74.3784866 39.9545612 5062

Amblyomma americanum americanum A. americanum -74.3784866 39.9545612 5062

Amblyomma americanum americanum A. americanum -74.3784866 39.9545612 5062

Amblyomma americanum americanum A. americanum -97.3375435 37.692234 5062

Amblyomma americanum americanum A. americanum -76.7074547 37.2707005 5062

Amblyomma americanum americanum A. americanum -76.7074547 37.2707005 5062

Amblyomma americanum americanum A. americanum -76.7074547 37.2707005 5062

Amblyomma americanum americanum A. americanum -76.7074547 37.2707005 5062

Amblyomma americanum americanum A. americanum -76.7074547 37.2707005 5062

Amblyomma americanum americanum A. americanum -76.7074547 37.2707005 5062

Amblyomma americanum americanum A. americanum -76.7074547 37.2707005 5062

Amblyomma americanum americanum A. americanum -76.7074547 37.2707005 5062

Amblyomma americanum americanum A. americanum -76.7074547 37.2707005 5062

Amblyomma americanum americanum A. americanum -76.7074547 37.2707005 5062

Amblyomma americanum americanum A. americanum -76.7074547 37.2707005 5062

Amblyomma americanum americanum A. americanum -76.7074547 37.2707005 5062

Amblyomma americanum americanum A. americanum -76.7074547 37.2707005 5062

Amblyomma americanum americanum A. americanum -76.7074547 37.2707005 5062

Amblyomma americanum americanum A. americanum -76.7074547 37.2707005 5062

Amblyomma americanum americanum A. americanum -76.7074547 37.2707005 5062

Amblyomma americanum americanum A. americanum -76.7074547 37.2707005 5062

Amblyomma americanum americanum A. americanum -76.7074547 37.2707005 5062

Amblyomma americanum americanum A. americanum -76.7074547 37.2707005 5062

Amblyomma americanum americanum A. americanum -76.7074547 37.2707005 5062

Amblyomma americanum americanum A. americanum -76.7074547 37.2707005 5062

Amblyomma americanum americanum A. americanum -76.7074547 37.2707005 5062

Amblyomma americanum americanum A. americanum -76.7074547 37.2707005 5062

Amblyomma americanum americanum A. americanum -76.7074547 37.2707005 5062

Amblyomma americanum americanum A. americanum -76.7074547 37.2707005 5062

Amblyomma americanum americanum A. americanum -76.7074547 37.2707005 5062

Amblyomma americanum americanum A. americanum -77.944706 34.2257252 5063

Amblyomma americanum americanum A. americanum -78.1633339 39.1856613 5062

Amblyomma americanum americanum A. americanum -81.08667 34.38056 3409

Amblyomma americanum americanum A. americanum -77.2671266 38.6540242 4893

Amblyomma americanum americanum A. americanum -81.72278 30.96361 3466

Amblyomma americanum americanum A. americanum -77.2671266 38.6540242 4893

Amblyomma americanum americanum A. americanum -77.2671266 38.6540242 4893

Amblyomma americanum americanum A. americanum -77.2671266 38.6540242 4893

Amblyomma americanum americanum A. americanum -77.2671266 38.6540242 4893

Amblyomma americanum americanum A. americanum -77.2671266 38.6540242 4893

Amblyomma americanum americanum A. americanum -77.2671266 38.6540242 4893

Amblyomma americanum americanum A. americanum -77.2671266 38.6540242 4893

Amblyomma americanum americanum A. americanum -77.2671266 38.6540242 4893

Amblyomma americanum americanum A. americanum -77.2671266 38.6540242 4893

Amblyomma americanum americanum A. americanum -77.2671266 38.6540242 4893

Amblyomma americanum americanum A. americanum -77.2671266 38.6540242 4893

Amblyomma americanum americanum A. americanum -77.2671266 38.6540242 4893

Amblyomma americanum americanum A. americanum -77.2671266 38.6540242 4893

Amblyomma americanum americanum A. americanum -77.2671266 38.6540242 4893

Amblyomma americanum americanum A. americanum -77.2671266 38.6540242 4893

Amblyomma americanum americanum A. americanum -77.2671266 38.6540242 4893

Amblyomma americanum americanum A. americanum -77.2671266 38.6540242 4893

Amblyomma americanum americanum A. americanum -77.2671266 38.6540242 4893

Amblyomma americanum americanum A. americanum -77.2671266 38.6540242 4893

Amblyomma americanum americanum A. americanum -77.2671266 38.6540242 4893

Amblyomma americanum americanum A. americanum -77.2671266 38.6540242 4893

Amblyomma americanum americanum A. americanum -77.2671266 38.6540242 4893

Amblyomma americanum americanum A. americanum -77.2671266 38.6540242 4893

Amblyomma americanum americanum A. americanum -77.2671266 38.6540242 4893

Amblyomma americanum americanum A. americanum -77.2671266 38.6540242 4893

Amblyomma americanum americanum A. americanum -77.2671266 38.6540242 4893

Amblyomma americanum americanum A. americanum -77.2671266 38.6540242 4893

Amblyomma americanum americanum A. americanum -77.2671266 38.6540242 4893

Amblyomma americanum americanum A. americanum -77.2671266 38.6540242 4893

Amblyomma americanum americanum A. americanum -77.2671266 38.6540242 4893

Amblyomma americanum americanum A. americanum -77.2671266 38.6540242 4893

Amblyomma americanum americanum A. americanum -77.2671266 38.6540242 4893

Amblyomma americanum americanum A. americanum -77.2671266 38.6540242 4893

Amblyomma americanum americanum A. americanum -77.2671266 38.6540242 4893

Amblyomma americanum americanum A. americanum -77.2671266 38.6540242 4893

Amblyomma americanum americanum A. americanum -77.2671266 38.6540242 4893

Amblyomma americanum americanum A. americanum -77.2671266 38.6540242 4893

Amblyomma americanum americanum A. americanum -77.2671266 38.6540242 4893

Amblyomma americanum americanum A. americanum -77.2671266 38.6540242 4893

Amblyomma americanum americanum A. americanum -77.2671266 38.6540242 4893

Amblyomma americanum americanum A. americanum -87.5105705 36.5442162 5063

Amblyomma americanum americanum A. americanum -87.5105705 36.5442162 5063

Amblyomma americanum americanum A. americanum -87.5105705 36.5442162 5063

Amblyomma americanum americanum A. americanum -87.5105705 36.5442162 5063

Amblyomma americanum americanum A. americanum -87.5105705 36.5442162 5063

Amblyomma americanum americanum A. americanum -87.5105705 36.5442162 5063

Amblyomma americanum americanum A. americanum -87.5105705 36.5442162 5063

Amblyomma americanum americanum A. americanum -87.5105705 36.5442162 5063

Amblyomma americanum americanum A. americanum -87.5105705 36.5442162 5063

Amblyomma americanum americanum A. americanum -87.5105705 36.5442162 5063

Amblyomma americanum americanum A. americanum -77.2671266 38.6540242 4893

Amblyomma americanum americanum A. americanum -76.5125753 37.2304516 5499

Amblyomma americanum americanum A. americanum -76.5125753 37.2304516 5499

Amblyomma americanum americanum A. americanum -76.5125753 37.2304516 5499

Amblyomma americanum americanum A. americanum -76.5125753 37.2304516 5499

Amblyomma americanum americanum A. americanum -76.5125753 37.2304516 5499

Amblyomma americanum americanum A. americanum -76.5125753 37.2304516 5499

Amblyomma americanum americanum A. americanum -76.5125753 37.2304516 5499

Amblyomma americanum americanum A. americanum -76.5125753 37.2304516 5499

Amblyomma americanum americanum A. americanum -76.5125753 37.2304516 5499

Amblyomma americanum americanum A. americanum -78.47472 36.02472 1822

Amblyomma americanum americanum A. americanum -77.39319 34.76305 28000

Amblyomma americanum americanum A. americanum -76.5676 38.99416 27000

Amblyomma americanum americanum A. americanum -77.27622 38.83469 28000

Amblyomma americanum americanum A. americanum -77.44675 37.54812 465000

Amblyomma americanum americanum A. americanum -81.95966 36.72448 34500

Amblyomma americanum americanum A. americanum -86.25027 35.75035 36300

Amblyomma americanum americanum A. americanum -78.86931 35.3686 31000

Amblyomma americanum americanum A. americanum -76.84729 38.82952 35000

Amblyomma americanum americanum A. americanum -86.25027 35.75035 36300

Amblyomma americanum americanum A. americanum -75.94811 39.56242 35000

Amblyomma americanum americanum A. americanum -75.94811 39.56242 35000

Amblyomma americanum americanum A. americanum -77.27622 38.83469 28000

Amblyomma americanum americanum A. americanum -77.27622 38.83469 28000

Amblyomma americanum americanum A. americanum -77.27622 38.83469 28000

Amblyomma americanum americanum A. americanum -78.44108 37.2243 25000

Amblyomma americanum americanum A. americanum -77.44675 37.54812 465000

Amblyomma americanum americanum A. americanum -78.82744 35.04859 40000

Amblyomma americanum americanum A. americanum -76.90567 40.27245 260000

Amblyomma americanum americanum A. americanum -76.74997 39.00039 228000

Amblyomma americanum americanum A. americanum -94.82226 38.88376 27500

Amblyomma americanum americanum A. americanum -94.82226 38.88376 27500

Amblyomma americanum americanum A. americanum -77.63572 39.09068 29000

Amblyomma americanum americanum A. americanum -77.27622 38.83469 28000

Amblyomma americanum americanum A. americanum -76.29887 39.53644 26000

Amblyomma americanum americanum A. americanum -77.27622 38.83469 28000

Amblyomma americanum americanum A. americanum -76.74997 39.00039 228000

Amblyomma americanum americanum A. americanum -77.65597 38.18502 23000

Amblyomma americanum americanum A. americanum -83.50018 32.75042 292500

Amblyomma americanum americanum A. americanum -79.52422 37.31494 31000

Amblyomma americanum americanum A. americanum -77.58694 37.37852 28000

Amblyomma americanum americanum A. americanum -80.00032 35.50069 458000

Amblyomma americanum americanum A. americanum -74.6682 39.87769 45000

Amblyomma americanum americanum A. americanum -80.00032 35.50069 458000

Amblyomma americanum americanum A. americanum -77.44675 37.54812 465000

Amblyomma americanum americanum A. americanum -77.44675 37.54812 465000

Amblyomma americanum americanum A. americanum -79.48131 35.31072 40000

Amblyomma americanum americanum A. americanum -76.5676 38.99416 27000

Amblyomma americanum americanum A. americanum -77.44675 37.54812 465000

Amblyomma americanum americanum A. americanum -111.50098 34.5003 453000

Amblyomma americanum americanum A. americanum -77.80927 38.73855 38000

Amblyomma americanum americanum A. americanum -75.94811 39.56242 35000

Amblyomma americanum americanum A. americanum -77.44675 37.54812 465000

Amblyomma americanum americanum A. americanum -77.27622 38.83469 28000

Amblyomma americanum americanum A. americanum -80.38232 33.91617 41500

Amblyomma americanum americanum A. americanum -80.38232 33.91617 41500

Amblyomma americanum americanum A. americanum -83.50018 32.75042 292500

Amblyomma americanum americanum A. americanum -79.39951 36.04407 29000

Amblyomma americanum americanum A. americanum -87.38289 36.49686 27500

Amblyomma americanum americanum A. americanum -76.09582 39.2356 27500

Amblyomma americanum americanum A. americanum -77.80927 38.73855 38000

Amblyomma americanum americanum A. americanum -77.00025 38.91706 11000

Amblyomma americanum americanum A. americanum -88.2632 32.01961 42000

Amblyomma americanum americanum A. americanum -77.47766 38.70167 27500

Amblyomma americanum americanum A. americanum -77.27622 38.83469 28000

Amblyomma americanum americanum A. americanum -77.27622 38.83469 28000

Amblyomma americanum americanum A. americanum -93.77598 38.03718 32500

Amblyomma americanum americanum A. americanum -77.47766 38.70167 27500

Amblyomma americanum americanum A. americanum -95.0379 39.19932 29000

Amblyomma americanum americanum A. americanum -79.25535 35.70258 39000

Amblyomma americanum americanum A. americanum -98.50063 38.50029 376000

Amblyomma americanum americanum A. americanum -86.25027 35.75035 36300

Amblyomma americanum americanum A. americanum -84.87575 34.50336 24000

Amblyomma americanum americanum A. americanum -77.47766 38.70167 27500

Amblyomma americanum americanum A. americanum -76.84729 38.82952 35000

Amblyomma americanum americanum A. americanum -98.50063 38.50029 376000

Amblyomma americanum americanum A. americanum -77.27622 38.83469 28000

Amblyomma americanum americanum A. americanum -83.50018 32.75042 292500

Amblyomma americanum americanum A. americanum -92.20766 37.82463 31000

Amblyomma americanum americanum A. americanum -77.63572 39.09068 29000

Amblyomma americanum americanum A. americanum -77.47766 38.70167 27500

Amblyomma americanum americanum A. americanum -77.27622 38.83469 28000

Amblyomma americanum americanum A. americanum -76.84729 38.82952 35000

Amblyomma americanum americanum A. americanum -75.14167 39.71731 26000

Amblyomma americanum americanum A. americanum -87.52503 33.28955 35000

Amblyomma americanum americanum A. americanum -77.72127 39.92742 41000

Amblyomma americanum americanum A. americanum -77.27622 38.83469 28000

Amblyomma americanum americanum A. americanum -77.27622 38.83469 28000

Amblyomma americanum americanum A. americanum -76.29887 39.53644 26000

Amblyomma americanum americanum A. americanum -92.50046 38.25031 367000

Amblyomma americanum americanum A. americanum -77.45743 38.4207 21500

Amblyomma americanum americanum A. americanum -83.56052 33.02513 25500

Amblyomma americanum americanum A. americanum -75.49992 39.00039 91500

Amblyomma americanum americanum A. americanum -81.40989 29.91218 24000

Amblyomma americanum americanum A. americanum -76.74997 39.00039 228000

Amblyomma americanum americanum A. americanum -86.17152 30.61847 37000

Amblyomma americanum americanum A. americanum -76.70913 36.90673 31000

Amblyomma americanum americanum A. americanum -77.58694 37.37852 28000

Amblyomma americanum americanum No data -77.33167 34.67922 34

Amblyomma americanum americanum No data -77.33167 34.67922 34

Amblyomma americanum americanum No data -77.33167 34.67922 34

Amblyomma americanum americanum No data -77.33167 34.67922 34

Amblyomma americanum americanum No data -77.33167 34.67922 34

Amblyomma americanum americanum No data -77.33167 34.67922 34

Amblyomma americanum americanum No data -77.33167 34.67922 34

Amblyomma americanum americanum No data -77.33167 34.67922 34

Amblyomma americanum americanum No data -77.33167 34.67922 34

Amblyomma americanum americanum No data -77.33167 34.67922 34

Amblyomma americanum americanum No data -77.33167 34.67922 34

Amblyomma americanum americanum No data -77.33167 34.67922 34

Amblyomma americanum americanum No data -77.33167 34.67922 34

Amblyomma americanum americanum No data -77.33167 34.67922 34

Amblyomma americanum americanum No data -77.33167 34.67922 34

Amblyomma americanum americanum No data -77.33167 34.67922 34

Amblyomma americanum americanum No data -77.33167 34.67922 34

Amblyomma americanum americanum No data -77.33167 34.67922 34

Amblyomma americanum americanum No data -77.3719 34.71877 34

Amblyomma americanum americanum No data -77.3719 34.71877 34

Amblyomma americanum americanum No data -77.3719 34.71877 34

Amblyomma americanum americanum No data -77.3719 34.71877 34

Amblyomma americanum americanum No data -77.3719 34.71877 34

Amblyomma americanum americanum No data -77.3719 34.71877 34

Amblyomma americanum americanum No data -77.3719 34.71877 34

Amblyomma americanum americanum No data -77.3719 34.71877 34

Amblyomma americanum americanum No data -77.3719 34.71877 34

Amblyomma americanum americanum No data -77.3719 34.71877 34

Amblyomma americanum americanum No data -77.3719 34.71877 34

Amblyomma americanum americanum No data -77.37261111 34.71966667 34

Amblyomma americanum americanum No data -77.37261111 34.71966667 34

Amblyomma americanum americanum No data -77.37261111 34.71966667 34

Amblyomma americanum americanum No data -77.37261111 34.71966667 34

Amblyomma americanum americanum No data -77.37261111 34.71966667 34

Amblyomma americanum americanum No data -77.37261111 34.71966667 34

Amblyomma americanum americanum No data -77.37261111 34.71966667 34

Amblyomma americanum americanum No data -77.37261111 34.71966667 34

Amblyomma americanum americanum No data -77.37261111 34.71966667 34

Amblyomma americanum americanum No data -77.37261111 34.71966667 34

Amblyomma americanum americanum No data -77.37261111 34.71966667 34

Amblyomma americanum americanum No data -77.37261111 34.71966667 34

Amblyomma americanum americanum No data -77.37261111 34.71966667 34

Amblyomma americanum americanum No data -77.37261111 34.71966667 34

Amblyomma americanum americanum No data -77.37261111 34.71966667 34

Amblyomma americanum americanum No data -77.37261111 34.71966667 34

Amblyomma americanum americanum No data -77.37261111 34.71966667 34

Amblyomma americanum americanum No data -77.37261111 34.71966667 34

Amblyomma americanum americanum No data -77.37261111 34.71966667 34

Amblyomma americanum americanum No data -77.37261111 34.71966667 34

Amblyomma americanum americanum No data -77.37261111 34.71966667 34

Amblyomma americanum americanum No data -77.37261111 34.71966667 34

Amblyomma americanum americanum No data -77.37261111 34.71966667 34

Amblyomma americanum americanum No data -77.37261111 34.71966667 34

Amblyomma americanum americanum No data -77.37261111 34.71966667 34

Amblyomma americanum americanum No data -77.37261111 34.71966667 34

Amblyomma americanum americanum No data -77.37261111 34.71966667 34

Amblyomma americanum americanum No data -77.37261111 34.71966667 34

Amblyomma americanum americanum No data -77.37261111 34.71966667 34

Amblyomma americanum americanum No data -77.37261111 34.71966667 34

Amblyomma americanum americanum No data -77.37261111 34.71966667 34

Amblyomma americanum americanum No data -77.37261111 34.71966667 34

Amblyomma americanum americanum No data -77.37261111 34.71966667 34

Amblyomma americanum americanum No data -77.37261111 34.71966667 34

Amblyomma americanum americanum No data -77.37261111 34.71966667 34

Amblyomma americanum americanum No data -77.33227778 34.68036111 34

Amblyomma americanum americanum No data -77.33227778 34.68036111 34

Amblyomma americanum americanum No data -77.33227778 34.68036111 34

Amblyomma americanum americanum No data -77.33227778 34.68036111 34

Amblyomma americanum americanum No data -77.33227778 34.68036111 34

Amblyomma americanum americanum No data -77.33227778 34.68036111 34

Amblyomma americanum americanum No data -77.33227778 34.68036111 34

Amblyomma americanum americanum No data -77.33227778 34.68036111 34

Amblyomma americanum americanum No data -77.33227778 34.68036111 34

Amblyomma americanum americanum No data -77.33227778 34.68036111 34

Amblyomma americanum americanum No data -77.33227778 34.68036111 34

Amblyomma americanum americanum No data -77.33227778 34.68036111 34

Amblyomma americanum americanum No data -77.33227778 34.68036111 34

Amblyomma americanum americanum No data -77.33227778 34.68036111 34

Amblyomma americanum americanum No data -77.33227778 34.68036111 34

Amblyomma americanum americanum No data -77.33227778 34.68036111 34

Amblyomma americanum americanum No data -77.33227778 34.68036111 34

Amblyomma americanum americanum No data -77.33227778 34.68036111 34

Amblyomma americanum americanum No data -77.35502778 34.71933333 34

Amblyomma americanum americanum No data -77.35502778 34.71933333 34

Amblyomma americanum americanum No data -77.35502778 34.71933333 34

Amblyomma americanum americanum No data -77.35502778 34.71933333 34

Amblyomma americanum americanum No data -77.35502778 34.71933333 34

Amblyomma americanum americanum No data -77.35502778 34.71933333 34

Amblyomma americanum americanum No data -77.35502778 34.71933333 34

Amblyomma americanum americanum No data -77.35502778 34.71933333 34

Amblyomma americanum americanum No data -77.35502778 34.71933333 34

Amblyomma americanum americanum No data -77.35502778 34.71933333 34

Amblyomma americanum americanum No data -77.35502778 34.71933333 34

Amblyomma americanum americanum No data -77.35502778 34.71933333 34

Amblyomma americanum americanum No data -77.35502778 34.71933333 34

Amblyomma americanum americanum No data -77.37527778 34.72202778 34

Amblyomma americanum americanum No data -77.3455 34.68361111 34

Amblyomma americanum americanum No data -77.3455 34.68361111 34

Amblyomma americanum americanum No data -77.3455 34.68361111 34

Amblyomma americanum americanum No data -77.34172222 34.65230556 34

Amblyomma americanum americanum No data -77.34172222 34.65230556 34

Amblyomma americanum americanum No data -77.37261111 34.71966667 34

Amblyomma americanum americanum No data -77.37261111 34.71966667 34

Amblyomma americanum americanum No data -77.37261111 34.71966667 34

Amblyomma americanum americanum No data -77.37261111 34.71966667 34

Amblyomma americanum americanum No data -77.37261111 34.71966667 34

Amblyomma americanum americanum No data -77.37261111 34.71966667 34

Amblyomma americanum americanum No data -77.37261111 34.71966667 34

Amblyomma americanum americanum No data -77.37261111 34.71966667 34

Amblyomma americanum americanum No data -77.37261111 34.71966667 34

Amblyomma americanum americanum No data -77.37261111 34.71966667 34

Amblyomma americanum americanum No data -77.37261111 34.71966667 34

Amblyomma americanum americanum No data -77.37261111 34.71966667 34

Amblyomma americanum americanum No data -77.37261111 34.71966667 34

Amblyomma americanum americanum No data -77.37261111 34.71966667 34

Amblyomma americanum americanum No data -77.37261111 34.71966667 34

Amblyomma americanum americanum No data -77.37261111 34.71966667 34

Amblyomma americanum americanum No data -77.37261111 34.71966667 34

Amblyomma americanum americanum No data -77.37261111 34.71966667 34

Amblyomma americanum americanum No data -77.37261111 34.71966667 34

Amblyomma americanum americanum No data -77.37261111 34.71966667 34

Amblyomma americanum americanum No data -77.37261111 34.71966667 34

Amblyomma americanum americanum No data -77.37261111 34.71966667 34

Amblyomma americanum americanum No data -77.37261111 34.71966667 34

Amblyomma americanum americanum No data -77.37261111 34.71966667 34

Amblyomma americanum americanum No data -77.37261111 34.71966667 34

Amblyomma americanum americanum No data -77.37261111 34.71966667 34

Amblyomma americanum americanum No data -77.37261111 34.71966667 34

Amblyomma americanum americanum No data -77.37261111 34.71966667 34

Amblyomma americanum americanum No data -77.37261111 34.71966667 34

Amblyomma americanum americanum No data -77.37261111 34.71966667 34

Amblyomma americanum americanum No data -77.37261111 34.71966667 34

Amblyomma americanum americanum No data -77.37261111 34.71966667 34

Amblyomma americanum americanum No data -77.37261111 34.71966667 34

Amblyomma americanum americanum No data -77.37261111 34.71966667 34

Amblyomma americanum americanum No data -77.37261111 34.71966667 34

Amblyomma americanum americanum No data -77.37261111 34.71966667 34

Amblyomma americanum americanum No data -77.37261111 34.71966667 34

Amblyomma americanum americanum No data -77.36536111 34.71997222 34

Amblyomma americanum americanum No data -77.36536111 34.71997222 34

Amblyomma americanum americanum No data -77.36536111 34.71997222 34

Amblyomma americanum americanum No data -77.36536111 34.71997222 34

Amblyomma americanum americanum No data -77.36536111 34.71997222 34

Amblyomma americanum americanum No data -77.36536111 34.71997222 34

Amblyomma americanum americanum No data -77.36536111 34.71997222 34

Amblyomma americanum americanum No data -77.36536111 34.71997222 34

Amblyomma americanum americanum No data -77.36536111 34.71997222 34

Amblyomma americanum americanum No data -77.36536111 34.71997222 34

Amblyomma americanum americanum No data -77.37319444 34.71919444 34

Amblyomma americanum americanum No data -77.37319444 34.71919444 34

Amblyomma americanum americanum No data -77.37319444 34.71919444 34

Amblyomma americanum americanum No data -77.37319444 34.71919444 34

Amblyomma americanum americanum No data -77.37319444 34.71919444 34

Amblyomma americanum americanum No data -77.37319444 34.71919444 34

Amblyomma americanum americanum No data -77.37319444 34.71919444 34

Amblyomma americanum americanum No data -77.37319444 34.71919444 34

Amblyomma americanum americanum No data -77.37319444 34.71919444 34

Amblyomma americanum americanum No data -77.37319444 34.71919444 34

Amblyomma americanum americanum No data -77.37319444 34.71919444 34

Amblyomma americanum americanum No data -77.37319444 34.71919444 34

Amblyomma americanum americanum No data -77.37319444 34.71919444 34

Amblyomma americanum americanum No data -77.37319444 34.71919444 34

Amblyomma americanum americanum No data -77.37319444 34.71919444 34

Amblyomma americanum americanum No data -77.36536111 34.71997222 34

Amblyomma americanum americanum No data -77.36536111 34.71997222 34

Amblyomma americanum americanum No data -77.36536111 34.71997222 34

Amblyomma americanum americanum No data -77.36536111 34.71997222 34

Amblyomma americanum americanum No data -77.36536111 34.71997222 34

Amblyomma americanum americanum No data -77.36536111 34.71997222 34

Amblyomma americanum americanum No data -77.36536111 34.71997222 34

Amblyomma americanum americanum No data -77.36536111 34.71997222 34

Amblyomma americanum americanum No data -77.36536111 34.71997222 34

Amblyomma americanum americanum No data -77.36536111 34.71997222 34

Amblyomma americanum americanum No data -77.36536111 34.71997222 34

Amblyomma americanum americanum No data -77.36536111 34.71997222 34

Amblyomma americanum americanum No data -77.36536111 34.71997222 34

Amblyomma americanum americanum No data -77.36536111 34.71997222 34

Amblyomma americanum americanum No data -77.36536111 34.71997222 34

Amblyomma americanum americanum No data -77.36536111 34.71997222 34

Amblyomma americanum americanum No data -77.36536111 34.71997222 34

Amblyomma americanum americanum No data -77.36536111 34.71997222 34

Amblyomma americanum americanum No data -77.36536111 34.71997222 34

Amblyomma americanum americanum No data -77.36536111 34.71997222 34

Amblyomma americanum americanum No data -77.36536111 34.71997222 34

Amblyomma americanum americanum No data -77.36536111 34.71997222 34

Amblyomma americanum americanum No data -77.35566667 34.71008333 34

Amblyomma americanum americanum No data -77.35566667 34.71008333 34

Amblyomma americanum americanum No data -77.35566667 34.71008333 34

Amblyomma americanum americanum No data -77.35566667 34.71008333 34

Amblyomma americanum americanum No data -77.35566667 34.71008333 34

Amblyomma americanum americanum No data -77.35566667 34.71008333 34

Amblyomma americanum americanum No data -77.35566667 34.71008333 34

Amblyomma americanum americanum No data -77.35566667 34.71008333 34

Amblyomma americanum americanum No data -77.35566667 34.71008333 34

Amblyomma americanum americanum No data -77.35566667 34.71008333 34

Amblyomma americanum americanum No data -77.35566667 34.71008333 34

Amblyomma americanum americanum No data -77.35566667 34.71008333 34

Amblyomma americanum americanum No data -77.35566667 34.71008333 34

Amblyomma americanum americanum No data -77.35566667 34.71008333 34

Amblyomma americanum americanum No data -77.35566667 34.71008333 34

Amblyomma americanum americanum No data -77.35566667 34.71008333 34

Amblyomma americanum americanum No data -77.35566667 34.71008333 34

Amblyomma americanum americanum No data -77.35566667 34.71008333 34

Amblyomma americanum americanum No data -77.35566667 34.71008333 34

Amblyomma americanum americanum No data -77.35566667 34.71008333 34

Amblyomma americanum americanum No data -77.35566667 34.71008333 34

Amblyomma americanum americanum No data -77.35566667 34.71008333 34

Amblyomma americanum americanum A. americanum -78.8881989 35.0527 6468

Amblyomma americanum americanum A. americanum -77.915039 37.0420244 10500

Amblyomma americanum americanum A. americanum -77.915039 37.0420244 10500

Amblyomma americanum americanum A. americanum -77.915039 37.0420244 10500

Amblyomma americanum americanum A. americanum -84.9670713 32.4614086 5776

Amblyomma americanum americanum A. americanum -78.8881989 35.0527 6468

Amblyomma americanum americanum A. americanum -98.3903313 34.6086864 5063

Amblyomma americanum americanum A. americanum -83.6035042 33.6531773 5000

Amblyomma americanum americanum A. americanum -76.5125753 37.2304516 5499

Amblyomma americanum americanum A. americanum -87.3594513 36.5297699 5063

Amblyomma americanum americanum A. americanum -87.3594513 36.5297699 5063

Amblyomma americanum americanum A. americanum -87.3594513 36.5297699 5063

Amblyomma americanum americanum A. americanum -82.3248253 29.6516342 5064

Amblyomma americanum americanum A. americanum -76.2959883 39.4187183 5189

Amblyomma americanum americanum A. americanum -77.1690739 34.7661703 5041

Amblyomma americanum americanum A. americanum -77.1735725 35.8348827 5063

Amblyomma americanum americanum A. americanum -92.2997268 34.757454 4813

Amblyomma americanum americanum A. americanum -78.244442 35.513216 5063

Amblyomma americanum americanum A. americanum -85.9474654 37.8403473 5198

Amblyomma americanum americanum A. americanum -76.5835648 36.7282047 5063

Amblyomma americanum americanum A. americanum -76.9391365 38.620069 5427

Amblyomma americanum americanum A. americanum -77.5891571 38.2009659 5062

Amblyomma americanum americanum A. americanum -76.2959883 39.4187183 5189

Amblyomma americanum americanum A. americanum -83.4612923 37.7720337 5062

Amblyomma americanum americanum A. americanum -74.1979218 39.9537296 5062

Amblyomma americanum americanum A. americanum -90.8700101 37.9736648 32898

Amblyomma americanum americanum A. americanum -76.5028111 37.0918568 16209

Amblyomma americanum americanum A. americanum -76.6077232 36.0598271 5250

Amblyomma americanum americanum A. americanum -76.3456386 37.0353572 5490

Amblyomma americanum americanum A. americanum -77.0283089 38.6676178 5062

Amblyomma americanum americanum A. americanum -87.3594513 36.5297699 5063

Amblyomma americanum americanum A. americanum -78.2933696 34.1051869 42199

Amblyomma americanum americanum A. americanum -76.5028111 37.0918568 16209

Amblyomma americanum americanum A. americanum -84.8607864 35.6920128 5063

Amblyomma americanum americanum A. americanum -77.5891571 38.2009659 5062

Amblyomma americanum americanum A. americanum -75.5243645 39.158165 5062

Amblyomma americanum americanum A. americanum -87.3594513 36.5297699 5063

Amblyomma americanum americanum A. americanum -76.5125753 37.2304516 5499

Amblyomma americanum americanum A. americanum -76.5028111 37.0918568 16209

Amblyomma americanum americanum A. americanum -77.4089934 38.4220676 5117

Amblyomma americanum americanum A. americanum -76.9752541 38.5292892 5062

Amblyomma americanum americanum A. americanum -74.2029733 39.7915096 5469

Amblyomma americanum americanum A. americanum -75.5779839 39.0084496 5062

Amblyomma americanum americanum A. americanum -77.0432507 37.4504118 5577

Amblyomma americanum americanum A. americanum -77.915039 37.0420244 10500

Amblyomma americanum americanum A. americanum -77.915039 37.0420244 10500

Amblyomma americanum americanum A. americanum -77.915039 37.0420244 10500

Amblyomma americanum americanum A. americanum -77.915039 37.0420244 10500

Amblyomma americanum americanum A. americanum -77.915039 37.0420244 10500

Amblyomma americanum americanum A. americanum -77.915039 37.0420244 10500

Amblyomma americanum americanum A. americanum -77.915039 37.0420244 10500

Amblyomma americanum americanum A. americanum -77.915039 37.0420244 10500

Amblyomma americanum americanum A. americanum -77.915039 37.0420244 10500

Amblyomma americanum americanum A. americanum -77.915039 37.0420244 10500

Amblyomma americanum americanum A. americanum -77.915039 37.0420244 10500

Amblyomma americanum americanum A. americanum -77.915039 37.0420244 10500

Amblyomma americanum americanum A. americanum -77.915039 37.0420244 10500

Amblyomma americanum americanum A. americanum -77.915039 37.0420244 10500

Amblyomma americanum americanum A. americanum -77.915039 37.0420244 10500

Amblyomma americanum americanum A. americanum -77.915039 37.0420244 10500

Amblyomma americanum americanum A. americanum -77.915039 37.0420244 10500

Amblyomma americanum americanum A. americanum -77.915039 37.0420244 10500

Amblyomma americanum americanum A. americanum -77.915039 37.0420244 10500

Amblyomma americanum americanum A. americanum -76.7074547 37.2707005 5062

Amblyomma americanum americanum A. americanum -77.915039 37.0420244 10500

Amblyomma americanum americanum A. americanum -87.4988899 37.3280983 5062

Amblyomma americanum americanum A. americanum -76.5125753 37.2304516 5499

Amblyomma americanum americanum A. americanum -77.3255424 38.8009472 5062

Amblyomma americanum americanum A. americanum -76.0763283 38.7742844 5062

Amblyomma americanum americanum A. americanum -77.9966583 38.4731827 5062

Amblyomma americanum americanum A. americanum -77.1872063 38.7892799 5062

Amblyomma americanum americanum A. americanum -74.575985 39.6840096 5062

Amblyomma americanum americanum A. americanum -77.561718 37.3910943 28230

Amblyomma americanum americanum A. americanum -98.3903313 34.6086864 5063

Amblyomma americanum americanum A. americanum -79.256467 35.3251075 5202

Amblyomma americanum americanum A. americanum -76.5028111 37.0918568 16209

Amblyomma americanum americanum A. americanum -76.5115539 37.2387543 5217

Amblyomma americanum americanum A. americanum -96.5724539 39.183609 5125

Amblyomma americanum americanum A. americanum -76.2791252 39.4623318 5062

Amblyomma americanum americanum A. americanum -78.8881989 35.0527 6468

Amblyomma americanum americanum A. americanum -98.3903313 34.6086864 5063

Amblyomma americanum americanum A. americanum -75.5777054 38.9237232 5062

Amblyomma americanum americanum A. americanum -78.8881989 35.0527 6468

Amblyomma americanum americanum A. americanum -77.0722032 38.7804695 3686

Amblyomma americanum americanum A. americanum -76.3457756 37.1224925 5076

Amblyomma americanum americanum A. americanum -76.5125753 37.2304516 5499

Amblyomma americanum americanum A. americanum -78.8572921 36.0512598 25293

Amblyomma americanum americanum A. americanum -75.9413338 39.6001148 5062

Amblyomma americanum americanum A. americanum -85.7595492 38.2698995 5102

Amblyomma americanum americanum A. americanum -77.915039 37.0420244 10500

Amblyomma americanum americanum A. americanum -77.915039 37.0420244 10500

Amblyomma americanum americanum A. americanum -81.033777 34.0008472 5147

Amblyomma americanum americanum A. americanum -87.3594513 36.5297699 5063

Amblyomma americanum americanum A. americanum -78.4766808 38.0293045 5062

Amblyomma americanum americanum A. americanum -77.0085907 40.2142544 5062

Amblyomma americanum americanum A. americanum -74.1723671 40.2876129 5062

Amblyomma americanum americanum A. americanum -74.1723671 40.2876129 5062

Amblyomma americanum americanum A. americanum -79.8430866 37.2313504 39206

Amblyomma americanum americanum A. americanum -87.3594513 36.5297699 5063

Amblyomma americanum americanum A. americanum -87.4427948 36.6757663 5772

Amblyomma americanum americanum A. americanum -77.915039 37.0420244 10500

Amblyomma americanum americanum A. americanum -77.915039 37.0420244 10500

Amblyomma americanum americanum A. americanum -77.915039 37.0420244 10500

Amblyomma americanum americanum A. americanum -77.915039 37.0420244 10500

Amblyomma americanum americanum A. americanum -77.915039 37.0420244 10500

Amblyomma americanum americanum A. americanum -77.915039 37.0420244 10500

Amblyomma americanum americanum A. americanum -77.915039 37.0420244 10500

Amblyomma americanum americanum A. americanum -77.915039 37.0420244 10500

Amblyomma americanum americanum A. americanum -77.915039 37.0420244 10500

Amblyomma americanum americanum A. americanum -77.915039 37.0420244 10500

Amblyomma americanum americanum A. americanum -77.915039 37.0420244 10500

Amblyomma americanum americanum A. americanum -77.915039 37.0420244 10500

Amblyomma americanum americanum A. americanum -77.915039 37.0420244 10500

Amblyomma americanum americanum A. americanum -77.915039 37.0420244 10500

Amblyomma americanum americanum A. americanum -79.9805035 40.4313764 10457

Amblyomma americanum americanum A. americanum -77.915039 37.0420244 10500

Amblyomma americanum americanum A. americanum -77.915039 37.0420244 10500

Amblyomma americanum americanum A. americanum -77.915039 37.0420244 10500

Amblyomma americanum americanum A. americanum -77.915039 37.0420244 10500

Amblyomma americanum americanum A. americanum -77.915039 37.0420244 10500

Amblyomma americanum americanum A. americanum -77.915039 37.0420244 10500

Amblyomma americanum americanum A. americanum -77.915039 37.0420244 10500

Amblyomma americanum americanum A. americanum -77.915039 37.0420244 10500

Amblyomma americanum americanum A. americanum -77.915039 37.0420244 10500

Amblyomma americanum americanum A. americanum -77.915039 37.0420244 10500

Amblyomma americanum americanum A. americanum -77.915039 37.0420244 10500

Amblyomma americanum americanum A. americanum -77.915039 37.0420244 10500

Amblyomma americanum americanum A. americanum -76.3884399 37.0070712 1610

Amblyomma americanum americanum A. americanum -77.915039 37.0420244 10500

Amblyomma americanum americanum A. americanum -77.915039 37.0420244 10500

Amblyomma americanum americanum A. americanum -77.915039 37.0420244 10500

Amblyomma americanum americanum A. americanum -78.8881989 35.0527 6468

Amblyomma americanum americanum A. americanum -77.915039 37.0420244 10500

Amblyomma americanum americanum A. americanum -77.915039 37.0420244 10500

Amblyomma americanum americanum A. americanum -77.915039 37.0420244 10500

Amblyomma americanum americanum A. americanum -77.915039 37.0420244 10500

Amblyomma americanum americanum A. americanum -85.9699593 37.7495079 5062

Amblyomma americanum americanum A. americanum -77.2880363 37.2204266 5062

Amblyomma americanum americanum A. americanum -75.5779839 39.0084496 5062

Amblyomma americanum americanum A. americanum -77.1872063 38.7892799 5062

Amblyomma americanum americanum A. americanum -75.7707634 38.0392895 5062

Amblyomma americanum americanum A. americanum -76.6896666 39.9731559 47417

Amblyomma americanum americanum A. americanum -78.8572921 36.0512598 25293

Amblyomma americanum americanum A. americanum -76.4956594 38.9784451 5315

Amblyomma americanum americanum A. americanum -77.0722032 38.7804695 3686

Amblyomma americanum americanum A. americanum -77.0722032 38.7804695 3686

Amblyomma americanum americanum A. americanum -77.915039 37.0420244 10500

Amblyomma americanum americanum A. americanum -77.915039 37.0420244 10500

Amblyomma americanum americanum A. americanum -77.915039 37.0420244 10500

Amblyomma americanum americanum A. americanum -77.915039 37.0420244 10500

Amblyomma americanum americanum A. americanum -77.915039 37.0420244 10500

Amblyomma americanum americanum A. americanum -77.915039 37.0420244 10500

Amblyomma americanum americanum A. americanum -77.915039 37.0420244 10500

Amblyomma americanum americanum A. americanum -77.915039 37.0420244 10500

Amblyomma americanum americanum A. americanum -77.915039 37.0420244 10500

Amblyomma americanum americanum A. americanum -77.915039 37.0420244 10500

Amblyomma americanum americanum A. americanum -77.915039 37.0420244 10500

Amblyomma americanum americanum A. americanum -77.915039 37.0420244 10500

Amblyomma americanum americanum A. americanum -77.915039 37.0420244 10500

Amblyomma americanum americanum A. americanum -77.915039 37.0420244 10500

Amblyomma americanum americanum A. americanum -77.915039 37.0420244 10500

Amblyomma americanum americanum A. americanum -77.915039 37.0420244 10500

Amblyomma americanum americanum A. americanum -77.915039 37.0420244 10500

Amblyomma americanum americanum A. americanum -77.915039 37.0420244 10500

Amblyomma americanum americanum A. americanum -77.915039 37.0420244 10500

Amblyomma americanum americanum A. americanum -77.915039 37.0420244 10500

Amblyomma americanum americanum A. americanum -77.915039 37.0420244 10500

Amblyomma americanum americanum A. americanum -77.915039 37.0420244 10500

Amblyomma americanum americanum A. americanum -77.915039 37.0420244 10500

Amblyomma americanum americanum A. americanum -77.915039 37.0420244 10500

Amblyomma americanum americanum A. americanum -77.915039 37.0420244 10500

Amblyomma americanum americanum A. americanum -77.915039 37.0420244 10500

Amblyomma americanum americanum A. americanum -77.915039 37.0420244 10500

Amblyomma americanum americanum A. americanum -77.915039 37.0420244 10500

Amblyomma americanum americanum A. americanum -77.915039 37.0420244 10500

Amblyomma americanum americanum A. americanum -77.915039 37.0420244 10500

Amblyomma americanum americanum A. americanum -77.915039 37.0420244 10500

Amblyomma americanum americanum A. americanum -77.915039 37.0420244 10500

Amblyomma americanum americanum A. americanum -77.915039 37.0420244 10500

Amblyomma americanum americanum A. americanum -77.915039 37.0420244 10500

Amblyomma americanum americanum A. americanum -77.915039 37.0420244 10500

Amblyomma americanum americanum A. americanum -77.915039 37.0420244 10500

Amblyomma americanum americanum A. americanum -77.915039 37.0420244 10500

Amblyomma americanum americanum A. americanum -77.915039 37.0420244 10500

Amblyomma americanum americanum A. americanum -77.915039 37.0420244 10500

Amblyomma americanum americanum A. americanum -77.915039 37.0420244 10500

Amblyomma americanum americanum A. americanum -77.915039 37.0420244 10500

Amblyomma americanum americanum A. americanum -77.915039 37.0420244 10500

Amblyomma americanum americanum A. americanum -77.915039 37.0420244 10500

Amblyomma americanum americanum A. americanum -77.915039 37.0420244 10500

Amblyomma americanum americanum A. americanum -77.915039 37.0420244 10500

Amblyomma americanum americanum A. americanum -77.915039 37.0420244 10500

Amblyomma americanum americanum A. americanum -77.915039 37.0420244 10500

Amblyomma americanum americanum A. americanum -77.915039 37.0420244 10500

Amblyomma americanum americanum A. americanum -77.915039 37.0420244 10500

Amblyomma americanum americanum A. americanum -77.915039 37.0420244 10500

Amblyomma americanum americanum A. americanum -77.915039 37.0420244 10500

Amblyomma americanum americanum A. americanum -77.915039 37.0420244 10500

Amblyomma americanum americanum A. americanum -77.915039 37.0420244 10500

Amblyomma americanum americanum A. americanum -77.915039 37.0420244 10500

Amblyomma americanum americanum A. americanum -77.915039 37.0420244 10500

Amblyomma americanum americanum A. americanum -77.915039 37.0420244 10500

Amblyomma americanum americanum A. americanum -77.915039 37.0420244 10500

Amblyomma americanum americanum A. americanum -77.915039 37.0420244 10500

Amblyomma americanum americanum A. americanum -77.915039 37.0420244 10500

Amblyomma americanum americanum A. americanum -77.915039 37.0420244 10500

Amblyomma americanum americanum A. americanum -77.915039 37.0420244 10500

Amblyomma americanum americanum A. americanum -76.3877411 39.5851097 5062

Amblyomma americanum americanum A. americanum -74.7104265 40.1422345 5087

Amblyomma americanum americanum A. americanum -77.1872063 38.7892799 5062

Amblyomma americanum americanum A. americanum -76.6077232 36.0598271 5250

Amblyomma americanum americanum A. americanum -77.4416542 37.3568172 5062

Amblyomma americanum americanum A. americanum -76.5028111 37.0918568 16209

Amblyomma americanum americanum A. americanum -76.5028111 37.0918568 16209

Amblyomma americanum americanum A. americanum -77.915039 37.0420244 10500

Amblyomma americanum americanum A. americanum -77.915039 37.0420244 10500

Amblyomma americanum americanum A. americanum -77.915039 37.0420244 10500

Amblyomma americanum americanum A. americanum -77.915039 37.0420244 10500

Amblyomma americanum americanum A. americanum -77.915039 37.0420244 10500

Amblyomma americanum americanum A. americanum -74.1370811 40.0592823 5062

Amblyomma americanum americanum A. americanum -76.2959883 39.4187183 5189

Amblyomma americanum americanum A. americanum -87.3594513 36.5297699 5063

Amblyomma americanum americanum A. americanum -77.1014742 38.9816529 5125

Amblyomma americanum americanum A. americanum -88.1436316 32.8355608 4756

Amblyomma americanum americanum A. americanum -88.1436316 32.8355608 4756

Amblyomma americanum americanum A. americanum -76.8394241 39.2403851 5062

Amblyomma americanum americanum A. americanum -77.915039 37.0420244 10500

Amblyomma americanum americanum A. americanum -77.915039 37.0420244 10500

Amblyomma americanum americanum A. americanum -77.915039 37.0420244 10500

Amblyomma americanum americanum A. americanum -77.915039 37.0420244 10500

Amblyomma americanum americanum A. americanum -74.2916107 39.8454492 11000

Amblyomma americanum americanum A. americanum -74.2916107 39.8454492 11000

Amblyomma americanum americanum A. americanum -78.8881989 35.0527 6468

Amblyomma americanum americanum A. americanum -77.8957481 34.5104348 35768

Amblyomma americanum americanum A. americanum -78.6386261 35.7720528 9703

Amblyomma americanum americanum A. americanum -88.8271134 30.4167174 5474

Amblyomma americanum americanum A. americanum -76.3456386 37.0353572 5490

Amblyomma americanum americanum A. americanum -76.3456386 37.0353572 5490

Amblyomma americanum americanum A. americanum -81.033777 34.0008472 5147

Amblyomma americanum americanum A. americanum -77.915039 37.0420244 10500

Amblyomma americanum americanum A. americanum -77.915039 37.0420244 10500

Amblyomma americanum americanum A. americanum -77.915039 37.0420244 10500

Amblyomma americanum americanum A. americanum -77.915039 37.0420244 10500

Amblyomma americanum americanum A. americanum -77.915039 37.0420244 10500

Amblyomma americanum americanum A. americanum -77.915039 37.0420244 10500

Amblyomma americanum americanum A. americanum -77.915039 37.0420244 10500

Amblyomma americanum americanum A. americanum -77.915039 37.0420244 10500

Amblyomma americanum americanum A. americanum -77.915039 37.0420244 10500

Amblyomma americanum americanum A. americanum -77.915039 37.0420244 10500

Amblyomma americanum americanum A. americanum -77.915039 37.0420244 10500

Amblyomma americanum americanum A. americanum -77.915039 37.0420244 10500

Amblyomma americanum americanum A. americanum -77.915039 37.0420244 10500

Amblyomma americanum americanum A. americanum -77.915039 37.0420244 10500

Amblyomma americanum americanum A. americanum -77.915039 37.0420244 10500

Amblyomma americanum americanum A. americanum -77.915039 37.0420244 10500

Amblyomma americanum americanum A. americanum -77.915039 37.0420244 10500

Amblyomma americanum americanum A. americanum -77.915039 37.0420244 10500

Amblyomma americanum americanum A. americanum -77.915039 37.0420244 10500

Amblyomma americanum americanum A. americanum -77.915039 37.0420244 10500

Amblyomma americanum americanum A. americanum -77.915039 37.0420244 10500

Amblyomma americanum americanum A. americanum -77.915039 37.0420244 10500

Amblyomma americanum americanum A. americanum -77.915039 37.0420244 10500

Amblyomma americanum americanum A. americanum -77.915039 37.0420244 10500

Amblyomma americanum americanum A. americanum -77.915039 37.0420244 10500

Amblyomma americanum americanum A. americanum -77.915039 37.0420244 10500

Amblyomma americanum americanum A. americanum -76.7074547 37.2707005 5062

Amblyomma americanum americanum A. americanum -76.6026324 37.2009057 3564

Amblyomma americanum americanum A. americanum -76.4176133 36.9937713 4464

Amblyomma americanum americanum A. americanum -76.9013329 34.8833651 5445

Amblyomma americanum americanum A. americanum -78.5813942 35.9418163 3054

Amblyomma americanum americanum A. americanum -76.0627251 39.6875458 5767

Amblyomma americanum americanum A. americanum -77.915039 37.0420244 10500

Amblyomma americanum americanum A. americanum -77.915039 37.0420244 10500

Amblyomma americanum americanum A. americanum -77.915039 37.0420244 10500

Amblyomma americanum americanum A. americanum -77.915039 37.0420244 10500

Amblyomma americanum americanum A. americanum -77.915039 37.0420244 10500

Amblyomma americanum americanum A. americanum -77.915039 37.0420244 10500

Amblyomma americanum americanum A. americanum -77.915039 37.0420244 10500

Amblyomma americanum americanum A. americanum -77.915039 37.0420244 10500

Amblyomma americanum americanum A. americanum -77.915039 37.0420244 10500

Amblyomma americanum americanum A. americanum -77.915039 37.0420244 10500

Amblyomma americanum americanum A. americanum -77.915039 37.0420244 10500

Amblyomma americanum americanum A. americanum -77.915039 37.0420244 10500

Amblyomma americanum americanum A. americanum -77.460537 38.3031826 5062

Amblyomma americanum americanum A. americanum -74.6167373 40.0086834 6500

Amblyomma americanum americanum A. americanum -76.5125753 37.2304516 5499

Amblyomma americanum americanum A. americanum -77.915039 37.0420244 10500

Amblyomma americanum americanum A. americanum -77.915039 37.0420244 10500

Amblyomma americanum americanum A. americanum -77.915039 37.0420244 10500

Amblyomma americanum americanum A. americanum -77.915039 37.0420244 10500

Amblyomma americanum americanum A. americanum -77.915039 37.0420244 10500

Amblyomma americanum americanum A. americanum -77.915039 37.0420244 10500

Amblyomma americanum americanum A. americanum -84.5825182 33.3985018 5636

Amblyomma americanum americanum A. americanum -77.2750854 38.1097082 13000

Amblyomma americanum americanum A. americanum -77.2750854 38.1097082 13000

Amblyomma americanum americanum A. americanum -77.915039 37.0420244 10500

Amblyomma americanum americanum A. americanum -77.915039 37.0420244 10500

Amblyomma americanum americanum A. americanum -77.915039 37.0420244 10500

Amblyomma americanum americanum A. americanum -77.915039 37.0420244 10500

Amblyomma americanum americanum A. americanum -77.915039 37.0420244 10500

Amblyomma americanum americanum A. americanum -77.915039 37.0420244 10500

Amblyomma americanum americanum A. americanum -77.915039 37.0420244 10500

Amblyomma americanum americanum A. americanum -77.915039 37.0420244 10500

Amblyomma americanum americanum A. americanum -77.915039 37.0420244 10500

Amblyomma americanum americanum A. americanum -77.915039 37.0420244 10500

Amblyomma americanum americanum A. americanum -77.915039 37.0420244 10500

Amblyomma americanum americanum A. americanum -77.915039 37.0420244 10500

Amblyomma americanum americanum A. americanum -77.915039 37.0420244 10500

Amblyomma americanum americanum A. americanum -77.915039 37.0420244 10500

Amblyomma americanum americanum A. americanum -77.915039 37.0420244 10500

Amblyomma americanum americanum A. americanum -77.915039 37.0420244 10500

Amblyomma americanum americanum A. americanum -77.915039 37.0420244 10500

Amblyomma americanum americanum A. americanum -77.915039 37.0420244 10500

Amblyomma americanum americanum A. americanum -77.915039 37.0420244 10500

Amblyomma americanum americanum A. americanum -77.915039 37.0420244 10500

Amblyomma americanum americanum A. americanum -77.915039 37.0420244 10500

Amblyomma americanum americanum A. americanum -77.915039 37.0420244 10500

Amblyomma americanum americanum A. americanum -77.915039 37.0420244 10500

Amblyomma americanum americanum A. americanum -77.915039 37.0420244 10500

Amblyomma americanum americanum A. americanum -77.915039 37.0420244 10500

Amblyomma americanum americanum A. americanum -77.915039 37.0420244 10500

Amblyomma americanum americanum A. americanum -77.915039 37.0420244 10500

Amblyomma americanum americanum A. americanum -77.915039 37.0420244 10500

Amblyomma americanum americanum A. americanum -77.915039 37.0420244 10500

Amblyomma americanum americanum A. americanum -77.915039 37.0420244 10500

Amblyomma americanum americanum A. americanum -77.915039 37.0420244 10500

Amblyomma americanum americanum A. americanum -74.1514316 40.2555169 6602

Amblyomma americanum americanum A. americanum -74.6167373 40.0086834 6500

Amblyomma americanum americanum A. americanum -74.2737541 40.2601109 5062

Amblyomma americanum americanum A. americanum -77.6633336 38.1847954 26993

Amblyomma americanum americanum A. americanum -77.5891571 38.2009659 5062

Amblyomma americanum americanum A. americanum -77.0722032 38.7804695 3686

Amblyomma americanum americanum A. americanum -84.3538094 33.5215015 5063

Amblyomma americanum americanum A. americanum -77.2750854 38.1097082 13000

Amblyomma americanum americanum A. americanum -77.2750854 38.1097082 13000

Amblyomma americanum americanum A. americanum -77.2750854 38.1097082 13000

Amblyomma americanum americanum A. americanum -77.2750854 38.1097082 13000

Amblyomma americanum americanum A. americanum -77.2750854 38.1097082 13000

Amblyomma americanum americanum A. americanum -77.2750854 38.1097082 13000

Amblyomma americanum americanum A. americanum -77.2750854 38.1097082 13000

Amblyomma americanum americanum A. americanum -77.2750854 38.1097082 13000

Amblyomma americanum americanum A. americanum -74.6167373 40.0086834 6500

Amblyomma americanum americanum A. americanum -75.5243645 39.158165 5062

Amblyomma americanum americanum A. americanum -75.5243645 39.158165 5062

Amblyomma americanum americanum A. americanum -92.8335228 38.1991978 5062

Amblyomma americanum americanum A. americanum -85.6985741 37.5120068 5062

Amblyomma americanum americanum A. americanum -76.6982994 39.1370525 5062

Amblyomma americanum americanum A. americanum -77.915039 37.0420244 10500

Amblyomma americanum americanum A. americanum -77.915039 37.0420244 10500

Amblyomma americanum americanum A. americanum -77.915039 37.0420244 10500

Amblyomma americanum americanum A. americanum -77.915039 37.0420244 10500

Amblyomma americanum americanum A. americanum -77.915039 37.0420244 10500

Amblyomma americanum americanum A. americanum -77.915039 37.0420244 10500

Amblyomma americanum americanum A. americanum -77.915039 37.0420244 10500

Amblyomma americanum americanum A. americanum -77.915039 37.0420244 10500

Amblyomma americanum americanum A. americanum -77.915039 37.0420244 10500

Amblyomma americanum americanum A. americanum -77.915039 37.0420244 10500

Amblyomma americanum americanum A. americanum -77.915039 37.0420244 10500

Amblyomma americanum americanum A. americanum -77.915039 37.0420244 10500

Amblyomma americanum americanum A. americanum -77.915039 37.0420244 10500

Amblyomma americanum americanum A. americanum -77.915039 37.0420244 10500

Amblyomma americanum americanum A. americanum -77.915039 37.0420244 10500

Amblyomma americanum americanum A. americanum -77.2064016 39.1409358 27531

Amblyomma americanum americanum A. americanum -77.9836409 33.8675254 7000

Amblyomma americanum americanum A. americanum -77.2671266 38.6540242 4893

Amblyomma americanum americanum A. americanum -77.0722032 38.7804695 3686

Amblyomma americanum americanum A. americanum -77.4888616 38.7683483 7325

Amblyomma americanum americanum A. americanum -76.3884399 37.0070712 1610

Amblyomma americanum americanum A. americanum -76.5028111 37.0918568 16209

Amblyomma americanum americanum A. americanum -76.5028111 37.0918568 16209

Amblyomma americanum americanum A. americanum -76.5028111 37.0918568 16209

Amblyomma americanum americanum A. americanum -77.2750854 38.1097082 13000

Amblyomma americanum americanum A. americanum -76.5028111 37.0918568 16209

Amblyomma americanum americanum A. americanum -76.4538383 38.2657419 5170

Amblyomma americanum americanum A. americanum -77.2750854 38.1097082 13000

Amblyomma americanum americanum A. americanum -77.2750854 38.1097082 13000

Amblyomma americanum americanum A. americanum -77.2750854 38.1097082 13000

Amblyomma americanum americanum A. americanum -77.2750854 38.1097082 13000

Amblyomma americanum americanum A. americanum -77.2750854 38.1097082 13000

Amblyomma americanum americanum A. americanum -77.2750854 38.1097082 13000

Amblyomma americanum americanum A. americanum -77.2750854 38.1097082 13000

Amblyomma americanum americanum A. americanum -77.2750854 38.1097082 13000

Amblyomma americanum americanum A. americanum -77.2750854 38.1097082 13000

Amblyomma americanum americanum A. americanum -77.2750854 38.1097082 13000

Amblyomma americanum americanum A. americanum -77.2750854 38.1097082 13000

Amblyomma americanum americanum A. americanum -87.4886208 36.8656044 5063

Amblyomma americanum americanum A. americanum -78.6154481 34.2141988 44670

Amblyomma americanum americanum A. americanum -79.256467 35.3251075 5202

Amblyomma americanum americanum A. americanum -79.0135964 35.1730495 3038

Amblyomma americanum americanum A. americanum -77.915039 37.0420244 10500

Amblyomma americanum americanum A. americanum -77.915039 37.0420244 10500

Amblyomma americanum americanum A. americanum -77.915039 37.0420244 10500

Amblyomma americanum americanum A. americanum -77.915039 37.0420244 10500

Amblyomma americanum americanum A. americanum -77.915039 37.0420244 10500

Amblyomma americanum americanum A. americanum -77.915039 37.0420244 10500

Amblyomma americanum americanum A. americanum -77.915039 37.0420244 10500

Amblyomma americanum americanum A. americanum -77.915039 37.0420244 10500

Amblyomma americanum americanum A. americanum -77.915039 37.0420244 10500

Amblyomma americanum americanum A. americanum -77.915039 37.0420244 10500

Amblyomma americanum americanum A. americanum -77.915039 37.0420244 10500

Amblyomma americanum americanum A. americanum -77.915039 37.0420244 10500

Amblyomma americanum americanum A. americanum -77.915039 37.0420244 10500

Amblyomma americanum americanum A. americanum -77.915039 37.0420244 10500

Amblyomma americanum americanum A. americanum -77.915039 37.0420244 10500

Amblyomma americanum americanum A. americanum -77.915039 37.0420244 10500

Amblyomma americanum americanum A. americanum -77.915039 37.0420244 10500

Amblyomma americanum americanum A. americanum -77.915039 37.0420244 10500

Amblyomma americanum americanum A. americanum -77.915039 37.0420244 10500

Amblyomma americanum americanum A. americanum -77.915039 37.0420244 10500

Amblyomma americanum americanum A. americanum -77.915039 37.0420244 10500

Amblyomma americanum americanum A. americanum -77.915039 37.0420244 10500

Amblyomma americanum americanum A. americanum -77.915039 37.0420244 10500

Amblyomma americanum americanum A. americanum -77.915039 37.0420244 10500

Amblyomma americanum americanum A. americanum -77.915039 37.0420244 10500

Amblyomma americanum americanum A. americanum -77.915039 37.0420244 10500

Amblyomma americanum americanum A. americanum -77.915039 37.0420244 10500

Amblyomma americanum americanum A. americanum -77.915039 37.0420244 10500

Amblyomma americanum americanum A. americanum -77.915039 37.0420244 10500

Amblyomma americanum americanum A. americanum -77.915039 37.0420244 10500

Amblyomma americanum americanum A. americanum -77.915039 37.0420244 10500

Amblyomma americanum americanum A. americanum -77.915039 37.0420244 10500

Amblyomma americanum americanum A. americanum -77.915039 37.0420244 10500

Amblyomma americanum americanum A. americanum -77.915039 37.0420244 10500

Amblyomma americanum americanum A. americanum -77.915039 37.0420244 10500

Amblyomma americanum americanum A. americanum -77.915039 37.0420244 10500

Amblyomma americanum americanum A. americanum -77.915039 37.0420244 10500

Amblyomma americanum americanum A. americanum -77.915039 37.0420244 10500

Amblyomma americanum americanum A. americanum -77.915039 37.0420244 10500

Amblyomma americanum americanum A. americanum -77.915039 37.0420244 10500

Amblyomma americanum americanum A. americanum -77.915039 37.0420244 10500

Amblyomma americanum americanum A. americanum -77.915039 37.0420244 10500

Amblyomma americanum americanum A. americanum -77.915039 37.0420244 10500

Amblyomma americanum americanum A. americanum -77.915039 37.0420244 10500

Amblyomma americanum americanum A. americanum -77.915039 37.0420244 10500

Amblyomma americanum americanum A. americanum -77.915039 37.0420244 10500

Amblyomma americanum americanum A. americanum -77.915039 37.0420244 10500

Amblyomma americanum americanum A. americanum -77.915039 37.0420244 10500

Amblyomma americanum americanum A. americanum -77.915039 37.0420244 10500

Amblyomma americanum americanum A. americanum -77.915039 37.0420244 10500

Amblyomma americanum americanum A. americanum -77.915039 37.0420244 10500

Amblyomma americanum americanum A. americanum -77.915039 37.0420244 10500

Amblyomma americanum americanum A. americanum -77.915039 37.0420244 10500

Amblyomma americanum americanum A. americanum -77.915039 37.0420244 10500

Amblyomma americanum americanum A. americanum -77.915039 37.0420244 10500

Amblyomma americanum americanum A. americanum -77.915039 37.0420244 10500

Amblyomma americanum americanum A. americanum -77.915039 37.0420244 10500

Amblyomma americanum americanum A. americanum -77.915039 37.0420244 10500

Amblyomma americanum americanum A. americanum -77.915039 37.0420244 10500

Amblyomma americanum americanum A. americanum -77.915039 37.0420244 10500

Amblyomma americanum americanum A. americanum -77.915039 37.0420244 10500

Amblyomma americanum americanum A. americanum -76.4472055 37.1098869 2000

Amblyomma americanum americanum A. americanum -77.915039 37.0420244 10500

Amblyomma americanum americanum A. americanum -77.915039 37.0420244 10500

Amblyomma americanum americanum A. americanum -77.915039 37.0420244 10500

Amblyomma americanum americanum A. americanum -77.915039 37.0420244 10500

Amblyomma americanum americanum A. americanum -77.915039 37.0420244 10500

Amblyomma americanum americanum A. americanum -77.915039 37.0420244 10500

Amblyomma americanum americanum A. americanum -77.915039 37.0420244 10500

Amblyomma americanum americanum A. americanum -77.915039 37.0420244 10500

Amblyomma americanum americanum A. americanum -77.915039 37.0420244 10500

Amblyomma americanum americanum A. americanum -77.915039 37.0420244 10500

Amblyomma americanum americanum A. americanum -76.5028111 37.0918568 16209

Amblyomma americanum americanum A. americanum -77.915039 37.0420244 10500

Amblyomma americanum americanum A. americanum -77.915039 37.0420244 10500

Amblyomma americanum americanum A. americanum -77.915039 37.0420244 10500

Amblyomma americanum americanum A. americanum -77.915039 37.0420244 10500

Amblyomma americanum americanum A. americanum -77.915039 37.0420244 10500

Amblyomma americanum americanum A. americanum -77.915039 37.0420244 10500

Amblyomma americanum americanum A. americanum -77.915039 37.0420244 10500

Amblyomma americanum americanum A. americanum -77.915039 37.0420244 10500

Amblyomma americanum americanum A. americanum -77.915039 37.0420244 10500

Amblyomma americanum americanum A. americanum -74.355812 39.9481742 5000

Amblyomma americanum americanum A. americanum -84.170278 33.635278 2000

Amblyomma americanum americanum A. americanum -80.8431282 35.2270889 5063

Amblyomma americanum americanum A. americanum -92.1192411 34.8662033 5640

Amblyomma americanum americanum A. americanum -74.0509758 40.2962208 5062

Amblyomma americanum americanum A. americanum -78.8881989 35.0527 6468

Amblyomma americanum americanum A. americanum -77.7457954 39.4577472 5183

Amblyomma americanum americanum A. americanum -74.0843672 41.5015441 1058

Amblyomma americanum americanum A. americanum -77.6491931 37.0727008 24752

Amblyomma americanum americanum A. americanum -78.0755501 38.9089985 5062

Amblyomma americanum americanum A. americanum -77.915039 37.0420244 10500

Amblyomma americanum americanum A. americanum -77.915039 37.0420244 10500

Amblyomma americanum americanum A. americanum -76.5028111 37.0918568 16209

Amblyomma americanum americanum A. americanum -77.915039 37.0420244 10500

Amblyomma americanum americanum A. americanum -77.915039 37.0420244 10500

Amblyomma americanum americanum A. americanum -77.915039 37.0420244 10500

Amblyomma americanum americanum A. americanum -77.915039 37.0420244 10500

Amblyomma americanum americanum A. americanum -77.915039 37.0420244 10500

Amblyomma americanum americanum A. americanum -77.915039 37.0420244 10500

Amblyomma americanum americanum A. americanum -77.915039 37.0420244 10500

Amblyomma americanum americanum A. americanum -77.915039 37.0420244 10500

Amblyomma americanum americanum A. americanum -77.915039 37.0420244 10500

Amblyomma americanum americanum A. americanum -77.915039 37.0420244 10500

Amblyomma americanum americanum A. americanum -77.915039 37.0420244 10500

Amblyomma americanum americanum A. americanum -77.915039 37.0420244 10500

Amblyomma americanum americanum A. americanum -77.915039 37.0420244 10500

Amblyomma americanum americanum A. americanum -77.915039 37.0420244 10500

Amblyomma americanum americanum A. americanum -77.915039 37.0420244 10500

Amblyomma americanum americanum A. americanum -77.915039 37.0420244 10500

Amblyomma americanum americanum A. americanum -77.915039 37.0420244 10500

Amblyomma americanum americanum A. americanum -77.915039 37.0420244 10500

Amblyomma americanum americanum A. americanum -77.915039 37.0420244 10500

Amblyomma americanum americanum A. americanum -77.915039 37.0420244 10500

Amblyomma americanum americanum A. americanum -77.915039 37.0420244 10500

Amblyomma americanum americanum A. americanum -77.915039 37.0420244 10500

Amblyomma americanum americanum A. americanum -77.915039 37.0420244 10500

Amblyomma americanum americanum A. americanum -81.3951569 30.22738 5810

Amblyomma americanum americanum A. americanum -95.7697105 34.9334316 5063

Amblyomma americanum americanum A. americanum -76.3456386 37.0353572 5490

Amblyomma americanum americanum A. americanum -87.8414116 36.320612 5063

Amblyomma americanum americanum A. americanum -77.2277641 38.7042868 5063

Amblyomma americanum americanum A. americanum -76.7074547 37.2707005 5062

Amblyomma americanum americanum A. americanum -77.1872063 38.7892799 5062

Amblyomma americanum americanum A. americanum -88.055519 36.780891 35000

Amblyomma americanum americanum A. americanum -76.5028111 37.0918568 16209

Amblyomma americanum americanum A. americanum -76.6310692 36.9823704 5063

Amblyomma americanum americanum A. americanum -78.0691605 34.1943398 5063

Amblyomma americanum americanum A. americanum -76.5028111 37.0918568 16209

Amblyomma americanum americanum A. americanum -87.3594513 36.5297699 5063

Amblyomma americanum americanum A. americanum -87.5105705 36.5442162 5063

Amblyomma americanum americanum A. americanum -78.2538872 38.955389 5062

Amblyomma americanum americanum A. americanum -92.2007294 37.8286514 5062

Amblyomma americanum americanum A. americanum -70.5439186 41.657053 5062

Amblyomma americanum americanum A. americanum -85.859127 37.6939526 5062

Amblyomma americanum americanum A. americanum -92.2884831 34.768982 4655

Amblyomma americanum americanum A. americanum -78.2386208 39.9992504 5062

Amblyomma americanum americanum A. americanum -75.5243645 39.158165 5062

Amblyomma americanum americanum A. americanum -85.8552132 31.3151712 5064

Amblyomma americanum americanum A. americanum -76.7074547 37.2707005 5062

Amblyomma americanum americanum A. americanum -76.5028111 37.0918568 16209

Amblyomma americanum americanum A. americanum -79.1803017 35.4798756 5063

Amblyomma americanum americanum A. americanum -77.3050865 38.5344991 4853

Amblyomma americanum americanum A. americanum -77.3050865 38.5344991 4853

Amblyomma americanum americanum A. americanum -80.6701655 32.4324804 5168

Amblyomma americanum americanum A. americanum -95.8991585 34.7219284 5195

Amblyomma americanum americanum A. americanum -77.3280373 38.5676212 5062

Amblyomma americanum americanum A. americanum -81.326462 30.022505 4000

Amblyomma americanum americanum A. americanum -77.4291414 37.2561321 4801

Amblyomma americanum americanum A. americanum -79.4298901 35.2779294 39940

Amblyomma americanum americanum A. americanum -81.6556511 30.3321848 5064

Amblyomma americanum americanum A. americanum -77.2671266 38.6540242 4893

Amblyomma americanum americanum A. americanum -77.044117 35.1084919 5063

Amblyomma americanum americanum A. americanum -76.3456386 37.0353572 5490

Amblyomma americanum americanum A. americanum -92.2243156 34.8150921 5063

Amblyomma americanum americanum A. americanum -78.3919411 37.3020954 5062

Amblyomma americanum americanum A. americanum -76.5028111 37.0918568 16209

Amblyomma americanum americanum A. americanum -76.5028111 37.0918568 16209

Amblyomma americanum americanum A. americanum -77.915039 37.0420244 10500

Amblyomma americanum americanum A. americanum -74.6329348 39.9723387 2696

Amblyomma americanum americanum A. americanum -77.9319026 34.2257729 7247

Amblyomma americanum americanum A. americanum -79.1803017 35.4798756 5063

Amblyomma americanum americanum A. americanum -76.5409355 38.5419254 26810

Amblyomma americanum americanum A. americanum -76.5028111 37.0918568 16209

Amblyomma americanum americanum A. americanum -76.5028111 37.0918568 16209

Amblyomma americanum americanum A. americanum -76.5028111 37.0918568 16209

Amblyomma americanum americanum A. americanum -77.915039 37.0420244 10500

Amblyomma americanum americanum A. americanum -77.915039 37.0420244 10500

Amblyomma americanum americanum A. americanum -81.033777 34.0008472 5147

Amblyomma americanum americanum A. americanum -75.4760284 39.0712223 5062

Amblyomma americanum americanum A. americanum -76.5125753 37.2304516 5499

Amblyomma americanum americanum A. americanum -76.8041267 37.3798676 5062

Amblyomma americanum americanum A. americanum -76.5028111 37.0918568 16209

Amblyomma americanum americanum A. americanum -77.0722032 38.7804695 3686

Amblyomma americanum americanum A. americanum -77.2671266 38.6540242 4893

Amblyomma americanum americanum A. americanum -84.4549294 33.4487247 5063

Amblyomma americanum americanum A. americanum -87.3594513 36.5297699 5063

Amblyomma americanum americanum A. americanum -74.3784866 39.9545612 5062

Amblyomma americanum americanum A. americanum -74.0344713 41.476757 5661

Amblyomma americanum americanum A. americanum -76.5028111 37.0918568 16209

Amblyomma americanum americanum A. americanum -74.2887916 40.2735349 32730

Amblyomma americanum americanum A. americanum -77.3992552 34.5527287 5227

Amblyomma americanum americanum A. americanum -77.1872063 38.7892799 5062

Amblyomma americanum americanum A. americanum -77.1872063 38.7892799 5062

Amblyomma americanum americanum A. americanum -77.2716522 38.7934494 5062

Amblyomma americanum americanum A. americanum -77.28825 38.8387894 26390

Amblyomma americanum americanum A. americanum -79.1803017 35.4798756 5063

Amblyomma americanum americanum A. americanum -86.2855343 37.8778473 5228

Amblyomma americanum americanum A. americanum -77.915039 37.0420244 10500

Amblyomma americanum americanum A. americanum -74.6829338 39.9720612 5062

Amblyomma americanum americanum A. americanum -77.915039 37.0420244 10500

Amblyomma americanum americanum A. americanum -77.2750854 38.1097082 13000

Amblyomma americanum americanum A. americanum -77.915039 37.0420244 10500

Amblyomma americanum americanum A. americanum -77.915039 37.0420244 10500

Amblyomma americanum americanum A. americanum -77.915039 37.0420244 10500

Amblyomma americanum americanum A. americanum -76.5028111 37.0918568 16209

Amblyomma americanum americanum A. americanum -84.1070945 33.7123318 5227

Amblyomma americanum americanum A. americanum -81.0348167 34.0007114 5063

Amblyomma americanum americanum A. americanum -77.3003651 38.6802599 5496

Amblyomma americanum americanum A. americanum -77.2671266 38.6540242 4893

Amblyomma americanum americanum A. americanum -77.2671266 38.6540242 4893

Amblyomma americanum americanum A. americanum -87.8352928 36.8650513 5063

Amblyomma americanum americanum A. americanum -94.9287936 39.3111076 5480

Amblyomma americanum americanum A. americanum -76.5125753 37.2304516 5499

Amblyomma americanum americanum A. americanum -77.3127632 39.1837196 5062

Amblyomma americanum americanum A. americanum -75.9413338 39.6001148 5062

Amblyomma americanum americanum A. americanum -77.3063698 38.8462238 5062

Amblyomma americanum americanum A. americanum -77.3063698 38.8462238 5062

Amblyomma americanum americanum A. americanum -76.5028111 37.0918568 16209

Amblyomma americanum americanum A. americanum -76.5028111 37.0918568 16209

Amblyomma americanum americanum A. americanum -87.1788826 37.2011547 5062

Amblyomma americanum americanum A. americanum -96.9125099 39.1683292 5062

Amblyomma americanum americanum A. americanum -77.0722032 38.7804695 3686

Amblyomma americanum americanum A. americanum -85.4666405 35.7472878 5063

Amblyomma americanum americanum A. americanum -85.4666405 35.7472878 5063

Amblyomma americanum americanum A. americanum -79.1803017 35.4798756 5063

Amblyomma americanum americanum A. americanum -76.1131431 39.548738 4688

Amblyomma americanum americanum A. americanum -75.0987625 38.7719524 4000

Amblyomma americanum americanum A. americanum -78.972805 35.1676098 5099

Amblyomma americanum americanum A. americanum -76.2959883 39.4187183 5189

Amblyomma americanum americanum A. americanum -78.2933696 34.1051869 42199

Amblyomma americanum americanum A. americanum -74.208267 40.164578 5000

Amblyomma americanum americanum A. americanum -79.1803017 35.4798756 5063

Amblyomma americanum americanum A. americanum -86.4694366 36.3136616 1000

Amblyomma americanum americanum A. americanum -80.8271254 34.0399588 14989

Amblyomma americanum americanum A. americanum -77.4089934 38.4220676 5117

Amblyomma americanum americanum A. americanum -76.5835648 36.7282047 5063

Amblyomma americanum americanum A. americanum -76.3456386 37.0353572 5490

Amblyomma americanum americanum A. americanum -76.946064 35.9984913 5063

Amblyomma americanum americanum A. americanum -79.1803017 35.4798756 5063

Amblyomma americanum americanum A. americanum -77.2671266 38.6540242 4893

Amblyomma americanum americanum A. americanum -74.031389 40.200556 5000

Amblyomma americanum americanum A. americanum -74.5027542 39.9018353 11000

Amblyomma americanum americanum A. americanum -80.7941747 34.1711005 5075

Amblyomma americanum americanum A. americanum -79.1803017 35.4798756 5063

Amblyomma americanum americanum A. americanum -76.3133466 38.9539793 5655

Amblyomma americanum americanum A. americanum -77.4312973 39.4391096 3000

Amblyomma americanum americanum A. americanum -79.0450859 35.2887871 5000

Amblyomma americanum americanum A. americanum -78.8881989 35.0527 6468

Amblyomma americanum americanum A. americanum -76.0006026 39.5435483 28202

Amblyomma americanum americanum A. americanum -76.7074547 37.2707005 5062

Amblyomma americanum americanum A. americanum -76.5028111 37.0918568 16209

Amblyomma americanum americanum A. americanum -76.3456386 37.0353572 5490

Amblyomma americanum americanum A. americanum -76.5069081 37.0917413 16378

Amblyomma americanum americanum A. americanum -76.3482933 39.5359402 5062

Amblyomma americanum americanum A. americanum -87.4886208 36.8656044 5063

Amblyomma americanum americanum A. americanum -87.3594513 36.5297699 5063

Amblyomma americanum americanum A. americanum -77.9202766 38.9151096 5062

Amblyomma americanum americanum A. americanum -77.3255424 38.8009472 5062

Amblyomma americanum americanum A. americanum -77.3003651 38.6802599 5496

Amblyomma americanum americanum A. americanum -77.3050865 38.5344991 4853

Amblyomma americanum americanum A. americanum -77.2750854 38.1097082 13000

Amblyomma americanum americanum A. americanum -85.859127 37.6939526 5062

Amblyomma americanum americanum A. americanum -76.6310692 36.9823704 5063

Amblyomma americanum americanum A. americanum -77.2851026 37.2737314 6750

Amblyomma americanum americanum A. americanum -79.1803017 35.4798756 5063

Amblyomma americanum americanum A. americanum -78.2933696 34.1051869 42199

Amblyomma americanum americanum A. americanum -76.7438507 38.4429035 5062

Amblyomma americanum americanum A. americanum -76.5028111 37.0918568 16209

Amblyomma americanum americanum A. americanum -77.28825 38.8387894 26390

Amblyomma americanum americanum A. americanum -77.3840356 38.7803305 5249

Amblyomma americanum americanum A. americanum -77.2671266 38.6540242 4893

Amblyomma americanum americanum A. americanum -79.1803017 35.4798756 5063

Amblyomma americanum americanum A. americanum -87.5105705 36.5442162 5063

Amblyomma americanum americanum A. americanum -74.0509758 40.2962208 5062

Amblyomma americanum americanum A. americanum -77.2907679 38.9981632 5250

Amblyomma americanum americanum A. americanum -77.2277641 38.7042868 5063

Amblyomma americanum americanum A. americanum -76.3150061 39.5017853 32780

Amblyomma americanum americanum A. americanum -78.0226922 33.9229367 5334

Amblyomma americanum americanum A. americanum -77.0352237 38.8958606 5123

Amblyomma americanum americanum A. americanum -81.033777 34.0008472 5147

Amblyomma americanum americanum A. americanum -76.2959883 39.4187183 5189

Amblyomma americanum americanum A. americanum -92.0165367 34.9745312 5063

Amblyomma americanum americanum A. americanum -77.4888616 38.7683483 7325

Amblyomma americanum americanum A. americanum -77.3063698 38.8462238 5062

Amblyomma americanum americanum A. americanum -77.465504 38.535665 3000

Amblyomma americanum americanum A. americanum -76.6807867 37.2815893 5542

Amblyomma americanum americanum A. americanum -76.7074547 37.2707005 5062

Amblyomma americanum americanum A. americanum -94.9287936 39.3111076 5480

Amblyomma americanum americanum A. americanum -79.1803017 35.4798756 5063

Amblyomma americanum americanum A. americanum -104.7562912 38.8753274 18870

Amblyomma americanum americanum A. americanum -77.911263 37.056273 5000

Amblyomma americanum americanum A. americanum -76.5871351 38.2763004 35511

Amblyomma americanum americanum A. americanum -77.4602623 37.5537605 5062

Amblyomma americanum americanum A. americanum -77.4302444 34.754055 5063

Amblyomma americanum americanum A. americanum -77.0722032 38.7804695 3686

Amblyomma americanum americanum A. americanum -77.1963654 38.830389 5062

Amblyomma americanum americanum A. americanum -78.0226922 33.9229367 5334

Amblyomma americanum americanum A. americanum -76.7958823 37.5315342 5103

Amblyomma americanum americanum A. americanum -76.5125753 37.2304516 5499

Amblyomma americanum americanum A. americanum -76.419172 37.157848 500

Amblyomma americanum americanum A. americanum -76.5028111 37.0918568 16209

Amblyomma americanum americanum A. americanum -77.0432507 37.4504118 5577

Amblyomma americanum americanum A. americanum -77.1872063 38.7892799 5062

Amblyomma americanum americanum A. americanum -76.5125753 37.2304516 5499

Amblyomma americanum americanum A. americanum -74.0074043 40.4037189 5762

Amblyomma americanum americanum A. americanum -79.1803017 35.4798756 5063

Amblyomma americanum americanum A. americanum -77.0179365 38.7053604 5351

Amblyomma americanum americanum A. americanum -94.6225907 39.3868619 4819

Amblyomma americanum americanum A. americanum -97.6744843 38.5690774 5458

Amblyomma americanum americanum A. americanum -76.4721718 37.9176311 5062

Amblyomma americanum americanum A. americanum -78.972805 35.1676098 5099

Amblyomma americanum americanum A. americanum -78.8255653 35.8234825 5063

Amblyomma americanum americanum A. americanum -78.1249962 36.9620972 5063

Amblyomma americanum americanum A. americanum -83.3779335 33.9609451 5063

Amblyomma americanum americanum A. americanum -75.7739242 39.1432468 2852

Amblyomma americanum americanum A. americanum -100.4370384 31.4637737 5064

Amblyomma americanum americanum A. americanum -76.9752541 38.5292892 5062

Amblyomma americanum americanum A. americanum -76.7074547 37.2707005 5062

Amblyomma americanum americanum A. americanum -76.3456386 37.0353572 5490

Amblyomma americanum americanum A. americanum -88.4272652 33.4956722 5063

Amblyomma americanum americanum A. americanum -75.7716944 38.6998745 5501

Amblyomma americanum americanum A. americanum -76.1646169 39.5095539 5104

Amblyomma americanum americanum A. americanum -76.5679497 38.0603556 5172

Amblyomma americanum americanum A. americanum -95.923889 34.826667 10000

Amblyomma americanum americanum A. americanum -77.3840356 38.7803305 5249

Amblyomma americanum americanum A. americanum -78.2261086 38.6570644 5062

Amblyomma americanum americanum A. americanum -76.5905113 37.2840191 5064

Amblyomma americanum americanum A. americanum -76.5028111 37.0918568 16209

Amblyomma americanum americanum A. americanum -76.7074547 37.2707005 5062

Amblyomma americanum americanum A. americanum -75.5779839 39.0084496 5062

Amblyomma americanum americanum A. americanum -87.5105705 36.5442162 5063

Amblyomma americanum americanum A. americanum -85.9658626 37.8897647 5709

Amblyomma americanum americanum A. americanum -76.5028111 37.0918568 16209

Amblyomma americanum americanum A. americanum -78.8881989 35.0527 6468

Amblyomma americanum americanum A. americanum -76.3482933 39.5359402 5062

Amblyomma americanum americanum A. americanum -78.6386261 35.7720528 9703

Amblyomma americanum americanum A. americanum -77.915039 37.0420244 10500

Amblyomma americanum americanum A. americanum -77.2277641 38.7042868 5063

Amblyomma americanum americanum A. americanum -87.3594513 36.5297699 5063

Amblyomma americanum americanum A. americanum -82.837368 34.6834393 5063

Amblyomma americanum americanum A. americanum -76.6077232 36.0598271 5250

Amblyomma americanum americanum A. americanum -79.1803017 35.4798756 5063

Amblyomma americanum americanum A. americanum -77.915039 37.0420244 10500

Amblyomma americanum americanum A. americanum -76.2802426 36.7081798 25751

Amblyomma americanum americanum A. americanum -76.2802426 36.7081798 25751

Amblyomma americanum americanum A. americanum -87.8375206 36.6039058 5153

Amblyomma americanum americanum A. americanum -86.1694145 37.9899147 5706

Amblyomma americanum americanum A. americanum -77.915039 37.0420244 10500

Amblyomma americanum americanum A. americanum -76.8144209 38.6372087 4515

Amblyomma americanum americanum A. americanum -77.3255424 38.8009472 5062

Amblyomma americanum americanum A. americanum -76.3457756 37.1224925 5076

Amblyomma americanum americanum A. americanum -77.2671266 38.6540242 4893

Amblyomma americanum americanum A. americanum -77.915039 37.0420244 10500

Amblyomma americanum americanum A. americanum -75.3099098 38.7776146 5062

Amblyomma americanum americanum A. americanum -87.3594513 36.5297699 5063

Amblyomma americanum americanum A. americanum -77.2750854 38.1097082 13000

Amblyomma americanum americanum A. americanum -87.3594513 36.5297699 5063

Amblyomma americanum americanum A. americanum -77.2277641 38.7042868 5063

Amblyomma americanum americanum A. americanum -74.0616098 40.1331143 5195

Amblyomma americanum americanum A. americanum -77.915039 37.0420244 10500

Amblyomma americanum americanum A. americanum -77.4888616 38.7683483 7325

Amblyomma americanum americanum A. americanum -74.3112602 40.0145645 5062

Amblyomma americanum americanum A. americanum -74.3112602 40.0145645 5062

Amblyomma americanum americanum A. americanum -76.466053 36.1901569 5063

Amblyomma americanum americanum A. americanum -75.3099098 38.7776146 5062

Amblyomma americanum americanum A. americanum -76.2959883 39.4187183 5189

Amblyomma americanum americanum A. americanum -83.9879646 33.9562168 5063

Amblyomma americanum americanum A. americanum -84.6485405 33.3392849 5063

Amblyomma americanum americanum A. americanum -87.3594513 36.5297699 5063

Amblyomma americanum americanum A. americanum -85.9569206 37.1367149 5038

Amblyomma americanum americanum A. americanum -77.1459102 38.7021234 3500

Amblyomma americanum americanum A. americanum -77.28825 38.8387894 26390

Amblyomma americanum americanum A. americanum -77.3840356 38.7803305 5249

Amblyomma americanum americanum A. americanum -77.0552521 39.0398331 5062

Amblyomma americanum americanum A. americanum -75.5243645 39.158165 5062

Amblyomma americanum americanum A. americanum -75.4488068 39.2659473 8000

Amblyomma americanum americanum A. americanum -79.1803017 35.4798756 5063

Amblyomma americanum americanum A. americanum -76.6077232 36.0598271 5250

Amblyomma americanum americanum A. americanum -87.693611 36.500833 5000

Amblyomma americanum americanum A. americanum -81.033777 34.0008472 5147

Amblyomma americanum americanum A. americanum -87.3594513 36.5297699 5063

Amblyomma americanum americanum A. americanum -87.3594513 36.5297699 5063

Amblyomma americanum americanum A. americanum -77.5597115 37.1948632 5178

Amblyomma americanum americanum A. americanum -87.3594513 36.5297699 5063

Amblyomma americanum americanum A. americanum -77.4874954 39.0437183 5062

Amblyomma americanum americanum A. americanum -87.3594513 36.5297699 5063

Amblyomma americanum americanum A. americanum -76.3456386 37.0353572 5490
[truncated: 714,241 more chars]
