# Supplementary material for: Current and Future Distribution of the Lone Star Tick, Amblyomma americanum (L.) (Acari: Ixodidae) in North America
Source: PLoS One. 2019 Jan 2;14(1):e0209082. doi: 10.1371/journal.pone.0209082 (PMC6314611; doi:10.1371/journal.pone.0209082)
Supplement: S3 File — (DOCX) [file pone.0209082.s003.docx]

Supplementary material 3.

| Model | Partial ROC | Omission rate (E = 5%) | AICc | Delta AICc | W. AICc | N. parameters |
| --- | --- | --- | --- | --- | --- | --- |
| M_0.5_F_lq_Set2 | 0.00 | 0.04 | 3371.05 | 14.20 | 7.39E-07 | 10 |
| M_3_F_h_Set3 | 0.00 | 0.04 | 3373.41 | 16.56 | 4.95E-07 | 12 |
| M_0.1_F_lq_Set2 | 0.00 | 0.04 | 3374.16 | 17.31 | 1.24E-07 | 13 |
| M_2_F_pth_Set3 | 0.00 | 0.03 | 3374.32 | 17.47 | 2.97E-07 | 18 |
| M_0.2_F_lq_Set2 | 0.00 | 0.04 | 3374.35 | 17.50 | 1.17E-07 | 13 |
| M_0.4_F_lq_Set2 | 0.00 | 0.04 | 3374.58 | 17.74 | 1.17E-07 | 12 |
| M_2_F_th_Set3 | 0.00 | 0.03 | 3376.21 | 19.36 | 1.10E-07 | 18 |
| M_5_F_th_Set3 | 0.00 | 0.04 | 3377.35 | 20.50 | 1.15E-07 | 7 |
| M_4_F_th_Set3 | 0.00 | 0.04 | 3377.62 | 20.77 | 7.84E-08 | 11 |
| M_3_F_pth_Set3 | 0.00 | 0.04 | 3379.12 | 22.27 | 3.22E-08 | 16 |
| M_3_F_th_Set3 | 0.00 | 0.03 | 3379.54 | 22.70 | 2.45E-08 | 16 |
| M_2_F_h_Set3 | 0.00 | 0.04 | 3382.48 | 25.64 | 4.54E-09 | 19 |
| M_2_F_ph_Set3 | 0.00 | 0.04 | 3384.23 | 27.38 | 1.99E-09 | 20 |
| M_4_F_h_Set3 | 0.00 | 0.04 | 3389.80 | 32.95 | 1.65E-10 | 15 |
| M_5_F_h_Set3 | 0.00 | 0.04 | 3389.96 | 33.11 | 1.91E-10 | 11 |
| M_2_F_th_Set2 | 0.00 | 0.05 | 3365.40 | 8.55 | 2.44E-05 | 19 |
| M_0.1_F_lq_Set1 | 0.00 | 0.05 | 3367.70 | 10.85 | 3.12E-06 | 20 |
| M_2_F_lqh_Set3 | 0.00 | 0.05 | 3368.04 | 11.19 | 6.67E-06 | 13 |
| M_2_F_pth_Set2 | 0.00 | 0.05 | 3368.29 | 11.44 | 6.06E-06 | 21 |
| M_2_F_qph_Set3 | 0.00 | 0.05 | 3369.10 | 12.25 | 3.99E-06 | 15 |
| M_2_F_lqph_Set3 | 0.00 | 0.05 | 3369.36 | 12.51 | 3.59E-06 | 15 |
| M_3_F_ph_Set3 | 0.00 | 0.05 | 3371.61 | 14.76 | 1.29E-06 | 11 |
| M_5_F_th_Set2 | 0.00 | 0.05 | 3371.74 | 14.89 | 1.89E-06 | 5 |
| M_2_F_lqpth_Set3 | 0.00 | 0.05 | 3372.22 | 15.37 | 8.74E-07 | 18 |
| M_2_F_qh_Set3 | 0.00 | 0.05 | 3372.27 | 15.42 | 7.78E-07 | 15 |
| M_0.6_F_lq_Set2 | 0.00 | 0.05 | 3373.17 | 16.32 | 2.78E-07 | 10 |
| M_2_F_lh_Set3 | 0.00 | 0.05 | 3374.20 | 17.35 | 2.92E-07 | 15 |
| M_2_F_h_Set2 | 0.00 | 0.05 | 3374.65 | 17.80 | 2.28E-07 | 20 |
| M_0.3_F_lq_Set2 | 0.00 | 0.05 | 3376.07 | 19.22 | 5.25E-08 | 14 |
| M_2_F_lph_Set3 | 0.00 | 0.05 | 3377.28 | 20.43 | 6.63E-08 | 17 |
| M_1_F_lph_Set2 | 0.00 | 0.05 | 3379.09 | 22.24 | 2.32E-08 | 27 |
| M_0.9_F_q_Set1 | 0.00 | 0.05 | 3380.28 | 23.43 | 1.04E-08 | 9 |
| M_5_F_h_Set2 | 0.00 | 0.05 | 3380.59 | 23.74 | 2.07E-08 | 7 |
| M_1_F_q_Set1 | 0.00 | 0.05 | 3380.69 | 23.84 | 9.58E-09 | 9 |
| M_0.5_F_q_Set1 | 0.00 | 0.05 | 3381.53 | 24.68 | 3.87E-09 | 10 |
| M_0.7_F_q_Set1 | 0.00 | 0.05 | 3381.97 | 25.12 | 3.67E-09 | 10 |
| M_0.8_F_q_Set1 | 0.00 | 0.05 | 3382.28 | 25.43 | 3.46E-09 | 10 |
| M_2_F_lpth_Set3 | 0.00 | 0.05 | 3382.28 | 25.43 | 5.69E-09 | 21 |
| M_6_F_th_Set2 | 0.00 | 0.05 | 3382.31 | 25.46 | 1.34E-08 | 6 |
| M_4_F_pth_Set3 | 0.00 | 0.05 | 3382.34 | 25.49 | 7.95E-09 | 13 |
| M_0.1_F_q_Set1 | 0.00 | 0.05 | 3383.14 | 26.29 | 1.38E-09 | 11 |
| M_6_F_th_Set1 | 0.00 | 0.05 | 3383.22 | 26.37 | 8.47E-09 | 7 |
| M_0.2_F_q_Set1 | 0.00 | 0.05 | 3383.27 | 26.42 | 1.35E-09 | 11 |
| M_0.3_F_q_Set1 | 0.00 | 0.05 | 3383.46 | 26.61 | 1.29E-09 | 11 |
| M_0.4_F_q_Set1 | 0.00 | 0.05 | 3383.59 | 26.74 | 1.29E-09 | 11 |
| M_0.6_F_q_Set1 | 0.00 | 0.05 | 3384.00 | 27.15 | 1.22E-09 | 11 |
| M_6_F_h_Set2 | 0.00 | 0.05 | 3384.27 | 27.42 | 4.43E-09 | 5 |
| M_6_F_h_Set1 | 0.00 | 0.05 | 3385.01 | 28.16 | 3.04E-09 | 6 |
| M_6_F_qth_Set3 | 0.00 | 0.05 | 3386.90 | 30.05 | 1.52E-09 | 5 |
| M_8_F_h_Set1 | 0.00 | 0.05 | 3387.51 | 30.66 | 1.33E-09 | 3 |
| M_8_F_h_Set2 | 0.00 | 0.05 | 3387.52 | 30.67 | 1.34E-09 | 3 |
| M_8_F_th_Set1 | 0.00 | 0.05 | 3387.92 | 31.07 | 1.33E-09 | 4 |
| M_8_F_th_Set2 | 0.00 | 0.05 | 3387.92 | 31.07 | 1.34E-09 | 4 |
| M_5_F_pth_Set3 | 0.00 | 0.05 | 3388.88 | 32.03 | 3.96E-10 | 11 |
| M_6_F_pth_Set3 | 0.00 | 0.05 | 3388.93 | 32.08 | 5.60E-10 | 8 |
| M_5_F_ph_Set3 | 0.00 | 0.05 | 3389.04 | 32.19 | 3.29E-10 | 10 |
| M_6_F_qh_Set3 | 0.00 | 0.05 | 3389.16 | 32.31 | 4.21E-10 | 6 |
| M_4_F_ph_Set3 | 0.00 | 0.05 | 3390.89 | 34.04 | 1.02E-10 | 15 |
| M_6_F_ph_Set3 | 0.00 | 0.05 | 3391.90 | 35.05 | 1.10E-10 | 9 |
| M_6_F_h_Set3 | 0.00 | 0.05 | 3394.36 | 37.51 | 2.87E-11 | 10 |
| M_0.8_F_lqph_Set2 | 0.00 | 0.05 | 3402.00 | 45.15 | 1.98E-13 | 36 |
| M_0.8_F_qph_Set2 | 0.00 | 0.05 | 3402.23 | 45.38 | 1.73E-13 | 36 |
| M_2_F_lpth_Set2 | 0.00 | 0.07 | 3363.26 | 6.41 | 7.66E-05 | 19 |
| M_1_F_lqp_Set2 | 0.00 | 0.07 | 3364.67 | 7.82 | 3.07E-05 | 11 |
| M_0.8_F_lqp_Set2 | 0.00 | 0.07 | 3367.06 | 10.21 | 7.35E-06 | 12 |
| M_1_F_lqph_Set2 | 0.00 | 0.07 | 3368.77 | 11.92 | 4.13E-06 | 23 |
| M_0.9_F_lqp_Set2 | 0.00 | 0.07 | 3369.23 | 12.38 | 2.78E-06 | 13 |
| M_0.8_F_lq_Set1 | 0.00 | 0.07 | 3369.24 | 12.39 | 2.38E-06 | 15 |
| M_2_F_qth_Set3 | 0.00 | 0.07 | 3370.31 | 13.46 | 2.20E-06 | 16 |
| M_0.3_F_lq_Set3 | 0.00 | 0.07 | 3370.46 | 13.61 | 8.70E-07 | 7 |
| M_2_F_p_Set1 | 0.00 | 0.07 | 3371.00 | 14.15 | 1.40E-06 | 12 |
| M_0.1_F_lq_Set3 | 0.00 | 0.07 | 3371.61 | 14.76 | 4.43E-07 | 8 |
| M_0.2_F_lq_Set3 | 0.00 | 0.07 | 3371.95 | 15.10 | 3.90E-07 | 8 |
| M_2_F_lqth_Set3 | 0.00 | 0.07 | 3372.76 | 15.91 | 6.61E-07 | 17 |
| M_2_F_lp_Set2 | 0.00 | 0.07 | 3372.79 | 15.94 | 5.83E-07 | 8 |
| M_5_F_th_Set1 | 0.00 | 0.07 | 3373.24 | 16.39 | 8.92E-07 | 7 |
| M_0.7_F_lq_Set2 | 0.00 | 0.07 | 3373.67 | 16.82 | 2.36E-07 | 10 |
| M_0.8_F_lq_Set2 | 0.00 | 0.07 | 3374.52 | 17.67 | 1.70E-07 | 10 |
| M_3_F_lqp_Set2 | 0.00 | 0.07 | 3375.17 | 18.32 | 2.19E-07 | 7 |
| M_3_F_qh_Set3 | 0.00 | 0.07 | 3376.18 | 19.33 | 1.29E-07 | 13 |
| M_2_F_lqp_Set3 | 0.00 | 0.07 | 3377.55 | 20.70 | 5.66E-08 | 8 |
| M_3_F_qph_Set3 | 0.00 | 0.07 | 3378.44 | 21.59 | 4.45E-08 | 14 |
| M_3_F_lqpth_Set2 | 0.00 | 0.07 | 3378.47 | 21.62 | 4.59E-08 | 20 |
| M_1_F_ph_Set2 | 0.00 | 0.07 | 3379.14 | 22.29 | 2.19E-08 | 27 |
| M_5_F_h_Set1 | 0.00 | 0.07 | 3379.26 | 22.41 | 4.01E-08 | 8 |
| M_1_F_qph_Set2 | 0.00 | 0.07 | 3379.50 | 22.65 | 1.90E-08 | 27 |
| M_3_F_qth_Set3 | 0.00 | 0.07 | 3379.51 | 22.66 | 2.63E-08 | 16 |
| M_3_F_p_Set1 | 0.00 | 0.07 | 3379.69 | 22.84 | 2.11E-08 | 13 |
| M_3_F_qph_Set2 | 0.00 | 0.07 | 3379.80 | 22.95 | 2.25E-08 | 18 |
| M_5_F_lqph_Set3 | 0.00 | 0.07 | 3379.90 | 23.05 | 3.60E-08 | 5 |
| M_3_F_lqph_Set3 | 0.00 | 0.07 | 3380.63 | 23.78 | 1.53E-08 | 15 |
| M_4_F_p_Set1 | 0.00 | 0.07 | 3381.24 | 24.39 | 1.17E-08 | 11 |
| M_4_F_lh_Set3 | 0.00 | 0.07 | 3381.44 | 24.59 | 1.11E-08 | 9 |
| M_3_F_lpt_Set3 | 0.00 | 0.07 | 3381.76 | 24.91 | 8.31E-09 | 10 |
| M_5_F_lqpth_Set3 | 0.00 | 0.07 | 3381.82 | 24.97 | 1.43E-08 | 6 |
| M_5_F_lph_Set3 | 0.00 | 0.07 | 3382.58 | 25.73 | 8.85E-09 | 6 |
| M_0.9_F_lph_Set2 | 0.00 | 0.07 | 3382.79 | 25.94 | 3.21E-09 | 29 |
| M_6_F_lh_Set3 | 0.00 | 0.07 | 3383.19 | 26.34 | 8.03E-09 | 4 |
| M_6_F_lpth_Set3 | 0.00 | 0.07 | 3383.19 | 26.34 | 1.05E-08 | 4 |
| M_5_F_lqph_Set2 | 0.00 | 0.07 | 3383.46 | 26.61 | 6.08E-09 | 9 |
| M_4_F_pt_Set1 | 0.00 | 0.07 | 3384.08 | 27.23 | 3.05E-09 | 13 |
| M_2_F_lq_Set3 | 0.00 | 0.07 | 3384.16 | 27.31 | 1.98E-09 | 5 |
| M_3_F_lqph_Set2 | 0.00 | 0.07 | 3384.19 | 27.34 | 2.58E-09 | 20 |
| M_6_F_th_Set3 | 0.00 | 0.07 | 3384.19 | 27.34 | 5.25E-09 | 7 |
| M_4_F_qh_Set3 | 0.00 | 0.07 | 3384.27 | 27.42 | 2.75E-09 | 12 |
| M_4_F_lqph_Set3 | 0.00 | 0.07 | 3384.44 | 27.59 | 2.82E-09 | 11 |
| M_5_F_lpth_Set3 | 0.00 | 0.07 | 3384.44 | 27.60 | 3.80E-09 | 7 |
| M_5_F_lqpth_Set2 | 0.00 | 0.07 | 3384.54 | 27.69 | 3.65E-09 | 10 |
| M_5_F_qph_Set2 | 0.00 | 0.07 | 3384.57 | 27.72 | 3.33E-09 | 9 |
| M_1_F_h_Set2 | 0.00 | 0.07 | 3384.64 | 27.79 | 1.35E-09 | 27 |
| M_6_F_lqth_Set3 | 0.00 | 0.07 | 3384.74 | 27.89 | 4.78E-09 | 5 |
| M_6_F_lqh_Set3 | 0.00 | 0.07 | 3384.74 | 27.89 | 4.16E-09 | 5 |
| M_6_F_lqpth_Set3 | 0.00 | 0.07 | 3384.74 | 27.89 | 4.93E-09 | 5 |
| M_6_F_ph_Set2 | 0.00 | 0.07 | 3384.87 | 28.02 | 3.68E-09 | 6 |
| M_3_F_lqpth_Set3 | 0.00 | 0.07 | 3385.28 | 28.43 | 1.53E-09 | 18 |
| M_6_F_lph_Set3 | 0.00 | 0.07 | 3385.29 | 28.45 | 3.25E-09 | 5 |
| M_3_F_lqp_Set3 | 0.00 | 0.07 | 3385.55 | 28.70 | 1.22E-09 | 5 |
| M_0.9_F_ph_Set2 | 0.00 | 0.07 | 3385.88 | 29.03 | 6.67E-10 | 30 |
| M_0.9_F_qph_Set2 | 0.00 | 0.07 | 3386.13 | 29.28 | 6.07E-10 | 30 |
| M_4_F_qth_Set3 | 0.00 | 0.07 | 3386.16 | 29.31 | 1.17E-09 | 14 |
| M_6_F_pth_Set2 | 0.00 | 0.07 | 3386.60 | 29.75 | 1.79E-09 | 7 |
| M_4_F_qph_Set3 | 0.00 | 0.07 | 3386.63 | 29.78 | 9.14E-10 | 13 |
| M_6_F_lqph_Set3 | 0.00 | 0.07 | 3386.88 | 30.03 | 1.61E-09 | 6 |
| M_4_F_lpth_Set3 | 0.00 | 0.07 | 3387.10 | 30.25 | 7.59E-10 | 13 |
| M_5_F_qth_Set3 | 0.00 | 0.07 | 3387.26 | 30.41 | 8.80E-10 | 9 |
| M_3_F_lpt_Set2 | 0.00 | 0.07 | 3387.38 | 30.53 | 4.99E-10 | 18 |
| M_5_F_qph_Set3 | 0.00 | 0.07 | 3387.50 | 30.65 | 7.72E-10 | 9 |
| M_5_F_qh_Set3 | 0.00 | 0.07 | 3387.52 | 30.67 | 6.92E-10 | 9 |
| M_6_F_lqth_Set2 | 0.00 | 0.07 | 3387.88 | 31.03 | 9.90E-10 | 8 |
| M_3_F_q_Set1 | 0.00 | 0.07 | 3388.42 | 31.57 | 2.67E-10 | 8 |
| M_1_F_h_Set3 | 0.00 | 0.07 | 3388.56 | 31.71 | 1.90E-10 | 24 |
| M_1_F_lqth_Set3 | 0.00 | 0.07 | 3388.60 | 31.75 | 2.07E-10 | 31 |
| M_4_F_lqth_Set3 | 0.00 | 0.07 | 3388.78 | 31.93 | 3.26E-10 | 14 |
| M_8_F_lph_Set3 | 0.00 | 0.07 | 3388.87 | 32.02 | 9.46E-10 | 4 |
| M_8_F_lqpth_Set3 | 0.00 | 0.07 | 3388.87 | 32.02 | 1.23E-09 | 4 |
| M_8_F_lqph_Set3 | 0.00 | 0.07 | 3388.87 | 32.02 | 1.11E-09 | 4 |
| M_8_F_lpth_Set3 | 0.00 | 0.07 | 3388.88 | 32.04 | 1.18E-09 | 4 |
| M_4_F_qth_Set2 | 0.00 | 0.07 | 3388.90 | 32.05 | 2.96E-10 | 17 |
| M_8_F_qth_Set3 | 0.00 | 0.07 | 3388.92 | 32.07 | 9.99E-10 | 3 |
| M_4_F_lqpth_Set3 | 0.00 | 0.07 | 3389.13 | 32.28 | 2.77E-10 | 14 |
| M_1_F_pth_Set3 | 0.00 | 0.07 | 3389.26 | 32.41 | 1.46E-10 | 33 |
| M_4_F_qh_Set2 | 0.00 | 0.07 | 3389.98 | 33.14 | 1.58E-10 | 16 |
| M_6_F_lqh_Set2 | 0.00 | 0.07 | 3390.17 | 33.32 | 2.75E-10 | 9 |
| M_8_F_qth_Set2 | 0.00 | 0.07 | 3390.69 | 33.84 | 4.09E-10 | 6 |
| M_8_F_qh_Set3 | 0.00 | 0.07 | 3391.01 | 34.16 | 2.69E-10 | 4 |
| M_8_F_qph_Set3 | 0.00 | 0.07 | 3391.01 | 34.16 | 3.41E-10 | 4 |
| M_0.7_F_ph_Set2 | 0.00 | 0.07 | 3391.31 | 34.46 | 3.58E-11 | 34 |
| M_0.8_F_lph_Set2 | 0.00 | 0.07 | 3391.74 | 34.89 | 3.26E-11 | 33 |
| M_4_F_qt_Set3 | 0.00 | 0.07 | 3392.05 | 35.20 | 5.61E-11 | 8 |
| M_2_F_ph_Set2 | 0.00 | 0.07 | 3392.41 | 35.56 | 3.31E-11 | 27 |
| M_1_F_lpth_Set3 | 0.00 | 0.07 | 3392.45 | 35.60 | 3.02E-11 | 34 |
| M_8_F_qh_Set2 | 0.00 | 0.07 | 3392.86 | 36.01 | 1.06E-10 | 7 |
| M_8_F_lqh_Set2 | 0.00 | 0.07 | 3393.30 | 36.45 | 9.72E-11 | 7 |
| M_8_F_lqth_Set2 | 0.00 | 0.07 | 3393.34 | 36.49 | 1.22E-10 | 7 |
| M_2_F_p_Set2 | 0.00 | 0.07 | 3393.52 | 36.67 | 1.80E-11 | 10 |
| M_4_F_lt_Set3 | 0.00 | 0.07 | 3394.40 | 37.55 | 1.69E-11 | 9 |
| M_4_F_lqt_Set3 | 0.00 | 0.07 | 3394.42 | 37.57 | 1.79E-11 | 9 |
| M_0.9_F_lqph_Set2 | 0.00 | 0.07 | 3394.99 | 38.14 | 7.35E-12 | 33 |
| M_10_F_qth_Set3 | 0.00 | 0.07 | 3395.20 | 38.35 | 2.16E-10 | 3 |
| M_4_F_qpt_Set3 | 0.00 | 0.07 | 3395.59 | 38.74 | 1.03E-11 | 9 |
| M_8_F_th_Set3 | 0.00 | 0.07 | 3396.99 | 40.14 | 1.45E-11 | 7 |
| M_10_F_lh_Set1 | 0.00 | 0.07 | 3397.21 | 40.36 | 2.62E-11 | 7 |
| M_10_F_qh_Set3 | 0.00 | 0.07 | 3397.29 | 40.44 | 3.02E-11 | 4 |
| M_4_F_lqpt_Set3 | 0.00 | 0.07 | 3397.35 | 40.50 | 4.42E-12 | 11 |
| M_0.3_F_lp_Set3 | 0.00 | 0.07 | 3397.75 | 40.90 | 1.03E-12 | 9 |
| M_4_F_lpt_Set3 | 0.00 | 0.07 | 3397.91 | 41.06 | 3.17E-12 | 11 |
| M_0.4_F_lp_Set3 | 0.00 | 0.07 | 3398.05 | 41.20 | 9.45E-13 | 9 |
| M_10_F_qh_Set2 | 0.00 | 0.07 | 3398.15 | 41.30 | 1.92E-11 | 6 |
| M_10_F_qth_Set2 | 0.00 | 0.07 | 3398.17 | 41.32 | 4.66E-11 | 6 |
| M_0.5_F_lp_Set3 | 0.00 | 0.07 | 3398.91 | 42.06 | 6.60E-13 | 9 |
| M_0.1_F_lp_Set3 | 0.00 | 0.07 | 3399.08 | 42.23 | 4.81E-13 | 10 |
| M_0.2_F_lp_Set3 | 0.00 | 0.07 | 3399.12 | 42.27 | 4.92E-13 | 10 |
| M_10_F_qph_Set3 | 0.00 | 0.07 | 3399.19 | 42.34 | 2.58E-11 | 4 |
| M_0.6_F_lp_Set3 | 0.00 | 0.07 | 3399.82 | 42.97 | 4.55E-13 | 9 |
| M_6_F_lqt_Set2 | 0.00 | 0.07 | 3400.88 | 44.03 | 1.28E-12 | 7 |
| M_5_F_qt_Set3 | 0.00 | 0.07 | 3401.10 | 44.25 | 7.70E-13 | 7 |
| M_10_F_lqh_Set2 | 0.00 | 0.07 | 3401.41 | 44.56 | 5.58E-12 | 6 |
| M_0.8_F_ph_Set2 | 0.00 | 0.07 | 3401.54 | 44.69 | 2.38E-13 | 36 |
| M_4_F_pt_Set2 | 0.00 | 0.07 | 3401.79 | 44.94 | 4.35E-13 | 16 |
| M_3_F_lq_Set3 | 0.00 | 0.07 | 3401.85 | 45.00 | 3.33E-13 | 6 |
| M_4_F_lt_Set2 | 0.00 | 0.07 | 3402.19 | 45.34 | 3.44E-13 | 15 |
| M_0.7_F_lp_Set3 | 0.00 | 0.07 | 3402.19 | 45.34 | 1.52E-13 | 9 |
| M_10_F_th_Set3 | 0.00 | 0.07 | 3402.97 | 46.12 | 2.11E-12 | 6 |
| M_0.8_F_lp_Set3 | 0.00 | 0.07 | 3403.47 | 46.62 | 8.85E-14 | 9 |
| M_10_F_lqth_Set2 | 0.00 | 0.07 | 3403.60 | 46.75 | 6.50E-12 | 7 |
| M_5_F_qpt_Set3 | 0.00 | 0.07 | 3404.35 | 47.50 | 1.67E-13 | 7 |
| M_10_F_h_Set3 | 0.00 | 0.07 | 3405.83 | 48.98 | 3.05E-13 | 7 |
| M_0.1_F_q_Set2 | 0.00 | 0.07 | 3406.11 | 49.26 | 1.42E-14 | 7 |
| M_0.2_F_q_Set2 | 0.00 | 0.07 | 3406.16 | 49.31 | 1.44E-14 | 7 |
| M_8_F_h_Set3 | 0.00 | 0.07 | 3406.36 | 49.51 | 1.09E-13 | 10 |
| M_0.9_F_lp_Set3 | 0.00 | 0.07 | 3406.59 | 49.74 | 2.07E-14 | 9 |
| M_0.8_F_q_Set2 | 0.00 | 0.07 | 3407.32 | 50.47 | 1.26E-14 | 7 |
| M_0.9_F_q_Set2 | 0.00 | 0.07 | 3407.66 | 50.81 | 1.18E-14 | 7 |
| M_1_F_q_Set2 | 0.00 | 0.07 | 3407.99 | 51.14 | 1.13E-14 | 7 |
| M_1_F_lp_Set3 | 0.00 | 0.07 | 3408.03 | 51.18 | 1.13E-14 | 9 |
| M_6_F_qt_Set2 | 0.00 | 0.07 | 3411.04 | 54.19 | 7.34E-15 | 6 |
| M_8_F_lqt_Set2 | 0.00 | 0.07 | 3411.31 | 54.46 | 1.17E-14 | 7 |
| M_5_F_lpt_Set3 | 0.00 | 0.07 | 3412.56 | 55.71 | 2.71E-15 | 10 |
| M_5_F_qt_Set2 | 0.00 | 0.07 | 3412.71 | 55.86 | 2.32E-15 | 10 |
| M_0.6_F_ph_Set2 | 0.00 | 0.07 | 3413.21 | 56.36 | 5.75E-16 | 42 |
| M_3_F_q_Set2 | 0.00 | 0.07 | 3414.17 | 57.32 | 6.85E-16 | 5 |
| M_5_F_lqpt_Set3 | 0.00 | 0.07 | 3414.68 | 57.83 | 9.99E-16 | 11 |
| M_6_F_qt_Set3 | 0.00 | 0.07 | 3415.45 | 58.60 | 8.12E-16 | 7 |
| M_2_F_qp_Set3 | 0.00 | 0.07 | 3415.73 | 58.88 | 2.81E-16 | 7 |
| M_6_F_qpt_Set3 | 0.00 | 0.07 | 3415.79 | 58.94 | 7.86E-16 | 6 |
| M_0.6_F_qph_Set2 | 0.00 | 0.07 | 3416.69 | 59.84 | 1.03E-16 | 43 |
| M_0.7_F_lqph_Set2 | 0.00 | 0.07 | 3418.12 | 61.27 | 5.61E-17 | 42 |
| M_5_F_q_Set2 | 0.00 | 0.07 | 3418.65 | 61.80 | 1.10E-16 | 4 |
| M_10_F_lqt_Set2 | 0.00 | 0.07 | 3419.08 | 62.23 | 7.51E-16 | 6 |
| M_4_F_q_Set2 | 0.00 | 0.07 | 3419.10 | 62.25 | 7.01E-17 | 5 |
| M_6_F_lqpt_Set3 | 0.00 | 0.07 | 3419.59 | 62.74 | 1.25E-16 | 8 |
| M_0.6_F_lph_Set2 | 0.00 | 0.07 | 3419.65 | 62.80 | 2.34E-17 | 44 |
| M_6_F_q_Set2 | 0.00 | 0.07 | 3419.73 | 62.88 | 8.52E-17 | 4 |
| M_8_F_lq_Set2 | 0.00 | 0.07 | 3419.79 | 62.94 | 1.34E-16 | 6 |
| M_6_F_lq_Set2 | 0.00 | 0.07 | 3419.84 | 62.99 | 8.48E-17 | 8 |
| M_8_F_qt_Set2 | 0.00 | 0.07 | 3420.10 | 63.25 | 1.27E-16 | 6 |
| M_3_F_lp_Set3 | 0.00 | 0.07 | 3421.27 | 64.42 | 2.03E-17 | 5 |
| M_8_F_q_Set2 | 0.00 | 0.07 | 3422.23 | 65.38 | 3.67E-17 | 4 |
| M_2_F_lp_Set3 | 0.00 | 0.07 | 3422.45 | 65.60 | 9.64E-18 | 8 |
| M_10_F_lq_Set2 | 0.00 | 0.07 | 3422.52 | 65.67 | 7.46E-17 | 6 |
| M_0.5_F_lph_Set2 | 0.00 | 0.07 | 3423.00 | 66.15 | 4.03E-18 | 46 |
| M_0.6_F_lqph_Set2 | 0.00 | 0.07 | 3424.02 | 67.17 | 2.67E-18 | 45 |
| M_10_F_q_Set2 | 0.00 | 0.07 | 3425.10 | 68.25 | 1.76E-17 | 4 |
| M_10_F_qt_Set2 | 0.00 | 0.07 | 3425.54 | 68.69 | 2.06E-17 | 5 |
| M_0.2_F_l_Set2 | 0.00 | 0.07 | 3426.49 | 69.64 | 5.54E-19 | 6 |
| M_0.3_F_l_Set2 | 0.00 | 0.07 | 3426.60 | 69.75 | 5.54E-19 | 6 |
| M_0.4_F_l_Set2 | 0.00 | 0.07 | 3426.75 | 69.90 | 5.46E-19 | 6 |
| M_0.5_F_l_Set2 | 0.00 | 0.07 | 3426.93 | 70.08 | 5.37E-19 | 6 |
| M_0.6_F_l_Set2 | 0.00 | 0.07 | 3427.12 | 70.27 | 5.27E-19 | 6 |
| M_0.5_F_h_Set2 | 0.00 | 0.07 | 3427.89 | 71.04 | 3.35E-19 | 46 |
| M_0.1_F_l_Set2 | 0.00 | 0.07 | 3428.56 | 71.71 | 1.89E-19 | 7 |
| M_8_F_l_Set1 | 0.00 | 0.07 | 3429.23 | 72.38 | 1.09E-18 | 5 |
| M_0.1_F_l_Set3 | 0.00 | 0.07 | 3429.37 | 72.52 | 1.26E-19 | 4 |
| M_0.2_F_l_Set3 | 0.00 | 0.07 | 3429.41 | 72.56 | 1.29E-19 | 4 |
| M_0.3_F_l_Set3 | 0.00 | 0.07 | 3429.47 | 72.62 | 1.32E-19 | 4 |
| M_0.5_F_ph_Set2 | 0.00 | 0.07 | 3430.45 | 73.61 | 9.52E-20 | 48 |
| M_8_F_qpt_Set3 | 0.00 | 0.07 | 3430.60 | 73.75 | 8.41E-19 | 4 |
| M_8_F_lt_Set1 | 0.00 | 0.07 | 3430.85 | 74.00 | 5.47E-19 | 8 |
| M_8_F_qt_Set3 | 0.00 | 0.07 | 3431.62 | 74.77 | 4.02E-19 | 6 |
| M_0.7_F_qph_Set2 | 0.00 | 0.07 | 3431.96 | 75.11 | 5.47E-20 | 46 |
| M_0.5_F_lqh_Set2 | 0.00 | 0.07 | 3432.89 | 76.04 | 2.85E-20 | 48 |
| M_0.5_F_lh_Set2 | 0.00 | 0.07 | 3433.08 | 76.23 | 2.53E-20 | 48 |
| M_10_F_l_Set1 | 0.00 | 0.07 | 3433.76 | 76.91 | 2.22E-19 | 5 |
| M_10_F_lt_Set1 | 0.00 | 0.07 | 3435.86 | 79.01 | 1.01E-19 | 7 |
| M_8_F_pt_Set3 | 0.00 | 0.07 | 3436.29 | 79.44 | 4.04E-20 | 7 |
| M_3_F_qp_Set3 | 0.00 | 0.07 | 3436.80 | 79.96 | 8.76E-21 | 5 |
| M_4_F_qp_Set3 | 0.00 | 0.07 | 3439.34 | 82.49 | 2.99E-21 | 4 |
| M_10_F_qt_Set3 | 0.00 | 0.07 | 3439.44 | 82.59 | 2.01E-20 | 4 |
| M_10_F_qpt_Set3 | 0.00 | 0.07 | 3440.14 | 83.29 | 2.94E-20 | 4 |
| M_5_F_qp_Set3 | 0.00 | 0.07 | 3441.66 | 84.81 | 1.19E-21 | 4 |
| M_6_F_qp_Set3 | 0.00 | 0.07 | 3442.13 | 85.28 | 1.29E-21 | 3 |
| M_2_F_q_Set3 | 0.00 | 0.07 | 3443.77 | 86.92 | 2.20E-22 | 2 |
| M_3_F_q_Set3 | 0.00 | 0.07 | 3443.96 | 87.11 | 2.33E-22 | 2 |
| M_4_F_q_Set3 | 0.00 | 0.07 | 3444.21 | 87.36 | 2.47E-22 | 2 |
| M_5_F_q_Set3 | 0.00 | 0.07 | 3444.51 | 87.66 | 2.66E-22 | 2 |
| M_6_F_q_Set3 | 0.00 | 0.07 | 3444.86 | 88.01 | 2.99E-22 | 2 |
| M_0.4_F_q_Set3 | 0.00 | 0.07 | 3445.43 | 88.58 | 4.79E-23 | 3 |
| M_0.5_F_q_Set3 | 0.00 | 0.07 | 3445.46 | 88.61 | 5.09E-23 | 3 |
| M_0.6_F_q_Set3 | 0.00 | 0.07 | 3445.49 | 88.64 | 5.42E-23 | 3 |
| M_0.7_F_q_Set3 | 0.00 | 0.07 | 3445.53 | 88.68 | 5.81E-23 | 3 |
| M_0.8_F_q_Set3 | 0.00 | 0.07 | 3445.56 | 88.71 | 6.28E-23 | 3 |
| M_0.9_F_q_Set3 | 0.00 | 0.07 | 3445.60 | 88.75 | 6.85E-23 | 3 |
| M_1_F_q_Set3 | 0.00 | 0.07 | 3445.65 | 88.80 | 7.53E-23 | 3 |
| M_10_F_pt_Set3 | 0.00 | 0.07 | 3446.25 | 89.40 | 7.46E-22 | 6 |
| M_8_F_qp_Set3 | 0.00 | 0.07 | 3447.26 | 90.41 | 1.58E-22 | 3 |
| M_0.1_F_q_Set3 | 0.00 | 0.07 | 3447.45 | 90.60 | 1.49E-23 | 4 |
| M_0.2_F_q_Set3 | 0.00 | 0.07 | 3447.47 | 90.62 | 1.54E-23 | 4 |
| M_0.3_F_q_Set3 | 0.00 | 0.07 | 3447.50 | 90.65 | 1.61E-23 | 4 |
| M_8_F_q_Set3 | 0.00 | 0.07 | 3447.76 | 90.92 | 1.05E-22 | 3 |
| M_10_F_q_Set3 | 0.00 | 0.07 | 3448.83 | 91.98 | 1.26E-22 | 3 |
| M_10_F_qp_Set3 | 0.00 | 0.07 | 3450.56 | 93.71 | 7.36E-23 | 4 |
| M_5_F_p_Set3 | 0.00 | 0.07 | 3471.62 | 114.77 | 3.50E-28 | 4 |
| M_4_F_p_Set3 | 0.00 | 0.07 | 3472.79 | 115.94 | 1.55E-28 | 5 |
| M_6_F_p_Set3 | 0.00 | 0.07 | 3474.16 | 117.31 | 1.31E-28 | 4 |
| M_10_F_p_Set3 | 0.00 | 0.07 | 3477.09 | 120.24 | 9.49E-29 | 3 |
| M_8_F_p_Set3 | 0.00 | 0.07 | 3478.48 | 121.63 | 2.29E-29 | 4 |
| M_0.5_F_lq_Set1 | 0.00 | 0.08 | 3356.85 | 0.00 | 0.000895 | 16 |
| M_0.2_F_lq_Set1 | 0.00 | 0.08 | 3357.40 | 0.55 | 0.000564 | 17 |
| M_0.6_F_lqp_Set3 | 0.00 | 0.08 | 3359.21 | 2.36 | 0.000308 | 8 |
| M_0.7_F_lqp_Set3 | 0.00 | 0.08 | 3359.76 | 2.91 | 0.000256 | 8 |
| M_0.5_F_lqp_Set3 | 0.00 | 0.08 | 3360.65 | 3.80 | 0.000138 | 9 |
| M_0.8_F_lqp_Set3 | 0.00 | 0.08 | 3360.92 | 4.07 | 0.000159 | 8 |
| M_0.9_F_lqp_Set3 | 0.00 | 0.08 | 3361.78 | 4.93 | 0.000115 | 8 |
| M_0.3_F_lq_Set1 | 0.00 | 0.08 | 3361.90 | 5.05 | 6.27E-05 | 17 |
| M_1_F_lp_Set2 | 0.00 | 0.08 | 3362.26 | 5.41 | 9.83E-05 | 10 |
| M_0.4_F_lq_Set1 | 0.00 | 0.08 | 3362.38 | 5.53 | 5.23E-05 | 18 |
| M_0.4_F_lqp_Set3 | 0.00 | 0.08 | 3362.46 | 5.61 | 5.16E-05 | 10 |
| M_0.7_F_lq_Set1 | 0.00 | 0.08 | 3362.65 | 5.80 | 5.82E-05 | 16 |
| M_1_F_lqp_Set3 | 0.00 | 0.08 | 3362.75 | 5.90 | 8.05E-05 | 8 |
| M_2_F_lqth_Set2 | 0.00 | 0.08 | 3362.92 | 6.07 | 9.02E-05 | 18 |
| M_0.9_F_lp_Set2 | 0.00 | 0.08 | 3363.20 | 6.36 | 5.44E-05 | 11 |
| M_0.3_F_lqp_Set3 | 0.00 | 0.08 | 3363.36 | 6.51 | 3.09E-05 | 11 |
| M_2_F_lqpth_Set2 | 0.00 | 0.08 | 3364.52 | 7.67 | 4.09E-05 | 20 |
| M_0.7_F_lp_Set2 | 0.00 | 0.08 | 3365.59 | 8.75 | 1.34E-05 | 13 |
| M_0.8_F_lp_Set2 | 0.00 | 0.08 | 3366.54 | 9.69 | 9.22E-06 | 13 |
| M_0.9_F_lq_Set1 | 0.00 | 0.08 | 3366.58 | 9.73 | 1.00E-05 | 14 |
| M_0.1_F_lqp_Set3 | 0.00 | 0.08 | 3366.88 | 10.03 | 4.80E-06 | 13 |
| M_0.2_F_lqp_Set3 | 0.00 | 0.08 | 3367.07 | 10.22 | 4.56E-06 | 13 |
| M_2_F_qth_Set2 | 0.00 | 0.08 | 3367.53 | 10.68 | 8.81E-06 | 20 |
| M_2_F_lq_Set1 | 0.00 | 0.08 | 3367.64 | 10.79 | 7.59E-06 | 9 |
| M_0.6_F_lqp_Set2 | 0.00 | 0.08 | 3368.76 | 11.91 | 2.59E-06 | 14 |
| M_0.5_F_lp_Set2 | 0.00 | 0.08 | 3369.15 | 12.30 | 1.92E-06 | 15 |
| M_1_F_lqh_Set2 | 0.00 | 0.08 | 3369.55 | 12.70 | 2.70E-06 | 23 |
| M_0.7_F_lqp_Set2 | 0.00 | 0.08 | 3369.68 | 12.83 | 1.79E-06 | 14 |
| M_3_F_lq_Set1 | 0.00 | 0.08 | 3369.74 | 12.89 | 3.12E-06 | 8 |
| M_2_F_lqh_Set2 | 0.00 | 0.08 | 3370.29 | 13.44 | 2.16E-06 | 18 |
| M_1_F_lq_Set1 | 0.00 | 0.08 | 3370.47 | 13.62 | 1.61E-06 | 15 |
| M_2_F_qp_Set1 | 0.00 | 0.08 | 3370.54 | 13.69 | 1.82E-06 | 12 |
| M_3_F_qth_Set1 | 0.00 | 0.08 | 3371.15 | 14.30 | 1.71E-06 | 19 |
| M_4_F_th_Set1 | 0.00 | 0.08 | 3371.70 | 14.85 | 1.50E-06 | 12 |
| M_3_F_h_Set1 | 0.00 | 0.08 | 3373.12 | 16.27 | 5.71E-07 | 17 |
| M_0.5_F_lq_Set3 | 0.00 | 0.08 | 3373.57 | 16.72 | 2.10E-07 | 8 |
| M_0.4_F_lq_Set3 | 0.00 | 0.08 | 3373.64 | 16.79 | 1.88E-07 | 8 |
| M_0.9_F_lq_Set2 | 0.00 | 0.08 | 3373.93 | 17.08 | 2.54E-07 | 9 |
| M_2_F_lqp_Set2 | 0.00 | 0.08 | 3374.33 | 17.48 | 2.82E-07 | 11 |
| M_2_F_lp_Set1 | 0.00 | 0.08 | 3374.65 | 17.81 | 2.29E-07 | 14 |
| M_4_F_h_Set2 | 0.00 | 0.08 | 3374.77 | 17.92 | 3.03E-07 | 10 |
| M_1_F_lq_Set2 | 0.00 | 0.08 | 3375.01 | 18.16 | 1.67E-07 | 9 |
| M_0.4_F_qp_Set3 | 0.00 | 0.08 | 3375.06 | 18.21 | 9.32E-08 | 9 |
| M_4_F_th_Set2 | 0.00 | 0.08 | 3375.11 | 18.26 | 2.75E-07 | 12 |
| M_3_F_qh_Set1 | 0.00 | 0.08 | 3375.65 | 18.80 | 1.68E-07 | 18 |
| M_0.9_F_lqh_Set2 | 0.00 | 0.08 | 3376.38 | 19.53 | 7.81E-08 | 26 |
| M_0.5_F_qp_Set2 | 0.00 | 0.08 | 3377.01 | 20.16 | 3.79E-08 | 16 |
| M_3_F_lpt_Set1 | 0.00 | 0.08 | 3377.15 | 20.30 | 8.28E-08 | 16 |
| M_3_F_th_Set2 | 0.00 | 0.08 | 3377.86 | 21.01 | 5.70E-08 | 19 |
| M_4_F_lph_Set3 | 0.00 | 0.08 | 3378.36 | 21.51 | 5.64E-08 | 8 |
| M_2_F_qh_Set2 | 0.00 | 0.08 | 3378.42 | 21.57 | 3.59E-08 | 21 |
| M_0.9_F_qp_Set2 | 0.00 | 0.08 | 3378.48 | 21.63 | 2.65E-08 | 15 |
| M_3_F_lph_Set2 | 0.00 | 0.08 | 3378.84 | 21.99 | 3.59E-08 | 17 |
| M_2_F_lqp_Set1 | 0.00 | 0.08 | 3378.95 | 22.10 | 2.80E-08 | 17 |
| M_0.3_F_lp_Set2 | 0.00 | 0.08 | 3379.10 | 22.25 | 1.16E-08 | 19 |
| M_4_F_lqh_Set2 | 0.00 | 0.08 | 3379.23 | 22.38 | 3.59E-08 | 12 |
| M_4_F_qph_Set2 | 0.00 | 0.08 | 3379.39 | 22.54 | 3.41E-08 | 12 |
| M_0.2_F_lp_Set2 | 0.00 | 0.08 | 3379.74 | 22.89 | 7.95E-09 | 21 |
| M_3_F_lpth_Set3 | 0.00 | 0.08 | 3379.80 | 22.95 | 2.35E-08 | 15 |
| M_5_F_lqh_Set3 | 0.00 | 0.08 | 3379.84 | 22.99 | 3.42E-08 | 5 |
| M_3_F_lp_Set1 | 0.00 | 0.08 | 3379.95 | 23.10 | 1.90E-08 | 13 |
| M_0.8_F_qp_Set2 | 0.00 | 0.08 | 3379.99 | 23.14 | 1.12E-08 | 16 |
| M_2_F_lh_Set2 | 0.00 | 0.08 | 3380.07 | 23.22 | 1.54E-08 | 22 |
| M_4_F_lpt_Set1 | 0.00 | 0.08 | 3380.29 | 23.44 | 2.12E-08 | 11 |
| M_3_F_lqpt_Set3 | 0.00 | 0.08 | 3380.35 | 23.50 | 1.75E-08 | 10 |
| M_1_F_lh_Set2 | 0.00 | 0.08 | 3380.47 | 23.62 | 1.10E-08 | 27 |
| M_0.5_F_lqp_Set2 | 0.00 | 0.08 | 3380.51 | 23.66 | 6.70E-09 | 19 |
| M_4_F_lph_Set2 | 0.00 | 0.08 | 3380.52 | 23.67 | 1.91E-08 | 12 |
| M_0.6_F_qp_Set2 | 0.00 | 0.08 | 3380.67 | 23.82 | 6.62E-09 | 17 |
| M_3_F_lt_Set3 | 0.00 | 0.08 | 3380.74 | 23.89 | 1.29E-08 | 9 |
| M_3_F_lqt_Set3 | 0.00 | 0.08 | 3380.74 | 23.89 | 1.36E-08 | 9 |
| M_4_F_lq_Set1 | 0.00 | 0.08 | 3380.85 | 24.00 | 1.45E-08 | 9 |
| M_0.7_F_qp_Set2 | 0.00 | 0.08 | 3380.89 | 24.04 | 6.48E-09 | 17 |
| M_6_F_lqph_Set1 | 0.00 | 0.08 | 3380.89 | 24.04 | 3.19E-08 | 8 |
| M_3_F_lp_Set2 | 0.00 | 0.08 | 3381.06 | 24.21 | 1.09E-08 | 8 |
| M_5_F_lh_Set1 | 0.00 | 0.08 | 3381.11 | 24.26 | 1.65E-08 | 9 |
| M_3_F_lph_Set3 | 0.00 | 0.08 | 3381.36 | 24.51 | 1.02E-08 | 14 |
| M_0.9_F_lh_Set2 | 0.00 | 0.08 | 3381.43 | 24.58 | 6.05E-09 | 28 |
| M_5_F_lh_Set3 | 0.00 | 0.08 | 3381.47 | 24.62 | 1.39E-08 | 5 |
| M_4_F_lpth_Set2 | 0.00 | 0.08 | 3381.54 | 24.69 | 1.22E-08 | 14 |
| M_4_F_pth_Set2 | 0.00 | 0.08 | 3381.76 | 24.91 | 1.06E-08 | 14 |
| M_5_F_lqth_Set3 | 0.00 | 0.08 | 3381.89 | 25.04 | 1.35E-08 | 6 |
| M_6_F_lh_Set2 | 0.00 | 0.08 | 3381.97 | 25.12 | 1.47E-08 | 4 |
| M_3_F_lpth_Set2 | 0.00 | 0.08 | 3382.09 | 25.24 | 7.45E-09 | 21 |
| M_4_F_lqp_Set2 | 0.00 | 0.08 | 3382.23 | 25.38 | 7.88E-09 | 7 |
| M_4_F_lp_Set1 | 0.00 | 0.08 | 3382.32 | 25.47 | 7.02E-09 | 11 |
| M_1_F_qh_Set3 | 0.00 | 0.08 | 3382.60 | 25.75 | 3.86E-09 | 22 |
| M_2_F_lqpt_Set2 | 0.00 | 0.08 | 3382.86 | 26.01 | 4.17E-09 | 25 |
| M_0.4_F_lp_Set2 | 0.00 | 0.08 | 3382.88 | 26.03 | 1.86E-09 | 21 |
| M_2_F_lq_Set2 | 0.00 | 0.08 | 3382.89 | 26.04 | 3.72E-09 | 9 |
| M_4_F_h_Set1 | 0.00 | 0.08 | 3383.00 | 26.15 | 4.93E-09 | 15 |
| M_5_F_lqh_Set2 | 0.00 | 0.08 | 3383.01 | 26.16 | 6.98E-09 | 9 |
| M_6_F_lqpth_Set1 | 0.00 | 0.08 | 3383.05 | 26.20 | 1.14E-08 | 9 |
| M_5_F_lpt_Set1 | 0.00 | 0.08 | 3383.05 | 26.20 | 6.88E-09 | 9 |
| M_0.4_F_lqp_Set2 | 0.00 | 0.08 | 3383.28 | 26.43 | 1.56E-09 | 21 |
| M_6_F_lpth_Set1 | 0.00 | 0.08 | 3383.65 | 26.80 | 8.28E-09 | 9 |
| M_4_F_lqth_Set2 | 0.00 | 0.08 | 3383.71 | 26.86 | 4.09E-09 | 15 |
| M_0.8_F_p_Set2 | 0.00 | 0.08 | 3383.80 | 26.95 | 1.62E-09 | 13 |
| M_6_F_lph_Set1 | 0.00 | 0.08 | 3383.82 | 26.97 | 6.74E-09 | 9 |
| M_1_F_qth_Set3 | 0.00 | 0.08 | 3383.99 | 27.14 | 2.03E-09 | 29 |
| M_5_F_qh_Set2 | 0.00 | 0.08 | 3384.52 | 27.67 | 3.09E-09 | 8 |
| M_5_F_lqpt_Set1 | 0.00 | 0.08 | 3384.55 | 27.70 | 3.46E-09 | 10 |
| M_1_F_p_Set2 | 0.00 | 0.08 | 3384.65 | 27.80 | 1.33E-09 | 13 |
| M_2_F_q_Set1 | 0.00 | 0.08 | 3384.70 | 27.85 | 1.47E-09 | 9 |
| M_5_F_pth_Set2 | 0.00 | 0.08 | 3384.75 | 27.90 | 3.11E-09 | 9 |
| M_5_F_lpth_Set1 | 0.00 | 0.08 | 3384.91 | 28.06 | 2.99E-09 | 13 |
| M_5_F_lpth_Set2 | 0.00 | 0.08 | 3385.06 | 28.21 | 2.79E-09 | 10 |
| M_6_F_lpth_Set2 | 0.00 | 0.08 | 3385.34 | 28.49 | 3.58E-09 | 6 |
| M_2_F_qp_Set2 | 0.00 | 0.08 | 3385.36 | 28.51 | 1.10E-09 | 12 |
| M_4_F_lph_Set1 | 0.00 | 0.08 | 3385.38 | 28.53 | 1.68E-09 | 17 |
| M_5_F_lqp_Set1 | 0.00 | 0.08 | 3385.54 | 28.69 | 1.93E-09 | 10 |
| M_4_F_ph_Set2 | 0.00 | 0.08 | 3385.71 | 28.86 | 1.36E-09 | 14 |
| M_6_F_qth_Set2 | 0.00 | 0.08 | 3385.94 | 29.09 | 2.44E-09 | 7 |
| M_6_F_qh_Set2 | 0.00 | 0.08 | 3385.96 | 29.11 | 2.08E-09 | 7 |
| M_5_F_ph_Set2 | 0.00 | 0.08 | 3385.99 | 29.14 | 1.51E-09 | 9 |
| M_0.4_F_qp_Set2 | 0.00 | 0.08 | 3386.41 | 29.56 | 3.20E-10 | 20 |
| M_0.9_F_qh_Set2 | 0.00 | 0.08 | 3386.65 | 29.80 | 4.50E-10 | 29 |
| M_5_F_lqth_Set2 | 0.00 | 0.08 | 3386.69 | 29.84 | 1.22E-09 | 11 |
| M_5_F_lph_Set2 | 0.00 | 0.08 | 3386.76 | 29.91 | 1.09E-09 | 10 |
| M_4_F_lqpt_Set2 | 0.00 | 0.08 | 3386.81 | 29.96 | 8.56E-10 | 10 |
| M_0.9_F_p_Set2 | 0.00 | 0.08 | 3386.85 | 30.00 | 3.92E-10 | 14 |
| M_6_F_lqpt_Set1 | 0.00 | 0.08 | 3387.04 | 30.19 | 1.45E-09 | 8 |
| M_4_F_lqh_Set3 | 0.00 | 0.08 | 3387.14 | 30.29 | 6.87E-10 | 12 |
| M_5_F_lph_Set1 | 0.00 | 0.08 | 3387.45 | 30.60 | 7.71E-10 | 14 |
| M_6_F_lph_Set2 | 0.00 | 0.08 | 3387.48 | 30.63 | 1.08E-09 | 7 |
| M_2_F_lph_Set2 | 0.00 | 0.08 | 3387.53 | 30.68 | 3.93E-10 | 25 |
| M_5_F_lqph_Set1 | 0.00 | 0.08 | 3387.54 | 30.69 | 7.85E-10 | 14 |
| M_5_F_lh_Set2 | 0.00 | 0.08 | 3387.93 | 31.08 | 5.47E-10 | 9 |
| M_3_F_lqpt_Set2 | 0.00 | 0.08 | 3388.19 | 31.34 | 3.46E-10 | 17 |
| M_4_F_lqpth_Set2 | 0.00 | 0.08 | 3388.29 | 31.44 | 4.21E-10 | 17 |
| M_5_F_lp_Set1 | 0.00 | 0.08 | 3388.41 | 31.56 | 4.21E-10 | 11 |
| M_0.2_F_p_Set2 | 0.00 | 0.08 | 3388.48 | 31.63 | 9.98E-11 | 18 |
| M_1_F_ph_Set1 | 0.00 | 0.08 | 3388.53 | 31.68 | 2.00E-10 | 34 |
| M_0.3_F_p_Set2 | 0.00 | 0.08 | 3388.60 | 31.75 | 9.93E-11 | 18 |
| M_8_F_lqth_Set3 | 0.00 | 0.08 | 3388.87 | 32.02 | 1.15E-09 | 4 |
| M_6_F_qph_Set3 | 0.00 | 0.08 | 3389.06 | 32.21 | 5.09E-10 | 6 |
| M_0.7_F_p_Set2 | 0.00 | 0.08 | 3389.19 | 32.34 | 9.98E-11 | 16 |
| M_4_F_qt_Set1 | 0.00 | 0.08 | 3389.20 | 32.35 | 2.31E-10 | 10 |
| M_3_F_qpt_Set2 | 0.00 | 0.08 | 3389.36 | 32.51 | 1.88E-10 | 17 |
| M_4_F_q_Set1 | 0.00 | 0.08 | 3389.40 | 32.55 | 1.97E-10 | 6 |
| M_6_F_lpt_Set1 | 0.00 | 0.08 | 3389.79 | 32.94 | 3.35E-10 | 9 |
| M_6_F_lqp_Set1 | 0.00 | 0.08 | 3389.80 | 32.95 | 3.20E-10 | 9 |
| M_3_F_ph_Set2 | 0.00 | 0.08 | 3389.89 | 33.04 | 1.38E-10 | 21 |
| M_6_F_qph_Set2 | 0.00 | 0.08 | 3389.91 | 33.06 | 3.31E-10 | 9 |
| M_1_F_h_Set1 | 0.00 | 0.08 | 3390.21 | 33.36 | 8.31E-11 | 34 |
| M_3_F_qp_Set2 | 0.00 | 0.08 | 3390.31 | 33.46 | 1.09E-10 | 11 |
| M_6_F_lp_Set1 | 0.00 | 0.08 | 3390.37 | 33.52 | 2.14E-10 | 9 |
| M_1_F_ph_Set3 | 0.00 | 0.08 | 3390.58 | 33.73 | 7.20E-11 | 27 |
| M_5_F_qth_Set2 | 0.00 | 0.08 | 3390.63 | 33.78 | 1.63E-10 | 11 |
| M_8_F_lh_Set3 | 0.00 | 0.08 | 3390.98 | 34.13 | 2.57E-10 | 5 |
| M_8_F_lqh_Set3 | 0.00 | 0.08 | 3391.01 | 34.16 | 3.08E-10 | 5 |
| M_4_F_lqph_Set2 | 0.00 | 0.08 | 3391.14 | 34.30 | 9.87E-11 | 17 |
| M_3_F_h_Set2 | 0.00 | 0.08 | 3391.22 | 34.37 | 6.71E-11 | 22 |
| M_8_F_pth_Set2 | 0.00 | 0.08 | 3391.50 | 34.65 | 2.79E-10 | 5 |
| M_0.9_F_lph_Set3 | 0.00 | 0.08 | 3391.63 | 34.78 | 3.85E-11 | 28 |
| M_1_F_qh_Set2 | 0.00 | 0.08 | 3391.67 | 34.83 | 4.12E-11 | 30 |
| M_0.1_F_p_Set2 | 0.00 | 0.08 | 3391.81 | 34.96 | 1.81E-11 | 20 |
| M_0.8_F_h_Set2 | 0.00 | 0.08 | 3392.12 | 35.27 | 2.56E-11 | 31 |
| M_3_F_lt_Set2 | 0.00 | 0.08 | 3392.18 | 35.33 | 4.23E-11 | 17 |
| M_5_F_lqpth_Set1 | 0.00 | 0.08 | 3392.19 | 35.34 | 7.93E-11 | 16 |
| M_10_F_th_Set1 | 0.00 | 0.08 | 3392.63 | 35.78 | 3.55E-10 | 3 |
| M_10_F_th_Set2 | 0.00 | 0.08 | 3392.63 | 35.79 | 3.62E-10 | 3 |
| M_10_F_h_Set1 | 0.00 | 0.08 | 3392.86 | 36.01 | 1.94E-10 | 3 |
| M_10_F_h_Set2 | 0.00 | 0.08 | 3392.87 | 36.02 | 1.97E-10 | 3 |
| M_3_F_pt_Set2 | 0.00 | 0.08 | 3392.87 | 36.02 | 3.09E-11 | 18 |
| M_1_F_lph_Set3 | 0.00 | 0.08 | 3393.38 | 36.53 | 1.83E-11 | 28 |
| M_8_F_ph_Set2 | 0.00 | 0.08 | 3393.70 | 36.85 | 7.26E-11 | 6 |
| M_4_F_lh_Set2 | 0.00 | 0.08 | 3394.27 | 37.42 | 1.82E-11 | 17 |
| M_4_F_lqt_Set2 | 0.00 | 0.08 | 3394.43 | 37.58 | 1.78E-11 | 10 |
| M_8_F_pth_Set3 | 0.00 | 0.08 | 3394.51 | 37.66 | 6.27E-11 | 6 |
| M_0.9_F_h_Set2 | 0.00 | 0.08 | 3394.70 | 37.85 | 7.83E-12 | 31 |
| M_1_F_lph_Set1 | 0.00 | 0.08 | 3395.02 | 38.17 | 8.04E-12 | 36 |
| M_8_F_lpth_Set2 | 0.00 | 0.08 | 3395.35 | 38.50 | 4.59E-11 | 7 |
| M_8_F_lph_Set2 | 0.00 | 0.08 | 3395.37 | 38.52 | 3.64E-11 | 7 |
| M_5_F_lqt_Set2 | 0.00 | 0.08 | 3396.04 | 39.19 | 1.02E-11 | 7 |
| M_4_F_qpt_Set2 | 0.00 | 0.08 | 3396.34 | 39.49 | 7.07E-12 | 13 |
| M_3_F_lq_Set2 | 0.00 | 0.08 | 3396.37 | 39.52 | 5.15E-12 | 9 |
| M_8_F_ph_Set3 | 0.00 | 0.08 | 3396.65 | 39.80 | 1.68E-11 | 7 |
| M_10_F_qph_Set1 | 0.00 | 0.08 | 3397.32 | 40.47 | 6.08E-11 | 6 |
| M_10_F_qp_Set1 | 0.00 | 0.08 | 3397.32 | 40.47 | 2.59E-11 | 6 |
| M_10_F_qpt_Set1 | 0.00 | 0.08 | 3397.33 | 40.48 | 5.44E-11 | 6 |
| M_10_F_lqth_Set3 | 0.00 | 0.08 | 3397.46 | 40.61 | 1.55E-10 | 4 |
| M_0.9_F_h_Set3 | 0.00 | 0.08 | 3397.94 | 41.09 | 1.55E-12 | 28 |
| M_0.1_F_l_Set1 | 0.00 | 0.08 | 3398.32 | 41.47 | 6.95E-13 | 11 |
| M_10_F_pth_Set2 | 0.00 | 0.08 | 3398.43 | 41.58 | 4.71E-11 | 4 |
| M_4_F_lqp_Set3 | 0.00 | 0.08 | 3398.68 | 41.83 | 2.11E-12 | 5 |
| M_1_F_qph_Set1 | 0.00 | 0.08 | 3399.18 | 42.33 | 1.01E-12 | 37 |
| M_10_F_lqh_Set3 | 0.00 | 0.08 | 3399.61 | 42.76 | 1.41E-11 | 5 |
| M_10_F_lh_Set3 | 0.00 | 0.08 | 3399.62 | 42.77 | 8.08E-12 | 5 |
| M_10_F_lqpth_Set3 | 0.00 | 0.08 | 3399.80 | 42.95 | 1.23E-10 | 5 |
| M_10_F_lpth_Set3 | 0.00 | 0.08 | 3399.81 | 42.96 | 6.87E-11 | 5 |
| M_10_F_lqph_Set3 | 0.00 | 0.08 | 3399.82 | 42.97 | 3.64E-11 | 5 |
| M_0.6_F_l_Set1 | 0.00 | 0.08 | 3399.84 | 42.99 | 4.43E-13 | 9 |
| M_0.7_F_l_Set1 | 0.00 | 0.08 | 3400.36 | 43.51 | 3.71E-13 | 9 |
| M_10_F_ph_Set2 | 0.00 | 0.08 | 3400.58 | 43.73 | 6.42E-12 | 5 |
| M_0.8_F_l_Set1 | 0.00 | 0.08 | 3401.02 | 44.18 | 2.93E-13 | 9 |
| M_0.9_F_l_Set1 | 0.00 | 0.08 | 3401.47 | 44.62 | 2.60E-13 | 9 |
| M_10_F_qph_Set2 | 0.00 | 0.08 | 3401.78 | 44.93 | 6.78E-12 | 6 |
| M_10_F_lph_Set2 | 0.00 | 0.08 | 3401.87 | 45.02 | 5.25E-12 | 6 |
| M_10_F_lpth_Set2 | 0.00 | 0.08 | 3401.89 | 45.04 | 2.12E-11 | 6 |
| M_10_F_pth_Set3 | 0.00 | 0.08 | 3401.92 | 45.07 | 8.67E-12 | 5 |
| M_10_F_lph_Set3 | 0.00 | 0.08 | 3402.04 | 45.19 | 4.98E-12 | 6 |
| M_0.4_F_l_Set1 | 0.00 | 0.08 | 3402.13 | 45.28 | 1.21E-13 | 11 |
| M_1_F_l_Set1 | 0.00 | 0.08 | 3402.25 | 45.40 | 1.98E-13 | 9 |
| M_0.5_F_l_Set1 | 0.00 | 0.08 | 3403.76 | 46.91 | 5.75E-14 | 11 |
| M_0.8_F_h_Set3 | 0.00 | 0.08 | 3403.89 | 47.04 | 7.10E-14 | 31 |
| M_10_F_ph_Set3 | 0.00 | 0.08 | 3404.05 | 47.20 | 1.15E-12 | 6 |
| M_10_F_lqph_Set2 | 0.00 | 0.08 | 3404.82 | 47.97 | 2.77E-12 | 7 |
| M_3_F_p_Set2 | 0.00 | 0.08 | 3405.04 | 48.19 | 6.62E-14 | 10 |
| M_0.3_F_q_Set2 | 0.00 | 0.08 | 3406.24 | 49.39 | 1.46E-14 | 7 |
| M_0.4_F_q_Set2 | 0.00 | 0.08 | 3406.37 | 49.52 | 1.45E-14 | 7 |
| M_0.5_F_q_Set2 | 0.00 | 0.08 | 3406.60 | 49.75 | 1.39E-14 | 7 |
| M_0.6_F_q_Set2 | 0.00 | 0.08 | 3406.76 | 49.91 | 1.39E-14 | 7 |
| M_0.7_F_q_Set2 | 0.00 | 0.08 | 3407.01 | 50.16 | 1.34E-14 | 7 |
| M_10_F_lqpth_Set2 | 0.00 | 0.08 | 3407.04 | 50.19 | 2.61E-12 | 8 |
| M_4_F_qt_Set2 | 0.00 | 0.08 | 3407.09 | 50.24 | 3.03E-14 | 14 |
| M_4_F_lq_Set2 | 0.00 | 0.08 | 3407.72 | 50.87 | 2.13E-14 | 8 |
| M_1_F_lqph_Set1 | 0.00 | 0.08 | 3408.23 | 51.38 | 1.12E-14 | 40 |
| M_4_F_lpt_Set2 | 0.00 | 0.08 | 3408.35 | 51.50 | 1.71E-14 | 19 |
| M_2_F_l_Set1 | 0.00 | 0.08 | 3408.50 | 51.65 | 9.95E-15 | 8 |
| M_0.7_F_h_Set2 | 0.00 | 0.08 | 3410.09 | 53.24 | 2.90E-15 | 38 |
| M_0.7_F_lph_Set2 | 0.00 | 0.08 | 3410.35 | 53.50 | 2.68E-15 | 40 |
| M_5_F_lt_Set3 | 0.00 | 0.08 | 3410.66 | 53.81 | 6.30E-15 | 9 |
| M_5_F_lqt_Set3 | 0.00 | 0.08 | 3410.69 | 53.84 | 6.76E-15 | 9 |
| M_2_F_q_Set2 | 0.00 | 0.08 | 3411.14 | 54.29 | 2.67E-15 | 6 |
| M_5_F_lq_Set2 | 0.00 | 0.08 | 3411.40 | 54.55 | 4.26E-15 | 7 |
| M_5_F_lqp_Set3 | 0.00 | 0.08 | 3411.72 | 54.88 | 3.99E-15 | 5 |
| M_4_F_p_Set2 | 0.00 | 0.08 | 3413.69 | 56.84 | 1.06E-15 | 9 |
| M_4_F_lq_Set3 | 0.00 | 0.08 | 3413.94 | 57.09 | 9.50E-16 | 5 |
| M_5_F_pt_Set2 | 0.00 | 0.08 | 3414.60 | 57.75 | 9.20E-16 | 15 |
| M_0.5_F_qph_Set2 | 0.00 | 0.08 | 3414.98 | 58.13 | 2.24E-16 | 44 |
| M_6_F_lqt_Set3 | 0.00 | 0.08 | 3415.37 | 58.52 | 9.16E-16 | 6 |
| M_6_F_lt_Set3 | 0.00 | 0.08 | 3415.37 | 58.52 | 8.13E-16 | 6 |
| M_5_F_lt_Set2 | 0.00 | 0.08 | 3415.83 | 58.98 | 4.73E-16 | 13 |
| M_0.9_F_lph_Set1 | 0.00 | 0.08 | 3415.88 | 59.03 | 2.09E-16 | 43 |
| M_10_F_lqpt_Set2 | 0.00 | 0.08 | 3416.80 | 59.95 | 5.70E-15 | 6 |
| M_10_F_lqp_Set2 | 0.00 | 0.08 | 3416.85 | 60.00 | 2.14E-15 | 6 |
| M_6_F_lpt_Set3 | 0.00 | 0.08 | 3419.58 | 62.73 | 1.15E-16 | 8 |
| M_0.6_F_h_Set2 | 0.00 | 0.08 | 3421.62 | 64.77 | 8.35E-18 | 43 |
| M_6_F_pt_Set2 | 0.00 | 0.08 | 3421.74 | 64.89 | 3.57E-17 | 13 |
| M_4_F_lp_Set3 | 0.00 | 0.08 | 3422.18 | 65.33 | 1.56E-17 | 4 |
| M_5_F_lpt_Set2 | 0.00 | 0.08 | 3422.22 | 65.37 | 2.16E-17 | 17 |
| M_3_F_l_Set1 | 0.00 | 0.08 | 3423.24 | 66.39 | 7.32E-18 | 9 |
| M_0.8_F_lqph_Set1 | 0.00 | 0.08 | 3423.29 | 66.44 | 4.69E-18 | 46 |
| M_5_F_p_Set2 | 0.00 | 0.08 | 3423.82 | 66.97 | 8.35E-18 | 9 |
| M_5_F_lq_Set3 | 0.00 | 0.08 | 3424.55 | 67.70 | 5.96E-18 | 5 |
| M_4_F_l_Set1 | 0.00 | 0.08 | 3424.99 | 68.14 | 3.66E-18 | 5 |
| M_5_F_lp_Set3 | 0.00 | 0.08 | 3425.58 | 68.73 | 3.59E-18 | 4 |
| M_5_F_l_Set1 | 0.00 | 0.08 | 3425.83 | 68.98 | 2.99E-18 | 5 |
| M_0.9_F_lqph_Set1 | 0.00 | 0.08 | 3426.70 | 69.85 | 9.59E-19 | 46 |
| M_6_F_l_Set1 | 0.00 | 0.08 | 3426.81 | 69.96 | 2.44E-18 | 5 |
| M_6_F_lqp_Set3 | 0.00 | 0.08 | 3426.81 | 69.96 | 2.96E-18 | 5 |
| M_8_F_lt_Set3 | 0.00 | 0.08 | 3427.04 | 70.19 | 3.73E-18 | 4 |
| M_8_F_lqt_Set3 | 0.00 | 0.08 | 3427.04 | 70.19 | 4.52E-18 | 4 |
| M_0.7_F_l_Set2 | 0.00 | 0.08 | 3427.33 | 70.48 | 5.17E-19 | 6 |
| M_0.9_F_qph_Set1 | 0.00 | 0.08 | 3427.57 | 70.72 | 6.09E-19 | 46 |
| M_0.8_F_l_Set2 | 0.00 | 0.08 | 3427.65 | 70.80 | 4.85E-19 | 6 |
| M_0.9_F_l_Set2 | 0.00 | 0.08 | 3427.94 | 71.09 | 4.66E-19 | 6 |
| M_1_F_l_Set2 | 0.00 | 0.08 | 3428.30 | 71.45 | 4.38E-19 | 6 |
| M_6_F_lt_Set1 | 0.00 | 0.08 | 3428.56 | 71.71 | 1.10E-18 | 15 |
| M_6_F_p_Set2 | 0.00 | 0.08 | 3429.08 | 72.23 | 8.05E-19 | 7 |
| M_8_F_lqpt_Set3 | 0.00 | 0.08 | 3429.33 | 72.48 | 1.78E-18 | 5 |
| M_8_F_lpt_Set3 | 0.00 | 0.08 | 3429.33 | 72.48 | 1.51E-18 | 5 |
| M_6_F_lp_Set3 | 0.00 | 0.08 | 3429.35 | 72.50 | 7.41E-19 | 4 |
| M_0.4_F_l_Set3 | 0.00 | 0.08 | 3429.55 | 72.70 | 1.35E-19 | 4 |
| M_0.5_F_l_Set3 | 0.00 | 0.08 | 3429.64 | 72.79 | 1.38E-19 | 4 |
| M_0.6_F_l_Set3 | 0.00 | 0.08 | 3429.75 | 72.90 | 1.42E-19 | 4 |
| M_3_F_l_Set3 | 0.00 | 0.08 | 3429.84 | 72.99 | 2.70E-19 | 2 |
| M_0.7_F_l_Set3 | 0.00 | 0.08 | 3429.88 | 73.03 | 1.45E-19 | 4 |
| M_0.8_F_l_Set3 | 0.00 | 0.08 | 3430.01 | 73.16 | 1.49E-19 | 4 |
| M_4_F_l_Set3 | 0.00 | 0.08 | 3430.02 | 73.17 | 2.97E-19 | 2 |
| M_0.9_F_l_Set3 | 0.00 | 0.08 | 3430.17 | 73.32 | 1.53E-19 | 4 |
| M_6_F_lq_Set3 | 0.00 | 0.08 | 3430.21 | 73.36 | 4.77E-19 | 3 |
| M_10_F_qp_Set2 | 0.00 | 0.08 | 3430.22 | 73.37 | 1.89E-18 | 8 |
| M_5_F_l_Set3 | 0.00 | 0.08 | 3430.22 | 73.38 | 3.34E-19 | 2 |
| M_1_F_l_Set3 | 0.00 | 0.08 | 3430.33 | 73.48 | 1.59E-19 | 4 |
| M_6_F_l_Set3 | 0.00 | 0.08 | 3430.47 | 73.62 | 3.95E-19 | 2 |
| M_8_F_lq_Set3 | 0.00 | 0.08 | 3431.04 | 74.19 | 4.85E-19 | 2 |
| M_8_F_l_Set3 | 0.00 | 0.08 | 3431.04 | 74.19 | 4.43E-19 | 2 |
| M_10_F_qpt_Set2 | 0.00 | 0.08 | 3431.34 | 74.50 | 2.31E-18 | 9 |
| M_10_F_l_Set3 | 0.00 | 0.08 | 3431.74 | 74.89 | 6.23E-19 | 2 |
| M_10_F_lq_Set3 | 0.00 | 0.08 | 3431.74 | 74.89 | 7.51E-19 | 2 |
| M_8_F_lqp_Set3 | 0.00 | 0.08 | 3431.98 | 75.13 | 3.73E-19 | 3 |
| M_8_F_lp_Set3 | 0.00 | 0.08 | 3431.98 | 75.13 | 3.09E-19 | 3 |
| M_10_F_lqt_Set3 | 0.00 | 0.08 | 3432.81 | 75.96 | 8.03E-19 | 3 |
| M_10_F_lt_Set3 | 0.00 | 0.08 | 3432.81 | 75.96 | 4.79E-19 | 3 |
| M_2_F_l_Set3 | 0.00 | 0.08 | 3432.88 | 76.03 | 5.07E-20 | 4 |
| M_10_F_lp_Set3 | 0.00 | 0.08 | 3433.71 | 76.86 | 2.93E-19 | 3 |
| M_10_F_lqp_Set3 | 0.00 | 0.08 | 3433.71 | 76.86 | 4.77E-19 | 3 |
| M_10_F_lpt_Set3 | 0.00 | 0.08 | 3434.58 | 77.73 | 3.91E-19 | 4 |
| M_10_F_lqpt_Set3 | 0.00 | 0.08 | 3434.58 | 77.73 | 8.35E-19 | 4 |
| M_10_F_lpt_Set2 | 0.00 | 0.08 | 3439.12 | 82.27 | 3.92E-20 | 7 |
| M_8_F_pt_Set2 | 0.00 | 0.08 | 3440.08 | 83.23 | 6.03E-21 | 11 |
| M_8_F_p_Set2 | 0.00 | 0.08 | 3440.81 | 83.96 | 3.46E-21 | 6 |
| M_10_F_pt_Set2 | 0.00 | 0.08 | 3443.46 | 86.61 | 2.95E-21 | 7 |
| M_0.2_F_qp_Set1 | 0.00 | 0.08 | 3444.36 | 87.51 | 7.41E-23 | 44 |
| M_10_F_p_Set2 | 0.00 | 0.08 | 3446.85 | 90.00 | 3.45E-22 | 4 |
| M_0.4_F_ph_Set2 | 0.00 | 0.08 | 3453.65 | 96.80 | 8.13E-25 | 55 |
| M_0.5_F_lqph_Set2 | 0.00 | 0.08 | 3458.12 | 101.27 | 9.65E-26 | 55 |
| M_0.1_F_p_Set3 | 0.00 | 0.08 | 3463.21 | 106.36 | 5.67E-27 | 6 |
| M_0.2_F_lp_Set1 | 0.00 | 0.08 | 3463.26 | 106.41 | 5.81E-27 | 51 |
| M_0.2_F_p_Set3 | 0.00 | 0.08 | 3463.29 | 106.44 | 5.68E-27 | 6 |
| M_0.3_F_p_Set3 | 0.00 | 0.08 | 3463.44 | 106.59 | 5.57E-27 | 6 |
| M_0.4_F_p_Set3 | 0.00 | 0.08 | 3463.57 | 106.72 | 5.53E-27 | 6 |
| M_0.5_F_p_Set3 | 0.00 | 0.08 | 3463.76 | 106.91 | 5.42E-27 | 6 |
| M_2_F_p_Set3 | 0.00 | 0.08 | 3465.74 | 108.89 | 3.75E-27 | 5 |
| M_3_F_p_Set3 | 0.00 | 0.08 | 3468.82 | 111.97 | 9.39E-28 | 5 |
| M_0.6_F_lq_Set1 | 0.00 | 0.09 | 3359.61 | 2.76 | 0.000244 | 16 |
| M_0.6_F_lp_Set2 | 0.00 | 0.09 | 3361.73 | 4.88 | 8.49E-05 | 11 |
| M_2_F_qph_Set1 | 0.00 | 0.09 | 3370.13 | 13.28 | 2.38E-06 | 21 |
| M_1_F_qph_Set3 | 0.00 | 0.09 | 3371.42 | 14.57 | 1.08E-06 | 20 |
| M_2_F_lqph_Set2 | 0.00 | 0.09 | 3372.53 | 15.68 | 7.35E-07 | 20 |
| M_0.2_F_qp_Set3 | 0.00 | 0.09 | 3372.72 | 15.87 | 2.68E-07 | 9 |
| M_3_F_lqth_Set2 | 0.00 | 0.09 | 3372.81 | 15.96 | 7.67E-07 | 17 |
| M_0.1_F_qp_Set3 | 0.00 | 0.09 | 3372.89 | 16.04 | 2.35E-07 | 10 |
| M_3_F_qth_Set2 | 0.00 | 0.09 | 3373.04 | 16.19 | 6.66E-07 | 17 |
| M_2_F_h_Set1 | 0.00 | 0.09 | 3373.77 | 16.92 | 3.54E-07 | 22 |
| M_1_F_qp_Set1 | 0.00 | 0.09 | 3374.14 | 17.29 | 2.62E-07 | 16 |
| M_0.3_F_qp_Set3 | 0.00 | 0.09 | 3374.54 | 17.69 | 1.14E-07 | 10 |
| M_0.5_F_qp_Set3 | 0.00 | 0.09 | 3375.03 | 18.18 | 1.02E-07 | 9 |
| M_3_F_qp_Set1 | 0.00 | 0.09 | 3377.06 | 20.21 | 8.23E-08 | 12 |
| M_3_F_lqh_Set1 | 0.00 | 0.09 | 3377.08 | 20.23 | 8.53E-08 | 19 |
| M_5_F_qth_Set1 | 0.00 | 0.09 | 3377.29 | 20.44 | 1.28E-07 | 9 |
| M_3_F_th_Set1 | 0.00 | 0.09 | 3377.54 | 20.69 | 6.66E-08 | 21 |
| M_2_F_lqph_Set1 | 0.00 | 0.09 | 3377.64 | 20.79 | 5.71E-08 | 24 |
| M_3_F_qpt_Set1 | 0.00 | 0.09 | 3377.79 | 20.95 | 6.09E-08 | 16 |
| M_2_F_qph_Set2 | 0.00 | 0.09 | 3377.99 | 21.14 | 4.68E-08 | 22 |
| M_0.6_F_qp_Set3 | 0.00 | 0.09 | 3378.48 | 21.63 | 1.97E-08 | 9 |
| M_3_F_lqh_Set2 | 0.00 | 0.09 | 3378.88 | 22.03 | 3.48E-08 | 17 |
| M_0.8_F_lh_Set2 | 0.00 | 0.09 | 3379.20 | 22.35 | 1.66E-08 | 28 |
| M_3_F_lh_Set3 | 0.00 | 0.09 | 3379.28 | 22.43 | 2.70E-08 | 13 |
| M_1_F_lqph_Set3 | 0.00 | 0.09 | 3379.45 | 22.60 | 1.99E-08 | 23 |
| M_6_F_lh_Set1 | 0.00 | 0.09 | 3379.49 | 22.64 | 5.06E-08 | 6 |
| M_1_F_qp_Set2 | 0.00 | 0.09 | 3379.57 | 22.72 | 1.74E-08 | 15 |
| M_5_F_qh_Set1 | 0.00 | 0.09 | 3379.70 | 22.85 | 3.44E-08 | 10 |
| M_3_F_ph_Set1 | 0.00 | 0.09 | 3379.92 | 23.07 | 2.01E-08 | 19 |
| M_4_F_qpt_Set1 | 0.00 | 0.09 | 3380.06 | 23.21 | 2.42E-08 | 11 |
| M_4_F_qph_Set1 | 0.00 | 0.09 | 3380.69 | 23.84 | 1.78E-08 | 15 |
| M_3_F_lqp_Set1 | 0.00 | 0.09 | 3380.76 | 23.91 | 1.34E-08 | 14 |
| M_6_F_qph_Set1 | 0.00 | 0.09 | 3380.81 | 23.96 | 3.12E-08 | 8 |
| M_5_F_lqh_Set1 | 0.00 | 0.09 | 3381.22 | 24.37 | 1.70E-08 | 11 |
| M_3_F_lqth_Set3 | 0.00 | 0.09 | 3381.23 | 24.38 | 1.14E-08 | 16 |
| M_1_F_lqh_Set3 | 0.00 | 0.09 | 3381.52 | 24.67 | 6.82E-09 | 22 |
| M_0.8_F_p_Set1 | 0.00 | 0.09 | 3381.57 | 24.72 | 4.96E-09 | 19 |
| M_3_F_pt_Set1 | 0.00 | 0.09 | 3381.69 | 24.84 | 8.25E-09 | 18 |
| M_4_F_lh_Set1 | 0.00 | 0.09 | 3381.72 | 24.87 | 9.59E-09 | 13 |
| M_4_F_lqpt_Set1 | 0.00 | 0.09 | 3381.86 | 25.01 | 1.01E-08 | 12 |
| M_0.6_F_p_Set2 | 0.00 | 0.09 | 3381.90 | 25.05 | 3.51E-09 | 14 |
| M_4_F_lqph_Set1 | 0.00 | 0.09 | 3382.06 | 25.21 | 9.23E-09 | 16 |
| M_3_F_qph_Set1 | 0.00 | 0.09 | 3382.14 | 25.29 | 6.98E-09 | 21 |
| M_4_F_lpth_Set1 | 0.00 | 0.09 | 3382.31 | 25.46 | 8.29E-09 | 16 |
| M_3_F_lqh_Set3 | 0.00 | 0.09 | 3382.34 | 25.50 | 6.17E-09 | 15 |
| M_0.4_F_p_Set2 | 0.00 | 0.09 | 3382.59 | 25.74 | 2.13E-09 | 15 |
| M_3_F_lqt_Set1 | 0.00 | 0.09 | 3382.74 | 25.89 | 5.01E-09 | 18 |
| M_4_F_lqh_Set1 | 0.00 | 0.09 | 3382.83 | 25.98 | 5.89E-09 | 16 |
| M_6_F_qth_Set1 | 0.00 | 0.09 | 3382.91 | 26.06 | 1.11E-08 | 9 |
| M_3_F_pth_Set2 | 0.00 | 0.09 | 3382.93 | 26.08 | 4.78E-09 | 21 |
| M_4_F_lqt_Set1 | 0.00 | 0.09 | 3383.09 | 26.24 | 5.13E-09 | 11 |
| M_6_F_qh_Set1 | 0.00 | 0.09 | 3383.16 | 26.31 | 8.39E-09 | 9 |
| M_0.7_F_lqp_Set1 | 0.00 | 0.09 | 3383.23 | 26.38 | 2.05E-09 | 22 |
| M_0.8_F_qh_Set2 | 0.00 | 0.09 | 3383.48 | 26.63 | 1.97E-09 | 29 |
| M_6_F_lqth_Set1 | 0.00 | 0.09 | 3383.53 | 26.68 | 8.67E-09 | 9 |
| M_4_F_qp_Set1 | 0.00 | 0.09 | 3383.59 | 26.74 | 3.81E-09 | 12 |
| M_5_F_lqth_Set1 | 0.00 | 0.09 | 3383.63 | 26.78 | 5.61E-09 | 12 |
| M_3_F_lqpt_Set1 | 0.00 | 0.09 | 3383.76 | 26.91 | 3.17E-09 | 19 |
| M_2_F_qpt_Set2 | 0.00 | 0.09 | 3383.78 | 26.93 | 2.58E-09 | 25 |
| M_3_F_qt_Set1 | 0.00 | 0.09 | 3384.12 | 27.27 | 2.42E-09 | 17 |
| M_1_F_th_Set3 | 0.00 | 0.09 | 3384.35 | 27.50 | 1.63E-09 | 29 |
| M_3_F_qh_Set2 | 0.00 | 0.09 | 3384.40 | 27.55 | 2.13E-09 | 19 |
| M_0.3_F_qp_Set2 | 0.00 | 0.09 | 3384.71 | 27.86 | 7.06E-10 | 20 |
| M_5_F_p_Set1 | 0.00 | 0.09 | 3384.88 | 28.03 | 2.38E-09 | 10 |
| M_1_F_lh_Set3 | 0.00 | 0.09 | 3384.94 | 28.09 | 1.18E-09 | 23 |
| M_6_F_qp_Set1 | 0.00 | 0.09 | 3385.46 | 28.61 | 2.59E-09 | 7 |
| M_4_F_lqp_Set1 | 0.00 | 0.09 | 3385.51 | 28.66 | 1.53E-09 | 13 |
| M_1_F_lqp_Set1 | 0.00 | 0.09 | 3385.52 | 28.67 | 9.12E-10 | 22 |
| M_6_F_pth_Set1 | 0.00 | 0.09 | 3385.63 | 28.78 | 2.88E-09 | 11 |
| M_5_F_qp_Set1 | 0.00 | 0.09 | 3385.68 | 28.83 | 1.70E-09 | 10 |
| M_0.9_F_qp_Set1 | 0.00 | 0.09 | 3385.68 | 28.83 | 7.23E-10 | 21 |
| M_6_F_ph_Set1 | 0.00 | 0.09 | 3385.71 | 28.86 | 2.41E-09 | 11 |
| M_6_F_lqh_Set1 | 0.00 | 0.09 | 3385.76 | 28.91 | 2.48E-09 | 10 |
| M_5_F_pt_Set1 | 0.00 | 0.09 | 3385.93 | 29.08 | 1.54E-09 | 11 |
| M_8_F_lh_Set1 | 0.00 | 0.09 | 3386.07 | 29.22 | 2.95E-09 | 6 |
| M_3_F_lqth_Set1 | 0.00 | 0.09 | 3386.08 | 29.23 | 1.01E-09 | 25 |
| M_2_F_lph_Set1 | 0.00 | 0.09 | 3386.64 | 29.79 | 6.12E-10 | 27 |
| M_4_F_lqpth_Set1 | 0.00 | 0.09 | 3386.65 | 29.80 | 9.57E-10 | 18 |
| M_5_F_qpt_Set1 | 0.00 | 0.09 | 3386.90 | 30.05 | 1.03E-09 | 11 |
| M_6_F_qpt_Set1 | 0.00 | 0.09 | 3386.98 | 30.13 | 1.41E-09 | 8 |
| M_5_F_qph_Set1 | 0.00 | 0.09 | 3387.24 | 30.39 | 8.75E-10 | 14 |
| M_3_F_lh_Set2 | 0.00 | 0.09 | 3387.67 | 30.82 | 4.07E-10 | 20 |
| M_0.8_F_qp_Set1 | 0.00 | 0.09 | 3387.69 | 30.84 | 2.38E-10 | 22 |
| M_5_F_ph_Set1 | 0.00 | 0.09 | 3387.73 | 30.88 | 6.31E-10 | 15 |
| M_6_F_lqph_Set2 | 0.00 | 0.09 | 3387.80 | 30.95 | 1.01E-09 | 8 |
| M_1_F_lqpth_Set3 | 0.00 | 0.09 | 3387.91 | 31.06 | 2.93E-10 | 32 |
| M_0.5_F_p_Set2 | 0.00 | 0.09 | 3388.51 | 31.66 | 1.19E-10 | 17 |
| M_4_F_lqth_Set1 | 0.00 | 0.09 | 3388.51 | 31.66 | 3.70E-10 | 19 |
| M_3_F_lh_Set1 | 0.00 | 0.09 | 3388.55 | 31.70 | 2.61E-10 | 22 |
| M_0.9_F_th_Set3 | 0.00 | 0.09 | 3388.83 | 31.98 | 1.53E-10 | 33 |
| M_5_F_lq_Set1 | 0.00 | 0.09 | 3388.87 | 32.02 | 3.32E-10 | 8 |
| M_0.8_F_lp_Set1 | 0.00 | 0.09 | 3389.59 | 32.74 | 9.10E-11 | 23 |
| M_3_F_lqt_Set2 | 0.00 | 0.09 | 3389.79 | 32.94 | 1.47E-10 | 16 |
| M_3_F_lqph_Set1 | 0.00 | 0.09 | 3390.02 | 33.17 | 1.39E-10 | 24 |
| M_5_F_qt_Set1 | 0.00 | 0.09 | 3390.05 | 33.20 | 1.93E-10 | 8 |
| M_5_F_pth_Set1 | 0.00 | 0.09 | 3390.07 | 33.22 | 2.16E-10 | 16 |
| M_0.6_F_p_Set1 | 0.00 | 0.09 | 3390.55 | 33.70 | 4.64E-11 | 23 |
| M_0.3_F_lqp_Set2 | 0.00 | 0.09 | 3390.63 | 33.78 | 3.69E-11 | 24 |
| M_0.7_F_lp_Set1 | 0.00 | 0.09 | 3390.90 | 34.05 | 4.30E-11 | 24 |
| M_0.1_F_qp_Set2 | 0.00 | 0.09 | 3390.94 | 34.10 | 2.82E-11 | 23 |
| M_0.9_F_qh_Set1 | 0.00 | 0.09 | 3390.96 | 34.11 | 5.20E-11 | 35 |
| M_5_F_lqt_Set1 | 0.00 | 0.09 | 3391.04 | 34.19 | 1.25E-10 | 10 |
| M_4_F_lp_Set2 | 0.00 | 0.09 | 3391.10 | 34.25 | 8.73E-11 | 8 |
| M_8_F_qpt_Set1 | 0.00 | 0.09 | 3391.92 | 35.07 | 2.07E-10 | 6 |
| M_0.6_F_qp_Set1 | 0.00 | 0.09 | 3391.95 | 35.10 | 2.35E-11 | 24 |
| M_8_F_qp_Set1 | 0.00 | 0.09 | 3392.11 | 35.26 | 1.47E-10 | 6 |
| M_5_F_q_Set1 | 0.00 | 0.09 | 3392.19 | 35.34 | 6.09E-11 | 5 |
| M_0.9_F_pth_Set3 | 0.00 | 0.09 | 3392.25 | 35.40 | 2.88E-11 | 36 |
| M_6_F_pt_Set1 | 0.00 | 0.09 | 3392.43 | 35.58 | 8.26E-11 | 11 |
| M_8_F_lpth_Set1 | 0.00 | 0.09 | 3392.53 | 35.68 | 1.86E-10 | 8 |
| M_0.7_F_qp_Set1 | 0.00 | 0.09 | 3392.57 | 35.72 | 1.88E-11 | 24 |
| M_8_F_lph_Set1 | 0.00 | 0.09 | 3392.69 | 35.84 | 1.38E-10 | 8 |
| M_0.6_F_h_Set3 | 0.00 | 0.09 | 3392.83 | 35.98 | 1.49E-11 | 30 |
| M_5_F_lqp_Set2 | 0.00 | 0.09 | 3392.91 | 36.06 | 4.84E-11 | 8 |
| M_6_F_p_Set1 | 0.00 | 0.09 | 3393.05 | 36.20 | 5.36E-11 | 11 |
| M_8_F_lqpth_Set1 | 0.00 | 0.09 | 3393.15 | 36.30 | 1.41E-10 | 8 |
| M_6_F_lq_Set1 | 0.00 | 0.09 | 3393.20 | 36.35 | 5.13E-11 | 7 |
| M_8_F_qph_Set1 | 0.00 | 0.09 | 3393.21 | 36.36 | 1.12E-10 | 8 |
| M_6_F_q_Set1 | 0.00 | 0.09 | 3393.28 | 36.43 | 4.71E-11 | 5 |
| M_8_F_lp_Set1 | 0.00 | 0.09 | 3393.64 | 36.79 | 6.47E-11 | 7 |
| M_8_F_lpt_Set1 | 0.00 | 0.09 | 3393.75 | 36.90 | 7.91E-11 | 7 |
| M_8_F_lqp_Set1 | 0.00 | 0.09 | 3393.88 | 37.03 | 6.90E-11 | 7 |
| M_8_F_lqpt_Set1 | 0.00 | 0.09 | 3393.95 | 37.10 | 8.39E-11 | 7 |
| M_6_F_qt_Set1 | 0.00 | 0.09 | 3394.06 | 37.21 | 3.56E-11 | 8 |
| M_0.9_F_lqth_Set3 | 0.00 | 0.09 | 3394.09 | 37.24 | 1.16E-11 | 35 |
| M_8_F_lqph_Set2 | 0.00 | 0.09 | 3394.24 | 37.39 | 7.54E-11 | 7 |
| M_6_F_lqt_Set1 | 0.00 | 0.09 | 3394.24 | 37.39 | 3.51E-11 | 8 |
| M_3_F_pth_Set1 | 0.00 | 0.09 | 3394.38 | 37.53 | 1.55E-11 | 27 |
| M_8_F_lh_Set2 | 0.00 | 0.09 | 3394.44 | 37.59 | 4.52E-11 | 7 |
| M_0.8_F_lqh_Set2 | 0.00 | 0.09 | 3394.65 | 37.80 | 7.55E-12 | 33 |
| M_8_F_qth_Set1 | 0.00 | 0.09 | 3395.03 | 38.18 | 4.63E-11 | 9 |
| M_8_F_lqph_Set1 | 0.00 | 0.09 | 3395.05 | 38.20 | 4.97E-11 | 9 |
| M_0.2_F_qp_Set2 | 0.00 | 0.09 | 3395.16 | 38.31 | 3.59E-12 | 24 |
| M_5_F_lqpt_Set2 | 0.00 | 0.09 | 3395.21 | 38.36 | 1.68E-11 | 10 |
| M_8_F_lq_Set1 | 0.00 | 0.09 | 3395.28 | 38.43 | 2.80E-11 | 5 |
| M_8_F_qph_Set2 | 0.00 | 0.09 | 3395.33 | 38.48 | 3.90E-11 | 7 |
| M_3_F_lpth_Set1 | 0.00 | 0.09 | 3395.37 | 38.52 | 9.73E-12 | 28 |
| M_0.9_F_qth_Set3 | 0.00 | 0.09 | 3395.55 | 38.70 | 5.49E-12 | 35 |
| M_8_F_q_Set1 | 0.00 | 0.09 | 3395.97 | 39.12 | 1.84E-11 | 5 |
| M_8_F_lqh_Set1 | 0.00 | 0.09 | 3396.26 | 39.41 | 2.20E-11 | 9 |
| M_8_F_qt_Set1 | 0.00 | 0.09 | 3396.30 | 39.45 | 1.84E-11 | 6 |
| M_8_F_lqpth_Set2 | 0.00 | 0.09 | 3396.42 | 39.57 | 2.79E-11 | 8 |
| M_8_F_lqth_Set1 | 0.00 | 0.09 | 3396.42 | 39.57 | 2.59E-11 | 9 |
| M_0.2_F_l_Set1 | 0.00 | 0.09 | 3396.75 | 39.90 | 1.59E-12 | 10 |
| M_3_F_lph_Set1 | 0.00 | 0.09 | 3396.91 | 40.06 | 4.27E-12 | 26 |
| M_8_F_lqt_Set1 | 0.00 | 0.09 | 3397.05 | 40.20 | 1.44E-11 | 6 |
| M_10_F_lqpt_Set1 | 0.00 | 0.09 | 3397.30 | 40.45 | 9.25E-11 | 6 |
| M_10_F_lqpth_Set1 | 0.00 | 0.09 | 3397.31 | 40.46 | 2.81E-10 | 6 |
| M_10_F_lqp_Set1 | 0.00 | 0.09 | 3397.31 | 40.46 | 3.65E-11 | 6 |
| M_8_F_qh_Set1 | 0.00 | 0.09 | 3397.34 | 40.49 | 1.12E-11 | 10 |
| M_10_F_lqph_Set1 | 0.00 | 0.09 | 3397.39 | 40.54 | 1.06E-10 | 6 |
| M_0.8_F_lh_Set3 | 0.00 | 0.09 | 3398.00 | 41.15 | 1.37E-12 | 30 |
| M_0.2_F_lqp_Set2 | 0.00 | 0.09 | 3398.40 | 41.55 | 7.19E-13 | 27 |
| M_10_F_lqt_Set1 | 0.00 | 0.09 | 3398.66 | 41.81 | 1.99E-11 | 5 |
| M_10_F_lq_Set1 | 0.00 | 0.09 | 3398.66 | 41.81 | 1.11E-11 | 5 |
| M_4_F_qp_Set2 | 0.00 | 0.09 | 3398.80 | 41.95 | 1.90E-12 | 11 |
| M_0.6_F_lp_Set1 | 0.00 | 0.09 | 3398.82 | 41.97 | 7.51E-13 | 27 |
| M_10_F_qt_Set1 | 0.00 | 0.09 | 3399.29 | 42.44 | 1.02E-11 | 5 |
| M_10_F_q_Set1 | 0.00 | 0.09 | 3399.29 | 42.44 | 7.00E-12 | 5 |
| M_0.6_F_lh_Set2 | 0.00 | 0.09 | 3399.56 | 42.71 | 5.21E-13 | 37 |
| M_10_F_lqh_Set1 | 0.00 | 0.09 | 3399.64 | 42.79 | 1.31E-11 | 6 |
| M_10_F_lqth_Set1 | 0.00 | 0.09 | 3399.65 | 42.80 | 4.31E-11 | 6 |
| M_10_F_qh_Set1 | 0.00 | 0.09 | 3400.28 | 43.43 | 6.54E-12 | 8 |
| M_0.8_F_lph_Set3 | 0.00 | 0.09 | 3400.35 | 43.50 | 4.40E-13 | 32 |
| M_10_F_qth_Set1 | 0.00 | 0.09 | 3400.39 | 43.54 | 1.47E-11 | 8 |
| M_10_F_lh_Set2 | 0.00 | 0.09 | 3402.14 | 45.29 | 2.25E-12 | 7 |
| M_0.7_F_lh_Set2 | 0.00 | 0.09 | 3402.85 | 46.00 | 1.10E-13 | 37 |
| M_0.8_F_th_Set3 | 0.00 | 0.09 | 3403.03 | 46.18 | 1.13E-13 | 40 |
| M_3_F_lqpth_Set1 | 0.00 | 0.09 | 3403.41 | 46.56 | 1.76E-13 | 31 |
| M_10_F_pth_Set1 | 0.00 | 0.09 | 3404.31 | 47.46 | 2.37E-12 | 7 |
| M_0.5_F_lp_Set1 | 0.00 | 0.09 | 3404.33 | 47.48 | 4.39E-14 | 30 |
| M_10_F_pt_Set1 | 0.00 | 0.09 | 3404.36 | 47.51 | 8.94E-13 | 7 |
| M_10_F_p_Set1 | 0.00 | 0.09 | 3404.37 | 47.52 | 5.73E-13 | 7 |
| M_10_F_ph_Set1 | 0.00 | 0.09 | 3404.57 | 47.72 | 8.54E-13 | 7 |
| M_0.8_F_lh_Set1 | 0.00 | 0.09 | 3405.17 | 48.32 | 3.79E-14 | 41 |
| M_5_F_lp_Set2 | 0.00 | 0.09 | 3405.24 | 48.39 | 9.36E-14 | 9 |
| M_0.4_F_p_Set1 | 0.00 | 0.09 | 3405.28 | 48.43 | 2.51E-14 | 29 |
| M_6_F_qpt_Set2 | 0.00 | 0.09 | 3405.36 | 48.51 | 1.45E-13 | 9 |
| M_6_F_lqp_Set2 | 0.00 | 0.09 | 3406.56 | 49.71 | 7.36E-14 | 9 |
| M_6_F_lqpt_Set2 | 0.00 | 0.09 | 3407.00 | 50.15 | 6.75E-14 | 10 |
| M_6_F_t_Set3 | 0.00 | 0.09 | 3407.30 | 50.45 | 4.38E-14 | 8 |
| M_0.3_F_p_Set1 | 0.00 | 0.09 | 3408.42 | 51.57 | 4.92E-15 | 31 |
| M_5_F_qp_Set2 | 0.00 | 0.09 | 3408.47 | 51.62 | 1.91E-14 | 11 |
| M_0.5_F_p_Set1 | 0.00 | 0.09 | 3409.49 | 52.64 | 3.30E-15 | 30 |
| M_0.6_F_qh_Set2 | 0.00 | 0.09 | 3410.00 | 53.15 | 2.84E-15 | 40 |
| M_0.4_F_lp_Set1 | 0.00 | 0.09 | 3410.01 | 53.16 | 2.38E-15 | 33 |
| M_8_F_lqpt_Set2 | 0.00 | 0.09 | 3410.23 | 53.38 | 2.46E-14 | 6 |
| M_8_F_lqp_Set2 | 0.00 | 0.09 | 3411.67 | 54.82 | 9.55E-15 | 5 |
| M_0.3_F_qp_Set1 | 0.00 | 0.09 | 3411.67 | 54.82 | 9.85E-16 | 32 |
| M_0.4_F_lqp_Set1 | 0.00 | 0.09 | 3411.83 | 54.98 | 9.80E-16 | 34 |
| M_0.7_F_h_Set3 | 0.00 | 0.09 | 3411.90 | 55.05 | 1.17E-15 | 35 |
| M_0.3_F_lqp_Set1 | 0.00 | 0.09 | 3412.24 | 55.39 | 7.51E-16 | 37 |
| M_0.7_F_qh_Set1 | 0.00 | 0.09 | 3412.79 | 55.94 | 7.68E-16 | 44 |
| M_0.9_F_lqh_Set1 | 0.00 | 0.09 | 3412.88 | 56.03 | 9.27E-16 | 42 |
| M_0.5_F_qp_Set1 | 0.00 | 0.09 | 3413.59 | 56.74 | 4.31E-16 | 32 |
| M_6_F_qp_Set2 | 0.00 | 0.09 | 3413.85 | 57.00 | 1.78E-15 | 9 |
| M_0.6_F_lqh_Set2 | 0.00 | 0.09 | 3415.71 | 58.86 | 1.66E-16 | 42 |
| M_0.7_F_lqh_Set2 | 0.00 | 0.09 | 3416.07 | 59.22 | 1.53E-16 | 41 |
| M_0.6_F_lqp_Set1 | 0.00 | 0.09 | 3416.54 | 59.69 | 1.09E-16 | 34 |
| M_0.1_F_lqp_Set2 | 0.00 | 0.09 | 3416.85 | 60.00 | 6.75E-17 | 34 |
| M_0.8_F_qh_Set1 | 0.00 | 0.09 | 3417.81 | 60.96 | 6.89E-17 | 44 |
| M_6_F_pt_Set3 | 0.00 | 0.09 | 3418.79 | 61.94 | 1.57E-16 | 8 |
| M_0.5_F_ph_Set3 | 0.00 | 0.09 | 3418.92 | 62.07 | 3.05E-17 | 41 |
| M_6_F_lp_Set2 | 0.00 | 0.09 | 3420.34 | 63.49 | 6.69E-17 | 9 |
| M_0.5_F_lqp_Set1 | 0.00 | 0.09 | 3420.47 | 63.62 | 1.41E-17 | 36 |
| M_0.8_F_lqh_Set1 | 0.00 | 0.09 | 3420.56 | 63.71 | 1.78E-17 | 45 |
| M_0.3_F_lp_Set1 | 0.00 | 0.09 | 3421.02 | 64.17 | 9.14E-18 | 37 |
| M_5_F_lt_Set1 | 0.00 | 0.09 | 3421.44 | 64.59 | 2.85E-17 | 18 |
| M_0.7_F_qh_Set2 | 0.00 | 0.09 | 3421.45 | 64.60 | 1.01E-17 | 42 |
| M_8_F_qpt_Set2 | 0.00 | 0.09 | 3421.81 | 64.96 | 6.76E-17 | 10 |
| M_6_F_lt_Set2 | 0.00 | 0.09 | 3422.06 | 65.21 | 2.86E-17 | 10 |
| M_8_F_qp_Set2 | 0.00 | 0.09 | 3423.97 | 67.12 | 1.79E-17 | 8 |
| M_8_F_t_Set3 | 0.00 | 0.09 | 3425.15 | 68.30 | 8.90E-18 | 6 |
| M_8_F_lp_Set2 | 0.00 | 0.09 | 3425.35 | 68.50 | 8.44E-18 | 5 |
| M_0.4_F_qp_Set1 | 0.00 | 0.09 | 3425.54 | 68.69 | 1.02E-18 | 37 |
| M_6_F_lpt_Set2 | 0.00 | 0.09 | 3426.25 | 69.40 | 4.08E-18 | 13 |
| M_0.7_F_lqh_Set1 | 0.00 | 0.09 | 3427.01 | 70.16 | 6.42E-19 | 48 |
| M_8_F_lt_Set2 | 0.00 | 0.09 | 3430.26 | 73.41 | 7.39E-19 | 6 |
| M_8_F_lpt_Set2 | 0.00 | 0.09 | 3430.83 | 73.98 | 7.06E-19 | 8 |
| M_3_F_l_Set2 | 0.00 | 0.09 | 3433.17 | 76.32 | 5.11E-20 | 4 |
| M_8_F_l_Set2 | 0.00 | 0.09 | 3433.28 | 76.43 | 1.44E-19 | 3 |
| M_2_F_l_Set2 | 0.00 | 0.09 | 3433.34 | 76.49 | 4.03E-20 | 6 |
| M_4_F_l_Set2 | 0.00 | 0.09 | 3433.51 | 76.66 | 5.16E-20 | 4 |
| M_10_F_t_Set2 | 0.00 | 0.09 | 3433.88 | 77.03 | 2.35E-19 | 6 |
| M_5_F_l_Set2 | 0.00 | 0.09 | 3433.91 | 77.06 | 5.27E-20 | 4 |
| M_10_F_l_Set2 | 0.00 | 0.09 | 3434.04 | 77.19 | 1.95E-19 | 3 |
| M_6_F_l_Set2 | 0.00 | 0.09 | 3434.37 | 77.52 | 5.58E-20 | 4 |
| M_10_F_lp_Set2 | 0.00 | 0.09 | 3435.04 | 78.19 | 1.48E-19 | 5 |
| M_0.8_F_qph_Set1 | 0.00 | 0.09 | 3435.96 | 79.11 | 8.20E-21 | 49 |
| M_0.4_F_qph_Set2 | 0.00 | 0.09 | 3436.85 | 80.00 | 3.68E-21 | 51 |
| M_10_F_lt_Set2 | 0.00 | 0.09 | 3436.93 | 80.08 | 6.02E-20 | 5 |
| M_0.5_F_qh_Set2 | 0.00 | 0.09 | 3437.08 | 80.23 | 3.44E-21 | 49 |
| M_10_F_t_Set3 | 0.00 | 0.09 | 3437.69 | 80.84 | 3.54E-20 | 5 |
| M_0.1_F_lp_Set1 | 0.00 | 0.09 | 3440.53 | 83.68 | 4.80E-22 | 46 |
| M_0.4_F_lph_Set2 | 0.00 | 0.09 | 3453.80 | 96.96 | 7.62E-25 | 55 |
| M_0.6_F_p_Set3 | 0.00 | 0.09 | 3464.03 | 107.18 | 5.13E-27 | 6 |
| M_0.7_F_p_Set3 | 0.00 | 0.09 | 3464.32 | 107.47 | 4.83E-27 | 6 |
| M_0.8_F_p_Set3 | 0.00 | 0.09 | 3464.70 | 107.85 | 4.41E-27 | 6 |
| M_0.9_F_p_Set3 | 0.00 | 0.09 | 3465.12 | 108.27 | 3.97E-27 | 6 |
| M_1_F_p_Set3 | 0.00 | 0.09 | 3465.56 | 108.71 | 3.60E-27 | 6 |
| M_0.7_F_lq_Set3 | 0.00 | 0.10 | 3372.22 | 15.37 | 4.89E-07 | 6 |
| M_0.8_F_lq_Set3 | 0.00 | 0.10 | 3372.93 | 16.08 | 3.76E-07 | 6 |
| M_0.6_F_lq_Set3 | 0.00 | 0.10 | 3373.44 | 16.59 | 2.43E-07 | 7 |
| M_0.9_F_lq_Set3 | 0.00 | 0.10 | 3373.45 | 16.60 | 3.24E-07 | 6 |
| M_1_F_lq_Set3 | 0.00 | 0.10 | 3374.34 | 17.49 | 2.33E-07 | 6 |
| M_2_F_lqt_Set3 | 0.00 | 0.10 | 3374.60 | 17.75 | 2.49E-07 | 16 |
| M_2_F_lpth_Set1 | 0.00 | 0.10 | 3377.02 | 20.17 | 7.87E-08 | 28 |
| M_1_F_lp_Set1 | 0.00 | 0.10 | 3377.66 | 20.81 | 4.46E-08 | 18 |
| M_1_F_p_Set1 | 0.00 | 0.10 | 3377.66 | 20.81 | 4.36E-08 | 17 |
| M_0.9_F_lqph_Set3 | 0.00 | 0.10 | 3377.99 | 21.14 | 3.62E-08 | 23 |
| M_0.8_F_lqp_Set1 | 0.00 | 0.10 | 3378.68 | 21.83 | 2.20E-08 | 20 |
| M_0.7_F_qp_Set3 | 0.00 | 0.10 | 3378.84 | 21.99 | 1.81E-08 | 9 |
| M_4_F_qth_Set1 | 0.00 | 0.10 | 3379.23 | 22.38 | 3.72E-08 | 15 |
| M_0.9_F_p_Set1 | 0.00 | 0.10 | 3379.63 | 22.78 | 1.45E-08 | 18 |
| M_0.9_F_qh_Set3 | 0.00 | 0.10 | 3380.11 | 23.26 | 1.19E-08 | 22 |
| M_2_F_lqpth_Set1 | 0.00 | 0.10 | 3380.11 | 23.26 | 1.69E-08 | 29 |
| M_0.8_F_qp_Set3 | 0.00 | 0.10 | 3380.72 | 23.88 | 7.76E-09 | 9 |
| M_0.9_F_qp_Set3 | 0.00 | 0.10 | 3382.71 | 25.86 | 3.21E-09 | 9 |
| M_0.8_F_lqph_Set3 | 0.00 | 0.10 | 3383.74 | 26.89 | 1.82E-09 | 26 |
| M_0.9_F_lqp_Set1 | 0.00 | 0.10 | 3384.07 | 27.22 | 1.66E-09 | 22 |
| M_0.9_F_lp_Set1 | 0.00 | 0.10 | 3384.88 | 28.04 | 1.06E-09 | 21 |
| M_2_F_pth_Set1 | 0.00 | 0.10 | 3385.26 | 28.41 | 1.25E-09 | 31 |
| M_0.7_F_p_Set1 | 0.00 | 0.10 | 3386.19 | 29.34 | 4.47E-10 | 21 |
| M_1_F_qp_Set3 | 0.00 | 0.10 | 3386.25 | 29.40 | 6.15E-10 | 9 |
| M_4_F_ph_Set1 | 0.00 | 0.10 | 3387.78 | 30.93 | 4.81E-10 | 18 |
| M_1_F_qh_Set1 | 0.00 | 0.10 | 3387.96 | 31.11 | 2.64E-10 | 33 |
| M_0.8_F_pt_Set3 | 0.00 | 0.10 | 3388.54 | 31.69 | 1.57E-10 | 38 |
| M_0.9_F_qph_Set3 | 0.00 | 0.10 | 3388.63 | 31.78 | 1.75E-10 | 27 |
| M_0.8_F_ph_Set3 | 0.00 | 0.10 | 3388.79 | 31.94 | 1.39E-10 | 28 |
| M_4_F_pth_Set1 | 0.00 | 0.10 | 3388.83 | 31.98 | 3.08E-10 | 19 |
| M_0.6_F_qh_Set3 | 0.00 | 0.10 | 3389.32 | 32.47 | 8.81E-11 | 29 |
| M_4_F_qh_Set1 | 0.00 | 0.10 | 3389.76 | 32.91 | 1.76E-10 | 18 |
| M_0.7_F_qh_Set3 | 0.00 | 0.10 | 3389.95 | 33.10 | 7.01E-11 | 28 |
| M_6_F_lqpth_Set2 | 0.00 | 0.10 | 3390.20 | 33.35 | 3.20E-10 | 9 |
| M_0.7_F_pt_Set3 | 0.00 | 0.10 | 3390.32 | 33.47 | 5.86E-11 | 41 |
| M_0.9_F_lqpth_Set3 | 0.00 | 0.10 | 3390.63 | 33.78 | 6.61E-11 | 35 |
| M_1_F_lqth_Set2 | 0.00 | 0.10 | 3391.03 | 34.18 | 6.09E-11 | 37 |
| M_0.8_F_pth_Set3 | 0.00 | 0.10 | 3391.25 | 34.40 | 4.23E-11 | 38 |
| M_0.6_F_lqh_Set3 | 0.00 | 0.10 | 3391.44 | 34.59 | 3.11E-11 | 30 |
| M_0.8_F_qph_Set3 | 0.00 | 0.10 | 3391.76 | 34.91 | 3.25E-11 | 29 |
| M_0.7_F_lqph_Set3 | 0.00 | 0.10 | 3391.98 | 35.13 | 2.67E-11 | 30 |
| M_0.9_F_lpth_Set3 | 0.00 | 0.10 | 3392.19 | 35.34 | 3.01E-11 | 36 |
| M_0.9_F_ph_Set3 | 0.00 | 0.10 | 3394.61 | 37.76 | 8.48E-12 | 29 |
| M_2_F_ph_Set1 | 0.00 | 0.10 | 3394.80 | 37.95 | 1.00E-11 | 30 |
| M_0.9_F_lh_Set1 | 0.00 | 0.10 | 3394.83 | 37.98 | 7.42E-12 | 37 |
| M_0.7_F_lqh_Set3 | 0.00 | 0.10 | 3395.30 | 38.45 | 4.93E-12 | 30 |
| M_1_F_qth_Set2 | 0.00 | 0.10 | 3396.73 | 39.88 | 3.46E-12 | 38 |
| M_0.6_F_lh_Set3 | 0.00 | 0.10 | 3397.36 | 40.51 | 1.57E-12 | 32 |
| M_10_F_lp_Set1 | 0.00 | 0.10 | 3397.84 | 40.99 | 1.75E-11 | 7 |
| M_0.7_F_lh_Set3 | 0.00 | 0.10 | 3398.03 | 41.18 | 1.22E-12 | 31 |
| M_5_F_qpt_Set2 | 0.00 | 0.10 | 3398.16 | 41.31 | 3.69E-12 | 10 |
| M_8_F_p_Set1 | 0.00 | 0.10 | 3398.23 | 41.38 | 6.04E-12 | 8 |
| M_8_F_pt_Set1 | 0.00 | 0.10 | 3398.31 | 41.46 | 7.06E-12 | 8 |
| M_8_F_ph_Set1 | 0.00 | 0.10 | 3398.32 | 41.47 | 7.17E-12 | 10 |
| M_0.7_F_pth_Set3 | 0.00 | 0.10 | 3399.97 | 43.12 | 4.88E-13 | 43 |
| M_0.9_F_h_Set1 | 0.00 | 0.10 | 3400.02 | 43.17 | 5.46E-13 | 38 |
| M_0.5_F_qh_Set3 | 0.00 | 0.10 | 3400.05 | 43.20 | 3.78E-13 | 34 |
| M_10_F_lpth_Set1 | 0.00 | 0.10 | 3400.07 | 43.22 | 4.67E-11 | 8 |
| M_0.3_F_l_Set1 | 0.00 | 0.10 | 3400.19 | 43.34 | 3.00E-13 | 11 |
| M_10_F_lpt_Set1 | 0.00 | 0.10 | 3400.24 | 43.39 | 1.06E-11 | 8 |
| M_8_F_pth_Set1 | 0.00 | 0.10 | 3400.56 | 43.71 | 3.00E-12 | 11 |
| M_0.8_F_qth_Set3 | 0.00 | 0.10 | 3400.92 | 44.07 | 3.36E-13 | 39 |
| M_0.9_F_qth_Set2 | 0.00 | 0.10 | 3401.80 | 44.96 | 2.41E-13 | 42 |
| M_10_F_lph_Set1 | 0.00 | 0.10 | 3402.34 | 45.50 | 4.02E-12 | 9 |
| M_0.7_F_t_Set3 | 0.00 | 0.10 | 3403.11 | 46.26 | 9.53E-14 | 43 |
| M_0.7_F_qph_Set3 | 0.00 | 0.10 | 3403.63 | 46.78 | 7.77E-14 | 34 |
| M_0.6_F_lqph_Set3 | 0.00 | 0.10 | 3403.71 | 46.86 | 6.90E-14 | 35 |
| M_0.6_F_qph_Set3 | 0.00 | 0.10 | 3403.72 | 46.87 | 6.78E-14 | 35 |
| M_0.7_F_lqpth_Set3 | 0.00 | 0.10 | 3404.02 | 47.17 | 6.56E-14 | 44 |
| M_0.7_F_lh_Set1 | 0.00 | 0.10 | 3404.85 | 48.00 | 4.04E-14 | 42 |
| M_0.6_F_lh_Set1 | 0.00 | 0.10 | 3405.02 | 48.18 | 3.39E-14 | 43 |
| M_0.5_F_lh_Set3 | 0.00 | 0.10 | 3405.78 | 48.93 | 2.14E-14 | 36 |
| M_0.7_F_lph_Set3 | 0.00 | 0.10 | 3406.35 | 49.50 | 1.98E-14 | 35 |
| M_0.9_F_lh_Set3 | 0.00 | 0.10 | 3407.40 | 50.55 | 1.39E-14 | 32 |
| M_0.7_F_th_Set3 | 0.00 | 0.10 | 3408.63 | 51.78 | 6.23E-15 | 44 |
| M_0.5_F_lqh_Set3 | 0.00 | 0.10 | 3408.90 | 52.05 | 4.61E-15 | 37 |
| M_0.5_F_lqph_Set3 | 0.00 | 0.10 | 3409.48 | 52.63 | 3.53E-15 | 38 |
| M_0.8_F_lqth_Set3 | 0.00 | 0.10 | 3409.57 | 52.72 | 4.50E-15 | 42 |
| M_0.7_F_ph_Set3 | 0.00 | 0.10 | 3409.57 | 52.72 | 3.88E-15 | 36 |
| M_0.5_F_qph_Set3 | 0.00 | 0.10 | 3409.82 | 52.97 | 2.95E-15 | 38 |
| M_0.7_F_lqth_Set3 | 0.00 | 0.10 | 3411.72 | 54.87 | 1.39E-15 | 45 |
| M_0.8_F_th_Set2 | 0.00 | 0.10 | 3411.99 | 55.14 | 1.28E-15 | 47 |
| M_0.5_F_h_Set3 | 0.00 | 0.10 | 3412.75 | 55.90 | 6.52E-16 | 38 |
| M_6_F_t_Set2 | 0.00 | 0.10 | 3415.23 | 58.38 | 8.29E-16 | 12 |
| M_0.7_F_qth_Set3 | 0.00 | 0.10 | 3415.67 | 58.82 | 1.89E-16 | 46 |
| M_0.5_F_lph_Set3 | 0.00 | 0.10 | 3415.76 | 58.91 | 1.50E-16 | 40 |
| M_0.6_F_lph_Set3 | 0.00 | 0.10 | 3415.97 | 59.12 | 1.48E-16 | 39 |
| M_0.8_F_qth_Set2 | 0.00 | 0.10 | 3417.57 | 60.72 | 8.10E-17 | 49 |
| M_0.4_F_qh_Set3 | 0.00 | 0.10 | 3418.34 | 61.49 | 3.75E-17 | 41 |
| M_0.4_F_lph_Set3 | 0.00 | 0.10 | 3419.01 | 62.16 | 2.74E-17 | 42 |
| M_0.6_F_h_Set1 | 0.00 | 0.10 | 3419.58 | 62.73 | 2.31E-17 | 47 |
| M_8_F_t_Set2 | 0.00 | 0.10 | 3421.02 | 64.17 | 6.97E-17 | 7 |
| M_0.6_F_lqth_Set3 | 0.00 | 0.10 | 3421.58 | 64.73 | 9.08E-18 | 50 |
| M_0.4_F_h_Set3 | 0.00 | 0.10 | 3422.11 | 65.26 | 5.60E-18 | 42 |
| M_0.4_F_ph_Set3 | 0.00 | 0.10 | 3422.49 | 65.64 | 4.75E-18 | 43 |
| M_0.6_F_ph_Set3 | 0.00 | 0.10 | 3425.94 | 69.09 | 9.87E-19 | 42 |
| M_0.8_F_ph_Set1 | 0.00 | 0.10 | 3426.97 | 70.12 | 7.11E-19 | 47 |
| M_0.4_F_qph_Set3 | 0.00 | 0.10 | 3429.47 | 72.62 | 1.47E-19 | 45 |
| M_0.2_F_p_Set1 | 0.00 | 0.10 | 3430.59 | 73.74 | 7.15E-20 | 38 |
| M_0.4_F_lqph_Set3 | 0.00 | 0.10 | 3433.18 | 76.33 | 2.33E-20 | 46 |
| M_0.6_F_th_Set3 | 0.00 | 0.10 | 3433.51 | 76.66 | 2.26E-20 | 53 |
| M_0.6_F_qth_Set3 | 0.00 | 0.10 | 3433.57 | 76.72 | 2.24E-20 | 53 |
| M_0.1_F_p_Set1 | 0.00 | 0.10 | 3445.29 | 88.44 | 4.41E-23 | 44 |
| M_0.4_F_h_Set2 | 0.00 | 0.10 | 3448.31 | 91.46 | 1.14E-23 | 53 |
| M_0.1_F_qp_Set1 | 0.00 | 0.10 | 3457.81 | 100.96 | 8.54E-26 | 48 |
| M_0.7_F_lqt_Set2 | 0.00 | 0.10 | 3463.20 | 106.35 | 8.84E-27 | 64 |
| M_0.2_F_lqp_Set1 | 0.00 | 0.10 | 3466.05 | 109.20 | 1.46E-27 | 52 |
| M_0.4_F_lqph_Set2 | 0.00 | 0.10 | 3466.11 | 109.26 | 1.65E-27 | 58 |
| M_2_F_qh_Set1 | 0.00 | 0.11 | 3364.07 | 7.22 | 4.67E-05 | 18 |
| M_2_F_th_Set1 | 0.00 | 0.11 | 3364.29 | 7.44 | 4.26E-05 | 22 |
| M_2_F_t_Set3 | 0.00 | 0.11 | 3364.44 | 7.59 | 3.75E-05 | 15 |
| M_2_F_qth_Set1 | 0.00 | 0.11 | 3364.78 | 7.93 | 3.47E-05 | 23 |
| M_2_F_pt_Set3 | 0.00 | 0.11 | 3367.49 | 10.64 | 8.51E-06 | 16 |
| M_2_F_lqh_Set1 | 0.00 | 0.11 | 3368.19 | 11.34 | 6.16E-06 | 20 |
| M_1_F_qt_Set3 | 0.00 | 0.11 | 3369.93 | 13.08 | 2.16E-06 | 26 |
| M_2_F_lt_Set3 | 0.00 | 0.11 | 3369.99 | 13.14 | 2.38E-06 | 14 |
| M_2_F_lqth_Set1 | 0.00 | 0.11 | 3372.26 | 15.41 | 8.45E-07 | 26 |
| M_1_F_lqt_Set3 | 0.00 | 0.11 | 3372.75 | 15.90 | 5.45E-07 | 27 |
| M_1_F_lt_Set3 | 0.00 | 0.11 | 3372.80 | 15.95 | 5.10E-07 | 27 |
| M_1_F_t_Set3 | 0.00 | 0.11 | 3372.99 | 16.14 | 4.54E-07 | 26 |
| M_2_F_lh_Set1 | 0.00 | 0.11 | 3373.42 | 16.57 | 4.29E-07 | 22 |
| M_0.9_F_lt_Set3 | 0.00 | 0.11 | 3378.53 | 21.68 | 2.57E-08 | 31 |
| M_0.9_F_qt_Set3 | 0.00 | 0.11 | 3379.07 | 22.22 | 1.99E-08 | 31 |
| M_1_F_pt_Set3 | 0.00 | 0.11 | 3379.95 | 23.10 | 1.46E-08 | 31 |
| M_0.9_F_t_Set3 | 0.00 | 0.11 | 3380.81 | 23.96 | 8.11E-09 | 31 |
| M_0.9_F_th_Set2 | 0.00 | 0.11 | 3381.75 | 24.90 | 5.28E-09 | 35 |
| M_0.9_F_lqt_Set3 | 0.00 | 0.11 | 3381.76 | 24.91 | 5.29E-09 | 32 |
| M_3_F_t_Set3 | 0.00 | 0.11 | 3381.89 | 25.04 | 7.12E-09 | 13 |
| M_0.9_F_pt_Set3 | 0.00 | 0.11 | 3383.38 | 26.53 | 2.32E-09 | 34 |
| M_0.8_F_t_Set3 | 0.00 | 0.11 | 3385.09 | 28.25 | 8.54E-10 | 35 |
| M_0.8_F_lt_Set3 | 0.00 | 0.11 | 3386.71 | 29.86 | 3.86E-10 | 36 |
| M_1_F_th_Set2 | 0.00 | 0.11 | 3387.26 | 30.41 | 3.80E-10 | 34 |
| M_3_F_pt_Set3 | 0.00 | 0.11 | 3387.96 | 31.11 | 3.61E-10 | 13 |
| M_0.1_F_lp_Set2 | 0.00 | 0.11 | 3388.70 | 31.85 | 8.62E-11 | 24 |
| M_0.9_F_lqh_Set3 | 0.00 | 0.11 | 3389.36 | 32.51 | 1.19E-10 | 26 |
| M_4_F_t_Set3 | 0.00 | 0.11 | 3389.73 | 32.88 | 1.70E-10 | 9 |
| M_0.8_F_qt_Set3 | 0.00 | 0.11 | 3390.77 | 33.93 | 5.12E-11 | 37 |
| M_0.8_F_lpth_Set3 | 0.00 | 0.11 | 3391.27 | 34.42 | 4.24E-11 | 38 |
| M_0.7_F_lt_Set3 | 0.00 | 0.11 | 3392.34 | 35.49 | 2.10E-11 | 40 |
| M_0.7_F_qt_Set3 | 0.00 | 0.11 | 3392.93 | 36.08 | 1.58E-11 | 40 |
| M_0.8_F_lqt_Set3 | 0.00 | 0.11 | 3393.23 | 36.38 | 1.53E-11 | 38 |
| M_3_F_qt_Set3 | 0.00 | 0.11 | 3393.83 | 36.98 | 1.89E-11 | 14 |
| M_1_F_lh_Set1 | 0.00 | 0.11 | 3394.22 | 37.37 | 1.14E-11 | 36 |
| M_0.8_F_lqpth_Set3 | 0.00 | 0.11 | 3395.67 | 38.82 | 4.72E-12 | 39 |
| M_5_F_t_Set3 | 0.00 | 0.11 | 3397.12 | 40.27 | 5.29E-12 | 8 |
| M_4_F_pt_Set3 | 0.00 | 0.11 | 3397.53 | 40.68 | 3.66E-12 | 10 |
| M_0.7_F_lqt_Set3 | 0.00 | 0.11 | 3399.17 | 42.32 | 7.10E-13 | 42 |
| M_1_F_lqh_Set1 | 0.00 | 0.11 | 3399.91 | 43.06 | 6.88E-13 | 37 |
| M_0.7_F_lpth_Set3 | 0.00 | 0.11 | 3400.21 | 43.36 | 4.38E-13 | 43 |
| M_4_F_lt_Set1 | 0.00 | 0.11 | 3401.66 | 44.81 | 4.45E-13 | 18 |
| M_0.9_F_lqth_Set2 | 0.00 | 0.11 | 3403.56 | 46.71 | 1.02E-13 | 43 |
| M_0.6_F_pt_Set3 | 0.00 | 0.11 | 3410.30 | 53.45 | 2.46E-15 | 49 |
| M_0.6_F_qh_Set1 | 0.00 | 0.11 | 3411.91 | 55.06 | 1.09E-15 | 45 |
| M_0.6_F_lt_Set3 | 0.00 | 0.11 | 3412.07 | 55.23 | 9.96E-16 | 48 |
| M_0.6_F_lqt_Set3 | 0.00 | 0.11 | 3412.09 | 55.24 | 1.02E-15 | 48 |
| M_0.9_F_ph_Set1 | 0.00 | 0.11 | 3412.73 | 55.88 | 9.84E-16 | 42 |
| M_0.8_F_h_Set1 | 0.00 | 0.11 | 3414.09 | 57.24 | 4.33E-16 | 43 |
| M_0.6_F_lqh_Set1 | 0.00 | 0.11 | 3415.77 | 58.92 | 1.61E-16 | 46 |
| M_0.6_F_t_Set3 | 0.00 | 0.11 | 3415.78 | 58.93 | 1.54E-16 | 49 |
| M_0.8_F_lqth_Set2 | 0.00 | 0.11 | 3415.96 | 59.11 | 1.84E-16 | 49 |
| M_0.6_F_qt_Set3 | 0.00 | 0.11 | 3416.04 | 59.19 | 1.39E-16 | 49 |
| M_0.4_F_lqh_Set3 | 0.00 | 0.11 | 3418.22 | 61.37 | 4.06E-17 | 41 |
| M_0.4_F_lh_Set3 | 0.00 | 0.11 | 3421.66 | 64.81 | 7.10E-18 | 42 |
| M_0.6_F_pth_Set3 | 0.00 | 0.11 | 3424.11 | 67.26 | 2.55E-18 | 52 |
| M_0.6_F_lpth_Set3 | 0.00 | 0.11 | 3424.11 | 67.26 | 2.58E-18 | 52 |
| M_0.3_F_h_Set3 | 0.00 | 0.11 | 3424.28 | 67.43 | 1.79E-18 | 44 |
| M_0.6_F_lqpth_Set3 | 0.00 | 0.11 | 3424.41 | 67.56 | 2.22E-18 | 52 |
| M_0.9_F_lqt_Set2 | 0.00 | 0.11 | 3425.29 | 68.44 | 1.87E-18 | 50 |
| M_0.8_F_lph_Set1 | 0.00 | 0.11 | 3430.93 | 74.08 | 1.01E-19 | 48 |
| M_0.7_F_h_Set1 | 0.00 | 0.11 | 3431.78 | 74.93 | 5.66E-20 | 49 |
| M_0.4_F_lh_Set2 | 0.00 | 0.11 | 3439.49 | 82.64 | 9.50E-22 | 51 |
| M_0.8_F_lqt_Set2 | 0.00 | 0.11 | 3440.00 | 83.15 | 1.07E-21 | 56 |
| M_0.7_F_lqph_Set1 | 0.00 | 0.11 | 3454.16 | 97.31 | 8.37E-25 | 55 |
| M_0.4_F_lqh_Set2 | 0.00 | 0.11 | 3463.72 | 106.87 | 5.33E-27 | 57 |
| M_0.1_F_lqp_Set1 | 0.00 | 0.11 | 3465.52 | 108.68 | 1.82E-27 | 52 |
| M_0.4_F_h_Set1 | 0.00 | 0.11 | 3465.79 | 108.94 | 1.83E-27 | 61 |
| M_0.5_F_lqth_Set3 | 0.00 | 0.11 | 3466.54 | 109.69 | 1.44E-27 | 64 |
| M_0.7_F_qt_Set2 | 0.00 | 0.11 | 3473.76 | 116.91 | 4.43E-29 | 66 |
| M_0.5_F_th_Set3 | 0.00 | 0.11 | 3481.07 | 124.22 | 9.76E-31 | 67 |
| M_0.5_F_lqpth_Set3 | 0.00 | 0.11 | 3481.80 | 124.95 | 7.04E-31 | 68 |
| M_0.5_F_pth_Set3 | 0.00 | 0.11 | 3481.81 | 124.96 | 6.90E-31 | 68 |
| M_0.5_F_lpth_Set3 | 0.00 | 0.11 | 3481.81 | 124.96 | 6.98E-31 | 68 |
| M_0.3_F_lph_Set2 | 0.00 | 0.11 | 3482.42 | 125.57 | 4.37E-31 | 63 |
| M_0.5_F_qth_Set3 | 0.00 | 0.11 | 3486.30 | 129.45 | 7.30E-32 | 68 |
| M_0.3_F_lqph_Set2 | 0.00 | 0.11 | 3487.81 | 130.96 | 2.97E-32 | 64 |
| M_0.3_F_h_Set1 | 0.00 | 0.11 | 3511.90 | 155.05 | 1.67E-37 | 72 |
| M_2_F_lpt_Set3 | 0.00 | 0.12 | 3369.09 | 12.24 | 3.97E-06 | 14 |
| M_0.8_F_qh_Set3 | 0.00 | 0.12 | 3371.18 | 14.33 | 9.22E-07 | 20 |
| M_2_F_qt_Set3 | 0.00 | 0.12 | 3372.98 | 16.13 | 5.43E-07 | 17 |
| M_2_F_qpt_Set3 | 0.00 | 0.12 | 3373.17 | 16.32 | 5.20E-07 | 17 |
| M_2_F_lqpt_Set3 | 0.00 | 0.12 | 3376.15 | 19.30 | 1.20E-07 | 17 |
| M_2_F_qt_Set1 | 0.00 | 0.12 | 3379.84 | 22.99 | 1.75E-08 | 27 |
| M_2_F_lpt_Set1 | 0.00 | 0.12 | 3380.37 | 23.52 | 1.40E-08 | 27 |
| M_0.8_F_lqh_Set3 | 0.00 | 0.12 | 3381.43 | 24.58 | 5.62E-09 | 24 |
| M_2_F_qpt_Set1 | 0.00 | 0.12 | 3381.81 | 24.96 | 6.91E-09 | 28 |
| M_2_F_lt_Set1 | 0.00 | 0.12 | 3385.13 | 28.28 | 1.23E-09 | 29 |
| M_2_F_lqt_Set1 | 0.00 | 0.12 | 3386.05 | 29.20 | 8.10E-10 | 29 |
| M_2_F_pt_Set1 | 0.00 | 0.12 | 3386.31 | 29.46 | 6.97E-10 | 29 |
| M_2_F_lqpt_Set1 | 0.00 | 0.12 | 3389.00 | 32.15 | 1.93E-10 | 31 |
| M_0.8_F_qpt_Set3 | 0.00 | 0.12 | 3391.42 | 34.57 | 3.84E-11 | 39 |
| M_3_F_qpt_Set3 | 0.00 | 0.12 | 3391.46 | 34.61 | 6.59E-11 | 13 |
| M_3_F_lt_Set1 | 0.00 | 0.12 | 3392.09 | 35.24 | 4.42E-11 | 21 |
| M_2_F_lqt_Set2 | 0.00 | 0.12 | 3393.13 | 36.28 | 2.35E-11 | 27 |
| M_0.7_F_qpt_Set3 | 0.00 | 0.12 | 3393.67 | 36.82 | 1.13E-11 | 42 |
| M_0.7_F_lqpt_Set3 | 0.00 | 0.12 | 3396.81 | 39.96 | 2.37E-12 | 43 |
| M_4_F_t_Set2 | 0.00 | 0.12 | 3399.93 | 43.08 | 1.03E-12 | 14 |
| M_3_F_t_Set2 | 0.00 | 0.12 | 3400.07 | 43.22 | 8.01E-13 | 22 |
| M_3_F_qt_Set2 | 0.00 | 0.12 | 3400.45 | 43.60 | 6.90E-13 | 21 |
| M_0.6_F_qpt_Set3 | 0.00 | 0.12 | 3406.88 | 50.03 | 1.39E-14 | 48 |
| M_5_F_t_Set2 | 0.00 | 0.12 | 3407.32 | 50.47 | 3.23E-14 | 13 |
| M_5_F_pt_Set3 | 0.00 | 0.12 | 3411.43 | 54.58 | 4.50E-15 | 10 |
| M_0.8_F_pth_Set2 | 0.00 | 0.12 | 3420.14 | 63.29 | 2.25E-17 | 51 |
| M_0.3_F_lh_Set3 | 0.00 | 0.12 | 3427.33 | 70.48 | 3.91E-19 | 45 |
| M_0.3_F_lqh_Set3 | 0.00 | 0.12 | 3427.68 | 70.83 | 3.35E-19 | 45 |
| M_0.3_F_qh_Set3 | 0.00 | 0.12 | 3428.02 | 71.17 | 2.78E-19 | 45 |
| M_0.8_F_lpth_Set2 | 0.00 | 0.12 | 3428.12 | 71.27 | 4.22E-19 | 53 |
| M_0.5_F_h_Set1 | 0.00 | 0.12 | 3433.46 | 76.61 | 2.07E-20 | 52 |
| M_0.7_F_lt_Set2 | 0.00 | 0.12 | 3444.15 | 87.30 | 1.18E-22 | 60 |
| M_0.5_F_lqt_Set3 | 0.00 | 0.12 | 3455.91 | 99.06 | 2.85E-25 | 62 |
| M_0.3_F_h_Set2 | 0.00 | 0.12 | 3457.56 | 100.71 | 1.06E-25 | 57 |
| M_0.3_F_lh_Set2 | 0.00 | 0.12 | 3466.36 | 109.51 | 1.31E-27 | 59 |
| M_0.4_F_qh_Set2 | 0.00 | 0.12 | 3468.29 | 111.44 | 5.33E-28 | 58 |
| M_0.3_F_lqph_Set3 | 0.00 | 0.12 | 3468.35 | 111.50 | 5.01E-28 | 56 |
| M_0.5_F_qt_Set3 | 0.00 | 0.12 | 3469.98 | 113.13 | 2.48E-28 | 65 |
| M_0.4_F_lh_Set1 | 0.00 | 0.12 | 3470.23 | 113.38 | 2.01E-28 | 62 |
| M_0.6_F_lqph_Set1 | 0.00 | 0.12 | 3470.31 | 113.46 | 2.37E-28 | 60 |
| M_0.3_F_qh_Set2 | 0.00 | 0.12 | 3478.84 | 121.99 | 2.57E-30 | 62 |
| M_0.4_F_lqh_Set1 | 0.00 | 0.12 | 3488.94 | 132.10 | 1.77E-32 | 66 |
| M_0.3_F_qh_Set1 | 0.00 | 0.12 | 3496.03 | 139.18 | 4.75E-34 | 69 |
| M_0.3_F_lqh_Set2 | 0.00 | 0.12 | 3504.09 | 147.24 | 8.54E-36 | 67 |
| M_0.3_F_lh_Set1 | 0.00 | 0.12 | 3523.96 | 167.11 | 4.07E-40 | 74 |
| M_1_F_qpt_Set3 | 0.00 | 0.13 | 3380.05 | 23.20 | 1.44E-08 | 31 |
| M_1_F_lqpt_Set3 | 0.00 | 0.13 | 3380.22 | 23.37 | 1.35E-08 | 31 |
| M_1_F_lpt_Set3 | 0.00 | 0.13 | 3380.26 | 23.41 | 1.29E-08 | 31 |
| M_2_F_t_Set2 | 0.00 | 0.13 | 3382.81 | 25.96 | 3.83E-09 | 26 |
| M_0.9_F_pth_Set2 | 0.00 | 0.13 | 3383.87 | 27.02 | 1.90E-09 | 38 |
| M_2_F_lt_Set2 | 0.00 | 0.13 | 3384.17 | 27.32 | 1.98E-09 | 25 |
| M_1_F_lqpth_Set2 | 0.00 | 0.13 | 3384.69 | 27.84 | 1.47E-09 | 36 |
| M_1_F_th_Set1 | 0.00 | 0.13 | 3384.97 | 28.12 | 1.19E-09 | 41 |
| M_1_F_pth_Set2 | 0.00 | 0.13 | 3385.05 | 28.20 | 1.20E-09 | 36 |
| M_0.9_F_lpt_Set3 | 0.00 | 0.13 | 3386.42 | 29.57 | 5.21E-10 | 35 |
| M_0.9_F_lqpt_Set3 | 0.00 | 0.13 | 3386.42 | 29.57 | 5.34E-10 | 35 |
| M_1_F_t_Set2 | 0.00 | 0.13 | 3387.09 | 30.24 | 3.93E-10 | 36 |
| M_0.8_F_lpt_Set3 | 0.00 | 0.13 | 3388.63 | 31.78 | 1.54E-10 | 38 |
| M_0.9_F_qpt_Set3 | 0.00 | 0.13 | 3389.37 | 32.52 | 1.20E-10 | 36 |
| M_2_F_qt_Set2 | 0.00 | 0.13 | 3389.42 | 32.57 | 1.46E-10 | 27 |
| M_1_F_lpth_Set2 | 0.00 | 0.13 | 3390.65 | 33.80 | 7.44E-11 | 38 |
| M_0.7_F_lpt_Set3 | 0.00 | 0.13 | 3390.69 | 33.84 | 4.96E-11 | 41 |
| M_0.9_F_t_Set2 | 0.00 | 0.13 | 3393.19 | 36.34 | 1.66E-11 | 40 |
| M_0.8_F_lqpt_Set3 | 0.00 | 0.13 | 3394.57 | 37.72 | 8.08E-12 | 40 |
| M_1_F_lt_Set1 | 0.00 | 0.13 | 3396.23 | 39.38 | 4.16E-12 | 46 |
| M_1_F_t_Set1 | 0.00 | 0.13 | 3400.74 | 43.89 | 4.28E-13 | 47 |
| M_0.9_F_lqpth_Set2 | 0.00 | 0.13 | 3403.37 | 46.52 | 1.13E-13 | 44 |
| M_0.9_F_lpth_Set2 | 0.00 | 0.13 | 3403.50 | 46.65 | 1.05E-13 | 44 |
| M_1_F_lt_Set2 | 0.00 | 0.13 | 3404.75 | 47.90 | 5.88E-14 | 43 |
| M_1_F_qt_Set2 | 0.00 | 0.13 | 3406.60 | 49.75 | 2.36E-14 | 43 |
| M_0.6_F_lqpt_Set3 | 0.00 | 0.13 | 3406.88 | 50.03 | 1.41E-14 | 48 |
| M_0.6_F_lpt_Set3 | 0.00 | 0.13 | 3410.30 | 53.45 | 2.50E-15 | 49 |
| M_0.9_F_t_Set1 | 0.00 | 0.13 | 3411.57 | 54.72 | 1.69E-15 | 52 |
| M_0.9_F_pt_Set2 | 0.00 | 0.13 | 3411.77 | 54.92 | 1.59E-15 | 48 |
| M_1_F_pt_Set2 | 0.00 | 0.13 | 3411.89 | 55.04 | 1.69E-15 | 46 |
| M_0.9_F_lt_Set2 | 0.00 | 0.13 | 3412.03 | 55.18 | 1.37E-15 | 47 |
| M_1_F_lqt_Set2 | 0.00 | 0.13 | 3412.58 | 55.73 | 1.22E-15 | 45 |
| M_0.9_F_lpt_Set2 | 0.00 | 0.13 | 3414.68 | 57.83 | 3.79E-16 | 49 |
| M_1_F_lpt_Set2 | 0.00 | 0.13 | 3423.02 | 66.17 | 6.66E-18 | 49 |
| M_0.8_F_t_Set2 | 0.00 | 0.13 | 3427.07 | 70.22 | 6.55E-19 | 52 |
| M_0.8_F_qt_Set2 | 0.00 | 0.13 | 3428.38 | 71.53 | 3.48E-19 | 53 |
| M_0.3_F_ph_Set3 | 0.00 | 0.13 | 3432.50 | 75.65 | 2.98E-20 | 47 |
| M_0.7_F_qph_Set1 | 0.00 | 0.13 | 3434.02 | 77.17 | 1.95E-20 | 50 |
| M_0.3_F_lph_Set3 | 0.00 | 0.13 | 3436.06 | 79.21 | 5.09E-21 | 48 |
| M_0.5_F_qpt_Set3 | 0.00 | 0.13 | 3446.75 | 89.91 | 2.80E-23 | 61 |
| M_0.5_F_lqpt_Set3 | 0.00 | 0.13 | 3446.75 | 89.91 | 2.84E-23 | 61 |
| M_0.5_F_pt_Set3 | 0.00 | 0.13 | 3446.83 | 89.98 | 2.64E-23 | 61 |
| M_0.7_F_pth_Set2 | 0.00 | 0.13 | 3449.33 | 92.48 | 9.33E-24 | 61 |
| M_0.7_F_lph_Set1 | 0.00 | 0.13 | 3449.46 | 92.61 | 8.61E-24 | 54 |
| M_0.7_F_t_Set2 | 0.00 | 0.13 | 3455.70 | 98.85 | 3.62E-25 | 62 |
| M_0.5_F_lt_Set3 | 0.00 | 0.13 | 3455.91 | 99.06 | 2.78E-25 | 62 |
| M_0.7_F_lqpth_Set2 | 0.00 | 0.13 | 3468.63 | 111.78 | 6.10E-28 | 65 |
| M_0.5_F_t_Set3 | 0.00 | 0.13 | 3469.87 | 113.02 | 2.56E-28 | 65 |
| M_0.7_F_lpth_Set2 | 0.00 | 0.13 | 3472.83 | 115.98 | 7.46E-29 | 66 |
| M_0.4_F_qh_Set1 | 0.00 | 0.13 | 3475.04 | 118.19 | 1.83E-29 | 63 |
| M_0.3_F_qph_Set2 | 0.00 | 0.13 | 3481.91 | 125.06 | 5.65E-31 | 63 |
| M_0.3_F_lqh_Set1 | 0.00 | 0.13 | 3541.16 | 184.31 | 7.61E-44 | 77 |
| M_0.3_F_qph_Set1 | 0.00 | 0.13 | 3628.70 | 271.85 | 7.54E-63 | 90 |
| M_2_F_lpt_Set2 | 0.00 | 0.14 | 3383.59 | 26.74 | 2.80E-09 | 25 |
| M_1_F_qth_Set1 | 0.00 | 0.14 | 3385.37 | 28.53 | 1.01E-09 | 41 |
| M_2_F_pt_Set2 | 0.00 | 0.14 | 3386.57 | 29.72 | 6.11E-10 | 26 |
| M_1_F_lqth_Set1 | 0.00 | 0.14 | 3392.38 | 35.53 | 3.10E-11 | 43 |
| M_1_F_qt_Set1 | 0.00 | 0.14 | 3392.96 | 36.11 | 2.16E-11 | 44 |
| M_2_F_t_Set1 | 0.00 | 0.14 | 3393.20 | 36.35 | 2.12E-11 | 32 |
| M_1_F_lqt_Set1 | 0.00 | 0.14 | 3396.16 | 39.31 | 4.47E-12 | 45 |
| M_1_F_pth_Set1 | 0.00 | 0.14 | 3397.00 | 40.15 | 3.04E-12 | 45 |
| M_1_F_lpt_Set1 | 0.00 | 0.14 | 3403.19 | 46.34 | 1.34E-13 | 47 |
| M_1_F_qpt_Set1 | 0.00 | 0.14 | 3404.29 | 47.44 | 7.81E-14 | 48 |
| M_3_F_t_Set1 | 0.00 | 0.14 | 3404.53 | 47.68 | 8.58E-14 | 24 |
| M_1_F_lpth_Set1 | 0.00 | 0.14 | 3404.68 | 47.83 | 6.64E-14 | 47 |
| M_4_F_t_Set1 | 0.00 | 0.14 | 3406.51 | 49.66 | 3.83E-14 | 17 |
| M_5_F_t_Set1 | 0.00 | 0.14 | 3406.86 | 50.01 | 4.04E-14 | 13 |
| M_0.9_F_qth_Set1 | 0.00 | 0.14 | 3412.29 | 55.44 | 1.27E-15 | 51 |
| M_0.9_F_lqth_Set1 | 0.00 | 0.14 | 3412.57 | 55.72 | 1.13E-15 | 51 |
| M_1_F_lqpth_Set1 | 0.00 | 0.14 | 3416.16 | 59.31 | 2.15E-16 | 50 |
| M_6_F_t_Set1 | 0.00 | 0.14 | 3417.10 | 60.25 | 3.24E-16 | 13 |
| M_0.9_F_qt_Set2 | 0.00 | 0.14 | 3417.72 | 60.87 | 8.01E-17 | 48 |
| M_0.9_F_th_Set1 | 0.00 | 0.14 | 3421.22 | 64.37 | 1.41E-17 | 53 |
| M_1_F_pt_Set1 | 0.00 | 0.14 | 3421.81 | 64.96 | 1.18E-17 | 52 |
| M_8_F_t_Set1 | 0.00 | 0.14 | 3422.15 | 65.30 | 3.93E-17 | 8 |
| M_1_F_lqpt_Set1 | 0.00 | 0.14 | 3423.70 | 66.85 | 4.86E-18 | 53 |
| M_0.8_F_lt_Set2 | 0.00 | 0.14 | 3426.04 | 69.19 | 1.11E-18 | 53 |
| M_0.8_F_pt_Set2 | 0.00 | 0.14 | 3426.38 | 69.53 | 9.55E-19 | 54 |
| M_0.8_F_lpt_Set2 | 0.00 | 0.14 | 3429.97 | 73.12 | 1.62E-19 | 55 |
| M_0.9_F_lpth_Set1 | 0.00 | 0.14 | 3432.37 | 75.52 | 5.67E-20 | 56 |
| M_0.5_F_qh_Set1 | 0.00 | 0.14 | 3434.23 | 77.38 | 1.44E-20 | 52 |
| M_10_F_t_Set1 | 0.00 | 0.14 | 3435.31 | 78.46 | 1.14E-19 | 7 |
| M_0.5_F_lqh_Set1 | 0.00 | 0.14 | 3437.93 | 81.08 | 2.29E-21 | 53 |
| M_0.7_F_ph_Set1 | 0.00 | 0.14 | 3438.02 | 81.17 | 2.58E-21 | 51 |
| M_0.6_F_lph_Set1 | 0.00 | 0.14 | 3440.68 | 83.83 | 6.32E-22 | 53 |
| M_0.2_F_lqh_Set3 | 0.00 | 0.14 | 3444.93 | 88.08 | 5.67E-23 | 51 |
| M_0.5_F_lpt_Set3 | 0.00 | 0.14 | 3446.83 | 89.98 | 2.69E-23 | 61 |
| M_0.3_F_qph_Set3 | 0.00 | 0.14 | 3455.71 | 98.86 | 2.77E-25 | 53 |
| M_0.2_F_lh_Set3 | 0.00 | 0.14 | 3456.88 | 100.03 | 1.42E-25 | 54 |
| M_0.2_F_h_Set3 | 0.00 | 0.14 | 3457.11 | 100.26 | 1.25E-25 | 54 |
| M_0.2_F_qh_Set3 | 0.00 | 0.14 | 3461.22 | 104.37 | 1.63E-26 | 55 |
| M_0.4_F_qt_Set3 | 0.00 | 0.14 | 3501.87 | 145.02 | 2.72E-35 | 77 |
| M_0.4_F_lqt_Set3 | 0.00 | 0.14 | 3501.87 | 145.02 | 2.77E-35 | 77 |
| M_0.1_F_qh_Set3 | 0.00 | 0.14 | 3512.36 | 155.51 | 1.22E-37 | 68 |
| M_0.1_F_qph_Set3 | 0.00 | 0.14 | 3517.44 | 160.59 | 9.77E-39 | 69 |
| M_0.1_F_h_Set3 | 0.00 | 0.14 | 3523.22 | 166.37 | 5.31E-40 | 70 |
| M_0.1_F_lqph_Set3 | 0.00 | 0.14 | 3527.51 | 170.67 | 6.38E-41 | 71 |
| M_0.2_F_lqh_Set2 | 0.00 | 0.14 | 3530.85 | 174.00 | 1.25E-41 | 74 |
| M_0.2_F_h_Set2 | 0.00 | 0.14 | 3531.05 | 174.20 | 1.10E-41 | 74 |
| M_0.1_F_lqh_Set3 | 0.00 | 0.14 | 3534.04 | 177.19 | 2.41E-42 | 72 |
| M_0.1_F_lh_Set3 | 0.00 | 0.14 | 3539.31 | 182.46 | 1.71E-43 | 73 |
| M_0.2_F_qh_Set1 | 0.00 | 0.14 | 3628.30 | 271.45 | 8.52E-63 | 92 |
| M_0.9_F_lqt_Set1 | 0.00 | 0.15 | 3399.02 | 42.17 | 9.45E-13 | 48 |
| M_0.9_F_qt_Set1 | 0.00 | 0.15 | 3399.24 | 42.39 | 8.25E-13 | 48 |
| M_1_F_qpt_Set2 | 0.00 | 0.15 | 3405.01 | 48.16 | 5.46E-14 | 44 |
| M_1_F_lqpt_Set2 | 0.00 | 0.15 | 3408.82 | 51.97 | 8.28E-15 | 45 |
| M_0.9_F_pt_Set1 | 0.00 | 0.15 | 3416.03 | 59.18 | 1.88E-16 | 53 |
| M_0.9_F_lpt_Set1 | 0.00 | 0.15 | 3421.19 | 64.34 | 1.46E-17 | 54 |
| M_0.5_F_lh_Set1 | 0.00 | 0.15 | 3433.24 | 76.39 | 2.33E-20 | 52 |
| M_0.2_F_lqph_Set3 | 0.00 | 0.15 | 3438.91 | 82.06 | 1.17E-21 | 50 |
| M_0.8_F_qpt_Set2 | 0.00 | 0.15 | 3439.82 | 82.97 | 1.19E-21 | 57 |
| M_0.2_F_lph_Set3 | 0.00 | 0.15 | 3447.01 | 90.16 | 2.02E-23 | 52 |
| M_0.6_F_ph_Set1 | 0.00 | 0.15 | 3447.70 | 90.85 | 1.86E-23 | 55 |
| M_0.9_F_lqpth_Set1 | 0.00 | 0.15 | 3450.43 | 93.58 | 6.80E-24 | 60 |
| M_0.8_F_lqpt_Set2 | 0.00 | 0.15 | 3458.16 | 101.31 | 1.25E-25 | 61 |
| M_0.7_F_lpt_Set2 | 0.00 | 0.15 | 3473.26 | 116.41 | 5.83E-29 | 67 |
| M_0.7_F_th_Set2 | 0.00 | 0.15 | 3478.88 | 122.03 | 3.47E-30 | 66 |
| M_0.7_F_lqpt_Set2 | 0.00 | 0.15 | 3479.73 | 122.88 | 2.34E-30 | 68 |
| M_0.8_F_qth_Set1 | 0.00 | 0.15 | 3480.49 | 123.64 | 1.76E-30 | 69 |
| M_0.7_F_qpt_Set2 | 0.00 | 0.15 | 3485.38 | 128.53 | 1.37E-31 | 69 |
| M_0.7_F_pt_Set2 | 0.00 | 0.15 | 3490.04 | 133.19 | 1.30E-32 | 70 |
| M_0.4_F_t_Set3 | 0.00 | 0.15 | 3501.87 | 145.02 | 2.68E-35 | 77 |
| M_0.4_F_lt_Set3 | 0.00 | 0.15 | 3501.87 | 145.02 | 2.70E-35 | 77 |
| M_0.3_F_ph_Set2 | 0.00 | 0.15 | 3501.92 | 145.07 | 2.50E-35 | 67 |
| M_0.2_F_ph_Set2 | 0.00 | 0.15 | 3511.74 | 154.89 | 1.75E-37 | 71 |
| M_0.2_F_qh_Set2 | 0.00 | 0.15 | 3524.41 | 167.56 | 3.09E-40 | 73 |
| M_0.2_F_lh_Set2 | 0.00 | 0.15 | 3535.93 | 179.08 | 9.70E-43 | 75 |
| M_0.6_F_lqt_Set2 | 0.00 | 0.15 | 3538.12 | 181.27 | 4.36E-43 | 81 |
| M_0.2_F_h_Set1 | 0.00 | 0.15 | 3604.49 | 247.64 | 1.24E-57 | 89 |
| M_0.3_F_lph_Set1 | 0.00 | 0.15 | 3613.88 | 257.03 | 1.24E-59 | 88 |
| M_0.3_F_lqph_Set1 | 0.00 | 0.15 | 3623.84 | 266.99 | 8.60E-62 | 89 |
| M_0.3_F_ph_Set1 | 0.00 | 0.15 | 3646.11 | 289.26 | 1.23E-66 | 92 |
| M_0.9_F_lt_Set1 | 0.00 | 0.16 | 3411.32 | 54.47 | 1.95E-15 | 52 |
| M_0.8_F_lqpth_Set2 | 0.00 | 0.16 | 3416.09 | 59.24 | 1.73E-16 | 50 |
| M_0.9_F_qpt_Set2 | 0.00 | 0.16 | 3416.74 | 59.89 | 1.37E-16 | 49 |
| M_0.9_F_lqpt_Set2 | 0.00 | 0.16 | 3428.58 | 71.73 | 3.72E-19 | 52 |
| M_0.9_F_lqpt_Set1 | 0.00 | 0.16 | 3432.46 | 75.61 | 5.35E-20 | 57 |
| M_0.9_F_qpt_Set1 | 0.00 | 0.16 | 3436.54 | 79.69 | 6.83E-21 | 58 |
| M_0.7_F_lqth_Set2 | 0.00 | 0.16 | 3456.49 | 99.64 | 2.62E-25 | 62 |
| M_0.2_F_qph_Set3 | 0.00 | 0.16 | 3458.73 | 101.88 | 5.77E-26 | 55 |
| M_0.8_F_lqth_Set1 | 0.00 | 0.16 | 3460.14 | 103.29 | 4.69E-26 | 65 |
| M_0.8_F_pt_Set1 | 0.00 | 0.16 | 3470.95 | 114.10 | 2.00E-28 | 68 |
| M_0.8_F_t_Set1 | 0.00 | 0.16 | 3471.98 | 115.13 | 1.16E-28 | 68 |
| M_0.2_F_ph_Set3 | 0.00 | 0.16 | 3476.55 | 119.70 | 7.65E-30 | 59 |
| M_0.4_F_ph_Set1 | 0.00 | 0.16 | 3481.71 | 124.86 | 6.53E-31 | 65 |
| M_0.8_F_qt_Set1 | 0.00 | 0.16 | 3482.82 | 125.97 | 5.24E-31 | 70 |
| M_0.8_F_lt_Set1 | 0.00 | 0.16 | 3485.56 | 128.71 | 1.32E-31 | 71 |
| M_0.8_F_lqt_Set1 | 0.00 | 0.16 | 3488.08 | 131.23 | 3.86E-32 | 71 |
| M_0.4_F_qph_Set1 | 0.00 | 0.16 | 3488.63 | 131.79 | 2.09E-32 | 66 |
| M_0.4_F_lqph_Set1 | 0.00 | 0.16 | 3501.89 | 145.04 | 2.80E-35 | 69 |
| M_0.2_F_qph_Set2 | 0.00 | 0.16 | 3523.74 | 166.89 | 4.39E-40 | 73 |
| M_0.1_F_lph_Set3 | 0.00 | 0.16 | 3533.22 | 176.37 | 3.65E-42 | 72 |
| M_0.2_F_lph_Set2 | 0.00 | 0.16 | 3547.04 | 190.19 | 3.83E-45 | 77 |
| M_0.6_F_lqpth_Set2 | 0.00 | 0.16 | 3587.05 | 230.20 | 1.07E-53 | 88 |
| M_0.2_F_lh_Set1 | 0.00 | 0.16 | 3604.49 | 247.64 | 1.25E-57 | 89 |
| M_0.6_F_lpth_Set2 | 0.00 | 0.16 | 3611.21 | 254.36 | 6.07E-59 | 91 |
| M_0.6_F_pth_Set2 | 0.00 | 0.16 | 3619.37 | 262.52 | 1.01E-60 | 92 |
| M_0.2_F_ph_Set1 | 0.00 | 0.16 | 3754.99 | 398.14 | 2.64E-90 | 105 |
| M_0.5_F_ph_Set1 | 0.00 | 0.18 | 3430.34 | 73.49 | 1.01E-19 | 52 |
| M_0.9_F_pth_Set1 | 0.00 | 0.18 | 3435.31 | 78.46 | 1.28E-20 | 57 |
| M_0.5_F_qph_Set1 | 0.00 | 0.18 | 3455.33 | 98.48 | 3.87E-25 | 58 |
| M_0.6_F_qph_Set1 | 0.00 | 0.18 | 3469.76 | 112.91 | 3.09E-28 | 60 |
| M_0.8_F_lqpt_Set1 | 0.00 | 0.18 | 3482.43 | 125.58 | 6.71E-31 | 70 |
| M_0.8_F_qpt_Set1 | 0.00 | 0.18 | 3483.02 | 126.17 | 4.94E-31 | 70 |
| M_0.8_F_lqpth_Set1 | 0.00 | 0.18 | 3486.09 | 129.24 | 1.09E-31 | 70 |
| M_0.8_F_lpt_Set1 | 0.00 | 0.18 | 3488.10 | 131.25 | 3.86E-32 | 71 |
| M_0.8_F_pth_Set1 | 0.00 | 0.18 | 3497.00 | 140.15 | 4.59E-34 | 72 |
| M_0.7_F_qth_Set2 | 0.00 | 0.18 | 3498.12 | 141.27 | 2.36E-34 | 70 |
| M_0.8_F_th_Set1 | 0.00 | 0.18 | 3501.86 | 145.01 | 3.90E-35 | 73 |
| M_0.8_F_lpth_Set1 | 0.00 | 0.18 | 3508.71 | 151.86 | 1.34E-36 | 74 |
| M_0.4_F_lph_Set1 | 0.00 | 0.18 | 3513.87 | 157.03 | 6.88E-38 | 71 |
| M_0.4_F_qpt_Set3 | 0.00 | 0.18 | 3519.06 | 162.21 | 5.16E-39 | 80 |
| M_0.4_F_lqpt_Set3 | 0.00 | 0.18 | 3519.06 | 162.21 | 5.21E-39 | 80 |
| M_0.1_F_ph_Set3 | 0.00 | 0.18 | 3526.93 | 170.08 | 8.41E-41 | 71 |
| M_0.6_F_th_Set2 | 0.00 | 0.18 | 3589.24 | 232.39 | 3.43E-54 | 88 |
| M_0.6_F_lqth_Set2 | 0.00 | 0.18 | 3597.16 | 240.31 | 6.80E-56 | 89 |
| M_0.6_F_lpt_Set2 | 0.00 | 0.18 | 3600.43 | 243.58 | 1.29E-56 | 90 |
| M_0.2_F_lqh_Set1 | 0.00 | 0.18 | 3666.32 | 309.47 | 4.78E-71 | 96 |
| M_0.2_F_qph_Set1 | 0.00 | 0.18 | 3830.89 | 474.04 | 8.84E-107 | 111 |
| M_0.2_F_lqph_Set1 | 0.00 | 0.18 | 3831.89 | 475.04 | 5.41E-107 | 111 |
| M_0.5_F_lph_Set1 | 0.00 | 0.19 | 3473.75 | 116.90 | 3.83E-29 | 62 |
| M_0.5_F_lqph_Set1 | 0.00 | 0.19 | 3492.97 | 136.12 | 2.61E-33 | 66 |
| M_0.2_F_lqph_Set2 | 0.00 | 0.19 | 3513.22 | 156.37 | 8.52E-38 | 71 |
| M_0.4_F_pt_Set3 | 0.00 | 0.19 | 3519.05 | 162.20 | 5.08E-39 | 80 |
| M_0.4_F_lpt_Set3 | 0.00 | 0.19 | 3519.05 | 162.20 | 5.16E-39 | 80 |
| M_0.6_F_pt_Set2 | 0.00 | 0.19 | 3556.07 | 199.22 | 5.45E-47 | 84 |
| M_0.6_F_t_Set2 | 0.00 | 0.19 | 3558.34 | 201.49 | 1.71E-47 | 84 |
| M_0.6_F_qth_Set2 | 0.00 | 0.19 | 3612.85 | 256.00 | 2.63E-59 | 91 |
| M_0.1_F_lqph_Set2 | 0.00 | 0.19 | 3908.18 | 551.33 | 1.40E-123 | 116 |
| M_0.1_F_ph_Set2 | 0.00 | 0.19 | 3958.10 | 601.25 | 1.99E-134 | 119 |
| M_0.7_F_lt_Set1 | 0.00 | 0.20 | 3552.92 | 196.07 | 2.83E-46 | 85 |
| M_0.6_F_lt_Set2 | 0.00 | 0.20 | 3558.48 | 201.63 | 1.61E-47 | 84 |
| M_0.6_F_qt_Set2 | 0.00 | 0.20 | 3558.54 | 201.69 | 1.58E-47 | 84 |
| M_0.7_F_lqt_Set1 | 0.00 | 0.20 | 3559.92 | 203.07 | 8.81E-48 | 86 |
| M_0.7_F_pt_Set1 | 0.00 | 0.20 | 3583.12 | 226.27 | 7.97E-53 | 89 |
| M_0.7_F_lqpt_Set1 | 0.00 | 0.20 | 3585.16 | 228.31 | 2.99E-53 | 89 |
| M_0.2_F_lph_Set1 | 0.00 | 0.20 | 3731.18 | 374.33 | 3.94E-85 | 103 |
| M_0.1_F_lqh_Set2 | 0.00 | 0.20 | 3810.05 | 453.20 | 2.80E-102 | 109 |
| M_0.1_F_lph_Set2 | 0.00 | 0.20 | 3977.35 | 620.50 | 1.32E-138 | 120 |
| M_0.7_F_qpt_Set1 | 0.00 | 0.21 | 3533.65 | 176.80 | 4.52E-42 | 82 |
| M_0.7_F_t_Set1 | 0.00 | 0.21 | 3552.29 | 195.44 | 3.82E-46 | 85 |
| M_0.1_F_qph_Set2 | 0.00 | 0.21 | 3849.89 | 493.05 | 6.29E-111 | 112 |
| M_0.1_F_lh_Set1 | 0.00 | 0.21 | 4395.38 | 1038.53 | 2.19E-229 | 138 |
| M_0.7_F_qth_Set1 | 0.00 | 0.22 | 3541.09 | 184.24 | 1.10E-43 | 83 |
| M_0.7_F_lqth_Set1 | 0.00 | 0.22 | 3547.59 | 190.74 | 4.33E-45 | 84 |
| M_0.7_F_qt_Set1 | 0.00 | 0.22 | 3560.73 | 203.88 | 5.75E-48 | 86 |
| M_0.7_F_lpt_Set1 | 0.00 | 0.22 | 3590.43 | 233.58 | 2.09E-54 | 90 |
| M_0.1_F_h_Set2 | 0.00 | 0.22 | 3774.03 | 417.18 | 1.82E-94 | 106 |
| M_0.1_F_lh_Set2 | 0.00 | 0.22 | 3774.03 | 417.18 | 1.84E-94 | 106 |
| M_0.1_F_qh_Set2 | 0.00 | 0.22 | 3774.03 | 417.18 | 1.85E-94 | 106 |
| M_0.4_F_pth_Set3 | 0.00 | 0.23 | 3550.85 | 194.00 | 6.53E-46 | 85 |
| M_0.4_F_lpth_Set3 | 0.00 | 0.23 | 3550.85 | 194.00 | 6.60E-46 | 85 |
| M_0.7_F_lqpth_Set1 | 0.00 | 0.23 | 3562.24 | 205.39 | 2.87E-48 | 86 |
| M_0.6_F_qpt_Set2 | 0.00 | 0.23 | 3568.94 | 212.09 | 8.95E-50 | 86 |
| M_0.6_F_lqpt_Set2 | 0.00 | 0.23 | 3569.88 | 213.03 | 5.66E-50 | 86 |
| M_0.4_F_lqpth_Set3 | 0.00 | 0.23 | 3573.81 | 216.96 | 6.81E-51 | 88 |
| M_0.1_F_ph_Set1 | 0.00 | 0.23 | 4126.22 | 769.37 | 6.17E-171 | 129 |
| M_0.1_F_lqh_Set1 | 0.00 | 0.23 | 4612.11 | 1255.27 | 1.91E-276 | 143 |
| M_0.4_F_th_Set3 | 0.00 | 0.24 | 3506.87 | 150.02 | 2.26E-36 | 78 |
| M_0.4_F_qth_Set3 | 0.00 | 0.24 | 3506.87 | 150.02 | 2.31E-36 | 78 |
| M_0.4_F_lqth_Set3 | 0.00 | 0.24 | 3506.87 | 150.02 | 2.33E-36 | 78 |
| M_0.7_F_lpth_Set1 | 0.00 | 0.24 | 3548.05 | 191.20 | 3.44E-45 | 84 |
| M_0.7_F_pth_Set1 | 0.00 | 0.24 | 3554.39 | 197.54 | 1.43E-46 | 85 |
| M_0.1_F_h_Set1 | 0.00 | 0.24 | 4395.38 | 1038.53 | 2.17E-229 | 138 |
| M_0.1_F_qh_Set1 | 0.00 | 0.24 | 4395.38 | 1038.53 | 2.20E-229 | 138 |
| M_0.1_F_lqph_Set1 | 0.00 | 0.24 | 4766.06 | 1409.21 | 7.21168398673543e-310 | 146 |
| M_0.1_F_lph_Set1 | 0.00 | 0.24 | 4822.55 | 1465.70 | 3.85371203756172e-322 | 147 |
| M_0.1_F_qph_Set1 | 0.00 | 0.24 | 5176.69 | 1819.84 | 0 | 152 |
| M_0.7_F_th_Set1 | 0.00 | 0.25 | 3534.15 | 177.30 | 3.45E-42 | 82 |
| M_0.5_F_qt_Set2 | 0.00 | 0.25 | 3649.06 | 292.21 | 3.20E-67 | 99 |
| M_0.5_F_lt_Set2 | 0.00 | 0.25 | 3649.67 | 292.82 | 2.34E-67 | 99 |
| M_0.5_F_lqt_Set2 | 0.00 | 0.25 | 3669.58 | 312.73 | 1.14E-71 | 101 |
| M_0.3_F_pt_Set3 | 0.00 | 0.26 | 3631.76 | 274.91 | 1.60E-63 | 98 |
| M_0.3_F_lpt_Set3 | 0.00 | 0.26 | 3631.76 | 274.91 | 1.62E-63 | 98 |
| M_0.5_F_t_Set2 | 0.00 | 0.26 | 3649.67 | 292.82 | 2.32E-67 | 99 |
| M_0.5_F_qpt_Set2 | 0.00 | 0.26 | 3688.36 | 331.51 | 9.62E-76 | 103 |
| M_0.5_F_th_Set2 | 0.00 | 0.26 | 3690.19 | 333.34 | 3.80E-76 | 103 |
| M_0.5_F_lqth_Set2 | 0.00 | 0.26 | 3700.44 | 343.59 | 2.33E-78 | 104 |
| M_0.5_F_lqpt_Set2 | 0.00 | 0.26 | 3700.74 | 343.89 | 2.00E-78 | 104 |
| M_0.5_F_qth_Set2 | 0.00 | 0.26 | 3735.84 | 378.99 | 4.74E-86 | 107 |
| M_0.5_F_pt_Set2 | 0.00 | 0.27 | 3668.98 | 312.13 | 1.52E-71 | 101 |
| M_0.5_F_lpth_Set2 | 0.00 | 0.27 | 3759.85 | 403.00 | 2.95E-91 | 109 |
| M_0.5_F_pth_Set2 | 0.00 | 0.27 | 3761.37 | 404.52 | 1.36E-91 | 109 |
| M_0.4_F_qt_Set2 | 0.00 | 0.27 | 3925.88 | 569.03 | 2.30E-127 | 122 |
| M_0.4_F_lqt_Set2 | 0.00 | 0.27 | 3925.88 | 569.03 | 2.33E-127 | 122 |
| M_0.4_F_t_Set2 | 0.00 | 0.27 | 3945.40 | 588.55 | 1.31E-131 | 123 |
| M_0.4_F_lt_Set2 | 0.00 | 0.27 | 3945.40 | 588.55 | 1.32E-131 | 123 |
| M_0.3_F_qpt_Set3 | 0.00 | 0.29 | 3632.21 | 275.36 | 1.30E-63 | 98 |
| M_0.5_F_lpt_Set2 | 0.00 | 0.29 | 3679.34 | 322.49 | 8.71E-74 | 102 |
| M_0.6_F_t_Set1 | 0.00 | 0.29 | 3706.20 | 349.35 | 1.33E-79 | 105 |
| M_0.5_F_lqpth_Set2 | 0.00 | 0.29 | 3760.06 | 403.21 | 2.65E-91 | 109 |
| M_0.6_F_qt_Set1 | 0.00 | 0.29 | 3794.79 | 437.94 | 7.87E-99 | 112 |
| M_0.6_F_qth_Set1 | 0.00 | 0.29 | 3795.05 | 438.20 | 7.17E-99 | 112 |
| M_0.3_F_qt_Set3 | 0.00 | 0.30 | 3605.04 | 248.19 | 1.01E-57 | 95 |
| M_0.3_F_t_Set3 | 0.00 | 0.30 | 3605.51 | 248.66 | 7.89E-58 | 95 |
| M_0.3_F_lt_Set3 | 0.00 | 0.30 | 3605.51 | 248.66 | 7.96E-58 | 95 |
| M_0.3_F_lqt_Set3 | 0.00 | 0.30 | 3605.51 | 248.66 | 8.10E-58 | 95 |
| M_0.6_F_lt_Set1 | 0.00 | 0.30 | 3742.07 | 385.22 | 2.19E-87 | 108 |
| M_0.6_F_lqt_Set1 | 0.00 | 0.30 | 3755.75 | 398.90 | 2.40E-90 | 109 |
| M_0.6_F_lqth_Set1 | 0.00 | 0.30 | 3795.81 | 438.96 | 4.96E-99 | 112 |
| M_0.6_F_lqpt_Set1 | 0.00 | 0.30 | 3808.16 | 451.31 | 1.03E-101 | 113 |
| M_0.3_F_lqpt_Set3 | 0.00 | 0.31 | 3622.29 | 265.44 | 1.86E-61 | 97 |
| M_0.6_F_qpt_Set1 | 0.00 | 0.31 | 3794.69 | 437.84 | 8.50E-99 | 112 |
| M_0.6_F_th_Set1 | 0.00 | 0.31 | 3795.59 | 438.74 | 5.35E-99 | 112 |
| M_0.6_F_lpt_Set1 | 0.00 | 0.31 | 3807.69 | 450.84 | 1.28E-101 | 113 |
| M_0.6_F_pth_Set1 | 0.00 | 0.31 | 3809.87 | 453.02 | 4.35E-102 | 113 |
| M_0.6_F_lpth_Set1 | 0.00 | 0.31 | 3838.79 | 481.94 | 2.31E-108 | 115 |
| M_0.6_F_lqpth_Set1 | 0.00 | 0.31 | 3855.63 | 498.78 | 5.12E-112 | 116 |
| M_0.6_F_pt_Set1 | 0.00 | 0.32 | 3824.70 | 467.85 | 2.53E-105 | 114 |
| M_0.4_F_lqpt_Set2 | 0.00 | 0.32 | 3966.19 | 609.34 | 4.22E-136 | 124 |
| M_0.4_F_qpt_Set2 | 0.00 | 0.32 | 4007.67 | 650.82 | 4.10E-145 | 126 |
| M_0.4_F_lpt_Set2 | 0.00 | 0.32 | 4031.00 | 674.15 | 3.50E-150 | 127 |
| M_0.4_F_pt_Set2 | 0.00 | 0.32 | 4031.63 | 674.78 | 2.52E-150 | 127 |
| M_0.5_F_lqpt_Set1 | 0.00 | 0.32 | 4128.54 | 771.69 | 2.53E-171 | 131 |
| M_0.5_F_qpt_Set1 | 0.00 | 0.32 | 4185.98 | 829.13 | 8.44E-184 | 133 |
| M_0.5_F_qt_Set1 | 0.00 | 0.32 | 4247.19 | 890.34 | 4.20E-197 | 135 |
| M_0.5_F_lqt_Set1 | 0.00 | 0.32 | 4247.19 | 890.34 | 4.27E-197 | 135 |
| M_0.5_F_th_Set1 | 0.00 | 0.32 | 4426.77 | 1069.92 | 4.28E-236 | 140 |
| M_0.5_F_qth_Set1 | 0.00 | 0.32 | 4426.77 | 1069.92 | 4.37E-236 | 140 |
| M_0.5_F_lqth_Set1 | 0.00 | 0.32 | 4426.77 | 1069.92 | 4.42E-236 | 140 |
| M_0.5_F_pth_Set1 | 0.00 | 0.32 | 4427.33 | 1070.48 | 3.32E-236 | 140 |
| M_0.5_F_lpth_Set1 | 0.00 | 0.32 | 4467.46 | 1110.61 | 6.51E-245 | 141 |
| M_0.4_F_qth_Set2 | 0.00 | 0.33 | 3984.45 | 627.60 | 4.54E-140 | 125 |
| M_0.4_F_lqth_Set2 | 0.00 | 0.33 | 3984.45 | 627.60 | 4.59E-140 | 125 |
| M_0.4_F_pth_Set2 | 0.00 | 0.33 | 4051.70 | 694.85 | 1.14E-154 | 128 |
| M_0.4_F_lpth_Set2 | 0.00 | 0.33 | 4053.04 | 696.19 | 5.87E-155 | 128 |
| M_0.4_F_th_Set2 | 0.00 | 0.33 | 4102.74 | 745.89 | 9.16E-166 | 130 |
| M_0.5_F_lt_Set1 | 0.00 | 0.33 | 4102.74 | 745.90 | 9.67E-166 | 130 |
| M_0.5_F_lpt_Set1 | 0.00 | 0.33 | 4213.57 | 856.72 | 8.57E-190 | 134 |
| M_0.5_F_t_Set1 | 0.00 | 0.33 | 4247.19 | 890.34 | 4.13E-197 | 135 |
| M_0.5_F_pt_Set1 | 0.00 | 0.33 | 4280.55 | 923.70 | 2.41E-204 | 136 |
| M_0.5_F_lqpth_Set1 | 0.00 | 0.33 | 4313.49 | 956.64 | 1.77E-211 | 137 |
| M_0.3_F_qth_Set3 | 0.00 | 0.34 | 3639.05 | 282.20 | 4.27E-65 | 99 |
| M_0.3_F_th_Set3 | 0.00 | 0.34 | 3648.87 | 292.02 | 3.10E-67 | 100 |
| M_0.3_F_lqth_Set3 | 0.00 | 0.34 | 3648.87 | 292.02 | 3.17E-67 | 100 |
| M_0.4_F_lqpth_Set2 | 0.00 | 0.34 | 4129.49 | 772.64 | 1.48E-171 | 131 |
| M_0.3_F_pth_Set3 | 0.00 | 0.35 | 3647.34 | 290.49 | 6.77E-67 | 100 |
| M_0.3_F_lpth_Set3 | 0.00 | 0.35 | 3647.34 | 290.49 | 6.82E-67 | 100 |
| M_0.3_F_lqpth_Set3 | 0.00 | 0.36 | 3658.98 | 302.13 | 2.03E-69 | 101 |
| M_0.4_F_lqpt_Set1 | 0.00 | 0.37 | 6183.24 | 2826.39 | 0 | 161 |
| M_0.4_F_pt_Set1 | 0.00 | 0.37 | 6587.31 | 3230.46 | 0 | 163 |
| M_0.4_F_t_Set1 | 0.00 | 0.37 | 7097.01 | 3740.16 | 0 | 165 |
| M_0.4_F_qt_Set1 | 0.00 | 0.37 | 7097.01 | 3740.16 | 0 | 165 |
| M_0.4_F_lqt_Set1 | 0.00 | 0.37 | 7097.01 | 3740.16 | 0 | 165 |
| M_0.4_F_lpt_Set1 | 0.00 | 0.37 | 7763.72 | 4406.87 | 0 | 167 |
| M_0.4_F_qpt_Set1 | 0.00 | 0.37 | 7765.98 | 4409.13 | 0 | 167 |
| M_0.4_F_lt_Set1 | 0.00 | 0.37 | 8676.32 | 5319.47 | 0 | 169 |
| M_0.4_F_pth_Set1 | 0.00 | 0.38 | 7764.48 | 4407.63 | 0 | 167 |
| M_0.4_F_th_Set1 | 0.00 | 0.38 | 8673.71 | 5316.86 | 0 | 169 |
| M_0.4_F_qth_Set1 | 0.00 | 0.38 | 8673.71 | 5316.86 | 0 | 169 |
| M_0.4_F_lqth_Set1 | 0.00 | 0.38 | 8673.71 | 5316.86 | 0 | 169 |
| M_0.4_F_lqpth_Set1 | 0.00 | 0.38 | 9991.00 | 6634.15 | 0 | 171 |
| M_0.4_F_lpth_Set1 | 0.00 | 0.38 | 12057.77 | 8700.92 | 0 | 173 |
| M_0.3_F_lt_Set2 | 0.00 | 0.43 | 4350.22 | 993.37 | 1.55E-219 | 139 |
| M_0.3_F_lqpt_Set2 | 0.00 | 0.43 | 4350.48 | 993.63 | 1.40E-219 | 139 |
| M_0.3_F_t_Set2 | 0.00 | 0.43 | 4431.63 | 1074.78 | 3.22E-237 | 141 |
| M_0.3_F_qpt_Set2 | 0.00 | 0.43 | 4476.77 | 1119.92 | 5.26E-247 | 142 |
| M_0.3_F_lpt_Set2 | 0.00 | 0.43 | 4522.16 | 1165.31 | 7.28E-257 | 143 |
| M_0.3_F_pt_Set2 | 0.00 | 0.43 | 4570.57 | 1213.72 | 2.21E-267 | 144 |
| M_0.2_F_t_Set3 | 0.00 | 0.44 | 3825.73 | 468.88 | 1.13E-105 | 116 |
| M_0.2_F_lt_Set3 | 0.00 | 0.44 | 3825.73 | 468.88 | 1.14E-105 | 116 |
| M_0.2_F_qt_Set3 | 0.00 | 0.44 | 3825.73 | 468.88 | 1.14E-105 | 116 |
| M_0.2_F_pt_Set3 | 0.00 | 0.44 | 3825.73 | 468.88 | 1.15E-105 | 116 |
| M_0.2_F_lpt_Set3 | 0.00 | 0.44 | 3825.73 | 468.88 | 1.16E-105 | 116 |
| M_0.2_F_lqt_Set3 | 0.00 | 0.44 | 3859.08 | 502.23 | 6.64E-113 | 118 |
| M_0.2_F_qpt_Set3 | 0.00 | 0.44 | 3859.08 | 502.23 | 6.69E-113 | 118 |
| M_0.2_F_lqpt_Set3 | 0.00 | 0.44 | 3859.12 | 502.27 | 6.60E-113 | 118 |
| M_0.3_F_qt_Set2 | 0.00 | 0.44 | 4431.63 | 1074.78 | 3.27E-237 | 141 |
| M_0.3_F_lqt_Set2 | 0.00 | 0.44 | 4431.63 | 1074.78 | 3.31E-237 | 141 |
| M_0.3_F_t_Set1 | 0.00 | 0.45 | NA | NA | NA | 188 |
| M_0.3_F_lt_Set1 | 0.00 | 0.45 | NA | NA | NA | 188 |
| M_0.3_F_qt_Set1 | 0.00 | 0.45 | NA | NA | NA | 188 |
| M_0.3_F_lqt_Set1 | 0.00 | 0.45 | NA | NA | NA | 188 |
| M_0.2_F_lqth_Set3 | 0.00 | 0.46 | 3888.16 | 531.31 | 3.27E-119 | 120 |
| M_0.2_F_th_Set3 | 0.00 | 0.46 | 3945.35 | 588.50 | 1.22E-131 | 123 |
| M_0.2_F_qth_Set3 | 0.00 | 0.46 | 3945.35 | 588.50 | 1.24E-131 | 123 |
| M_0.2_F_pth_Set3 | 0.00 | 0.46 | 3945.35 | 588.50 | 1.24E-131 | 123 |
| M_0.2_F_lqpth_Set3 | 0.00 | 0.46 | 4007.88 | 651.03 | 3.30E-145 | 126 |
| M_0.3_F_pth_Set2 | 0.00 | 0.47 | 4566.85 | 1210.00 | 1.45E-266 | 144 |
| M_0.3_F_th_Set2 | 0.00 | 0.47 | 4567.15 | 1210.30 | 1.23E-266 | 144 |
| M_0.3_F_qth_Set2 | 0.00 | 0.47 | 4567.15 | 1210.30 | 1.25E-266 | 144 |
| M_0.3_F_lqth_Set2 | 0.00 | 0.47 | 4567.15 | 1210.30 | 1.26E-266 | 144 |
| M_0.3_F_lpth_Set2 | 0.00 | 0.47 | 4792.79 | 1435.94 | 1.26496163860026e-315 | 148 |
| M_0.3_F_lqpth_Set2 | 0.00 | 0.47 | 4859.47 | 1502.62 | 0 | 149 |
| M_0.3_F_pth_Set1 | 0.00 | 0.47 | NA | NA | NA | 194 |
| M_0.3_F_lpth_Set1 | 0.00 | 0.47 | NA | NA | NA | 198 |
| M_0.3_F_lqpth_Set1 | 0.00 | 0.47 | NA | NA | NA | 197 |
| M_0.2_F_lpth_Set3 | 0.00 | 0.48 | 3945.35 | 588.50 | 1.25E-131 | 123 |
| M_0.3_F_pt_Set1 | 0.00 | 0.48 | NA | NA | NA | 186 |
| M_0.3_F_lpt_Set1 | 0.00 | 0.48 | NA | NA | NA | 195 |
| M_0.1_F_t_Set3 | 0.00 | 0.49 | 4080.72 | 723.87 | 4.61E-161 | 130 |
| M_0.1_F_lt_Set3 | 0.00 | 0.49 | 4080.72 | 723.87 | 4.64E-161 | 130 |
| M_0.1_F_qt_Set3 | 0.00 | 0.49 | 4080.72 | 723.87 | 4.66E-161 | 130 |
| M_0.1_F_pt_Set3 | 0.00 | 0.49 | 4080.72 | 723.87 | 4.68E-161 | 130 |
| M_0.1_F_lqt_Set3 | 0.00 | 0.49 | 4080.72 | 723.87 | 4.70E-161 | 130 |
| M_0.1_F_lpt_Set3 | 0.00 | 0.49 | 4080.72 | 723.87 | 4.72E-161 | 130 |
| M_0.1_F_qpt_Set3 | 0.00 | 0.49 | 4080.72 | 723.87 | 4.73E-161 | 130 |
| M_0.1_F_lqpt_Set3 | 0.00 | 0.49 | 4080.72 | 723.87 | 4.75E-161 | 130 |
| M_0.1_F_th_Set3 | 0.00 | 0.49 | 4186.53 | 829.68 | 4.96E-184 | 134 |
| M_0.1_F_qth_Set3 | 0.00 | 0.49 | 4186.53 | 829.68 | 5.02E-184 | 134 |
| M_0.1_F_pth_Set3 | 0.00 | 0.49 | 4186.53 | 829.68 | 5.03E-184 | 134 |
| M_0.1_F_lqth_Set3 | 0.00 | 0.49 | 4186.53 | 829.68 | 5.04E-184 | 134 |
| M_0.1_F_lpth_Set3 | 0.00 | 0.49 | 4186.53 | 829.68 | 5.05E-184 | 134 |
| M_0.1_F_lqpth_Set3 | 0.00 | 0.49 | 4186.53 | 829.68 | 5.05E-184 | 134 |
| M_0.3_F_th_Set1 | 0.00 | 0.49 | NA | NA | NA | 193 |
| M_0.3_F_qth_Set1 | 0.00 | 0.49 | NA | NA | NA | 193 |
| M_0.3_F_lqth_Set1 | 0.00 | 0.49 | NA | NA | NA | 193 |
| M_0.3_F_qpt_Set1 | 0.00 | 0.53 | NA | NA | NA | 188 |
| M_0.3_F_lqpt_Set1 | 0.00 | 0.53 | NA | NA | NA | 191 |
| M_0.2_F_t_Set2 | 0.00 | 0.54 | 6341.85 | 2985.00 | 0 | 162 |
| M_0.2_F_lt_Set2 | 0.00 | 0.54 | 6341.85 | 2985.00 | 0 | 162 |
| M_0.2_F_qt_Set2 | 0.00 | 0.54 | 6341.85 | 2985.00 | 0 | 162 |
| M_0.2_F_lqt_Set2 | 0.00 | 0.54 | 6341.85 | 2985.00 | 0 | 162 |
| M_0.2_F_qpt_Set2 | 0.00 | 0.54 | 6341.85 | 2985.00 | 0 | 162 |
| M_0.2_F_lqpt_Set2 | 0.00 | 0.54 | 6341.85 | 2985.00 | 0 | 162 |
| M_0.2_F_pt_Set2 | 0.00 | 0.54 | 7377.11 | 4020.26 | 0 | 166 |
| M_0.2_F_lpt_Set2 | 0.00 | 0.55 | 7377.11 | 4020.26 | 0 | 166 |
| M_0.1_F_lpt_Set2 | 0.00 | 0.55 | NA | NA | NA | 185 |
| M_0.1_F_lqt_Set2 | 0.00 | 0.56 | NA | NA | NA | 187 |
| M_0.2_F_pth_Set2 | 0.00 | 0.57 | 12022.90 | 8666.05 | 0 | 173 |
| M_0.2_F_th_Set2 | 0.00 | 0.57 | 12023.04 | 8666.19 | 0 | 173 |
| M_0.2_F_qth_Set2 | 0.00 | 0.57 | 12023.04 | 8666.19 | 0 | 173 |
| M_0.2_F_lqth_Set2 | 0.00 | 0.57 | 12023.04 | 8666.19 | 0 | 173 |
| M_0.2_F_lqpth_Set2 | 0.00 | 0.57 | 12023.04 | 8666.19 | 0 | 173 |
| M_0.2_F_pth_Set1 | 0.00 | 0.58 | NA | NA | NA | 212 |
| M_0.2_F_lpth_Set1 | 0.00 | 0.58 | NA | NA | NA | 212 |
| M_0.2_F_lpth_Set2 | 0.00 | 0.59 | 12022.90 | 8666.05 | 0 | 173 |
| M_0.1_F_t_Set1 | 0.00 | 0.59 | NA | NA | NA | 227 |
| M_0.1_F_lt_Set1 | 0.00 | 0.59 | NA | NA | NA | 227 |
| M_0.1_F_qt_Set1 | 0.00 | 0.59 | NA | NA | NA | 227 |
| M_0.1_F_pt_Set1 | 0.00 | 0.59 | NA | NA | NA | 227 |
| M_0.1_F_lqt_Set1 | 0.00 | 0.59 | NA | NA | NA | 227 |
| M_0.1_F_lpt_Set1 | 0.00 | 0.59 | NA | NA | NA | 227 |
| M_0.2_F_t_Set1 | 0.00 | 0.59 | NA | NA | NA | 210 |
| M_0.2_F_lt_Set1 | 0.00 | 0.59 | NA | NA | NA | 210 |
| M_0.2_F_qt_Set1 | 0.00 | 0.59 | NA | NA | NA | 210 |
| M_0.2_F_pt_Set1 | 0.00 | 0.59 | NA | NA | NA | 210 |
| M_0.2_F_th_Set1 | 0.00 | 0.59 | NA | NA | NA | 212 |
| M_0.2_F_lqt_Set1 | 0.00 | 0.59 | NA | NA | NA | 210 |
| M_0.2_F_lpt_Set1 | 0.00 | 0.59 | NA | NA | NA | 210 |
| M_0.2_F_qth_Set1 | 0.00 | 0.59 | NA | NA | NA | 212 |
| M_0.2_F_lqth_Set1 | 0.00 | 0.59 | NA | NA | NA | 212 |
| M_0.1_F_t_Set2 | 0.00 | 0.60 | NA | NA | NA | 187 |
| M_0.1_F_lt_Set2 | 0.00 | 0.60 | NA | NA | NA | 187 |
| M_0.1_F_qt_Set2 | 0.00 | 0.60 | NA | NA | NA | 187 |
| M_0.1_F_pt_Set2 | 0.00 | 0.60 | NA | NA | NA | 192 |
| M_0.1_F_qpt_Set2 | 0.00 | 0.60 | NA | NA | NA | 187 |
| M_0.1_F_lqpt_Set2 | 0.00 | 0.60 | NA | NA | NA | 187 |
| M_0.2_F_qpt_Set1 | 0.00 | 0.60 | NA | NA | NA | 218 |
| M_0.2_F_lqpt_Set1 | 0.00 | 0.60 | NA | NA | NA | 210 |
| M_0.1_F_qpt_Set1 | 0.00 | 0.62 | NA | NA | NA | 227 |
| M_0.1_F_lqpt_Set1 | 0.00 | 0.62 | NA | NA | NA | 229 |
| M_0.1_F_lpth_Set2 | 0.00 | 0.62 | NA | NA | NA | 198 |
| M_0.2_F_lqpth_Set1 | 0.00 | 0.62 | NA | NA | NA | 216 |
| M_0.1_F_lqth_Set2 | 0.01 | 0.63 | NA | NA | NA | 190 |
| M_0.1_F_th_Set1 | 0.00 | 0.64 | NA | NA | NA | 226 |
| M_0.1_F_qth_Set1 | 0.00 | 0.64 | NA | NA | NA | 226 |
| M_0.1_F_pth_Set1 | 0.00 | 0.64 | NA | NA | NA | 226 |
| M_0.1_F_lqth_Set1 | 0.00 | 0.64 | NA | NA | NA | 226 |
| M_0.1_F_lpth_Set1 | 0.00 | 0.64 | NA | NA | NA | 226 |
| M_0.1_F_lqpth_Set1 | 0.01 | 0.66 | NA | NA | NA | 226 |
| M_0.1_F_th_Set2 | 0.01 | 0.68 | NA | NA | NA | 190 |
| M_0.1_F_qth_Set2 | 0.01 | 0.68 | NA | NA | NA | 190 |
| M_0.1_F_pth_Set2 | 0.00 | 0.68 | NA | NA | NA | 191 |
| M_0.1_F_lqpth_Set2 | 0.00 | 0.68 | NA | NA | NA | 190 |
